# Nurses’ experiences with inhospital continuous monitoring of vital signs in general wards: A systematic review

**DOI:** 10.1371/journal.pdig.0000949

**Published:** 2025-08-22

**Authors:** Berte van Zeist - de Jonge, Janneke de Man-van Ginkel, Mick Olvers, Kees van den Berge, Laura Kooij, Paul J. T. Rood

**Affiliations:** 1 Department of Gastroenterology and Hepatology, Rijnstate Hospital, Arnhem, the Netherlands; 2 Nursing Sciences, Program in Clinical Health Sciences, Utrecht University, Utrecht, the Netherlands; 3 Department of Nursing Science, Leiden University Medical Center, Leiden, the Netherlands; 4 Department of Nursing, Maastricht University Medical Center, Maastricht, the Netherlands; 5 Regional Emergency Medical Services Brabant Midden-West-Noord, s-Hertogenbosch, the Netherlands; 6 Department of Innovation and Care Transformation, Rijnstate Hospital, Arnhem, the Netherlands; 7 Department of Quality, Research and Development, Rijnstate Hospital, Arnhem, the Netherlands; 8 Research Departments ‘Technology for Health’ and ‘Emergency and Critical Care’, School of Health Studies Nijmegen, HAN University of Applied Sciences, Nijmegen, the Netherlands; 9 Radboud Institute for Health Sciences, Radboud University Medical Center, Nijmegen, the Netherlands; Instituto Politécnico Nacional Escuela Superior de Medicina: Instituto Politecnico Nacional Escuela Superior de Medicina, MEXICO

## Abstract

Recent developments make the continuous monitoring of vital signs outside the critical care setting feasible, and may provide a benefit in terms of improved patient outcomes and cost efficiency. A meta-aggregative systematic review was conducted to provide an overview of the experiences of nurses working with continuous monitoring of vital signs in patients admitted to a hospital general ward. All study designs describing nurses’ experiences in a qualitative manner were included. Relevant studies were identified by searching the electronic databases Pubmed, Cinahl and Embase from 2017 up to September 2024. The search strategy combined ‘nurses’, ‘continuous monitoring/measuring’, ‘vital signs’, and ‘hospital/(general) wards’ as well as synonyms. Of 3066 articles found, nine were included. Four themes were synthesized: 1) Emotional and practical advantages, e.g., patients feel safer and nurses feel more secure, as continuous monitoring detects patients deterioration earlier; 2) practical disadvantages, e.g., reduced nurse-patient interaction and stress related to changing vital signs, potential over-monitoring and data overloading; 3) important aspects related to the implementation process, e.g., training and coaching, properly working technical infrastructure; and 4) alarm strategies and clinical assessment, e.g., reducing unnecessary alarms. We conclude that nurses report varying experiences in working with continuous monitoring of vital signs on the general ward. The advantages of continuous monitoring seem to justify investing in further development of the systems and sensors in order to reduce the practical disadvantages. The findings may facilitate optimal implementation of continuous monitoring of vital signs.

## Author summary

Vital signs, such as blood pressure, body temperature, pulse rate and respiratory rate, are important parameters for monitoring the patients’ health status during hospital admission. Recent developments make the continuous monitoring of vital signs outside the critical care setting feasible, and may provide a benefit in terms of improved patient outcomes and cost efficiency. A paramount step for the successful introduction of this new technology is to determine barriers and facilitators for introduction, and provide the needed oversight for end-users to facilitate proper incorporation of these technologies into daily nursing practice. This review article provides an overview of the experiences of nurses working with continuous monitoring of vital signs in patients admitted to a hospital general ward, based on existing scientific literature that was published between 2017 and 2024. Nurses report varying experiences in working with continuous monitoring of vital signs on the general ward. The advantages of continuous monitoring seem to justify investing in further development of the systems and sensors in order to reduce the practical disadvantages.

## Introduction

Vital signs, such as blood pressure, body temperature, pulse rate and respiratory rate, are important parameters for monitoring the patients’ health status during hospital admission, as changes in vital signs may indicate patients’ deterioration and thereby prevent development of morbidities and long term disabilities. Within hospitals, combined assessment of vital signs including a “track-and-trigger” system for escalation, such as increased frequency of patient’s vital signs or urgent review by a rapid response team are known as *Early Warning Systems* (EWS) [1]. Although these systems provide the language and preconditions for timely escalation in patient care emergencies, they are limited by their intermittent and user-dependent nature. In-depth analyses demonstrate that 46% of the unplanned ICU admissions from the general ward had healthcare worker related causes, mostly due to monitoring failures in clinically deteriorating patients. Improving the monitoring of patients is therefore warranted, which may prevent iatrogenic adverse outcomes such as unplanned ICU-admissions [2,3].

*Continuous Monitoring of Vital Signs* (CMVS) may be a promising solution for these limitations [4–6]. The rapid development in digital health technologies such as wearable, wireless devices and cameras enable monitoring patients on general wards while maintaining or improving mobility and self-care [7,8]. Recent studies concluded that CMVS outside the critical care setting is feasible and may provide a benefit in terms of improved patient outcomes and cost efficiency [5]. The use of CMVS devices seems to be well accepted by patients [9–11], which report to feel safer knowing they are continuously remotely monitored [12]. However, large and well-controlled studies in high-risk populations to evaluate the clinical benefit of continuous monitoring systems are still lacking, which is possibly because most monitoring devices are still in the clinical validation and feasibility testing phases [13].

As end‐users, nurses have the practical expertise and knowledge which is vital to the successful adoption of CMVS ensuring optimal implementation [14]. The infrastructural as well as technical issues, psychological barriers and workload-related concerns are important barriers to comprehensively and holistically adopting digital health technologies by health professionals. Conversely, strategies such as deploying training, evaluating perceived usefulness and willingness to use may enhance the adoption of digital interventions [15]. Currently, such insights in current experiences of general ward nurses with CMVS on the general ward are lacking.

Therefore, the aim of this systematic review was to provide an overview of the experiences of nurses working with continuous monitoring of vital signs in patients admitted to a hospital general ward.

## Methods

### Design

A meta-aggregative systematic review was conducted, adhering to the guideline of the *Johanna Briggs Institute* (JBI) for systematic reviews of qualitative data [16], and reported according to the *Preferred Reporting Items for Systematic reviews and Meta-Analysis* (PRISMA) statement [17].

### Search methods

Relevant studies were identified by searching the electronic databases Pubmed, Cinahl and Embase from inception up to September 2024. The search strategy combined ‘nurses’, ‘(continuous) monitoring/measuring’, ‘vital signs’, and hospital/(general) wards as well as synonyms. The complete search strategy can be found in S1 Text.

### Inclusion and exclusion criteria

All qualitative study designs describing experiences of nurses working with CMVS were considered eligible. Studies describing home monitoring, continuous glucose monitoring, telemetric monitoring (for example on cardiac wards), ICU monitoring, monitoring during pregnancy and delivery and paediatric monitoring were excluded. Lastly, studies with a quality appraisal lower than five out of ten points or those presenting findings unsupported by the data were excluded from the review, according to the JBI guideline [16].

### Search outcome

After removing duplicates, all title and abstracts were screened using the online screening tool Rayyan [18]. Selected studies were retrieved in full-text and assessed based on the in- and exclusion criteria. The reference lists of included studies were manually checked for other relevant studies for this review. Data of the individual studies were summarized using the JBI QARI Data extraction tool for qualitative research [16], including author, method, phenomena of interest, setting, participants, data analysis and key themes.

### Quality appraisal

Methodological quality of all studies was appraised using the JBI appraisal tool for qualitative studies [16], which consists of 10 questions. Quality appraisal of each article was individually checked by two researchers (BZJ and MO or KB) Disagreements were discussed, if needed with a third researcher, until consensus was reached.

### Synthesis

The JBI meta-aggregative approach for qualitative synthesis was followed [16], to enable the formulation of generalizable statements into recommendations for practise [19]. First, all the findings from the included studies were extracted and where possible, illustrated with a quote from the article. Second, all the findings describing similar outcomes were categorized, with each category containing at least two findings. In the third and final step, synthesized findings of at least two categories were developed [19].

The confidence in the output of qualitative research synthesis (ConQual) [20] was determined by assessing the dependability and credibility of each synthesized finding. First, the dependability of the individual studies was assessed using the JBI critical appraisal checklist for statements addressing bracketing, bias, representativity, ethics and congruity with the data. All studies started with a high dependability and were downgraded based on the amount of elements were not met [20]. Second, the credibility of the findings was assessed. All individual findings from the studies were allocated a level of credibility, consisting of three levels: unequivocal, credible and unsupported [20]. Based on the dependability and credibility of each finding, the ConQual score for each synthesized finding was labelled as high, moderate, low or very low. According to the ConQual guideline, all findings that are unsupported by the data were be excluded from this review.

## Results

**Fig 1:**
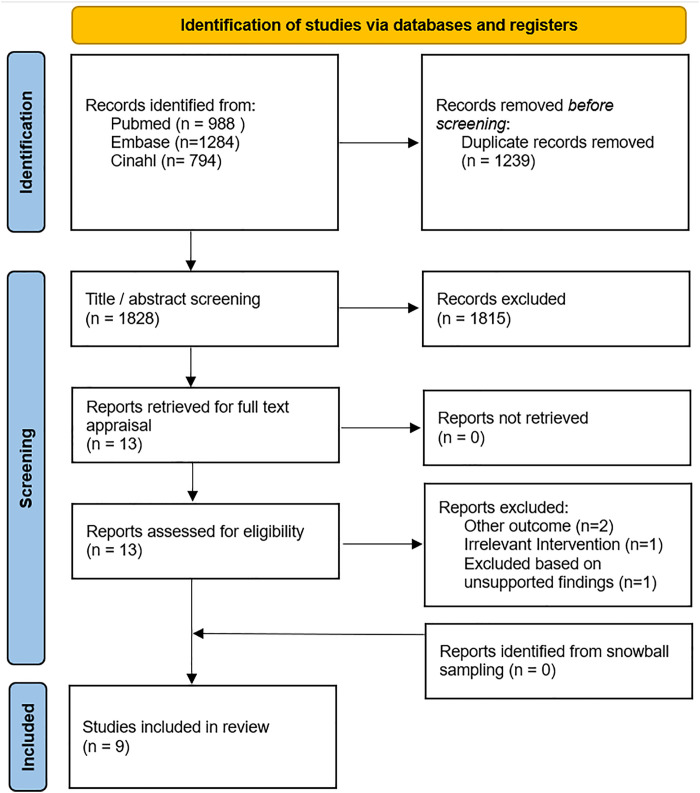
Flowchart of Included Studies.

A total of 3066 articles were identified, after duplicate removal 1828 articles were screened by title and/or abstract. Thirteen articles were retrieved for full text appraisal of which four were excluded based on the in- and exclusion criteria. The selection process is visualized in the flowchart in Fig 1 and S2 Text.

Nine studies were included in the review: seven studies from The Netherlands [21–27], and two studies from the United Kingdom [28]. Five studies had a primary qualitative design [23,26–28]. The others were mixed-methods studies, that included a qualitative element [24,25]. A total of 118 nurses were included in the nine studies, varying from 9 to 21 participants per study. Nurses were predominantly female, ageing between 20 and 60. Four studies were conducted on a surgical ward [23,25,26,28], one on a Covid-19 isolation ward [29], and four studies on combined surgical and internal medicine wards [22,24,27,30]. Eight studies described using wireless, wearable sensors to measure vital signs [22–27,30] and one study wired, non-wearable, CMVS [28] (Table 1).

**Table 1:** Study Characteristics.

Author, Year	Design	Data collection and participants	Phenomena of interest	Setting and monitoring device	Analyses	Key themes
Areia, 2021	Qualitative	One-to-one interviews 15 nurses 3 male, 12 female Years qualified: <1 up to>10	To understand clinical staffs current perceptions of monitoring practices.	Setting: Surgical ward, large teaching hospital UK Wired sensor	Thematic analysis	Finding a balance between continuous and intermittent monitoring
Becking-Verhaar, 2023	Cross-sectional Survey	Survey comprising open and closed questions 58 nurses (51,3%) completed the survey 9 male, 49 female Age 21 – 60 years	Exploring facilitators and barriers for nurses using a wireless device to monitor vital signs continuously in a post-implementation period.	Three general wards in a Dutch tertiary university hospital, surgical and internal medicine wards. Portable device with 3-lead wired electrocardiogram	Qualitative thematic analysis to evaluate responses to the open questions.	Timely signalling and early action. Time Savings and time consumption Patient Comfort and satisfaction Preconditions
Buss, 2023	Qualitative	One-to-one interviews 15 staff members	To explore the ways that staff working on a COVID-19 isolation ward environment experienced the implementation and impact of an ambulatory monitoring system.	Setting: Covid-19 isolation ward in hospital in the UK	Thematic analysis	Adopting innovation to assist patient safety. Patient selection Trust in de AMS Resource management
Kooij, 2022	General Qualitative	Semi-structured interviews 16 nurses 0 male, 16 female Age 22–56 years Work experience: 1 - > 15 years	Provide an overview of factors affecting implementation in continuous monitoring using wireless wearable sensors by evaluating nurses; experiences with its use on the clinical ward.	Setting: Three teaching hospitals in The Netherlands Dec 2019 – July 2020: Mix of surgical and internal medicine patients. Wireless sensor	Deductive approach of directed content analysis	Intervention characteristics, Outer setting, Inner setting, Characteristics of individuals, Process.
Leenen, 2022 (1)	Qualitative study based on the behaviour change wheel	Semi structured interviews. 12 (out of 35) All female Age Median age 27.5 (IQR 23-31.5) Work experience 5.5 (IQR 2-8.5)	To provide insight in de capability, opportunity and motivation of nurses working with CMVS, in order to inform future implementation efforts.	Setting: General surgical ward of a large tertiary teaching hospital Wireless sensor	Deductive thematic analysis.	Learning and coaching on the job Interpretation of vital sign trends Added value for nursing care Management of alarms Integration and compatibility with clinical workflow
Leenen 2023	Mixed-methods sequential explanatory design	Semi-structured interviews 11 nurses surgical ward 10 internal medicine 8 senior nurses 3 male, 18 female	To assess an compare implementation fidelity, technical fidelity, appropriateness, acceptability, usability, adoption and feasibility according to nurses.	Setting: September 2021-july 2022 Surgical and internal ward tertiary teaching hospital in The Netherlands Wireless sensor	Deductive thematic analysis	Prioritizing CMVS The importance of bedside nursing assessment Experiencing CMVS as an added value for patient care Experienced usability of the CMVS system Future perspectives of CMVS on the general ward
Leenen 2022 (2)	Explanatory sequential Mixed Methods	Two semi-structured focus groups with a total of 9 nurses.	To determine feasibility, in terms of acceptability and fidelity, of continuous monitoring on a general surgical ward without the use of alarms, exclusively focusing on regular vital sign trend assessments.	Setting: July-october 2020, surgical ward in a large tertiary teaching hospital in The Netherlands Wireless sensor	Thematic content analysis.	Faster anticipation and action upon changed patient status from insight into vital sign trends. Successful use of the technology Integration in the nursing process Willingness to use the technology Gaining practical Experience Application of alarm strategy for deviating vital signs.
Van Noort 2024	Qualitative	Individual interviews and a focus group 4 nurses, 1 male, 3 female 6 nurses in the focus group, all female	To provide in-depth insight into nurses’ perspectives regarding the use of continuous vital signs monitoring system, and how it can be further innovated to ensure the sustainable and effective use of this technology.	Setting: February and April 2021 Surgical oncology ward of a Dutch academic hospital. Wireless device	Deductive content analysis approach.	Factors related to ICT: Individual factors of healthcare professionals: Human environment: Organizational aspects
Weenk 2020	Randomized controlled trial with qualitative outcome	Semi-structured interviews 20 nurses, No demographics available	To Identify experiences of nurses about the use of VM an HP in daily practice for continuous monitoring of vital signs in the general ward.	Setting: University hospital between April 2015 and august 2016 Surgical and internal medicine patients. Wireless device	Thematic content analysis, saturation may not have been reached.	Positive effects Negative effects Facilitators Barriers

Overall appraisal tool quality of the included studies was high, varying from 7/10 up to 10/10 points. The results of the quality appraisal including dependability scores can be found in Table 2. One study was excluded because of a quality assessment of 3/10 points (S2 Text). According to their study design, all studies start with a high ConQual level of confidence ranking, of which seven studies were downgraded one level because of a moderate dependability score. All synthesized findings were supported by unequivocal and credible data and therefore, the level of confidence was downgraded one level for credibility. The level of confidence for all synthesized findings was assessed as low. (Table 3).

**Table 2:** Methodological Quality.

	Areia, 2022	Becking-Verhaar 2023	Buss, 2023	Kooij, 2022	Leenen, 2022 (1)	Leenen, 2022 (2)	Leenen, 2023	Van Noort, 2024	Weenk, 2020
V1. Congruity philosophical perspective and methodology?	Y	U	Y	Y	Y	Y	Y	Y	U
V2. Congruity methodology and objective^a^	Y	Y	Y	Y	Y	Y	Y	Y	Y
V3. Congruity methodology and methods^a^	Y	Y	Y	Y	Y	Y	Y	Y	Y
V4. Congruity methodology and analysis^a^	Y	Y	Y	Y	Y	Y	Y	Y	Y
V5. Congruity methodology and interpretation of results	Y	Y	Y	Y	Y	Y	U	Y	Y
V6. Statement locating researcher theoretically^a^	N	N	N	N	Y	N	N	Y	N
V7. Influence of researcher on the research (and vice-versa)^a^	N	N	N	N	Y	N	N	N	N
V8. Adequate Representation of the participants	Y	Y	Y	Y	Y	Y	Y	Y	Y
V9. Ethical statement	Y	Y	Y	Y	Y	Y	Y	Y	Y
V10. Do the conclusions flow from the data	Y	Y	Y	Y	Y	Y	Y	Y	Y
Total	8/10	8/10	8/10	8/10	10/10	8/10	7/10	9/10	7/10
Dependability score	Moderate	Moderate	Moderate	Moderate	High	Moderate	Moderate	High	Moderate

^a^: questions regarding dependability score. Y = Yes, N = No, U = Unclear.

**Table 3:** Summary of Findings.

Synthesises finding	Type of research	Dependability	Credibility	ConQual score
Emotional and practical advantages	High	Downgrade 1 level^a^	Downgrade 1 level^b^	Low
Disadvantages of continuous monitoring	High	Downgrade 1 level^a^	Downgrade 1 level^b^	Low
Aspects regarding implementation of continuous monitoring	High	Downgrade 1 level^a^	Downgrade 1 level^b^	Low
False alarms and the importance of clinical assessment	High	Downgrade 1 level^a^	Downgrade 1 level^b^	Low

^a^Downgraded 1 level for each synthesized finding having findings from studies with high and moderate dependability ratings.

^b^Downgraded 1 level for having a mix of unequivocal and credible findings in the synthesized finding.

### Synthesis of the findings

The experiences of nurses described in this review mainly focus on short-term experiences since this intervention is relatively new. Most studies are executed during, or shortly after implementing CMVS on the general ward [22,24–28,30]. One study was aimed to test the feasibility of CMVS without using alarms [25]. Two studies focussed on the implementation process of CMVS [24,27]. Only one study described the experiences of nurses with CMVS after they have been working with this intervention for three years [23].

A total of 124 findings were identified in the individual studies and grouped into 15 categories. These were combined into four synthesized findings: 1) Emotional and practical advantages of CMVS; 2) Disadvantages of CMVS; 3) Aspects regarding implementation and 4) Alarm strategies and clinical assessment. A summary of the synthesized findings and their categories can be found in Table 4 and S3 Text, all extracted data can be found in S4 Data.

**Table 4:** Synthesized Findings and Categories.

Synthesized finding	Included categories
Emotional and practical advantages of working with continuous monitoring of vital signs on general wards	Patients feel safer knowing they are continuously monitored.
	Continuous monitoring provides a feeling of trust for nurses.
	Continuous monitoring helps nurses detect deterioration in an earlier stage.
	Under the right circumstances, continuous monitoring can be time saving for the nurses, especially in shifts with high patient to nurse ratio
Practical disadvantages of working with continuous monitoring of vital signs	Patient related disadvantages, like mobility, interaction between nurse and patient, and distress in patients
	Over-monitoring of patients and causing a data overload of measurements by continuously monitoring to many patients
	Continuous monitoring can be a time consuming intervention
	Limitations of the sensor used in vital signs monitoring, like limited measurements and non-reliable measurements
Important aspects regarding the implementation of continuous monitoring	Continuous monitoring is not a substitute for an intensive care admission
	Aspects regarding the implementation process
	Nurses perceived training and on-the-job coaching as crucial elements for effective utilization of continuous monitoring in their daily practice
	The importance of a properly working technical infrastructure
False alarms and the importance of clinical assessment	Nurses stress the need for their own clinical assessment besides continuous monitoring and relying entirely on alarms
	Nurses describe strategies to reduce false alarms
	Nurses experience feelings of uncertainty and agitation caused by the technical downsides of the specific monitoring systems used for continuous monitoring

1. **Emotional and practical advantages of working with CMVS.**

Several aspects of working with CMVS are experienced as positive by the nurses. They notice patients feel safer and it provides a feeling of trust for the nurses. CMVS helps to detect deterioration in patients in an earlier stage. Under the right circumstances, CMVS can be time saving.

1.1 Patients feel safer knowing they are continuously monitored.

In three studies [23,27,30] nurses described that patients feel safer when they are monitored continuously, knowing nurses can keep an eye on their vital signs from a distance. Nurses also observed improved comfort and satisfaction using CMVS [30].

*There were also patients that felt safe: So you monitor my vitals 24 hours per day. So even if you are not in my room, you monitor me. That gave patients a feeling of safety.* [27]

1.2 Continuous monitoring provides a feeling of trust for the nurses

Nurses perceive CMVS as an extra set of eyes which gives them a feeling of trust [23]. It improves their confidence in managing and prioritizing their caseload, increasing patient safety [28].

*From a distance, you can estimate how your patient is doing, to some extent. Also, if your patient does not feel well, and you really want to be in the room all the time, which is not possible in a nursing ward, you feel you can better monitor your patient. So yes, it provides me a safe feeling.* [23]

1.3 Continuous monitoring helps nurses detect patient deterioration at an earlier stage

Early detection of deterioration is perceived as the most critical advantage of CMVS and is a topic in all of the individual studies [22–28,30]. Earlier detection of deterioration, helps nurses to act sooner and therefore prevent further deterioration of the patient’s condition. CMVS also helps nurses better monitor their interventions’ effect on vital signs.

*Last week, we had a successful resuscitation because CM showed a low heart rate.* [30]

*‘I guess you’re more likely to pick up on things that are changing quite slowly if someone’s blood pressure drops a little bit and you’re doing it more thoroughly and more frequently you’d be able to act on that sooner rather than if you left it four hours and then saw a massive drop and you could intercept and do something about it.’* [28]

1.4 Under the right circumstances, CMVS is perceived as time-saving by the nurses, especially in shifts with high patients to nurse ratio.

When the monitoring devices work properly, and nurses feel confident to work with CMVS devices, they say it can save time because it reduces the workload of current routine manual measuring and registering vital signs [22,24–28,30]. Especially in evening and night shifts where nurses are responsible for a higher number of patients, it can be beneficial and increase efficiency.

*I think because of the frequency of observations and the fact it was recording so frequently it took a little bit of pressure off if you had multiple unwell patients yourself (…)so it’s really reassuring to be able to walk past, have a quick look at other patients and go okay prioritise or not.* [29]

1. **Nurses experience several practical disadvantages of working with continuous monitoring of vital signs.**

Nurses experience four different practical disadvantages of CMVS, 1) patient related disadvantages such as restrictions in mobility and reduced interaction. 2) an overload of measurement data because of continuously monitoring large groups of patients. 3) CMVS can be very time-consuming and 4) limitations to the sensors used for CMVS, such as the usability for specific patients.

2.1 Patients related disadvantages, like mobility, reduced interaction between nurse and patient and distress over changing vital signs.

Some devices used for CMVS have a large battery that patients wore on the wrist [23,30]. This was perceived as impractical and had an negative influence on the daily activities of patients. In another study, the device was not wireless, which means patients were restricted to their bed [28]. This greatly impacted patients’ mobility and was therefore perceived as negative.

*‘They feel like they can’t move with it on. Not that they want to lie there, it just makes it harder for them to mobilise and do things for themselves so, in some in stances it does actually increase our workload … it kind of ties the patient down and it affects other areas of their care’* [28]

Furthermore, nurses mentioned being afraid that CMVS would lead to reduced interactions between nurses and patients, which was very important to them [22,28].

*You need the confidence from the nurses, I would miss that. However, quantity time might become quality time.* [22]

Finally, nurses observed patients who become restless or distressed by seeing their own vital signs, it makes them more aware of their condition [22,28,30].

*Some patients get restless form the data they can see, the beeping of the device, let alone the many cords, adhesives and the fairly heavy device that was attached to the body.* [30]

2.2 The risk of over-monitoring and causing a data overload by continuously monitoring to many patients

Not all patients require CMVS, and nurses acknowledge there should be a clear rationale to measure vital signs continuously [24,26–28]. Patients with a high risk of clinical deterioration have the best benefits of CMVS. Measuring vital signs when the clinical value is not clear can provoke a tired feeling towards CMVS [23]. In addition, CMVS can generate an overload of unnecessary data, which enables nurses to make trend analysis if needed, but this does not always seem useful and this process is still far from optimal [22,23].

*If I only just once had a case where you can actually see deviating trends, then you’ll probably use CM better. My experience is (mainly) with stable patients who have CM that shows the same trends over three consecutive shifts; I think in that case actual use and usefulness fades a bit.* [24]

2.3 Continuous monitoring can be a time-consuming intervention.

In most of the studies in this review, CMVS was implemented as a new intervention on the general ward. Therefore, CMVS can in some situations be more time-consuming and increase workload [22,27,30]. Assigning and connecting patients to the devices and replacing parts are examples of time-consuming elements of CMVS.

*First, we had to open the system, search for the patient in the system. That will already take approximately 5 minutes, so it takes extra time.* [27]

2.4 Limitations of the sensor used in vital signs monitoring.

Most sensors studied could only measure a limited number of vital signs. Nurses experience uncertainty when assessing the patient’s condition based on limited information. The full range of vital signs is needed to measure an early warning score, which provides a more complete insight into the clinical status of the patients, something CMVS alone cannot achieve [22,24,26,27]. In addition, some devices were not suitable for patients with a pacemaker, had to be removed before scans, or when patients wanted to take a shower. Nurses perceived this as a disadvantages of the CMVS system [24,27]. In addition, the device could not be used in patients with cold peripheries and patients who were confused or restless [29].

*Nowadays we work with the EWS. Those are recognizable and guiding in our follow-up actions, like calling a physician when a score is 5. The trends and thresholds did not provide such clear follow-up. Also because CM still does not measure all the vital signs to generate a proper EWS.* [25]

*The patients who are confused and restless or got cold hands or who is just fidgety and they just want to take it off … you find the monitoring on the floor or on the table because they just want it off.* [29]

1. **Nurses suggest multiple important aspects regarding the implementation process which should be considered in case of further upscaling of continuous monitoring on general wards.**

The implementation of CMVS is an important aspect that was mentioned by the nurses in multiple individual studies. Nurses describe the importance of recognizing that CMVS cannot be a substitution for an intensive care admission; they perceive training and coaching on-the-job as crucial elements for a proper implementation and stress the importance of a properly working technical infrastructure.

3.1 Continuous monitoring is not a substitute for an intensive care (ICU) admission

Nurses worry that the ward would become like an ICU because it can lead to reluctance in patient transfers to the ICU, or earlier discharge of patients from the ICU to the general ward [22,23,26]. Nurses described the importance of a clear definition of what CMVS entails, and what the boundaries are for what is possible on a general ward and what is not [22].

*The difference between a high care ward and our general ward is getting smaller using this system. Subsequently, it is difficult to set boundaries, and to frame, between what you should do and not do.* [23]

3.2 Aspects regarding the implementation process itself

Communication is considered an important aspect by nurses when implementing CMVS. A formally appointed implementation leader and key-users were perceived as valuable for the implementation process [25–27].

*The project leader was accessible, and visible on the nursing ward. I think that is important especially at the start, that somebody is always available to answer your questions.* [27]

3.3 Nurses perceived training and on-the-job coaching as crucial elements for effectively utilising CMVS in their daily practice.

In order to successfully work with CMVS nurses consider it essential to receive proper training about the devices and monitoring systems [25–27]. Besides training and easy access to a manual before the start of the intervention, bedside training and coaching on-the-job were perceived as necessary to practice working with the devices and interpret vital sign trends in patients. Repeating the training regularly during the implementation in order to keep their acquired knowledge up to date was also considered important.

*In the beginning I had to get used to it for a while and I still felt insecure about some aspects of CMVS. But it helped that we just started doing it and having an involved project leader and key users. There was always an opportunity to ask questions and she was also often present in the department, so that you just become really confident in working with it.* [25]

3.4 Nurses express the importance of a properly working technical infrastructure

Poorly working Wi-Fi connection or bad technical infrastructure leads to frustration and negative thoughts among nurses working with CMVS [24–27,30]. In multiple studies the data from CMVS was not automatically integrated into the Electronic Medical Record. This was experienced as a downside of the implementation. Automatic integration allows more effective documentation and evaluation of vital signs [24–27].

*Sometimes the separate mobile phone with the specific codes malfunctions and it simple takes too much time, which eventually results in that you leave it at that.* [24]

1. False alarms and the importance of clinical assessment.

The large number of alarms and especially false alarms caused by CMVS are the biggest downside of CMVS as experienced by nurses. This depends on the type of system used for CMVS.

4.1 Nurses stress the need for their own clinical assessment besides CMVS and relying entirely on alarms

Although CMVS can help detect deterioration earlier, nurses still experience the importance of their own clinical assessment. Multiple studies show that trends of CMVS are often a confirmation of their clinical perspective and that it is necessary to take clinical status and context factors into account when assessing the vital signs trend [23–27].

*For example, when the patient is washing and dressing in the morning, you expect a higher breathing and heart rate. In that case this is not clinically relevant and you should not take any action.* [25]

4.2 Nurses experience feelings of uncertainty and agitation caused by the technical downsides of the specific monitoring systems used for CMVS

Different types of systems were used in the studies to cope with alarms of CMVS. In studies where nurses had a mobile device to receive alarms, they experienced feelings of agitation and uncertainty because of the amount of alarms and false alarms that they received [25,26,28]. In one study, they did not have a mobile device and nurses could only see the vital signs on screens in a designated area on the ward, this method also gave the nurses a feeling of uncertainty because they did not get a warning in case of deterioration [23].

*‘I think it is very disconcerting for relatives. Apart from anything else, as the nurses and doctors know they alarm frequently. If they lose the signal, because somebody has moved, or something like that, or the parameters haven’t been set up in a certain way, then it will alarm constantly. And I think that relative perception of those alarms going off is that their relative is, is deteriorating and I think it can be quite panicking for them to hear beep beep beep when their relative is on the monitor.’* [28]

4.3 Nurses describe strategies to reduce false alarms

Nurses suggested that alarm setting could be adjusted, after consultation with a doctor, to reduce the amount of false alarms they receive [24–26,28]. In one study [25], the aim was to assess the feasibility of CMVS without the use of alarms, nurses experienced no added value of alarms when trend analysis was carried out according to the protocol used in this study. Another study [23], showed that when nurses have been working with CMVS for three years, they understand all types and reasons for alarms and can handle the continuous data availability of vital signs efficiently.

*Nine out of ten times you do not have to respond to a false alarm, but you just wait a few seconds before breathing frequency or saturation will improve. I have the idea we are on the right track in recognizing false alarms.* [23]

## Discussion

In this study we aimed to provide an overview of nurses’ experiences working with continuous monitoring of vital signs on the general ward. The findings showed that nurses’ have varying experiences when it comes to CMVS on the general ward. Positive experiences include earlier detection of deterioration and feeling safe. Negative experiences include reduced mobility and data overload. Factors which affect the reported experience include the implementation process and experiences with alarms in relation to their clinical assessment.

The advantages of CMVS experienced by nurses seem to outweigh the practical disadvantages, which justifies investing in further development of CMVS. The early detection of deterioration seems to be the most important positive aspect of CMVS. Besides nurses clinical assessment, CMVS can support them in detecting deterioration and act on deteriorating vital signs earlier to prevent further decline and adverse events. Our findings align with a recent review, showing a significant reduction of mortality and a trend towards a reduced risk of intensive care unit transfers, for which CMVS seems to be the most important factor [31].

Our review showed multiple disadvantages, related to the device used as well as the design of the technical infrastructure. Downey et al.(2022) also found that reduced mobility because of hindering devices and an overload of data caused by continuously monitoring of all patients, especially when patients are considered haemodynamically stable, are experienced as the most important disadvantage. Nurses may feel overwhelmed by the amount of data leading to a lack of confidence when interpreting the trends of this information [32]. These disadvantages are of a practical nature and could potentially be reduced with further development of the devices. Nurse leaders, healthcare administrators and policy makers could, e.g., stimulate and facilitate improvements of the technical infrastructure based on ‘user-centered design’, clear hospital policies on which patients to include in CMVS and invest in the use of AI to aid interpretation of monitoring data.

There are several factors that nurses consider important regarding the implementation of CMVS. Factors such as training and nurses’ doubts about fading boundaries between the nursing ward and the ICU were mentioned in multiple studies [26–28,30]. Considering this, contextual factors seem an important consideration in the implementation process of new interventions [33]. This review showed good communication, implementation leaders and on-the-job coaching are perceived as important aspects by nurses regarding the implementation of CMVS. This aligns with known knowledge concerning implementation of new interventions [33].

The amount of, and especially false alarms caused by the CMVS system lead to frustration and uncertainty among nurses. Alarm fatigue is a common, and well described problem among hospital nursing staff especially in the ICU. Possibly, use of intelligent management interventions to reduce false alarms in the ICU [21] may also be effective in the general ward. Evidence for this effectiveness is limited and more research is needed to further develop alarm management to reduce the amount of false alarms caused by these kinds of interventions [21]. Interestingly, when nurses become more familiar with interpreting the data and alarms, the frustration and agitation seem to diminish [23].

The overall confidence level of the findings in this review is low, which may be due to the inclusion of all study designs. The confidence level is based on the quality of the individual studies, the dependability, and the credibility of the synthesized findings based on the results from qualitative studies [20]. The inclusion of multiple study designs like surveys and mixed method studies may provide less in-depth insight into this topic and could therefore lead to lower confidence levels of the synthesized findings from this review. Future research should gain a more in-depth perspective of the impact CMVS has on nurses’ work and their relationship with the patient, preferably across different hospital settings and countries. Furthermore, more research is needed to describe the long term experiences of CMVS on a general ward which includes stratification for different types of monitoring and devices. When CMVS is more integrated into daily routines and work processes over a longer period of time and nurses are more used to working with CMVS. This can provide more insight into how CMVS has changed their way of working and which solutions for practical disadvantages like alarm fatigue are handled on the ward.

Considering the advantages of CMVS, experienced by nurses and proven in other studies(Sun et al., 2020) investing in CMVS seems to be promising to improve patient safety on general wards. Further development of the sensors and systems supporting CMVS could reduce the practical disadvantages described in this review, and therefore make CMVS a useful tool to besides increasing safety, reduce workload for nurses. Future research could be aimed at the experiences of nurses and patients after CMVS has been integrated into daily practice for a longer period of time, or focus on the implementation process of CMVS on a more widespread basis throughout general hospital wards.

### Strengths and limitations

Strengths of the current review include the concise methodological execution adhering to the JBI guideline for meta-aggregative reviews, as well as the ConQual methodology used for quality assessment, which was performed by two individual researchers. Moreover, the appraisal tool for qualitative studies was used in all of these studies, although four out of eight studies had a mixed methods design. As our primary interest was to extract the qualitative elements of these studies, we purposefully chose this approach to ensure that we only included studies which concisely reported the gathering of the qualitative data. Also, some limitations need to be addressed. First, our search was restricted to English-language articles, which might have excluded relevant literature in other languages. Second, based on the limited number of studies, we could not identify trends or perform subgroup analyses, which may have led to additional insights. Third, the majority of the included studies originated from the Netherlands, which may limit transferability of the results described to other contexts. Fourth, CVMS is a rapidly developing technique, which may result in other experiences when the technique becomes more imbedded into the daily practice. This may warrant revaluation in the future.

## Conclusion

Nurses’ have varying experiences when it comes to CMVS on the general ward. Positive experiences include earlier detection of deterioration and feeling safe, which were considered important positive aspects of CMVS by nurses. Negative experiences include reduced mobility and data overload. Nurses experience multiple important aspects related to the implementation process and experiences with alarms in relation to their own clinical assessment. The advantages of CMVS experienced by nurses seem to outweigh practical disadvantages, which justifies investing in further development of CMVS.

### Relevance to clinical practice

This review provides important insight into the experiences of nurses with continuous monitoring of vital signs on general wards. The findings may facilitate optimal implementation of continuous monitoring of vital signs. Nurses have varying experiences when it comes to CMVS on the general ward. The advantages of CMVS experienced by nurses seem to outweigh practical disadvantages, which justifies investing in further development of CMVS.

**What does this paper contribute to the wider global clinical community?**

- Nurses’ have varying positive and negative experiences when it comes to CMVS on the general ward, e.g., feeling safer, but also additional stress due to (false) alarms.
- The advantages of CMVS experienced by nurses seem to outweigh practical disadvantages, which justifies investing in further development of CMVS.

## Supporting information

S1 Text Search strings for Cinahl, Pubmed and Embase. (DOCX)

S2 Text Study selection. (DOCX)

S3 Text Synthesis of findings. (DOCX)

S4 Data Extracted data from all included studies. (DOCX)

S5 PRISMA Checklist PRISMA 2020 checklist. (DOCX)
