# Supplementary material for: Nurses’ experiences with inhospital continuous monitoring of vital signs in general wards: A systematic review
Source: PLOS Digit Health. 2025 Aug 22;4(8):e0000949. doi: 10.1371/journal.pdig.0000949 (PMC12373230; doi:10.1371/journal.pdig.0000949)
Supplement: S1 Text — (DOCX) [file pdig.0000949.s001.docx]

**Supplemental 1: Search strings for Cinahl, Pubmed and Embase**

**Searchstring CINAHL**

MH "Nurses" OR "Registered Nurse" OR "Nurse" OR "Nursing Personnel " OR "Registered Nurses " OR "RN" OR "Nurs*" OR "Nurses"

AND

MH "Hospitals” OR "Hospitals" OR "Nursing department" OR "Nursing departments" OR "General Hospital"OR "Nursing ward" OR "Ward" OR "General Ward" OR "Surgical Ward" OR "non-surgical ward" OR "Wards"OR "Hospital" OR "General Hospitals" OR "Nursing Unit"

AND

"Hemodynamic monitoring" OR "Continuous monitoring" OR "Monitor*" OR "continuous measuring" OR "Measuring"OR "wearable electronic devices"OR "Wearable Devices" OR "Wearables"  OR "Wireless monitoring"

AND

MH “vital signs” OR "Vital Signs" OR "Vital Sign" OR "Blood pressure"  OR "Pulse rate" OR "Respiratory rate"  OR "Temperature" OR "Body temperature" OR "clinical deterioration" OR "Deterioration"

**Searchstring Pubmed**

((("Nurses"[Mesh] OR "Registered Nurse"[Title/abstract] OR "Nurse" [Title/abstract] OR "Nursing Personnel "[Title/abstract] OR "Registered Nurses "[Title/abstract] OR "RN"[Title/abstract] OR "Nurs*"[Title/abstract] OR "Nurses" [Title/Abstract])

AND

 "Hospitals"[Mesh] OR "Hospitals"[Title/Abstract] OR "Nursing department" [Title/Abstract] OR "Nursing departments" [Title/Abstract] OR "General Hospital" [Title/Abstract] OR "Nursing ward" [Title/Abstract] OR "Ward"[Title/Abstract] OR "General Ward"[Title/Abstract] OR "Surgical Ward"[Title/abstract] OR "non-surgical ward"[Title/Abstract] OR "Wards"[Title/Abstract] OR "Hospital"[Title/Abstract] OR "General Hospitals"[Title/Abstract] OR "Nursing Unit"[Title/Abstract]

AND

 "Hemodynamic monitoring"[Mesh] OR "Hemodynamic monitoring" [Title/Abstract] OR "Continuous monitoring" [Title/Abstract] OR "Monitor*"[Title/Abstract] OR "continuous measuring" [Title/Abstract] OR "Measuring" [Title/Abstract] OR "wearable electronic devices"[Title/abstract] OR "Wearable Devices"[Title/Abstract] OR "Wearables" [Title/Abstract] OR "Wireless monitoring" [Title/Abstract]

AND

 "Vital signs"[Mesh] OR "Body Temperature"[Mesh] OR "Vital Signs" [Title/Abstract] OR "Vital Sign" [Title/Abstract] OR "Blood pressure" [Title/Abstract] OR "Pulse rate" [Title/Abstract] OR "Respiratory rate" [Title/Abstract] OR "Temperature" [Title/Abstract] OR "Body temperature" [Title/Abstract] OR "clinical deterioration"[Title/Abstract] OR "Deterioration" [Title/Abstract]

**Searchstring Embase**

‘Nurse’/exp  OR ‘Registered Nurse’:ti,ab,kw OR ‘Nurse’:ti,ab,kw OR ‘Nursing Personnel’:ti,ab,kw OR ‘Registered Nurses’:ti,ab,kw OR ‘RN’:ti,ab,kw OR ‘Nurs*’:ti,ab,kw OR ‘Nurses’:ti,ab,kw

 AND

'hospital'/exp OR ‘Hospitals’:ti,ab,kw OR ‘Nursing department’:ti,ab,kw OR ‘Nursing departments’:ti,ab,kw OR ‘General Hospital’:ti,ab,kw OR ‘Nursing ward’:ti,ab,kw OR ‘Ward’:ti,ab,kw OR ‘General Ward’:ti,ab,kw OR ‘Surgical Ward’:ti,ab,kw OR ‘non-surgical ward’:ti,ab,kw OR ‘Wards’:ti,ab,kw OR ‘Hospital’:ti,ab,kw OR ‘General Hospitals’:ti,ab,kw OR ‘Nursing Unit’:ti,ab,kw

  AND

 'hemodynamic monitoring'/exp  OR ‘Hemodynamic monitoring’:ti,ab,kw OR ‘Continuous monitoring’:ti,ab,kw OR ‘Monitor*’:ti,ab,kw OR ‘continuous measuring’:ti,ab,kw OR ‘Measuring’:ti,ab,kw OR ‘wearable electronic devices’:ti,ab,kw OR ‘Wearable Devices’:ti,ab,kw OR ‘Wearables’:ti,ab,kw OR ‘Wireless monitoring’:ti,ab,kw

 AND

 'vital sign'/exp  OR 'body temperature'/exp OR ‘Vital Signs’:ti,ab,kw OR ‘Vital Sign’:ti,ab,kw OR ‘Blood pressure’:ti,ab,kw OR ‘Pulse rate’:ti,ab,kw OR ‘Respiratory rate’:ti,ab,kw OR ‘Temperature’:ti,ab,kw OR ‘Body temperature’:ti,ab,kw OR ‘clinical deterioration’:ti,ab,kw OR ‘Deterioration’:ti,ab,kw
