# Supplementary material for: Nurses’ experiences with inhospital continuous monitoring of vital signs in general wards: A systematic review
Source: PLOS Digit Health. 2025 Aug 22;4(8):e0000949. doi: 10.1371/journal.pdig.0000949 (PMC12373230; doi:10.1371/journal.pdig.0000949)
Supplement: S2 Text — (DOCX) [file pdig.0000949.s002.docx]

**Supplemental file 2: All excluded studies with reasons**

| 1 | Wrong outcome |  | 2006 | Dijkstra, R. |
| --- | --- | --- | --- | --- |
| 2 | Wrong outcome | A 4-year implementation strategy of aggressive post-resuscitation care and temperature management after cardiac arrest | 2014 | Pellis, T. and Sanfilippo, F. and Roncarati, A. and Dibenedetto, F. and Franceschino, E. and Lovisa, D. and Magagnin, L. and Mercante, W. P. and Mione, V. |
| 3 | Wrong outcome | 22 ASSESSMENT OF FLUID MANAGEMENT AND IT'S RELATION TO OUTCOMES FOLLOWING FRACTURE NECK OF FEMUR | 2014 | Mercer, M. and Rippingale, C. and Lines, E. |
| 4 | Wrong outcome | 31 POSTURAL HYPOTENSION MEASUREMENT â€“ A PROSPECTIVE OBSERVATIONAL STUDY IN A DISTRICT GENERAL HOSPITAL | 2014 | Barsaiyan, G. and Mildner, R. |
| 5 | Background Article | 36th International Symposium on Intensive Care and Emergency Medicine : Brussels, Belgium. 15-18 March 2016 | 2016 | Bateman, R. M. and Sharpe, M. D. and Jagger, J. E. and Ellis, C. G. et al |
| 6 | Wrong outcome | 110 THE ACUTELY UNWELL PATIENT AND SINGLE ROOMS | 2014 | Preston, J. C. and Sahota, H. K. and Maskell, P. M. |
| 7 | Wrong outcome | 0310Napping during night shift and self-reported hypertension among nursing workers | 2014 | Rotenberg, Lucia and Silva-Costa, Aline and Roberto Vasconcellos-Silva, Paulo and Harter Griep, Rosane |
| 8 | Wrong outcome | 817 AVOIDING THAT SINKING FEELING: A QIP TO IMPROVE THE IDENTIFICATION OF POSTURAL HYPOTENSION ON A MEDICINE FOR THE ELDERLY WARD...British Geriatrics Society Abstracts from the Autumn Meeting (Virtual), November 24-26, 2021 | 2022 | Gilmartin, C. G. S. and Peacock, M. and Coultas, J. and Alavi, N. and Long, S. |
| 9 | Wrong outcome | An Academic-Practice Partnership to Advance Million HeartsÂ® | 2021 | Cooper, Jennifer |
| 10 | Wrong outcome | Acceptability and usability of a nurse-assisted remote patient monitoring intervention for the post-hospital follow-up of patients with long-term illness: A qualitative study | 2024 | Wathne, H. and May, C. and Morken, I. M. and Storm, M. and HusebÃ¸, A. M. L. |
| 11 | Wrong population | Acceptability of a parental early warning tool for parents of infants with complex congenital heart disease: a qualitative feasibility study | 2018 | Gaskin, K. L. and Wray, J. and Barron, D. J. |
| 12 | Wrong population | Acceptance and Tolerability of Helmet CPAP in Pediatric Bronchiolitis and Pneumonia: A Feasibility Study | 2023 | Smith, M. E. and Gray, M. and Wilson, P. T. |
| 13 | Wrong Population | Acceptance and User Experiences of a Wearable Device for the Management of Hospitalized Patients in COVID-19-Designated Wards in Ho Chi Minh City, Vietnam: Action Learning Project | 2024 | Luu, A. P. and Nguyen, T. T. and Cao, V. T. C. and Ha, T. H. D. and Chung, L. T. T. and Truong, T. N. and Nguyen Le Nhu, T. and Dao, K. B. and Nguyen, H. V. and Khanh, P. N. Q. and Le, K. T. T. and Tran, L. H. B. and Nhat, P. T. H. and Tran, D. M. and Lam, Y. M. and Thwaites, C. L. and McKnight, J. and Vinh Chau, N. V. and Van Nuil, J. I. |
| 14 | Wrong outcome | Accuracy and applicability of the Terumo ES-H55 double-cuff sphygmomanometer for hospital use | 2003 | Tochikubo, O. and Nishijima, K. and Ohshige, K. and Kimura, K. |
| 15 | Wrong outcome | [Accuracy and precision in blood pressure measurement. Comparative study of home self-measurement with measurement in the clinic and out-patient monitoring] | 2001 | DivisÃ³n, J. and Puras, A. and Sanchis, C. and Artigao, L. and LÃ³pez Abril, J. and LÃ³pez De Coca, E. and MassÃ³, J. and RodrÃ­guez PaÃ±os, B. |
| 16 | Wrong population | Accuracy of different temperature devices in the postpartum population | 2009 | Hutton, S. and Probst, E. and Kenyon, C. and Morse, D. and Friedman, B. and Arnold, K. and Helsley, L. |
| 17 | Wrong outcome | Accuracy of intra-arterial lineÂ transducer levelling practice in a general intensive care unit | 2024 | Jacobs, K. and Jarrett, P. and Ballard, E. and Fox, A. |
| 18 | Wrong population | Accuracy of pacifier thermometers in young children | 2006 | Braun, C. A. |
| 19 | Wrong population | Accuracy of parents in measuring body temperature with a tympanic thermometer | 2005 | Robinson, J. L. and Jou, H. and Spady, D. W. |
| 20 | Wrong outcome | The accuracy of undergraduate paramedic students in measuring blood pressure: A pilot study | 2014 | Boyle, M. and Williams, B. and Sawyer, S. |
| 21 | Wrong population | Accuracy, precision, and validity of fever detection using non-invasive temperature measurement in adult coronary care unit patients with pulmonary catheters | 2012 | Joo, G. and Sohng, K. Y. |
| 22 | Wrong outcome | Activity Monitoring and Heart Rate Variability as Indicators of Fall Risk: Proof-of-Concept for Application of Wearable Sensors in the Acute Care Setting | 2017 | Razjouyan, J. and Grewal, G. S. and Rishel, C. and Parthasarathy, S. and Mohler, J. and Najafi, B. |
| 23 | Wrong outcome | The acute effects of Red Ginseng on working stress of nursing stuff at the care hospital for the aged (ROUJIN BYOUIN) | 1998 | Kaneko, H. and Nakanishi, K. and Murakami, A. and Kuwashima, K. and Ikeda, K. and Samukawa, K. |
| 24 | Wrong population | Acute fatty liver of pregnancy causing multiple organ dysfunction syndrome in a Chinese intensive care unit | 2023 | Shen, Y. and Wang, X. and Yao, Y. and Zhou, X. |
| 25 | Wrong outcome | Acute kidney injury and continuous renal replacement therapy: A nursing perspective for my shift today in the intensive care unit | 2021 | Baldwin, I. and Mottes, T. |
| 26 | Wrong outcome | Acute pain services in the United Kingdom | 2004 | Nagi, H. |
| 27 | Wrong outcome | Acute stroke nursing: Standards and practical applications Turkish cerebrovascular disease society and the society of neurological nursing joint strategy project | 2020 | TopÃ§uoÄŸlu, M. A. and TÃ¼lek, Z. and Boyraz, S. and Ã–zdemir, A. Ã– and Ã–zakgÃ¼l, A. and GÃ¼ler, A. and Nazliel, B. and Togay IÅŸikay, C. and Yaka, E. and Arsava, E. M. and Ã‡aÄŸlar, G. and Åžirin, H. and Midi, I. and Atmaca, M. M. and Alankaya, N. and Ongun, N. and Yildirim, N. and AykaÃ§, Ã– and KÃ¼Ã§Ã¼kgÃ¼Ã§lÃ¼, Ã– and Usta YeÅŸilbalkan, Ã– and Baydemir, R. and Ã–ztÃ¼rk, Åž and Acar, T. and MollaoÄŸlu, M. and Karadakovan, A. and Durna, Z. |
| 28 | Wrong outcome | Adaptive threshold-based alarm strategies for continuous vital signs monitoring | 2022 | van Rossum, M. C. and Vlaskamp, L. B. and Posthuma, L. M. and Visscher, M. J. and Breteler, M. J. M. and Hermens, H. J. and Kalkman, C. J. and Preckel, B. |
| 29 | Wrong outcome | Adding story-centered care to standard lifestyle intervention for people with Stage 1 hypertension | 2006 | Liehr, P. and Meininger, J. C. and Vogler, R. and Chan, W. and Frazier, L. and Smalling, S. and Fuentes, F. |
| 30 | Wrong outcome | Addition of topical airway anaesthesia to conventional induction techniques to reduce haemodynamic instability during the induction period in patients undergoing cardiac surgery: Protocol for a randomised controlled study | 2022 | Chen, T. T. and Lv, M. and Wang, J. H. and Wei, C. S. and Gu, C. P. and Wang, Y. L. |
| 31 | Wrong outcome | Addressing Hypertension Care in Africa (ADHINCRA): Study protocol for a cluster-randomized controlled pilot trial | 2023 | Commodore-Mensah, Y. and Sarfo, F. S. and Turkson-Ocran, R. A. and Foti, K. and Mobula, L. M. and Himmelfarb, C. D. and Carson, K. A. and Appiah, L. T. and Degani, M. and Lang'at, C. and Nyamekye, G. and Molello, N. E. and Ahima, R. and Cooper, L. A. |
| 32 | Wrong outcome | Adequacy of physician clinical rounds and nursing care elements for non-COVID-19 infected patients admitted during the COVID-19 pandemic | 2022 | Alrasheed, A. and Shamou, J. and Rajendram, R. and Boqaeid, A. and Qasim, S. and Baharoon, W. and Layqah, L. and Baharoon, S. |
| 33 | Wrong population | Adherence to the bedside paediatric early warning system (BedsidePEWS) in a pediatric tertiary care hospital | 2021 | Gawronski, O. and Ferro, F. and Cecchetti, C. and Ciofi Degli Atti, M. and Dall'Oglio, I. and Tiozzo, E. and Raponi, M. |
| 34 | Wrong outcome | Administration of high-dose continuous infusion interleukin-2 to patients age 70 or over | 2005 | Quan Jr, W. and Ramirez, M. and Taylor, C. and Quan, F. and Vinogradov, M. and Walker, P. |
| 35 | Wrong outcome | Administrative Experiences for Safety of Mild COVID-19 Patients in Community Treatment Centers in South Korea | 2022 | Kim, H. B. and Han, S. and Kim, G. W. |
| 36 | Wrong population | Admission and discharge guidelines for the pediatric patient requiring intermediate care | 2004 | Jaimovich, David G. |
| 37 | Wrong population | Admissions to a Low-Resource Neonatal Unit in Malawi Using a Mobile App and Dashboard: A 1-Year Digital Perinatal Outcome Audit | 2021 | Mgusha, Y. and Nkhoma, D. B. and Chiume, M. and Gundo, B. and Gundo, R. and Shair, F. and Hull-Bailey, T. and Lakhanpaul, M. and Lorencatto, F. and Heys, M. and Crehan, C. |
| 38 | Wrong outcome | Advanced practice nurse intervention versus usual care for hypertension control: study protocol for an open-label randomized controlled trial | 2023 | Vay-Demouy, J. and Cinaud, A. and Malka, N. and Mion, B. and Kretz, S. and Lelong, H. and Blacher, J. |
| 39 | Wrong outcome | Advanced technology leads to earlier intervention for clinical deterioration on medical/surgical units | 2019 | Mau, Kathleen A. and Fink, Suzanne and Hicks, Brent and Brookhouse, Andrew and Flannery, Ann Marie and Siedlecki, Sandra L. |
| 40 | Wrong outcome | ADVERSE EVENTS DURING INTRA-HOSPITAL TRANSPORTATION IN INTENSIVE CARE UNIT | 2016 | da Silva, Renata and Nazareth Amante, Lucia and Chiodelli Salum, Nadia and Martins, Tatiana and Werner, Joane |
| 41 | Wrong outcome | Adverse reactions associated with mobile therapeutic apheresis: analysis of 17,940 procedures | 2001 | Kiprov, D. D. and Golden, P. and Rohe, R. and Smith, S. and Hofmann, J. and Hunnicutt, J. |
| 42 | Wrong outcome | Agency calls for incident reviews after unexpected cardiac arrests | 2010 | Waters, A. |
| 43 | Wrong outcome | Aggregate National Early Warning Score (NEWS) values are more important than high scores for a single vital signs parameter for discriminating the risk of adverse outcomes | 2015 | Jarvis, S. and Kovacs, C. and Briggs, J. and Meredith, P. and Schmidt, P. E. and Featherstone, P. I. and Prytherch, D. R. and Smith, G. B. |
| 44 | Wrong outcome | Alarm fatigue and its influence on staff performance | 2015 | Deb, S. and Claudio, D. |
| 45 | Wrong outcome | [Alarm fatigue during continuous monitoring at the general ward] | 2022 | Leenen, J. P. L. and Kalkman, C. J. and Patijn, G. A. |
| 46 | Wrong outcome | Alarm limit settings for early warning systems to identify at-risk patients | 2009 | Burgess, L. P. A. and Herdman, T. H. and Berg, B. W. and Feaster, W. W. and Hebsur, S. |
| 47 | Wrong outcome | Alarm Management in Intensive Care: Qualitative Triangulation Study | 2024 | Mosch, L. and SÃ¼mer, M. and Flint, A. R. and Feufel, M. and Balzer, F. and MÃ¶rike, F. and Poncette, A. S. |
| 48 | Wrong outcome | Alarm of monitoring invasive of blood pressure: are we giving the attention required? | 2015 | Kuckartz Pergher, Adele and Lyra da Silva, Roberto Carlos |
| 49 | Wrong outcome | Alarms in a neurocritical care unit: a prospective study | 2022 | Unal, A. and Arsava, E. M. and Caglar, G. and Topcuoglu, M. A. |
| 50 | Wrong outcome | Altered diurnal variation of blood pressure in elderly subjects with decreased activity of daily living and impaired cognitive function | 2001 | Ohya, Y. and Ohtsubo, T. and Tsuchihashi, T. and Eto, K. and Sadanaga, T. and Nagao, T. and Abe, I. and Fujishima, M. |
| 51 | Wrong outcome | An alternative facility for a stroke unit in a community hospital | 2011 | Bergman, M. and Ori, Y. and Blumberger, N. and Salman, H. |
| 52 | Wrong outcome | The AMBITIOUS Study Design and Rationale: Ambulatory Blood Pressure in Taiwanese Occupational Healthcare Staff | 2014 | Yang, L. T. and Chen, P. W. and Lin, T. H. and Chiang, K. H. and Shih, C. M. and Hsieh, M. C. and Tseng, W. K. and Yeh, H. I. and Liu, P. Y. |
| 53 | Wrong outcome | Ambivalence in nurses' use of the early warning score: AÂ focussed ethnography in a hospital setting | 2022 | MÃ¸lgaard, R. R. and JÃ¸rgensen, L. and Christensen, E. F. and GrÃ¸nkjaer, M. and Voldbjerg, S. L. |
| 54 | Wrong outcome | Ambulatory blood pressure measurement as a predictor of outcome in an Irish population: Methodology for ascertaining mortality outcome | 2003 | Dolan, E. and Atkins, N. and McClory, S. and Hinedi, K. and Sharif, S. and McCormack, P. and Staessen, J. and Thijs, L. and Stanton, A. and O'Brien, E. |
| 55 | Wrong outcome | Ambulatory blood pressure monitoring: A nurse practitioner run program | 2020 | Burke, C. and Haut, C. and Hussong, K. and Zaritsky, J. |
| 56 | Wrong outcome | Ambulatory blood pressure: Normality and comparison with other measurements | 1999 | Schettini, C. and Bianchi, M. and Nieto, F. and Sandoya, E. and Senra, H. |
| 57 | Wrong outcome | Ambulatory Intensive, Multidisciplinary Telehealth for High-Risk Discharges: Program Development, Implementation, and Early Impact | 2023 | Hilgeman, B. C. and Lamb, G. |
| 58 | Wrong outcome | American Society for Pain Management Nursing Guidelines on Monitoring for Opioid-Induced Sedation and Respiratory Depression | 2011 | Jarzyna, Donna and Jungquist, Carla R. and Pasero, Chris and Willens, Joyce S. and Nisbet, Allison and Oakes, Linda and Dempsey, Susan J. and Santangelo, Diane and Polomano, Rosemary C. |
| 59 | Wrong outcome | Anaemia management on a haemodialysis unit | 2012 | Gerrish, Martin |
| 60 | Wrong outcome | Analysing Pre-Operative Gait Patterns Using Inertial Wearable Sensors: An Observational Study of Participants Undergoing Total Hip and Knee Replacement | 2024 | Natarajan, P. and Yin, A. L. C. and Fonseka, R. D. and Abi-Hanna, D. and Rooke, K. and Sy, L. and Maharaj, M. and Broe, D. and Koinis, L. and Mobbs, R. J. |
| 61 | Wrong population | Analysis of 4 sedation rating scales in the critical patient | 2009 | Frade Mera, M. J. and Guirao Moya, A. and Esteban SÃ¡nchez, M. E. and Rivera Alvarez, J. and Cruz Ramos, A. M. and Bretones Chorro, B. and ViÃ±as SÃ¡nchez, S. and Jacue Izquierdo, S. and Montane LÃ³pez, M. |
| 62 | Wrong outcome | Analysis of ROX Index, ROX-HR Index, and SpO2/FIO2 Ratio in Patients Who Received High-Flow Nasal Cannula Oxygen Therapy in Pediatric Intensive Care Unit | 2023 | Choi, S. H. and Kim, D. Y. and Song, B. Y. and Yoo, Y. S. |
| 63 | Wrong outcome | Analysis of Symptomatic Venous Thromboembolism Occurrence in Elderly Patients Following Total Hip Arthroplasty and the Impact of Formal Nursing Intervention | 2024 | Shou, J. and Li, Y. and Dong, C. |
| 64 | Wrong outcome | An Analysis of Tasks of Nurses Caring for Patients with COVID-19 in a Nationally-Designated Inpatient Treatment Unit | 2022 | Jung, Minho and Kim, Moon-Sook and Lee, Joo-Yeon and Lee Kyung, Yi and Park, Yeon-Hwan |
| 65 | Wrong outcome | An analysis of the decisions in the management of premature rupture of the membranes | 1986 | Nagey, D. A. and Saller, D. N., Jr. |
| 66 | Wrong outcome | Analysis of the effect of comprehensive physical and mental nursing for patients with acute cerebral infarction in intravenous thrombolytic therapy | 2024 | Zhang, Y. and Wang, M. and Zhao, T. and Zhang, J. and Shen, Z. |
| 67 | Wrong population | Analysis of the Effect of Incentive Nursing Intervention in Children with Severe Viral Encephalitis and Myocarditis during Rehabilitation Based on Diffusion Weighted MRI | 2021 | Ren, Q. and Guo, L. and Liu, X. and Xiao, P. and Tang, S. and Sharma, A. and Walia, T. S. and Shah, M. A. |
| 68 | Wrong outcome | Analysis of the registry of the initial care given to the patient with severe trauma | 2011 | LÃ³pez, Candelas L. and SÃ¡nchez, Cindia M. and Vela, Silvia T. and PÃ©rez, MÂª del A. M. and SÃ¡nchez, Iluminada P. and Gaspar, Raquel V. and LÃ³pez, Olga M. and GÃ³mez, Gemma S. and Solanas, Manuela C. and LÃ³pez, Emilio A. |
| 69 | Wrong population | Analysis of visual attention and team communications during neonatal endotracheal intubations using eye-tracking: An observational study | 2020 | Law, B. H. Y. and SchmÃ¶lzer, G. M. |
| 70 | Wrong outcome | Anesthesia capacity in Ghana: A teaching hospital's resources, and the national workforce and education | 2017 | Brouillette, M. A. and Aidoo, A. J. and Hondras, M. A. and Boateng, N. A. and Antwi-Kusi, A. and Addison, W. and Hermanson, A. R. |
| 71 | Wrong outcome | Anesthesia practice and clinical trends in interventional radiology: A European survey | 2000 | Haslam, P. J. and Yap, B. and Mueller, P. R. and Lee, M. J. |
| 72 | Wrong outcome | Anesthesia Practice and Perioperative Outcomes at Two Tertiary Care Hospitals in Freetown, Sierra Leone | 2016 | Koka, R. and Chima, A. M. and Sampson, J. B. and Jackson, E. V. and Ogbuagu, O. O. and Rosen, M. A. and Koroma, M. and Tran, T. P. and Marx, M. K. and Lee, B. H. |
| 73 | Wrong outcome | An annotated ventricular tachycardia (VT) alarm database: Toward a uniform standard for optimizing automated VT identification in hospitalized patients | 2023 | Pelter, M. M. and Carey, M. G. and Al-Zaiti, S. and Zegre-Hemsey, J. and Sommargren, C. and Isola, L. and Prasad, P. and Mortara, D. and Badilini, F. |
| 74 | Wrong outcome | Antibiotics administered as continuous intravenous infusion over 24â€‰hours by elastomeric devices to patients treated at home: a study of infusion efficiency | 2024 | Docherty, Toni and David, Michael and Schneider, Jennifer and O'Kane, Gabrielle and Morris, Joni and Paavola, Catherine and Sawers, Janelle and O'Mahony, Deirdre and Cooper, Joyce |
| 75 | Wrong outcome | Antihypertensive Medication and Fracture Risk in Older Veterans Health Administration Nursing Home Residents | 2024 | Dave, C. V. and Li, Y. and Steinman, M. A. and Lee, S. J. and Liu, X. and Jing, B. and Graham, L. A. and Marcum, Z. A. and Fung, K. Z. and Odden, M. C. |
| 76 | Wrong outcome | Anxiety and labile hypertension in a 16-year-old male: The value of biopsychosocial medicine | 2003 | Stein, M. T. and Dickstein, D. P. and Pine, D. S. and Stoughton, P. |
| 77 | Wrong outcome | Application Effect of Cluster-Based Care in Patients with Hypertensive Disorders of Pregnancy and Osteoarthritis | 2022 | Ye, L. and Yu, C. and Chen, X. and Han, Y. |
| 78 | Wrong outcome | Application Effect of Doctor-Nurse-Patient Integration Model Based on Heart Rate Management Strategies in Middle-Aged and Young Outpatients with Hypertension | 2022 | Zhang, J. and Han, Z. and Jia, M. and Guo, J. and Guo, H. and Deng, H. |
| 79 | Wrong outcome | Application Effect of Time Nursing Theory Based Clinical Nursing Pathway on Gestational Hypertension Patients | 2021 | Zhou, Y. and Ji, Z. and Du, R. and Rao, G. M. and Zhao, Y. N. |
| 80 | Wrong outcome | Application of bundle management strategy in early mobility of mechanically ventilated patients | 2021 | Ying, W. and Zhaoqing, S. and Xiaoying, R. and Runling, G. |
| 81 | Wrong outcome | [Application of bundle management strategy in early mobility of mechanically ventilated patients] | 2021 | Wang, Y. and Sun, Z. and Ren, X. and Guo, R. |
| 82 | Wrong outcome | Application of End-Tidal CO2Monitoring to ICU Management | 2024 | Owens, B. and Hall, C. |
| 83 | Wrong outcome | Application of infrared thermography in the early warning of pressure injury: A prospective observational study | 2021 | Cai, Fuman and Jiang, Xiaoqiong and Hou, Xiangqing and Wang, Duolao and Wang, Yu and Deng, Haisong and Guo, Hailei and Wang, Haishuang and Li, Xiaomei |
| 84 | Wrong outcome | Application of intelligent nursing based on cloud computing of internet of things in children with pneumonia and sepsis treated with human gamma globulin | 2023 | Qin, A. and Liu, Y. and Shao, C. and Dong, H. |
| 85 | Wrong outcome | Application of Medical Information Systems for the detection of high risk patients: rapid care alerts. Pilot study of the ARA-Son LlÃ tzer Project | 2013 | SocÃ­as CrespÃ­, L. and Heras La Calle, G. and Estrada RodrÃ­guez, V. M. and GarcÃ­a SÃ¡nchez, A. and IbÃ¡Ã±ez-LucÃ­a, P. |
| 86 | Wrong outcome | Application of non-contact sensors for health monitoring in hospitals: a narrative review | 2024 | Choo, Y. J. and Lee, G. W. and Moon, J. S. and Chang, M. C. |
| 87 | Wrong outcome | Application of PDCA Process Management in Day Operation Ward and the Influence of Nursing Quality and Safety | 2022 | Ma, H. and Cao, J. and Li, M. |
| 88 | Wrong outcome | APPLICATION OF REFINED MANAGEMENT UNDER THE GUIDANCE OF TEMPERATURE AGITATION MONITORING IN RECOVERY PERIOD MANAGEMENT OF COLORECTAL CANCER PATIENTS UNDERGOING LAPAROSCOPIC SURGERY UNDER GENERAL ANESTHESIA | 2023 | Cao, M. and Feng, R. |
| 89 | Wrong outcome | Application of the Consolidated Framework for Implementation Research to examine nurses' perception of the task shifting strategy for hypertension control trial in Ghana | 2020 | Gyamfi, J. and Allegrante, J. P. and Iwelunmor, J. and Williams, O. and Plange-Rhule, J. and Blackstone, S. and Ntim, M. and Apusiga, K. and Peprah, E. and Ogedegbe, G. |
| 90 | Wrong outcome | Application of the national early warning score (NEWS) in patients with acute aortic dissection: A caseâ€“control study | 2022 | Liu, Yuwen and Li, Qingyin and Zhang, Yanjuan and Zhao, Rui and Pang, Ran and Ren, Hua |
| 91 | Wrong outcome | APPLIED RESEARCH. Pre-existing variables and outcome of cardiac arrest resuscitation in hospitalized patients | 1998 | Chaplik, S. and Neafsey, P. J. |
| 92 | Wrong outcome | Appropriate use of hospital monitoring capabilities | 1989 | Curry, K. and Scott, L. and Kearney, R. and Rosemurgy, A. S. |
| 93 | Wrong outcome | Are BP readings taken after a patient-physician encounter in a real-world clinic scenario the lowest of all the readings in a clinic visit | 2015 | Shahab, H. and Khan, H. S. and Almas, A. and Khan, S. A. and Khan, A. H. |
| 94 | Wrong outcome | Are current wireless monitoring systems capable of detecting adverse events in high-risk surgical patients? A descriptive study | 2020 | Breteler, M. J. M. and KleinJan, E. and Numan, L. and Ruurda, J. P. and Van Hillegersberg, R. and Leenen, L. P. H. and Hermans, M. and Kalkman, C. J. and Blokhuis, T. J. |
| 95 | Wrong outcome | Are our nurses healthy? Cardiorespiratory fitness in a very exhausting profession | 2020 | SovovÃ¡, M. and SovovÃ¡, E. and NaklÃ¡dalovÃ¡, M. and PokornÃ¡, T. and Å tÃ©gnerovÃ¡, L. and MasnÃ½, O. and MoravcovÃ¡, K. and Å tÄ›pÃ¡nek, L. |
| 96 | Wrong outcome | Are We Ready for Video Recognition and Computer Vision in the Intensive Care Unit? A Survey | 2021 | Glancova, A. and Do, Q. T. and Sanghavi, D. K. and Franco, P. M. and Gopal, N. and Lehman, L. M. and Dong, Y. and Pickering, B. W. and Herasevich, V. |
| 97 | Wrong outcome | Arterial blood oxygen saturation and sedation level of the patients hospitalized in ICUs | 2016 | Keykha, A. A. and Arbabshastan, M. E. and Askari, H. and Abbaszadeh, A. and Hosseini, B. M. K. |
| 98 | Wrong outcome | [Arterial hypertension difficult to control in the elderly patient. The significance of the "white coat effect"] | 1999 | Amado, P. and Vasconcelos, N. and Santos, I. and Almeida, L. and NazarÃ©, J. and Carmona, J. |
| 99 | Wrong outcome | Arterial hypertension in the adolescent. Conventional clinical measurement (CCM) versus continuous monitoring of ambulatory blood pressure data (CMABP) | 1994 | Jabary, N. S. and Bretana, M. and Ardura, J. and Sanchez, G. and Dapena, F. and Bustamante, J. |
| 100 | Wrong outcome | Arterial hypertension: nursing workers' health profile of a university hospital | 2004 | Reiners, A. A. O. and da Costa, A. L. R. and de Arruda, A. L. G. and da Costa, L. M. F. and Nogueira, M. S. |
| 101 | Wrong outcome | Arterial pre- and hypertension prevalence in nursing personnel | 2007 | Mata, E. R. F. and PÃ©rez, C. M. |
| 102 | Wrong outcome | Assess Before Rx: Reducing the Overtreatment of Asymptomatic Blood Pressure Elevation in the Inpatient Setting | 2019 | Pasik, Sara D. and Sophia, Chiu and Jeong, Yang and Sinfield, Catherine and Zubizarreta, Nicole and Ramkeesoon, Rosemarie and Cho, Hyung J. and Krouss, Mona and Chiu, Sophia and Yang, Jeong |
| 103 | Wrong outcome | Assessing blood administering practices | 1999 | Shulman, I. A. and Saxena, S. and Ramer, L. |
| 104 | Wrong outcome | Assessing effects of diet alteration on selected parameters of chronically mentally ill residents of a 24-hour Nursing Home. Part 3: Effects of diet modification on selected health indicators | 2023 | Friedrich, M. E. and Fugiel, J. and Dziaduch, I. |
| 105 | Wrong outcome | Assessing health literacy in safety net primary care practices | 2016 | McCune, R. L. and Lee, H. and Pohl, J. M. |
| 106 | Wrong outcome | Assessing Structural Quality Elements of Pediatric Emergency Care | 2016 | Schroeder, Lisa L. and Alpern, Elizabeth R. and Blecher, Shuntel M. and Peska, Patty A. and White, Marjorie L. and Shaw, Julie A. and Hronek, Carla and Thurm, Cary W. and Alessandrini, Evaline A. |
| 107 | Wrong outcome | Assessing the performance of nurses in the proper adjustment of monitoring instruments in the neonatal intensive care unit | 2019 | Farhat, A. S. and Mohammadzadeh, A. and Saeidi, R. and Rahimi, F. C. and Forough, A. R. and Izanloo, A. |
| 108 | Wrong outcome | Assessing Workplace Stress Among Nurses Using Heart Rate Variability Analysis With Wearable ECG Device-A Pilot Study | 2021 | Li, X. and Zhu, W. and Sui, X. and Zhang, A. and Chi, L. and Lv, L. |
| 109 | Wrong outcome | Assessing, monitoring and managing continuous intravenous sedation for critically ill adult patients and implications for emergency nursing practice: A systematic literature review | 2015 | Varndell, Wayne and Elliott, Doug and Fry, Margaret |
| 110 | Wrong outcome | Assessment and monitoring of nutritional status in patients with advanced cancer: part 1 | 2007 | Dewey, A. and Dean, T. |
| 111 | Wrong population | [Assessment by a national survey of needs for NICU and intermediate NICU in France] | 2003 | Gouyon-Cornet, B. and BrÃ©art, G. and Chabernaud, J. L. and Dehan, M. and Foucaud, P. and Gigonnet, J. M. and Gouyon, J. B. and Lejeune, C. and Lequien, P. |
| 112 | Wrong outcome | Assessment of a telerehabilitation and a telehomecare program for veterans with chronic illnesses | 2006 | Bendixen, R. M. |
| 113 | Wrong outcome | Assessment of blood pressure in patients with Type 2 diabetes: Comparison between home blood pressure monitoring, clinic blood pressure measurement and 24-h ambulatory blood pressure monitoring | 2001 | Masding, M. and Jones, J. R. and Bartley, E. and Sandeman, D. D. |
| 114 | Wrong population | Assessment of sedation levels in pediatric intensive care patients can be improved by using the COMFORT "behavior" scale | 2005 | Ista, E. and Van Dijk, M. and Tibboel, D. and De Hoog, M. |
| 115 | Wrong outcome | Assessment of the techniques of blood pressure measurement by health professionals | 2003 | Veiga, E. V. and Nogueira, M. S. and CÃ¡rnio, E. C. and Marques, S. and Lavrador, M. A. and de Moraes, S. A. and Souza, L. A. and Lima, N. K. and Nobre, F. |
| 116 | Wrong outcome | Assessment, Treatment, and Follow-Up of Phlebitis Related to Peripheral Venous Catheterisation: A Delphi Study in Spain | 2024 | TornÃ©-Ruiz, A. and Reguant, M. and SanromÃ -Ortiz, M. and Piriz, M. and Roca, J. and GarcÃ­a-ExpÃ³sito, J. |
| 117 | Wrong outcome | Assessments of perioperative respiratory pattern with non-contact vital sign monitor in children undergoing minor surgery: a prospective observational study | 2023 | Hateruma, Y. and Nozaki-Taguchi, N. and Son, K. and Tarao, K. and Kawakami, S. and Sato, Y. and Isono, S. |
| 118 | Foreign Language | AssistÃªncia de enfermagem perioperatÃ³ria aos pacientes com cÃ¢ncer de bexiga | 2016 | Megumi Sonobe, Helena and Sabino Ravena, Rouziane and Saia Moreno, FÃ¡bio and Scatralhe Buetto, Luciana and Silva de Oliveira, Marissa |
| 119 | Wrong outcome | The association between nurse staffing levels and the timeliness of vital signs monitoring: a retrospective observational study in the UK | 2019 | Redfern, O. C. and Griffiths, P. and Maruotti, A. and Recio Saucedo, A. and Smith, G. B. |
| 120 | Wrong outcome | Association of Adherence to Weight Telemonitoring With Health Care Use and Death: A Secondary Analysis of a Randomized Clinical Trial | 2020 | Haynes, Sarah C. and Tancredi, Daniel J. and Tong, Kathleen and Hoch, Jeffrey S. and Ong, Michael K. and Ganiats, Theodore G. and Evangelista, Lorraine S. and Black, Jeanne T. and Auerbach, Andrew and Romano, Patrick S. |
| 121 | Wrong outcome | Association of blood pressure documentation with adverse outcomes in an emergency department in Brazil | 2019 | Daniel, Acqg and Veiga, E. V. and Mafra, Accn |
| 122 | Wrong outcome | Association of blood pressure documentation with adverse outcomes in an emergency department in Brazil | 2019 | Daniel, Ana Carolina Queiroz Godoy and Veiga, Eugenia Velludo and Mafra, Ana Carolina Cintra Nunes |
| 123 | Wrong outcome | Association of pulse pressure with allâ€cause mortality in older Japanese patients with type 2 diabetes mellitus: A observational cohort study | 2023 | Fukunaga, Naoko and Tamakoshi, Koji and Hayashi, Toshio |
| 124 | Wrong outcome | Attitudes and perceptions of registered nurses during and shortly after acute care restructuring in Newfoundland and Labrador | 2005 | Way, C. and Gregory, D. and Baker, N. and Lefort, S. and Barrett, B. and Parfrey, P. |
| 125 | Wrong population | Attitudes and practices towards vital signs monitoring on paediatric wards: Cross-validation of the Ped-V scale | 2022 | Gawronski, O. and Biagioli, V. and Dall'oglio, I. and Cecchetti, C. and Ferro, F. and Tiozzo, E. and Raponi, M. |
| 126 | Wrong outcome | Attitudes of nursing staff towards a Modified Early Warning System | 2015 | Cherry, P. G. and Jones, C. P. |
| 127 | Wrong outcome | Attitudes towards terminal care among the general population and medical practitioners in Japan | 1999 | Miyashita, M. and Hashimoto, S. and Kawa, M. and Kojima, M. |
| 128 | Wrong outcome | Attitudes towards vital signs monitoring in the detection of clinical deterioration: scale development and survey of ward nurses | 2015 | Mok, W. and Wang, W. and Cooper, S. and Ang, E. N. and Liaw, S. Y. |
| 129 | Wrong outcome | Audit of an apoplexy department -- an indispensable tool | 2008 | Pedersen, B. B. and Bock, V. and Plesner, M. |
| 130 | Wrong outcome | Audit of healthy lifestyle behaviors among patients with diabetes and hypertension attending ambulatory health care services in the United Arab Emirates | 2014 | Baynouna, L. M. and Neglekerke, N. J. and Ali, H. E. and ZeinAlDeen, S. M. and Al Ameri, T. A. |
| 131 | Wrong outcome | Audit of the bedside monitor alarms in a critical care unit | 2014 | Morales SÃ¡nchez, C. and Murillo PÃ©rez, M. A. and Torrente Vela, S. and GarcÃ­a Iglesias, M. and Cornejo Bauer, C. and LÃ³pez LÃ³pez, C. and Orejana MartÃ­n, M. and Cuenca Solanas, M. and Alted LÃ³pez, E. |
| 132 | Wrong outcome | An audit of the safety of an acute pain service | 1997 | Tsui, S. L. and Irwin, M. G. and Wong, C. M. and Fung, S. K. and Hui, T. W. and Ng, K. F. and Chan, W. S. and O'Reagan, A. M. |
| 133 | Wrong outcome | Augmenting Critical Care Patient Monitoring Using Wearable Technology: Review of Usability and Human Factors | 2021 | Andrade, E. and Quinlan, L. and Harte, R. and Byrne, D. and Fallon, E. and Kelly, M. and Casey, S. and Kirrane, F. and O'Connor, P. and O'Hora, D. and Scully, M. and Laffey, J. and Pladys, P. and BeuchÃ©e, A. and Ã“Laighin, G. |
| 134 | Wrong outcome | Automated correction of room location errors in anesthesia information management systems | 2008 | Epstein, R. H. and Dexter, F. and Piotrowski, E. |
| 135 | Wrong outcome | Automated detection of physiologic deterioration in hospitalized patients | 2015 | Evans, R. Scott and Kuttler, Kathryn G. and Simpson, Kathy J. and Howe, Stephen and Crossno, Peter F. and Johnson, Kyle V. and Schreiner, Misty N. and Lloyd, James F. and Tettelbach, William H. and Keddington, Roger K. and Tanner, Alden and Wilde, Chelbi and Clemmer, Terry P. |
| 136 | Wrong outcome | Automated detection of physiologic deterioration in hospitalized patients | 2015 | Scott Evans, R. and Kuttler, K. G. and Simpson, K. J. and Howe, S. and Crossno, P. F. and Johnson, K. V. and Schreiner, M. N. and Lloyd, J. F. and Tettelbach, W. H. and Keddington, R. K. and Tanner, A. and Wilde, C. and Clemmer, T. P. |
| 137 | Wrong outcome | Automated Identification of Adults at Risk for In-Hospital Clinical Deterioration | 2020 | Escobar, G. J. and Liu, V. X. and Schuler, A. and Lawson, B. and Greene, J. D. and Kipnis, P. |
| 138 | Wrong outcome | Automated office blood pressure measurement by elderly patients in the waiting room | 2021 | Toba, A. and Ishikawa, J. and Suzuki, A. and Harada, K. |
| 139 | Wrong outcome | Automated office blood pressure-being alone and not location is what matters most | 2015 | Armstrong, D. and Matangi, M. and Brouillard, D. and Myers, M. G. |
| 140 | Wrong outcome | Automated rapid response system activation-Impact on nurses' attitudes and perceptions towards recognising and responding to clinical deterioration: Mixed-methods study | 2023 | Chua, W. L. and Wee, L. C. and Lim, J. Y. G. and Yeo, M. L. K. and Jones, D. and Tan, C. K. and Khan, F. A. and Liaw, S. Y. |
| 141 | Wrong outcome | Automated versus manual blood pressure measurement: A randomized crossover trial in the emergency department of a tertiary care hospital in Karachi, Pakistan: Are third world countries ready for the change? | 2016 | Mansoor, K. and Shahnawaz, S. and Rasool, M. and Chaudhry, H. and Ahuja, G. and Shahnawaz, S. |
| 142 | Wrong population | Automobile trauma in pregnancy: Prevention and treatment | 1996 | Crosby, W. M. |
| 143 | Wrong outcome | Availability of services to transplant recipients | 1993 | McKevitt, P. M. and Norwood, K. |
| 144 | Wrong outcome | Avatar-based patient monitoring improves information transfer, diagnostic confidence and reduces perceived workload in intensive care units: computer-based, multicentre comparison study | 2023 | Bergauer, L. and Braun, J. and Roche, T. R. and Meybohm, P. and Hottenrott, S. and Zacharowski, K. and Raimann, F. J. and Rivas, E. and LÃ³pez-Baamonde, M. and Ganter, M. T. and NÃ¶thiger, C. B. and Spahn, D. R. and Tscholl, D. W. and Akbas, S. |
| 145 | Wrong outcome | Avatar-based versus conventional vital sign display in a central monitor for monitoring multiple patients: a multicenter computer-based laboratory study | 2020 | Garot, O. and RÃ¶ssler, J. and Pfarr, J. and Ganter, M. T. and Spahn, D. R. and NÃ¶thiger, C. B. and Tscholl, D. W. |
| 146 | Wrong outcome | Axillary, Tympanic, and Temporal Thermometry Comparison in a Community Hospital Pediatric Unit | 2018 | Kurnat-Thoma, Emma and Edwards, Vanessa and Emery, Kathleen |
| 147 | Wrong outcome | Barriers and facilitating factors related to use of early warning score among acute care nurses: a qualitative study | 2017 | Petersen, J. A. and Rasmussen, L. S. and Rydahl-Hansen, S. |
| 148 | Wrong outcome | Barriers to Accurate Postoperative Temperature Assessment | 2018 | Scanlan, Zachery |
| 149 | Wrong population | Barriers to early detection of deterioration in hospitalized infants using predictive analytics | 2021 | Sullivan, B. A. and Keim-Malpass, J. |
| 150 | Wrong outcome | A basic needs assessment of Kenyan health care practitioners' training and ability in providing resuscitation management for patients in Mbagathi Hospital, Nairobi | 2013 | Thaler, Adam and Dunlevy, Hillary and Cohn, Jennifer and Speck, Rebecca and O'Brien, Meghan and McCunn, Maureen |
| 151 | Wrong outcome | Battling Alarm Fatigue: Safely Modifying Age-Based Heart Rate and Respiratory Rate Parameters | 2023 | Clemenson, Sally and Tellson, Alaina |
| 152 | Wrong outcome | Bedside analysis of the sublingual microvascular glycocalyx in the emergency room and intensive care unit - the GlycoNurse study | 2018 | Rovas, A. and Lukasz, A. H. and Vink, H. and Urban, M. and Sackarnd, J. and PavenstÃ¤dt, H. and KÃ¼mpers, P. |
| 153 | Wrong outcome | Bedside Blood Transfusion - What Nurses Know and Perform: A Cross-Sectional Study from A Tertiary-Level Cancer Hospital in Rural Kerala | 2021 | Jogi, I. E. and Mohanan, N. and Nedungalaparambil, N. M. |
| 154 | Wrong outcome | Bedside percutaneous tracheostomy performed by pulmonologist in respiratory ICU: A 20-month experience and literature review | 2010 | Baglioni, S. and Iacoacci, C. and Scoscia, E. and Dottorini, M. and Eslami, A. and Penza, O. |
| 155 | Wrong outcome | A before and after study assessing the impact of a new model for recognizing and responding to early signs of deterioration in an acute hospital | 2013 | McDonnell, A. and Tod, A. and Bray, K. and Bainbridge, D. and Adsetts, D. and Walters, S. |
| 156 | Wrong outcome | Behavioral effects and body activity level in female hospital staff nurses during work hour | 1995 | Mathur, K. and Bhattacharya, S. K. and Kashyap, S. K. |
| 157 | Wrong outcome | Benefits of telemonitoring in the care of patients with heart failure | 2012 | Atkin, P. and Barrett, D. |
| 158 | Wrong outcome | Bevacizumab-induced proteinuria and its association with antihypertensive drugs: A retrospective cohort study using a Japanese administrative database | 2023 | Kiyomi, A. and Koizumi, F. and Imai, S. and Yamana, H. and Horiguchi, H. and Fushimi, K. and Sugiura, M. |
| 159 | Wrong outcome | Bioequivalence of vildagliptin tablets in healthy volunteers | 2018 | Jiang, S. Y. and Tong, J. C. and Hu, H. and Wang, M. H. and Xie, H. T. and Shen, J. and Chen, F. H. |
| 160 | Wrong outcome | Biopsia Renal en receptores de Trasplante Renal: Cuidados de enfermerÃ­a y complicaciones | 2015 | Sanz Izquierdo, Esther and Alonso Torres, M. Teresa and Mirada Ariet, Carmen and Franquet Barnils, Esther and Palomino MartÃ­nez, Ana and Facundo Molas, Carme |
| 161 | Wrong outcome | Blood pressure control in patients submitted to cardiac surgery | 2010 | Ponte, Keila Maria de Azevedo and Aragao, Antonia Eliana de Araujo and Marques, Marilia Braga and Ferreira, Adriana Gomes Nogueira and Vasconcelos, Michelle Alves and Silva, Maria Adelane Monteiro |
| 162 | Wrong outcome | Blood pressure cuffs and pulse oximeter sensors: a potential source of cross-contamination | 2009 | Davis, C. |
| 163 | Wrong outcome | Blood pressure decrease prior to initiating pharmacological therapy in nonemergent hypertension | 1990 | Lebby, T. and Paloucek, F. and Dela Cruz, F. and Leikin, J. B. |
| 164 | Wrong outcome | [Blood pressure in 6- to 45-month-old children. Apropos of a study of 264 children from nurseries] | 1988 | BÃ©rard, E. and BouttÃ©, P. and Macone, F. and Albertini, M. and Mariani, R. |
| 165 | Wrong outcome | Blood Pressure Measurements with Different Currently Available Methods in Elderly Hypertensive Hospitalized Patients: A Real World Cross-Sectional Study | 2019 | Del Giorno, R. and Heiniger, P. S. and Balestra, L. and Gabutti, L. |
| 166 | Wrong outcome | Blood Pressure Trends, Demographic Data, Workload, and Lifestyle Factors Among Nurses in the Subcarpathian Region of Poland: A Cross-Sectional Observational Study | 2024 | Bartosiewicz, A. and Åuszczki, E. and Pieczonka, M. and Nowak, J. and Oleksy, Å and Stolarczyk, A. and Lewandowska, A. and Dymek, A. |
| 167 | Wrong outcome | Blood pressure variability with different measurement methods: Reliability and predictors. A proof of concept cross sectional study in elderly hypertensive hospitalized patients | 2019 | Del Giorno, R. and Balestra, L. and Heiniger, P. S. and Gabutti, L. |
| 168 | Wrong outcome | Body temperature measurement methods and targets in Australian and New Zealand intensive care units | 2018 | Cutuli, S. L. and Osawa, E. A. and Glassford, N. J. and Marshall, D. and Eyeington, C. T. and Eastwood, G. M. and Young, P. J. and Bellomo, R. |
| 169 | Wrong outcome | Body-temperature circadian rhythm in 67 patients after heart valve replacement surgery secondary to valvular heart disease | 2014 | Jiang, X. Y. and Wang, C. |
| 170 | Wrong outcome | Breathing better: A techâ€monitored study of positive expiratory pressure and reading aloud for chronic obstructive pulmonary disease | 2023 | Ã–zden, GÃ¼rkan and Parlar KÄ±lÄ±Ã§, Serap |
| 171 | Wrong outcome | Bridging the gap from knowledge to practice; Get Tough! Clean Your Stuff! | 2013 | Schoon, Jenette and Sudoma, Cara |
| 172 | Wrong outcome | Brief report. The effects of crossed leg on blood pressure measurement | 1999 | Foster-Fitzpatrick, L. and Ortiz, A. and Sibilano, H. and Marcantonio, R. and Braun, L. T. |
| 173 | Wrong outcome | The British Rheumatoid Outcome Study Group (BROSG) randomised controlled trial to compare the effectiveness and cost-effectiveness of aggressive versus symptomatic therapy in established rheumatoid arthritis | 2005 | Symmons, D. and Tricker, K. and Roberts, C. and Davies, L. and Dawes, P. and Scott, D. L. |
| 174 | Wrong outcome | BTG-AC: Break-the-Glass Access Control Model for Medical Data in Wireless Sensor Networks | 2016 | Maw, H. A. and Hannan, Xiao and Christianson, B. and Malcolm, J. A. |
| 175 | Wrong outcome | Buccal midazolam and rectal diazepam for treatment of prolonged seizures in childhood and adolescence: a randomised trial | 1999 | Scott, R. C. and Besag, F. M. and Neville, B. G. |
| 176 | Wrong outcome | Building a Real-Time Remote Patient Monitoring Patient Safety Program for COVID-19 Patients | 2022 | Patel, H. and Hassell, A. and Cyriacks, B. and Fisher, B. and Tonelli, W. and Davis, C. |
| 177 | Wrong outcome | Burden of care related to monitoring patient vital signs during intensive care; a descriptive retrospective database study | 2022 | Romare, Charlotte and Anderberg, Peter and Sanmartin Berglund, Johan and SkÃ¤r, Lisa |
| 178 | Wrong outcome | C19TM: A nurse practitioner and physician assistant-led telemonitoring initiative ensures timely transfer of critically ill coronavirus disease 2019 patients | 2021 | Santos, C. D. and Grek, A. A. and Ojard, M. M. and Propst, J. A. and Hastings, J. J. and Krider, T. L. and Villar, D. C. and Sanghavi, D. K. and Freeman, W. D. and Siegel, J. L. |
| 179 | Wrong outcome | CADERNETA DE SAÃšDE DA CRIANÃ‡A: INCOMPLETUDE DOS PARÃ‚METROS AVALIADOS NA CONSULTA | 2021 | Marques, Karolaine Fernanda and Silva, Luana da and dos Santos Silva Canario, MÃ¡rcia Aparecida and Pimenta Ferrari, RosÃ¢ngela Aparecida |
| 180 | Wrong outcome | Calculating Intraoperative Fluid Deficit to Prevent Abdominal Compartment Syndrome in Hip Arthroscopy | 2022 | Yalamanchili, D. R. and Shively, S. and Banffy, M. B. and Taliwal, N. and Clark, E. and Hunter, G. and Mayle, A. and Dumont, G. D. and Westermann, R. W. and Harris, J. D. and Laskovski, J. R. |
| 181 | Wrong outcome | Call 4 Concern: patient and relative activated critical care outreach | 2010 | Odell, M. and Gerber, K. and Gager, M. |
| 182 | Wrong Study Design | Can continuous remote vital sign monitoring reduce the number of room visits to patients suspected of COVID-19: A quasi-experimental study | 2021 | van Goor, H. M. R. and Eddahchouri, Y. and van Loon, K. and Bredie, S. J. H. and Schoonhoven, L. and Kaasjager, H. A. H. and van Goor, H. |
| 183 | Wrong outcome | Can improved software facilitate the wider use of ambulatory blood pressure measurement in clinical practice? | 2004 | O'Brien, E. and Atkins, N. and O'Brien, Eoin and Atkins, Neil |
| 184 | Wrong outcome | Can proactive rapid response team rounding improve surveillance and reduce unplanned escalations in care? A controlled before and after study | 2019 | Danesh, Valerie and Neff, Donna and Jones, Terry L. and Aroian, Karen and Unruh, Lynn and Andrews, Diane and Guerrier, Lotricia and Venus, Sam J. and Jimenez, Edgar |
| 185 | Wrong outcome | Can Sepsis Be Detected in the Nursing Home Prior to the Need for Hospital Transfer? | 2018 | Sloane, Philip D. and Ward, Kimberly and Weber, David J. and Kistler, Christine E. and Brown, Benjamin and Davis, Katherine and Zimmerman, Sheryl |
| 186 | Wrong outcome | Can We Do Something About the Noise in Our Unit?...2018 National Teaching Institute Research Abstracts Presented at the AACN National Teaching Institute in Boston, Massachusetts, May 21-24, 2018 | 2018 | Meehan, Patricia and O'Brien, Mary and Marine, Kathleen and Curley, Martha |
| 187 | Wrong outcome | Can we trust the new generation of infrared tympanic thermometers in clinical practice? | 2013 | Haugan, Berit and Langerud, Anne K. and KalvÃ¸y, HÃ¥vard and FrÃ¸slie, Kathrine F. and Riise, Else and Kapstad, Heidi |
| 188 | Wrong outcome | A Canadian survey of critical care physiciansâ€™ hemodynamic management of deceased organ donors | 2019 | Frenette, A. J. and Charbonney, E. and Dâ€™Aragon, F. and Serri, K. and Marsolais, P. and ChassÃ©, M. and Meade, M. and Williamson, D. |
| 189 | Wrong outcome | Cancellation of elective procedures on the day of surgery | 2018 | Karashi, A. R. and Alsaif, M. and Rashid, F. and Alboosta, H. and Almalki, A. |
| 190 | Wrong outcome | CANConnect Cancer Access Nurse...Cancer Nurses Society of Australia (CNSA), 26th Annual Congress, June 19-21, 2024, Brisbane, Queensland | 2024 | Kourakis, Samantha |
| 191 | Wrong outcome | Capillary blood glucose measurement: Technical, clinical and legal aspects | 2004 | ParramÃ³n, M. |
| 192 | Wrong outcome | Capillary refill time in the hands and feet of normal newborn infants | 1999 | Raju, N. V. and Maisels, M. J. and Kring, E. and Schwarz-Warner, L. |
| 193 | Wrong outcome | Capnography and respiratory depression: is capnography a good way to monitor at-risk postsurgical patients? A prospective trial examines the question | 2008 | Hutchison, R. and Rodriguez, L. |
| 194 | Wrong outcome | Capturing portable medical equipment disinfection data via an automated novel disinfection tracking system | 2021 | Martel, J. A. and Chatterjee, P. and Coppin, J. D. and Williams, M. and Choi, H. and Stibich, M. and Simmons, S. and Passey, D. and Jinadatha, C. |
| 195 | Wrong outcome | Cardiauvergne: Heart failure home management and remote monitoring system | 2014 | Eschalier, R. and D'Agrosa-Boiteux, M. C. and Mannenq, P. H. and Vallot, S. and Bastard, J. P. and Cassagnes, J. |
| 196 | Wrong outcome | Cardiorespiratory physical assessment for the acutely ill: 2 | 2002 | Doucherty, B. |
| 197 | Wrong outcome | Care erosion in sedation assessment: A prospective comparison of usual care Richmond Agitation-Sedation Scale assessment with protocolized assessment for medical intensive care unit patients | 2021 | Anderson, C. C. and Johnson, J. L. and deBoisblanc, B. P. and Jolley, S. E. |
| 198 | Wrong outcome | Carotid endarterectomy: What difference does a clinical protocol make? | 2016 | Chen, Tanghua and Crozier, John A. |
| 199 | Wrong population | Case for change: a standardised inpatient paediatric early warning system in England | 2021 | Roland, D. and Stilwell, P. A. and Fortune, P. M. and Alexander, J. and Clark, S. J. and Kenny, S. |
| 200 | Wrong Study Design | A case of gadobenate dimeglumine-induced anaphylactic shock: a case report | 2021 | Huang, J. and Liu, Z. and Jiang, C. and Xu, L. and Zheng, W. |
| 201 | Wrong population | A case of post-operative abortive malignant hyperthermia | 1995 | Yokoyama, K. and Nanpo, T. and Kumakawa, K. |
| 202 | Wrong outcome | Case report on coronary artery disease | 2020 | Kamdi, P. and Sharma, R. and Singh, S. and Gujar, S. and Patil, M. |
| 203 | Wrong outcome | Case report on- Management and complication of vesicular calculi with obstructive Hydronephrosis with CKD | 2022 | Raut, P. and Kolhe, S. and Munjewar, P. and Pathade, A. |
| 204 | Wrong outcome | Case Study: Daily Review of Telemonitoring Data by a Critical Care Nurse Improves Outcomes in a Patient with Class IV, Stage D Heart Failure with Reduced Ejection Fraction Receiving Home Intravenous Dobutamine for Palliation | 2020 | Houben, Erin and Joiner, Jennifer and Miller, Christine and Prosser, Barbara |
| 205 | Wrong outcome | CE: Treating pneumonia in older patients | 2004 | Doering, P. L. |
| 206 | Wrong outcome | "Center of Geriatric Care" project- the development of the interdisciplinary home-based care model for elderly patients in Gdansk, Poland. Pilot study | 2019 | Wierzba, Karol and Kujawska-Danecka, Hanna and Szalewska, Dominika and Popowski, Piotr and Damps-KonstaÅ„ska, Iwona and Å»arczyÅ„ska-Buchowiecka, Marta and ÅšwiÄ™tnicka, Katarzyna and Hajduk, Adam and StopczyÅ„ska, Iwona and Olszewska-Karaban, Marzena and Zdrojewski, Zbigniew and GruchaÅ‚a, Marcin and Jas, Ewa |
| 207 | Wrong outcome | Central station data displays: An experimental evaluation of observer performance. Part II: Factors affecting performance, and a comparison of analogue and digital data | 1989 | Crew, A. D. and Old, S. and Craig, A. and Unsworth, G. D. and Fletcher, P. C. |
| 208 | Wrong outcome | Cerebral oxygen desaturation monitored by intraoperative near-infrared spectroscopy, and incidence of post-operative cognitive dysfunction: A systematic review protocol | 2013 | Lopez, O. and Gollaher, T. and Riddle, D. |
| 209 | Wrong outcome | Cerebral Perfusion Pressure Variability Between Patients and Between Centres | 2018 | Depreitere, B. and GÃ¼iza, F. and Piper, I. and Citerio, G. and Chambers, I. and Jones, P. A. and Lo, T. M. and Enblad, P. and Nilsson, P. and Feyen, B. and Jorens, P. and Maas, A. and Schuhmann, M. U. and Donald, R. and Moss, L. and Van den Berghe, G. and Meyfroidt, G. |
| 210 | Wrong outcome | The challenges in monitoring and preventing patient safety incidents for people with intellectual disabilities in NHS acute hospitals: evidence from a mixed-methods study | 2014 | Tuffrey-Wijne, I. and Goulding, L. and Gordon, V. and Abraham, E. and Giatras, N. and Edwards, C. and Gillard, S. and Hollins, S. |
| 211 | Wrong outcome | Challenges in the nursing care of intracranial carbapenem-resistant Escherichia coli infection after severe traumatic brain injury: a case report | 2020 | Wang, P. and Gao, B. and Wang, M. and Sheng, Q. and Tu, M. |
| 212 | Wrong outcome | Challenges of customizing electrocardiography alarms in intensive care units: A mixed methods study | 2018 | Ruppel, H. and Funk, M. and Kennedy, H. P. and Bonafide, C. P. and Wung, S. F. and Whittemore, R. |
| 213 | Wrong Study Design | Challenges With Continuous Pulse Oximetry Monitoring and Wireless Clinician Notification Systems After Surgery: Reactive Analysis of a Randomized Controlled Trial | 2019 | Harsha, P. and Paul, J. E. and Chong, M. A. and Buckley, N. and Tidy, A. and Clarke, A. and Buckley, D. and Sirko, Z. and Vanniyasingam, T. and Walsh, J. and McGillion, M. and Thabane, L. |
| 214 | Wrong outcome | Change in Physiological Variables in the Last TwoÂ Weeks of Life: An Observational Study of Hospitalized Adults With Heart Failure | 2018 | Taylor, Paul and Crouch, Simon and Howell, Debra A. and Dowding, Dawn W. and Johnson, Miriam J. |
| 215 | Wrong outcome | Changes in practice and organisation surrounding blood transfusion in NHS trusts in England 1995-2005 | 2008 | Taylor, C. J. and Murphy, M. F. and Lowe, D. and Pearson, M. |
| 216 | Wrong outcome | Changing nurses' views of the therapeutic environment: randomised controlled trial | 2019 | Csipke, E. and Wykes, T. and Nash, S. and Williams, P. and Koeser, L. and McCrone, P. and Rose, D. and Craig, T. |
| 217 | Wrong outcome | The characteristics of ICU physical restraint use and related influencing factors in China: a multi-center study | 2021 | Zhang, C. and Liu, D. and He, Q. |
| 218 | Wrong outcome | Characteristics of patients with cardiorespiratory instability in a step-down unit | 2012 | Yousef, K. and Pinsky, M. R. and DeVita, M. A. and Sereika, S. and Hravnak, M. |
| 219 | Wrong outcome | Characterizing the inpatient care of young adults experiencing early psychosis: A medical record review | 2019 | Puyat, J. H. and Kamieniecki, R. and Vaughan, B. and Mihic, T. and Bonnie, K. and Danielson, J. and Williams, S. |
| 220 | Wrong outcome | CHECKLIST FOR MONITORING OF HEART CATHETERIZATION: A STRATEGY FOR NURSING MANAGEMENT | 2015 | Meira de Sousa, Solange and Bernardino, Elizabeth and Loures Bueno, Ronaldo da Rocha and Nayara Mizuno, Tironi and Alves das MercÃªs, Nen NalÃº and Knop Aued, Gisele |
| 221 | Wrong outcome | [Chemical restraint and nursing care in the intensive care unit] | 2010 | Yu, C. C. and Huang, H. C. |
| 222 | Wrong population | Children's nursing. Review of home paediatric parenteral nutrition in the UK | 2001 | Holden, C. E. |
| 223 | Wrong outcome | Chloral hydrate sedation for auditory brainstem response (ABR) testing in children: Safety and effectiveness | 2016 | Valenzuela, D. G. and Kumar, D. S. and Atkins, C. L. and Beers, A. and Kozak, F. K. and Chadha, N. K. |
| 224 | Wrong outcome | ChroniSense National Early Warning Score Study (CHESS): a wearable wrist device to measure vital signs in hospitalised patients-protocol and study design | 2019 | Van Velthoven, M. H. and Adjei, F. and Vavoulis, D. and Wells, G. and Brindley, D. and Kardos, A. |
| 225 | Wrong outcome | ChroniSense National Early Warning Score Study: Comparison Study of a Wearable Wrist Device to Measure Vital Signs in Patients Who Are Hospitalized | 2023 | Van Velthoven, M. H. and Oke, J. and Kardos, A. |
| 226 | Wrong population | The circadian rhythm of blood pressure in school-age children of normotensive and hypertensive parents | 1989 | Grossman, D. G. S. |
| 227 | Wrong outcome | Cleanliness audit of clinical surfaces and equipment: who cleans what? | 2011 | Anderson, R. E. and Young, V. and Stewart, M. and Robertson, C. and Dancer, S. J. |
| 228 | Wrong outcome | Clinical comparison of automatic, noninvasive measurements of blood pressure in the forearm and upper arm | 2005 | Schell, K. and Bradley, E. and Bucher, L. and Seckel, M. and Lyons, D. and Wakai, S. and Bartell, D. and Carson, E. and Chichester, M. and Foraker, T. and Simpson, K. |
| 229 | Wrong outcome | Clinical comparison of automatic, noninvasive measurements of blood pressure in the forearm and upper arm with the patient supine or with the head of the bed raised 45Â°: a follow-up study | 2006 | Schell, K. and Lyons, D. and Bradley, E. and Bucher, L. and Seckel, M. and Wakai, S. and Carson, E. and Waterhouse, J. and Chichester, M. and Bartell, D. and Foraker, T. and Simpson, E. K. |
| 230 | Wrong outcome | Clinical course of induced hypothermia after cardiac arrest | 2010 | Irigoyen Aristorena, M. I. and YagÃ¼e GastÃ³n, A. and RoldÃ¡n RamÃ­rez, J. |
| 231 | Wrong outcome | Clinical deterioration in patients with ST-elevation myocardial infarction during and for 24Â h after percutaneous coronary intervention: An observational study | 2020 | D'Rosario, D. and Currey, J. and Considine, J. and Cameron, J. |
| 232 | Wrong outcome | Clinical evaluation of a novel respiratory rate monitor | 2016 | Lee, Peter and Lee, Peter J. |
| 233 | Wrong outcome | Clinical evaluation of the Life Support for Trauma and Transport (LSTATâ„¢) platform | 2002 | Johnson, K. and Pearce, F. and Westenskow, D. and Ogden, L. L. and Farnsworth, S. and Peterson, S. and White, J. and Slade, T. |
| 234 | Wrong outcome | Clinical Implementation of Self-Measured Blood Pressure Monitoring, 2015â€“2016 | 2019 | Jackson, S. L. and Ayala, C. and Tong, X. and Wall, H. K. |
| 235 | Wrong outcome | Clinical implications of white-coat effect among patients attending at a lipid clinic | 2008 | Bo, M. and Comba, M. and Canade, A. and Brescianini, A. and Corsinovi, L. and Astengo, M. A. and Sona, A. and Fonte, G. |
| 236 | Wrong outcome | Clinical indicators to monitor patients with risk for ineffective cerebral tissue perfusion | 2015 | de Abreu Almeida, Miriam and Barragan da Silva, Marcos and Paulsen Panato, Bruna and de Oliveira Siqueira, Ana Paula and Palma da Silva, Mariana and Engelman, Bruna and Marques Severo, Isis and Gaedke Nomura, Aline Tsuma |
| 237 | Wrong outcome | A Clinical Monitoring Approach for Early Onset Sepsis: A Community Hospital Experience | 2022 | Bain, L. and Sivakumar, D. and McCallie, K. and Balasundaram, M. and Frymoyer, A. |
| 238 | Wrong outcome | Clinical Mortality in a Large COVID-19 Cohort: Observational Study | 2020 | Jarrett, Mark and Schultz, Susanne and Lyall, Julie and Wang, Jason and Stier, Lori and Geronimo, Marcella De and Nelson, Karen and De Geromino, Marcella and De Geronimo, Marcella |
| 239 | Wrong outcome | Clinical Mortality Review in a Large COVID-19 Cohort | 2020 | Jarrett, M. P. and Schultz, S. F. and Lyall, J. S. and Wang, J. J. and Stier, L. and De Geronimo, M. and Nelson, K. L. |
| 240 | Wrong outcome | Clinical Nurse Specialist-Driven Practice Change | 2017 | Derby, Kelly M. and Hartung, Natalie A. and Wolf, Sherry L. and Zak, Heather L. and Evenson, Laura K. |
| 241 | Wrong outcome | Clinical nursing for the application of continuous renal replacement therapy in the intensive care unit | 2009 | Baldwin, I. and Fealy, N. |
| 242 | Wrong population | Clinical Outcomes Associated With a Remote Postpartum Hypertension Monitoring Program | 2024 | Lemon, L. S. and Quinn, B. and Binstock, A. and Larkin, J. C. and Simhan, H. N. and Hauspurg, A. |
| 243 | Wrong outcome | Clinical pathways for patients with chronic heart failure: the nursing focus | 2008 | Holzmann, N. and Panfil, E. |
| 244 | Wrong outcome | Clinical predictors of duration of action of cisatracurium and rocuronium administered long-term | 2009 | Fassbender, P. and Geldner, G. and Blobner, M. and Hofmockel, R. and Rex, C. and Gautam, S. and Malhotra, A. and Eikermann, M. |
| 245 | Wrong outcome | Clinical relevance of routinely measured vital signs in hospitalized patients: a systematic review | 2014 | Storm-Versloot, M. N. and Verweij, L. and Lucas, C. and Ludikhuize, J. and Goslings, J. C. and Legemate, D. A. and Vermeulen, H. |
| 246 | Wrong outcome | Clinical rounds. Heart failure: an overview of consensus guidelines and nursing implications | 2003 | Svendsen, A. |
| 247 | Wrong outcome | Clinical-epidemiological profile of patients undergoing cardiac catheterization procedures at a university hospital in Rio de Janeiro state | 2019 | Marotto Vila, Karolyne and GonÃ§alves Rocha, Ronilson and de Oliveira Carvalho Naves, Camila BenicÃ¡ and Ferreira de Almeida, Luana and Bertolossi Marta, Cristiano and Rocha Oliveira, Claudia Silvia |
| 248 | Wrong outcome | Clinician blood pressure documentation of stable intensive care patients: an intelligent archiving agent has a higher association with future hypotension | 2011 | Hug, C. W. and Clifford, G. D. and Reisner, A. T. |
| 249 | Wrong population | Clinician-Driven Design of VitalPAD-An Intelligent Monitoring and Communication Device to Improve Patient Safety in the Intensive Care Unit | 2018 | Flohr, L. and Beaudry, S. and Johnson, K. T. and West, N. and Burns, C. M. and Ansermino, J. M. and Dumont, G. A. and Wensley, D. and Skippen, P. and Gorges, M. |
| 250 | Wrong population | Clinicians' perspectives on wearable sensor technology as an alternative bedside monitoring tool in two West African countries | 2023 | Ghomrawi, H. M. and Many, B. T. and Holl, J. L. and Ahmed, A. G. and Jackson, M. E. and Sibley, J. and Khan, R. and Kaufmann, E. E. and Appeadu-Mensah, W. and Abdullah, F. |
| 251 | Wrong outcome | Clonidine addition prolongs the duration of caudal analgesia | 2006 | Yildiz, T. S. and Korkmaz, F. and Solak, M. and Toker, K. |
| 252 | Wrong outcome | A cognitive task analysis of final year nursing students' situation awareness in simulated deteriorating patient events: A mixed methods study | 2024 | Walshe, N. and Drennan, J. and Hegarty, J. and O'Brien, S. and Crowley, C. and Ryng, S. and O'Connor, P. |
| 253 | Wrong outcome | Combination of comprehensive thermal care and detail-oriented nursing care in the operating room for managing gestational diabetes mellitus | 2024 | Pan, Y. Y. and Zhang, Q. X. |
| 254 | Wrong outcome | Combination therapy with rituximab and methotrexate in the management of rheumatoid arthritis | 2019 | Al-Samman, D. and Al-Asaady, N. and Al-Jader, S. |
| 255 | Wrong outcome | Combining the Nurse Intuition Patient Deterioration Scale with the National Early Warning Score provides more Net Benefit in predicting serious adverse events: A prospective cohort study in medical, surgical, and geriatric wards | 2024 | Haegdorens, F. and Lefebvre, J. and Wils, C. and Franck, E. and Van Bogaert, P. |
| 256 | Background Article | Commentary on Profits with a purpose: an interview with Tom Chapman [original article by Nichols N appears in HARVARD BUS REV 1992;70(6):87-95] | 1993 | Pinkerton, S. |
| 257 | Wrong outcome | Community based trial of home blood pressure monitoring with nurse-led telephone support in patients with stroke or transient ischaemic attack recently discharged from hospital | 2008 | Kerry, S. and Markus, H. and Khong, T. and Doshi, R. and Conroy, R. and Oakeshott, P. |
| 258 | Wrong outcome | Community-based parenteral anti-infective therapy (CoPAT). Pharmacokinetic and monitoring issues | 1998 | Williams, D. N. and Raymond, J. L. |
| 259 | Wrong outcome | Comparative effectiveness of home blood pressure telemonitoring (HBPTM) plus nurse case management versus HBPTM alone among Black and Hispanic stroke survivors: Study protocol for a randomized controlled trial | 2015 | Spruill, T. M. and Williams, O. and Teresi, J. A. and Lehrer, S. and Pezzin, L. and Waddy, S. P. and Lazar, R. M. and Williams, S. K. and Jean-Louis, G. and Ravenell, J. and Penesetti, S. and Favate, A. and Flores, J. and Henry, K. A. and Kleiman, A. and Levine, S. R. and Sinert, R. and Smith, T. Y. and Stern, M. and Valsamis, H. and Ogedegbe, G. |
| 260 | Wrong outcome | Comparing mean and single automated office blood pressure measurement in a US ambulatory care setting | 2022 | Buettner, Tammy and Bertram, Amanda and Floyd, Olivia and Dowd-Green, Caitlin and Stewart, Rosalyn |
| 261 | Wrong outcome | Comparing the Effect of Virtual Reality and Rhythmic Breathing on the Physiologic Parameters of Patient's Candidate for Angiography: A Clinical Trial Study | 2023 | Pouryousef, Faezeh and Navidian, Ali and Yaghoubi, Saeedeh and Yaghoubinia, Fariba |
| 262 | Wrong outcome | Comparing three methods of assessing peripheral perfusion in critically ill children | 2009 | Ridling, D. A. and Kroon, L. |
| 263 | Wrong outcome | Comparison between telephone and outpatient nursing management in patients with chronic heart failure in a large territorial area in Piedmont, Italy | 2010 | Mainardi, L. and Lazzolino, E. and Asteggiano, R. and Lusardi, R. and Varbella, F. and Sasso, L. and Conte, M. R. |
| 264 | Wrong outcome | Comparison between Temperature Watch and Mercury Thermometer in Monitoring Temperature of Low-Birth-Weight Newborns at Kangaroo Mother Ward Children Hospital Chandaka Medical College Larkana | 2022 | Jamro, S. and Jamro, F. S. and Bhojwani, S. L. and Qureshi, R. and Gemnani, V. K. and Mugheri, D. J. |
| 265 | Wrong outcome | Comparison of a novel clinical score to estimate the risk of REsidual neuromuscular block Prediction Score and the last train-of-four count documented in the electronic anaesthesia record: A retrospective cohort study of electronic data on file | 2018 | Rudolph, MaÃ­ra I. and Ng, Pauline Y. and Hao, Deng and Scheffenbichler, Flora T. and Grabitz, Stephanie D. and Wanderer, Jonathan P. and Houle, Timothy T. and Eikermann, Matthias and Deng, Hao |
| 266 | Wrong outcome | Comparison of acceptability of and preferences for different methods of measuring blood pressure in primary care | 2002 | Little, P. and Barnett, J. and Barnsley, L. and Marjoram, J. and Fitzgerald-Barron, A. and Mant, D. |
| 267 | Wrong outcome | A comparison of an evidence based regime with the standard protocol for monitoring postoperative observation: a randomised controlled trial | 2005 | Fernandez, R. and Griffiths, R. |
| 268 | Wrong outcome | Comparison of blood pressure and pulse readings measured on a bare arm, a clothed arm and on an arm with a rolled-up sleeve | 2020 | TuÄŸrul, E. and KaraÃ§am, Z. |
| 269 | Wrong outcome | Comparison of different methods of blood pressure measurements | 2007 | Chrubasik, S. and Droste, C. and Glimm, E. and Black, A. |
| 270 | Wrong outcome | Comparison of health record vitals and continuously acquired vitals data identifies key differences in clinical impression | 2020 | Olson, D. M. and Dombrowski, K. and Lynch, C. and Mace, B. and Sinha, R. and Spainhour, S. and Naglich, M. and Riemen, K. and Kolls, B. J. |
| 271 | Wrong population | A comparison of kangaroo mother care and conventional incubator care for thermal regulation of infants < 2000 g in Nigeria using continuous ambulatory temperature monitoring | 2004 | Ibe, O. E. and Austin, T. and Sullivan, K. and Fabanwo, O. and Disu, E. and Costello, A. M. |
| 272 | Wrong population | A comparison of kangaroo mother care and conventional incubator care for thermal regulation of infants <2000 g in Nigeria using continuous ambulatory temperature monitoring | 2004 | Ibe, O. E. and Austin, T. and Sullivan, K. and Fabanwo, O. and Disu, E. and Costello, A. M. deL |
| 273 | Wrong outcome | A comparison of noninvasive body temperature monitoring devices in the PACU | 1994 | Darm, R. M. and Hecker, R. B. and Rubal, B. J. |
| 274 | Wrong outcome | Comparison of patient perceptions of Telehealth-supported and specialist nursing interventions for early stage COPD: a qualitative study | 2016 | Fitzsimmons, D. A. and Thompson, J. and Bentley, C. L. and Mountain, G. A. |
| 275 | Wrong outcome | Comparison of policies for recognising and responding to clinical deterioration across five Victorian health services | 2018 | Considine, J. and Hutchison, A. F. and Rawson, H. and Hutchinson, A. M. and Bucknall, T. and Dunning, T. and Botti, M. and Duke, M. M. and Street, M. |
| 276 | Wrong outcome | Comparison of self- and nurse-measured office blood pressure in patients with chronic kidney disease | 2020 | Tougaard, B. G. and Laursen, K. S. and Jensen, J. D. and Buus, N. H. |
| 277 | Wrong outcome | Comparison of the effectiveness and safety of two methods of endotracheal suctioning | 2009 | Kostaki, Z. and Giakoumidakis, K. and Baltopoulos, G. I. and Anthopoulos, G. and Brokalaki-Pananoudaki, H. |
| 278 | Wrong outcome | Comparison of the heart and breathing rate of acutely ill medical patients recorded by nursing staff with those measured over 5min by a piezoelectric belt and ECG monitor at the time of admission to hospital | 2011 | Kellett, J. and Li, M. and Rasool, S. and Green, G. C. and Seely, A. |
| 279 | Wrong outcome | A comparison of the incidence of hypercapnea in non-obese and morbidly obese peri-operative patients using the SenTec transcutaneous pCO2 monitor | 2014 | Soto, R. G. and Davis, M. and Faulkner, M. J. |
| 280 | Wrong outcome | Comparison of the quality and timeliness of vital signs data using three different data-entry devices | 2010 | Wager, K. A. and Schaffner, M. J. and Foulois, B. and Swanson Kazley, A. and Parker, C. and Walo, H. |
| 281 | Wrong outcome | Comparison of the quality of diabetes care in primary care diabetic clinics and general practice clinics | 2005 | Al Khaja, K. A. J. and Sequeira, R. P. and Damanhori, A. H. H. |
| 282 | Wrong outcome | Comparison of three sites to check the pulse and count heart rate in hypotensive infants | 2006 | Sarti, A. and Savron, F. and Ronfani, L. and Pelizzo, G. and Barbi, E. |
| 283 | Wrong outcome | Comparison of two non-invasive body temperature measurement methods for the detection of febrile neutropenia in children with cancer | 2024 | Kavlak, R. S. and AktaÅŸ, E. |
| 284 | Wrong population | Comparison of Two Pediatric Early Warning Systems: A Randomized Trial | 2019 | Jensen, Claus Sixtus and Olesen, Hanne Vebert and Aagaard, Hanne and Svendsen, Marie Louise Overgaard and Kirkegaard, Hans |
| 285 | Wrong outcome | Comparison of tympanic and rectal thermometry: diagnosis of neonatal hypothermia in Uganda | 2004 | Bergstrom, A. and Byaruhanga, R. and Okong, P. |
| 286 | Wrong outcome | Comparison of venous hemoglobin saturation measurements obtained by in vivo oximetry and calculated from blood gas analysis in critically ill dogs | 2021 | Walton, R. A. L. and Hansen, B. |
| 287 | Wrong outcome | Comparisons of acute physiological parameters influencing outcome in patients with traumatic brain injury and hemorrhagic stroke | 2009 | Seo, W. and Oh, H. |
| 288 | Wrong outcome | Complications associated with the use of radial arterial catheters in relation to their length: Does size matter? | 2023 | Watts, F. A. and OrdoÃ±ez, A. and GonzÃ¡lez, A. and Gomez, K. J. D. and Carvajal, D. and Quintero, J. and Uribe-Buritica, F. L. |
| 289 | Wrong outcome | A comprehensive anesthesia simulation environment: re-creating the operating room for research and training | 1988 | Gaba, D. M. and DeAnda, A. |
| 290 | Wrong outcome | Computed Tomography Practice Standards for Severe Pediatric Traumatic Brain Injury in the Emergency Department: a National Survey | 2021 | Yoo, G. and Leach, A. and Woods, R. and Holt, T. and Hansen, G. |
| 291 | Wrong outcome | Computer assisted monitoring in intensive medicine | 1978 | Lauwers, P. and Ferdinande, P. and Van de Walle, J. and Willems, J. and Theunissen, W. |
| 292 | Wrong outcome | Computer based haemodynamic guidance system is effective and safe in management of postoperative cardiac surgery patients | 2011 | Pellegrino, V. A. and Mudaliar, Y. and Gopalakrishnan, M. and Horton, M. D. and Killick, C. J. and Parkin, W. G. and Playford, H. R. and Raper, R. F. |
| 293 | Wrong population | Computer-assisted prescription improves the delay of morphine infusion after nurse triage in patients admitted in the emergency department with intense pain: A randomized trial | 2006 | Lecomte, F. and Oppenheimer, A. and Ginsburg, C. and Dhainaut, J. F. and Claessens, Y. E. |
| 294 | Wrong population | Computer-based patient monitoring | 1982 | Prakash, O. and Meij, S. and Zeelenberg, C. and van der Borden, B. |
| 295 | Wrong population | The Connection Between Caring, Knowing and Preventing Failure to Rescue in Nursing | 2024 | Parker, Carlo |
| 296 | Wrong outcome | Conscious sedation in a specialty hospital | 1994 | Covell, C. A. and Annand, F. |
| 297 | Wrong outcome | Constructing Inpatient Pressure Injury Prediction Models Using Machine Learning Techniques | 2020 | Hu, Y. H. and Lee, Y. L. and Kang, M. F. and Lee, P. J. |
| 298 | Wrong outcome | Construction and application of an ICU nursing electronic medical record quality control system in a Chinese tertiary hospital: a prospective controlled trial | 2024 | Zhang, S. and Quan, Y. Y. and Chen, J. |
| 299 | Background Article | Contactless Body Temperature Monitoring of In-Patient Department (IPD) Using 2.4Â GHz Microwave Frequency via the Internet of Things (IoT) Network | 2022 | Boonsong, W. and Senajit, N. and Prasongchan, P. |
| 300 | Wrong outcome | Contactless Heartbeat Measurement Using Speckle Vibrometry | 2022 | Que, S. and Verkruijsse, W. and van Gastel, M. and Stuijk, S. |
| 301 | Wrong outcome | Contactless monitoring of respiratory rate (RR) and heart rate (HR) in non-acuity settings: a clinical validity study | 2022 | Varma, M. and Sequeira, T. and Naidu, N. K. S. and Mallya, Y. and Sunkara, A. and Patil, P. and Poojary, N. and Vaidyanathan, M. K. and Balmaekers, B. and Thomas, J. and Prasad, N. S. and Badagabettu, S. |
| 302 | Wrong outcome | A Contemporary Review of Community and Transitional Heart Failure Programs | 2023 | Fahd, Saad and Kalsi, Mandeep Singh and Vaddadi, Gautam and Sharma, Naveen |
| 303 | Wrong outcome | Content validity of an instrument to document recovery of patients in past anesthesia care unit | 2007 | Cunha, A. L. S. and Peniche, A. C. G. |
| 304 | Wrong outcome | Continuity of Nursing Care in Patients with Coronary Artery Disease: A Systematic Review | 2022 | Posadas-Collado, G. and Membrive-JimÃ©nez, M. J. and Romero-BÃ©jar, J. L. and GÃ³mez-Urquiza, J. L. and AlbendÃ­n-GarcÃ­a, L. and Suleiman-Martos, N. and CaÃ±adas-De La Fuente, G. A. |
| 305 | Wrong outcome | Continuous Glucose Monitoring for Patients with COVID-19 Pneumonia: Initial Experience at a Tertiary Care Center | 2023 | Dumitrascu, A. G. and Perry, M. F. and Boone, R. J. and Guzman, M. P. and Chirila, R. M. and McNally, A. W. and Colibaseanu, D. T. and Meek, S. E. and Ball, C. T. and White, L. J. and Chindris, A. M. |
| 306 | Wrong outcome | Continuous monitoring after laparoscopic Roux-En-Y gastric bypass: a pathway to ambulatory care surgery - a pilot study | 2024 | Ferreira-Santos, R. and Pinto, J. P. and Pinho, J. P. and Ribeiro, A. C. and da Costa, M. and Vieira, V. and Ferreira, C. and Manso, F. and Pereira, J. C. |
| 307 | Wrong Study Design | Continuous monitoring in COVID-19 care: a retrospective study in time of crisis | 2021 | de Ree, R. and Willemsen, J. and Te Grotenhuis, G. and de Ree, R. and Kolkert, J. and Peppelman, M. |
| 308 | Wrong Study Design | Continuous Monitoring of Respiratory Rate with Wearable Sensor in Patients Admitted to Hospital with Pneumonia Compared with Intermittent Nurse-Led Monitoring in the United Kingdom: A Cost-Utility Analysis | 2022 | Javanbakht, M. and Moradi-Lakeh, M. and Mashayekhi, A. and Atkinson, J. |
| 309 | Wrong Study Design | Continuous Monitoring of Vital Signs Using Wearable Devices on the General Ward: Pilot Study | 2017 | Weenk, M. and van Goor, H. and Frietman, B. and Engelen, L. J. and van Laarhoven, C. J. and Smit, J. and Bredie, S. J. and van de Belt, T. H. |
| 310 | Wrong Study Design | Continuous monitoring of vital signs with the Everion biosensor on the surgical ward: a clinical validation study | 2021 | Haveman, M. E. and van Melzen, R. and Schuurmann, R. C. L. and El Moumni, M. and Hermens, H. J. and Tabak, M. and de Vries, J. P. M. |
| 311 | Wrong population | Continuous non-contact vital sign monitoring in neonatal intensive care unit | 2014 | Villarroel, M. and Guazzi, A. and Jorge, J. and Davis, S. and Watkinson, P. and Green, G. and Shenvi, A. and McCormick, K. and Tarassenko, L. |
| 312 | Wrong outcome | Continuous oximetry/capnometry monitoring reveals frequent desaturation and bradypnea during patient-controlled analgesia | 2007 | Overdyk, F. J. and Carter, R. and Maddox, R. R. and Callura, J. and Herrin, A. E. and Henriquez, C. |
| 313 | Wrong outcome | Continuous Physiological Monitoring Improves Patient Outcomes | 2021 | Stellpflug, Courtney and Pierson, Laura and Roloff, Devin and Mosman, Elton and Gross, Tera and Marsh, Scott and Willis, Valerie and Gabrielson, Donald |
| 314 | Wrong outcome | Continuous physiological signal measurement over 24-hour periods to assess the impact of work-related stress and workplace violence | 2023 | Hamidi Shishavan, H. and Garza, J. and Henning, R. and Cherniack, M. and Hirabayashi, L. and Scott, E. and Kim, I. |
| 315 | Wrong outcome | Continuous vancomycin infusion, a new posological scheme for intensive care units | 2005 | Alonso-FernÃ¡ndez, M. A. and EstÃ©banez-Montiel, M. B. and Rico-Cepeda, M. P. and CatalÃ¡n-GonzÃ¡lez, M. and Montejo-GonzÃ¡lez, J. C. |
| 316 | Wrong outcome | Continuous versus intermittent physiological monitoring for acute stroke | 2013 | Ciccone, A. and Celani, M. G. and Chiaramonte, R. and Rossi, C. and Righetti, E. |
| 317 | Wrong Study Design | Continuous Versus Intermittent Vital Signs Monitoring Using a Wearable, Wireless Patch in Patients Admitted to Surgical Wards: Pilot Cluster Randomized Controlled Trial | 2018 | Downey, C. and Randell, R. and Brown, J. and Jayne, D. G. |
| 318 | Background Article | Continuous ward monitoring and intensive postoperative management | 2024 | Li, K. and Wang, J. and Sessler, D. I. |
| 319 | Wrong outcome | Contrast media induced nephropathy: A literature review of the available evidence and recommendations for practice | 2014 | Deek, Hiba and Newton, Phillip and Sheerin, Noella and Noureddine, Samar and Davidson, Patricia M. |
| 320 | Wrong outcome | Contribution of proactive management of healthcare risks to the reduction of adverse events in a maternity hospital | 2024 | Negrini, R. and Appel, L. C. and Beck, A. P. A. and Eisencraft, A. C. G. and Fascina, L. P. and Fernandes, F. P. |
| 321 | Wrong outcome | Controlling the exotic diseases: II Nursing management | 1980 | Best, H. R. and Clayton, A. J. |
| 322 | Wrong outcome | Coronavirus disease 2019 (COVID-19) complicated with acute myocardial infarction: etiology and nursing experience of three case reports | 2021 | Lei, Y. and Wang, Y. and Song, Y. and Cai, Y. |
| 323 | Wrong outcome | [Corporal hygiene of the critical patient. Adverse effects] | 2010 | Planas Canals, M. |
| 324 | Wrong outcome | Cost analysis of glatiramer acetate vs. fingolimod for the treatment of patients with relapsing-remitting multiple sclerosis in Spain | 2013 | Sanchez-de la Rosa, R. and Sabater, E. and Casado, M. A. |
| 325 | Wrong outcome | Cost effectiveness of a telerehabilitation program to support chronically ill and disabled elders in their homes | 2009 | Bendixen, R. M. and Levy, C. E. and Olive, E. S. and Kobb, R. F. and Mann, W. C. |
| 326 | Wrong outcome | Cost of hospital care for elderly at risk of falling | 2005 | Titler, M. and Dochterman, J. and Picone, D. M. and Everett, L. and Xie, X. J. and Kanak, M. and Fei, Q. |
| 327 | Wrong outcome | Cost of nursing most frequent procedures performed on severely burned patients | 2017 | De Oliveira Melo, Talita and Fernandes Costa Lima, AntÃ´nio |
| 328 | Wrong outcome | Cost of nursing most frequent procedures performed on severely burned patients | 2017 | Melo, T. O. and Lima, A. F. C. |
| 329 | Wrong outcome | Cost-effectiveness of telehealth with remote patient monitoring for postpartum hypertension | 2022 | Niu, B. and Mukhtarova, N. and Alagoz, O. and Hoppe, K. |
| 330 | Wrong outcome | Costs and benefits of personalized healthcare for patients with chronic heart failure in the care and education program "Telemedicine for the Heart" | 2012 | Sohn, S. and Helms, T. M. and Pelleter, J. T. and MÃ¼ller, A. and KrÃ¶ttinger, A. I. and SchÃ¶ffski, O. |
| 331 | Wrong outcome | Costs of inadvertent perioperative hypothermia in Australia: A cost-of-illness study | 2020 | Ralph, Nicholas and Gow, Jeffrey and Conway, Aaron and Duff, Jed and Edward, Karen-Leigh and Alexander, Kim and BrÃ¤uer, Anselm |
| 332 | Wrong outcome | Costs vs quality in different types of primary care settings | 1994 | Starfield, B. and Powe, N. R. and Weiner, J. R. and Stuart, M. and Steinwachs, D. and Scholle, S. H. and Gerstenberger, A. |
| 333 | Wrong outcome | Coupling wearable devices and decision theory in the united states emergency department triage process: A narrative review | 2020 | Nino, V. and Claudio, D. and Schiel, C. and Bellows, B. |
| 334 | Wrong outcome | Course and cares of intestinal transplant in immediate post-operative period | 2007 | Frade Mera, M. J. and Jacue Izquierdo, S. and Fontan Vinagre, G. and Montejo GonzÃ¡lez, J. C. |
| 335 | Wrong outcome | COVID-19 Proactive Disease Management Using COVID Virtual Hospital in a Rural Community | 2023 | Loomis, G. and Rhodes, R. and Bujold, E. and Sharafsaleh, G. and Collett, E. and Irwin, M. and Staton, E. W. and Westfall, J. M. |
| 336 | Wrong outcome | COVID-19 teleassistance and teleconsultation: a matched case-control study (MIRATO project, Lombardy, Italy) | 2023 | Bernocchi, P. and Crotti, G. and Beato, E. and Bonometti, F. and Giudici, V. and Bertolaia, P. and Perger, E. and Remuzzi, A. and Bachetti, T. and La Rovere, M. T. and Dalla Vecchia, L. A. and Angeli, F. and Parati, G. and Borghi, G. and Vitacca, M. and Scalvini, S. |
| 337 | Wrong outcome | Creation of a temperature stability database for refrigerated medications | 2010 | Davis, S. R. and Anderson, E. A. |
| 338 | Foreign Language | Crise hipertensiva: competÃªncias elencadas pelo enfermeiro para o atendimento em hospitais de Curitiba-PR | 2014 | de Moraes, Eliane Oliveira and Nunes, Luana Stephanie de Almeida and CaveiÃ£o, Cristiano and Visentin, Angelita and Hey, Ana Paula and de Oliveira, Vanessa Bertoglio Comassetto Antunes |
| 339 | Wrong outcome | Criteria-based audit to improve quality of care of foetal distress: standardising obstetric care at a national referral hospital in a low resource setting, Tanzania | 2016 | Mgaya, A. H. and Litorp, H. and Kidanto, H. L. and NystrÃ¶m, L. and EssÃ©n, B. |
| 340 | Wrong Study Design | A critical assessment of monitoring practices, patient deterioration, and alarm fatigue on inpatient wards: a review | 2014 | Curry, J. P. and Jungquist, C. R. |
| 341 | Wrong outcome | Critical care capacity in Addis Ababa, Ethiopia: A citywide survey of public hospitals | 2021 | Laytin, A. D. and Sultan, M. and Debebe, F. and Walelign, Y. and Fisseha, G. and Gebreyesus, A. |
| 342 | Wrong outcome | Critical Care Network in the State of Qatar | 2019 | Hijjeh, M. and Al Shaikh, L. and Alinier, G. and Selwood, D. and Malmstrom, F. and Hassan, I. F. |
| 343 | Wrong outcome | Critical care nurses' knowledge of arterial pressure monitoring | 2001 | McGhee, B. H. and Woods, S. L. |
| 344 | Wrong outcome | Critical care nurses' perceptions of appropriate care of the patient with orders not to resuscitate | 1995 | Sherman, D. A. and Branum, K. |
| 345 | Wrong population | Critical incidents related to opioid infusions in children: a five-year review and analysis | 2014 | West, N. and Nilforushan, V. and Stinson, J. and Ansermino, J. M. and Lauder, G. |
| 346 | Wrong outcome | CRITICAL PATIENT TRANSPORT: A C HALLENGE FOR THE 21ST CENTURY | 2017 | Aguiar Carneiro, Tatiane and da PaixÃ£o Duarte, Tayse TÃ¢mara and da Silva Magro, Marcia Cristina |
| 347 | Wrong outcome | Cross-mapping ICNP terms with Taiwanese gynecological nursing records | 2006 | Kuo, C. and Yen, M. |
| 348 | Wrong outcome | Cross-mapping ICNP terms with Taiwanese gynecological nursing records | 2006 | Kuo, C. H. and Yen, M. |
| 349 | Wrong outcome | Cross-mapping of nursing diagnoses and interventions in decompensated heart failure | 2022 | Padua, B. L. R. and Tinoco, Jmvp and Dias, B. F. and Carmo, T. G. D. and Flores, P. V. P. and Cavalcanti, A. C. D. |
| 350 | Wrong outcome | Cross-mapping of nursing diagnoses and interventions in decompensated heart failure | 2022 | Lins Rocha de Padua, Bruna and de Melo Vellozo Pereira Tinoco, Juliana and Fernandes Dias, Beatriz and Gomes do Carmo, Thalita and Peclat Flores, Paula Vanessa and Dantas Cavalcanti, Ana Carla |
| 351 | Wrong outcome | Cross-mapping of nursing diagnoses and interventions in decompensated heart failure | 2022 | Padua, B. L. R. and Tinoco, J. M. V. P. and Dias, B. F. and Carmo, T. G. D. and Flores, P. V. P. and Cavalcanti, A. C. D. |
| 352 | Wrong outcome | Cross-sectoral collaboration by telehealth for prevention of acute admissions in elderly | 2016 | Rasmussen, Janne and Andersen-Ranberg, Karen and Fournaise, Anders |
| 353 | Wrong outcome | Cuff Management Practices In Adult Intensive Care Units In Turkey | 2022 | Kin, Ozlem Kardas and Yesilbalkan, Oznur Usta and Akyol, Asiye |
| 354 | Wrong outcome | Cuff pressure monitoring by manual palpation in intubated patients: How accurate is it? A manikin simulation study | 2017 | Giusti, Gian Domenico and Rogari, Cecilia and Gili, Alessio and Nisi, Fulvio |
| 355 | Foreign Language | Cuidados de enfermagem ao utente sob ventilaÃ§Ã£o mecÃ¢nica internado em unidade de terapia intensiva | 2014 | Melo, Elizabeth Mesquita and Teixeira, Carlos Santos and Oliveira, RogÃ©ria Terto de and Almeida, Diva Teixeira de and Veras, Joelna Eline Gomes Lacerda de Freitas and Frota, Natasha Marques and Studart, Rita MÃ´nica Borges |
| 356 | Wrong outcome | Current practice and knowledge of nurses regarding patient temperature measurement | 2006 | Evans, J. and Kenkre, J. |
| 357 | Wrong outcome | Current practices and evaluation of barriers and facilitators to surgical site infection prevention measures in Jimma, Ethiopia | 2021 | Berman, L. R. and Lang, A. and Gelana, B. and Starke, S. and Siraj, D. and Yilma, D. and Shirley, D. |
| 358 | Wrong outcome | Current state of sedation, analgesia and blood glucose management in intensive care units of county hospitals: A multicenter cross-sectional survey in Guizhou Province of China | 2019 | Xu, L. and Jie, X. and Yumei, C. and Ying, L. and Difen, W. |
| 359 | Wrong outcome | [Current state of sedation, analgesia and blood glucose management in intensive care units of county hospitals: a multicenter cross-sectional survey in Guizhou Province of China] | 2019 | Liu, X. and Xiong, J. and Cheng, Y. and Liu, Y. and Wang, D. |
| 360 | Wrong population | Dangers of oxytocin-induced labour to fetuses | 1974 | Liston, W. A. and Campbell, A. J. |
| 361 | Wrong outcome | Data Mining Models for Automatic Problem Identification in Intensive Medicine | 2022 | Quesado, I. and Duarte, J. and Silva, Ã and Manuel, M. and Quintas, C. |
| 362 | Wrong outcome | Data-driven implementation of alarm reduction interventions in a cardiovascular surgical ICU | 2017 | Allan, S. H. and Doyle, P. A. and Sapirstein, A. and Cvach, M. |
| 363 | Wrong outcome | Decay in quality of closed-chest compressions over time | 1995 | Hightower, D. and Thomas, S. H. and Stone, C. K. and Dunn, K. and March, J. A. and Hightower, D. and Thomas, S. H. and Stone, C. K. and Dunn, K. and March, J. A. |
| 364 | Wrong outcome | Decay in quality of closed-chest compressions over time | 1995 | Hightower, D. and Thomas, S. H. and Stone, C. K. and Dunn, K. and March, J. A. |
| 365 | Wrong outcome | Decrease in as-needed sedative use by limiting nighttime sleep disruptions from hospital staff | 2010 | Bartick, M. C. and Thai, X. and Schmidt, T. and Altaye, A. and Solet, J. M. |
| 366 | Wrong outcome | Decreasing inconsistent alarms notifications: a pragmatic clinical trial in a post-anesthesia care unit | 2024 | Silveira, S. Q. and Nersessian, R. S. F. and Abib, A. D. C. V. and Santos, L. B. and Bellicieri, F. N. and Botelho, K. K. and Lima, H. D. O. and Queiroz, R. M. D. and Anjos, G. S. D. and Fernandes, H. D. S. and Mizubuti, G. B. and Vieira, J. E. and da Silva, L. M. |
| 367 | Wrong outcome | Deep learning for deterioration prediction of COVID-19 patients based on time-series of three vital signs | 2023 | Mehrdad, S. and Shamout, F. E. and Wang, Y. and Atashzar, S. F. |
| 368 | Wrong outcome | [Deep residual convolutional neural network for recognition of electrocardiogram signal arrhythmias] | 2019 | Li, D. and Zhang, H. and Liu, Z. and Huang, J. and Wang, T. |
| 369 | Wrong outcome | Defining patient deterioration through acute care and intensive care nurses' perspectives | 2016 | Lavoie, Patrick and Pepin, Jacinthe and Alderson, Marie |
| 370 | Wrong outcome | Defining the hemodynamic response of hypertensive and normotensive subjects through serial timed blood pressure readings in the clinic | 2019 | Shahab, H. and Khan, H. S. and Almas, A. and Khan, S. A. and Artani, A. and Khan, A. H. |
| 371 | Wrong outcome | Dehydration and death during febrile episodes in the nursing home | 1994 | Weinberg, A. D. and Pals, J. K. and Levesque, P. G. and Beal, L. F. and Cunningham, T. J. and Minaker, K. L. |
| 372 | Wrong outcome | Delayed Recognition of Deterioration of Patients in General Wards Is Mostly Caused by Human Related Monitoring Failures: A Root Cause Analysis of Unplanned ICU Admissions | 2016 | van Galen, L. S. and Struik, P. W. and Driesen, B. E. and Merten, H. and Ludikhuize, J. and van der Spoel, J. I. and Kramer, M. H. and Nanayakkara, P. W. |
| 373 | Wrong outcome | Delayed Sudden Respiratory Arrest After a High-energy Motorcycle Accident | 2019 | Ohsaka, H. and Jitsuiki, K. and Yanagawa, Y. |
| 374 | Wrong outcome | Depressive Symptoms and Missed Nursing Care among Clinical Nurses: A Cross-Sectional Survey | 2023 | Yoo Mi, Jeong and Hyoung Eun, Chang |
| 375 | Wrong outcome | Descriptive study of ambulatory blood pressure monitoring in the Primary Care Nursing clinic | 2013 | GarzÃ³n-QuiÃ±ones, M. and Gallardo-Gonzalo, C. and PadÃ­n-Minaya, C. and LÃ³pez-Pisa, R. M. and RodrÃ­guez-Latre, L. M. |
| 376 | Wrong outcome | Descriptive study of nurses' compliance with postprocedural vital sign measurement in a gastrointestinal investigation unit | 2002 | Dempsey, D. and Conroy-Hiller, T. and O'Neill, S. and McCutcheon, H. |
| 377 | Wrong outcome | A descriptive survey of operating theatre and intensive care unit temperature management of burn patients in the united kingdom | 2021 | Mullhi, R. and Ewington, I. and Chipp, E. and Torlinski, T. |
| 378 | Wrong Study Design | Design and implementation of wireless patient monitoring system for postoperative patients on general care floor | 2012 | Choi, J. S. and Kim, D. |
| 379 | Wrong outcome | Design and manufacture of medical carbon fiber thermostatic heating pads | 2019 | Yan, P. and Mu, X. L. and Zheng, W. D. and Cui, J. F. and Liu, J. H. and Ma, Y. F. and Gao, X. and Hao, S. F. and Du, J. |
| 380 | Wrong outcome | Design, implementation, and impact of a cirrhosis-specific remote patient monitoring program | 2024 | Penrice, D. D. and Hara, K. S. and Sordi-Chara, B. and Kezer, C. and Schmidt, K. and Kassmeyer, B. and Lennon, R. and Rosedahl, J. and Roellinger, D. and Rattan, P. and Williams, K. and Kloft-Nelson, S. and Leuenberger, A. and Kamath, P. S. and Shah, V. H. and Simonetto, D. A. |
| 381 | Wrong outcome | Designing and Implementation of a Heart Failure Telemonitoring System | 2017 | Safdari, R. and Jafarpour, M. and Mokhtaran, M. and Naderi, N. |
| 382 | Wrong outcome | Detecting early signs of deterioration and preventing hospitalizations in skilled nursing facilities using remote respiratory monitoring | 2024 | Lauteslager, T. and Dishakjian, V. and Watson, L. and Savarese, J. and Williams, A. J. and Leschziner, G. D. |
| 383 | Wrong outcome | Detection and management of the deteriorating ward patient: an evaluation of nursing practice | 2015 | Odell, M. |
| 384 | Wrong outcome | Detection of Fever Using Continuous Skin Temperature Monitoring in Hospitalized Older Adults | 2023 | Yi-Jhen, Chen and Yi-Ting, Chung and Chang-Chun, Chen and Yen-Chin, Chen and Shih-Hsin, Liang and Jiun-Ling, Wang and Chun-Yin, Y. E. H. and Kun-Ta, Chuang and Nai-Ying, K. O. |
| 385 | Wrong outcome | [Detection of Fever Using Continuous Skin Temperature Monitoring in Hospitalized Older Adults] | 2023 | Chen, Y. J. and Chung, Y. T. and Chen, C. C. and Chen, Y. C. and Liang, S. H. and Wang, J. L. and Yeh, C. Y. and Chuang, K. T. and Ko, N. Y. |
| 386 | Wrong outcome | Deterioration in hospital patients: early signs and appropriate actions | 2008 | Beaumont, K. and Luettel, D. and Thomson, R. |
| 387 | Wrong outcome | Deterioration to decision: a comprehensive literature review of rapid response applications for deteriorating patients in acute care settings | 2020 | Baig, M. M. and Afifi, S. and GholamHosseini, H. and Ullah, E. |
| 388 | Wrong outcome | Determinants of Self-Care and Home-Based Management of Hypertension: An Integrative Review | 2023 | Konlan, K. D. and Shin, J. |
| 389 | Wrong outcome | Determination knowledge of nursesâ€™ concerning measurement of blood pressure in emergency department at al-hussein teaching hospital in al-nasiriya city | 2020 | Naser, A. M. and Abed, Q. J. O. |
| 390 | Wrong outcome | Determination of information requirements of mothers with regard to taking care of their premature babies who have hospitalised in the neonatal intensive care unit | 2021 | Kabasakal, A. and Vural, G. |
| 391 | Wrong outcome | Determination of platinum in workroom air and in blood and urine from nursing staff attending patients receiving cisplatin chemotherapy | 1997 | Nygren, O. and Lundgren, C. |
| 392 | Wrong outcome | Determination of the Frequency of Clinical Skills Implementation by Senior Nursing Students in an Emergency Department | 2014 | Cinar, Fatma Ilknur and Unver, Vesile and Seven, Memnun and Fidanci, Berna Eren and Cicek, Hatice Sutcu and Yava, Ayla |
| 393 | Wrong outcome | Determining the Awareness of Nurses Regarding the Basics of Blood Pressure Control | 2012 | Mohammad, Heidari and Sara, Shahbazi and Fatemeh, Aliakbari |
| 394 | Wrong outcome | Determining the Effectiveness of Forced-Air Warming Blankets in Maintaining Postoperative Body Temperature: A Randomized Controlled Trial | 2022 | IÅŸÄ±klÄ±, A. G. and FÄ±ndÄ±k Ãœ, Y. |
| 395 | Wrong outcome | DEveloping a Complex Intervention for DEteriorating patients using theoretical modelling (DECIDE study): Study protocol | 2019 | Smith, D. and Francis, J. J. and Aitken, L. M. |
| 396 | Wrong outcome | Developing a core outcome set for patient-reported symptom monitoring to reduce hospital admissions for patients with heart failure | 2022 | Lawson, C. A. and Lam, C. and Jaarsma, T. and Kadam, U. and Stromberg, A. and Ali, M. and Tay, W. T. and Clayton, L. and Khunti, K. and Squire, I. |
| 397 | Wrong outcome | Developing a sociocultural framework of compliance: an exploration of factors related to the use of early warning systems among acute care clinicians | 2020 | Flenady, T. and Dwyer, T. and Sobolewska, A. and Lagadec, D. L. and Connor, J. and Kahl, J. and Signal, T. and Browne, M. |
| 398 | Wrong outcome | Developing a Virtual Nursing Team to Support Predictive Analytics and Gaps in Patient Care | 2020 | Lisk, Laure E. and Buckley, Jacqueline D. and Wilson, Kristine and Martinez, Vanessa A. and Cadiz, Vilma R. and Poropat, Lorelle and Scruth, Elizabeth Ann |
| 399 | Wrong outcome | Developing a Vital Sign Alert System | 2013 | Jones, Barbara G. |
| 400 | Wrong Study Design | Developing a wireless sensor network based on a proposed algorithm for healthcare purposes | 2020 | Abbasi-Kesbi, R. and Asadi, Z. and Nikfarjam, A. |
| 401 | Wrong outcome | Developing and using a dengue patient care guideline for patients admitted from households to primary care units and the district hospital: A community participatory approach in Southern Thailand | 2020 | Suwanbamrung, C. and Le, C. N. and Maneerattanasak, S. and Satian, P. and Talunkphet, C. and Nuprasert, Y. and Siwarin, A. and Kotchawat, S. and Srimoung, P. and Ponprasert, C. and Nontapet, O. |
| 402 | Wrong outcome | Development and clinical empirical validation of the chronic critical illness prognosis prediction model | 2024 | Zhao, L. and Xu, W. K. and Wang, Y. and Lu, W. Y. and Wu, Y. and Hu, R. |
| 403 | Wrong outcome | Development and Comparative Performance of Physiologic Monitoring Strategies in the Emergency Department | 2022 | Kim, D. and Jin, B. T. |
| 404 | Wrong outcome | Development and implementation of a nurse-based remote patient monitoring program for ambulatory disease management | 2022 | Coffey, J. D. and Christopherson, L. A. and Williams, R. D. and Gathje, S. R. and Bell, S. J. and Pahl, D. F. and Manka, L. and Blegen, R. N. and Maniaci, M. J. and Ommen, S. R. and Haddad, T. C. |
| 405 | Wrong outcome | Development and Psychometric Properties of a Scale Measuring Barriers to Perioperative Hypothermia Prevention for Anesthesiologists and Nurses | 2023 | Huang, J. and Qi, H. and Lv, K. and Zhu, Y. and Wang, Y. and Jin, L. |
| 406 | Wrong outcome | Development and Validation of a Machine Learning Algorithm Using Clinical Pages to Predict Imminent Clinical Deterioration | 2024 | Steitz, B. D. and McCoy, A. B. and Reese, T. J. and Liu, S. and Weavind, L. and Shipley, K. and Russo, E. and Wright, A. |
| 407 | Wrong outcome | Development and validation of a methodology to measure the time taken by hospital nurses to make vital signs observations | 2020 | Dall'Ora, C. and Hope, J. and Bridges, J. and Griffiths, P. |
| 408 | Wrong outcome | Development and validation of an instrument for measuring junior nurses' recognition and response abilities to clinical deterioration (RRCD) | 2023 | Xu, Laiyu and Tan, Jianwen and Chen, Qirong and Luo, Zhen and Song, Lili and Liu, Qingqing and Peng, Lingli |
| 409 | Wrong outcome | Development and validation of an instrument measuring deterioration in social and spiritual aspects among elderly patients in Indonesia hospitals | 2019 | Silaswati, Shintha and Sahar, Junaiti and Sari, Nina Kemala and Bardosono, Saptawati and Umar, Jahja |
| 410 | Wrong outcome | Development and Validation of Early Warning Criteria to Identify Escalated Care Events in Neonatal Intensive Care Unit Patients | 2020 | Shivananda, S. and Twiss, J. and Paterson, D. and Dyck, G. and Becker, S. and Razack, A. and Gupta, S. and Dutta, S. and Suresh, G. |
| 411 | Wrong outcome | Development of a Decision Tree Analysis model that predicts recovery from acute brain injury | 2013 | Oh, Hyun Soo and Seo, Wha Sook |
| 412 | Wrong outcome | Development of a diabetic retinopathy screening model for a district health system in Limpopo Province, South Africa | 2022 | Abdool, Z. and Naidoo, K. and Visser, L. |
| 413 | Wrong outcome | Development of a neonatal incubator with phototherapy, biometric fingerprint reader, remote monitoring, and heart rate control adapted for developing countries hospitals | 2019 | Kapen, Pascalin Tiam and Mohamadou, Youssoufa and Momo, Foutse and Jauspin, Dongmeza Koudjou and Kanmagne, Nenkam and Jordan, Dongmeza Dongmo |
| 414 | Wrong outcome | Development of a practicable non-contact bedside autonomic activation monitoring system using microwave radars and its clinical application in elderly people | 2013 | Matsui, Takemi and Yoshida, Yuto and Kagawa, Masayuki and Kubota, Masayuki and Kurita, Akira |
| 415 | Wrong outcome | Development of an Acute Pain Assessment Tool for Patients in Post-anesthesia Care Units...American Society for Pain Management Nursing (ASPMN) 33rd National Conference, September 20-23, 2023, Minneapolis, Minnesota | 2024 | Kim, Kyoungsook and Choi, Suna and Park, Myouyun and Kim, Junghee and Kim, Kwanghee and Song, Eunjin and Kim, Nagyeong and Park, Seyeon and Song, Youngshin |
| 416 | Wrong outcome | Development of an Early Warning System to Prevent Crises in the Palliative Home Care Setting of Patients and Their Informal Caregivers: Protocol for a Mixed Method Study | 2019 | Fringer, A. and Arrer, E. and Maier, E. and Schnepp, W. and Ulmer, T. |
| 417 | Wrong outcome | Development of an enhanced scoring system to predict ICU readmission or in-hospital death within 24 hours using routine patient data from two NHS Foundation Trusts | 2024 | Pimentel, M. A. F. and Johnson, A. and Darbyshire, J. L. and Tarassenko, L. and Clifton, D. A. and Walden, A. and Rechner, I. and Watkinson, P. J. and Young, J. D. |
| 418 | Wrong outcome | Development of early warning and rapid response system for patients with novel coronavirus pneumonia (COVID-19): A research protocol | 2020 | Zhou, H. and Huang, H. and Xie, X. and Gao, J. and Wu, J. and Zhu, Y. and He, W. and Liu, J. and Li, A. and Xu, Y. |
| 419 | Wrong outcome | Development of early warning and rapid response system for patients with novel coronavirus pneumonia (COVID-19): A research protocol | 2020 | Hua, Zhou and Huibin, Huang and Xiaolei, Xie and Jiandong, Gao and Ji, Wu and Yan, Zhu and Wei, H. and Jingyuan, Liu and Ang, Li and Yuan, Xu and Zhou, Hua and Huang, Huibin and Xie, Xiaolei and Gao, Jiandong and Wu, Ji and Zhu, Yan and He, Wei and Liu, Jingyuan and Li, Ang and Xu, Yuan |
| 420 | Wrong outcome | Development of Heart and Respiratory Rate Percentile Curves for Hospitalized Children | 2013 | Bonafide, Christopher P. and Brady, Patrick W. and Keren, Ron and Conway, Patrick H. and Marsolo, Keith and Daymon, Carrie |
| 421 | Wrong outcome | Development of novel optical character recognition system to reduce recording time for vital signs and prescriptions: A simulation-based study | 2024 | Soeno, S. and Liu, K. and Watanabe, S. and Sonoo, T. and Goto, T. |
| 422 | Wrong outcome | The development of nursing intervention based on indonesian nursing intervention standard on infarction stroke in hospital | 2019 | Ariyanti, H. R. and Nursalam, N. and Yuwono, S. R. |
| 423 | Wrong outcome | Developmental patterns of physiological response to a multisensory intervention in extremely premature and high-risk infants | 2004 | White-Traut, R. C. and Nelson, M. N. and Silverstri, J. M. and Patel, M. and Berbaum, M. and Gu, G. and Rey, P. M. |
| 424 | Wrong outcome | Dexmedetomidine versus propofol for operator-directed nurse-administered procedural sedation during catheter ablation of atrial fibrillation: A randomized controlled study | 2022 | Servatius, H. and KÃ¼ffer, T. and Baldinger, S. H. and Asatryan, B. and Seiler, J. and Tanner, H. and Novak, J. and Lam, A. and Noti, F. and Haeberlin, A. and Madaffari, A. and Sweda, R. and MÃ¼hl, A. and Branca, M. and DÃ¼tschler, S. and Erdoes, G. and StÃ¼ber, F. and Theiler, L. and Reichlin, T. and Roten, L. |
| 425 | Wrong outcome | Diabetes Alliance in the Hunter and New England region | 2018 | Parsons, Martha and Luu, Judy and Acharya, Shamasunder and Philcox, Annalise |
| 426 | Wrong outcome | Diagnosing and remediating harmful data shifts for the responsible deployment of clinical AI models | 2023 | Subasri, V. and Krishnan, A. and Dhalla, A. and Pandya, D. and Malkin, D. and Razak, F. and Verma, A. A. and Goldenberg, A. and Dolatabadi, E. |
| 427 | Wrong outcome | Diagnostic accuracy of clinical outcome prediction using nursing data in intensive care patients: A systematic review | 2023 | Kim, Mihui and Park, Sangwoo and Kim, Changhwan and Choi, Mona |
| 428 | Wrong outcome | Diagnostic power and healthcare resource consumption of a dedicated workflow algorithm designed to manage thoracic impedance alerts in heart failure patients by remote monitoring | 2018 | Ricci, R. P. and Morichelli, L. and Porfili, A. and Quarta, L. and Sassi, A. |
| 429 | Wrong outcome | Differences between patients with asymptomatic and symptomatic myocardial infarction: the relevance of psychological factors | 1994 | Myrtek, M. and Fichtler, A. and KÃ¶nig, K. and BrÃ¼gner, G. and MÃ¼ller, W. |
| 430 | Wrong outcome | Differences in symptoms during and post PTCA versus rotational ablation | 1994 | Murphy, M. C. and Hansell, H. N. and Ward, K. and Shaw, R. E. |
| 431 | Wrong outcome | Different fate of herpes simplex encephalitis (Clinical and eeg cases report) | 2020 | DrobnÃ½, M. and KrkoÅ¡ka, D. and SobolovÃ¡, G. and BabuÅ¡Ã­k, M. and UÄÅˆovÃ¡, S. and SÃ¡niovÃ¡, B. D. and TulejovÃ¡, L. and Priadka, D. |
| 432 | Wrong outcome | Difficult hospital discharges in internal medicine wards | 2007 | Nardi, R. and Scanelli, G. and Tragnone, A. and Lolli, A. and Kalfus, P. and Baldini, A. and Ghedini, T. and Bombarda, S. and Fiadino, L. and Di Ciommo, S. |
| 433 | Wrong outcome | Difficult Hypertension Clinic Utilizing a Nurse Specialist: A Cost-Efficient Model for the Modern Era? | 2015 | van der Merwe, W. and van der Merwe, V. |
| 434 | Wrong outcome | Difficult-to-control hypertension: identification of clinical predictors and use of ICT-based integrated care to facilitate blood pressure control | 2018 | Visco, V. and Finelli, R. and Pascale, A. V. and Mazzeo, P. and Ragosa, N. and Trimarco, V. and Illario, M. and Ciccarelli, M. and Iaccarino, G. |
| 435 | Wrong outcome | Diminishing surgical site infections after colorectal surgery with surgical care improvement project: Is it time to move on? | 2011 | Larochelle, M. and Hyman, N. and Gruppi, L. and Osler, T. |
| 436 | Wrong population | A Direct Assessment of Noninvasive Continuous Blood Pressure Monitoring in the Emergency Department and Intensive Care Unit | 2024 | Hamilton, Landon D. and Binns, Scott and McFann, Kim and Nudell, Nikiah and Dunn, Julie A. |
| 437 | Wrong outcome | Discussion on a new model of holistic treatment for chronic critical illness patients by internal cross-disciplinary team in the department of intensive care unit: clinical data analysis of a case of acute exacerbation of chronic obstructive pulmonary disease | 2022 | Chen, L. and Zheng, C. and Hong, X. and Chen, Y. and Sun, X. and Liu, Y. |
| 438 | Wrong outcome | Distribution of Lifestyle Risk Factors and Biophysical/Biochemical Parameters Related to NCD Prevention Among Nurses in Selected Government Hospitals of Chandigarh, India (2020â€“2022): A Multicentered Cross-sectional Study | 2023 | Kaur, S. and Dhandapani, M. and Dhaliwal, N. and Kaur, J. and Singh, M. and Kathwal, Jyoti |
| 439 | Wrong outcome | Distribution of the National Early Warning Score (NEWS) in care home residents | 2020 | Barker, Robert Oliver and Stocker, Rachel and Russell, SiÃ¢n and Roberts, Anthony and Kingston, Andrew and Adamson, Joy and Hanratty, Barbara |
| 440 | Wrong outcome | Diurnal variation in the performance of rapid response systems: the role of critical care services-a review article | 2016 | Sundararajan, K. and Flabouris, A. and Thompson, C. |
| 441 | Wrong outcome | Divergent views of hospital staff on detecting and managing hypertension | 1979 | Taylor, L. and Foster, M. C. and Beevers, D. G. |
| 442 | Wrong outcome | Do anaesthetized patients recover better after Bispectral Index monitoring? | 2001 | Burrow, B. and McKenzie, B. and Case, C. |
| 443 | Wrong outcome | Do Not Disturb: Vital Sign Monitoring as a Predictor of Clinical Deterioration in Monitored Patients | 2017 | Newman, Susan |
| 444 | Wrong population | Do we know how to take the arterial pressure in the pediatric patient? Knowledge of infirmary health care personnel about determination of arterial pressure in the pediatric patient | 2008 | EmÃ©rita Gabriela, L. V. and Vera, J. |
| 445 | Wrong outcome | Do We Need a Systematic Activation of Alarm Soundings for Blood Pressure Monitoring for the Safety of ICU Patients? | 2003 | Biot, L. and Holzapfel, L. and Becq, G. and MÃ©lot, C. and Baconnier, P. |
| 446 | Wrong outcome | Documenting pain as the fifth vital sign: a feasibility study in an oncology ward in Sarawak, Malaysia | 2008 | Devi, B. C. and Tang, T. S. |
| 447 | Wrong outcome | Does Accidental Hypothermia Increase Morbidity and Mortality in Mature Neonates? | 2024 | Petersen, L. and Kainer, F. and Schroth, M. A. |
| 448 | Wrong outcome | Does case management improve physiologic outcomes for patients with poorly controlled diabetes? | 2004 | Persell, S. D. and Murff, H. J. and Spigel, D. R. and Jha, A. K. and Horng, M. S. |
| 449 | Wrong outcome | Does Telehealth Monitoring Identify Exacerbations of Chronic Obstructive Pulmonary Disease and Reduce Hospitalisations? An Analysis of System Data | 2017 | Kargiannakis, M. and Fitzsimmons, D. A. and Bentley, C. L. and Mountain, G. A. |
| 450 | Wrong outcome | Does this patient need telemetry? An analysis of telemetry ordering practices at an academic medical center | 2017 | Chen, Stephanie and Palchaudhuri, Sonali and Johnson, Amber and Trost, Jeff and Ponor, Ileana and Zakaria, Sammy |
| 451 | Wrong outcome | Dorsal penile nerve block vs topical placebo for circumcision in low- birth-weight neonates | 1999 | Holliday, M. A. and Pinckert, T. L. and Kiernan, S. C. and Kunos, I. and Angelus, P. and Keszler, M. |
| 452 | Wrong outcome | [Drawing up guidelines for the attendance of physical health of patients with severe mental illness] | 2009 | Saravane, D. and Feve, B. and Frances, Y. and Corruble, E. and Lancon, C. and Chanson, P. and Maison, P. and Terra, J. L. and Azorin, J. M. |
| 453 | Wrong outcome | Dressings and Securement Devices of Peripheral Arterial Catheters in Intensive Care Units and Operating Theaters: A Systematic Review | 2020 | Gravante, F. and Lombardi, A. and Gagliardi, A. M. and Pucci, A. and Latina, R. |
| 454 | Wrong outcome | Driving environment in Iran increases blood pressure even in healthy taxi drivers | 2008 | Navadeh, S. and Moazenzadeh, M. and Mirzazadeh, A. |
| 455 | Wrong outcome | Dynamic composition of medical support services in the ICU: Platform and algorithm design details | 2010 | Hristoskova, A. and Moeyersoon, D. and Van Hoecke, S. and Verstichel, S. and Decruyenaere, J. and De Turck, F. |
| 456 | Wrong outcome | A dynamic multi-attribute utility theory-based decision support system for patient prioritization in the emergency department | 2014 | Claudio, D. and Kremer, G. E. O. and Bravo-Llerena, W. and Freivalds, A. |
| 457 | Wrong outcome | Dynamic vitals monitoring for patient prioritization in the emergency department: A technology enabled utility approach | 2010 | Claudio, David |
| 458 | Wrong outcome | Early changes in skin surface temperature predict body temperature increases in patients with fever: A pilot study | 2024 | Chung, Yi-Ting and Yeh, Chun-Yin and Chen, Chang-Chun and Lai, Chao-Han and Lin, Yi-Hsuan and Lin, Chung-Ying and Shu, Yu-Chen and Ko, Nai-Ying |
| 459 | Wrong outcome | Early diagnosis of sepsis using an E-health application for a clinical early warning system outside of theÂ intensive care unit: a case report | 2022 | Ghazali, D. A. and Kenway, P. and Choquet, C. and Casalino, E. |
| 460 | Wrong outcome | Early Hospital Discharge Using Remote Monitoring for Patients Hospitalized for COVID-19, Regardless of Need for Home Oxygen Therapy: A Descriptive Study | 2023 | Talha, S. and Lamrous, S. and Kassegne, L. and Lefebvre, N. and Zulfiqar, A. A. and Tran Ba Loc, P. and Geny, M. and Meyer, N. and Hajjam, M. and AndrÃ¨s, E. and Geny, B. |
| 461 | Wrong outcome | Early monitoring of intravenous thrombolysis in acute ischaemic stroke using wearable intelligent vital sign devices: protocol for a prospective, multicentre, observational registry cohort study | 2023 | Liu, M. and Zhao, J. and Li, S. and Han, J. and Ma, G. and Wang, Y. and Chang, H. |
| 462 | Wrong outcome | Early prediction of delirium upon intensive care unit admission: Model development, validation, and deployment | 2023 | Wang, M. L. and Kuo, Y. T. and Kuo, L. C. and Liang, H. P. and Cheng, Y. W. and Yeh, Y. C. and Tsai, M. T. and Chan, W. S. and Chiu, C. T. and Chao, A. and Chou, N. K. and Yeh, Y. C. and Ku, S. C. |
| 463 | Wrong outcome | Early rehabilitation nursing in ICU promotes rehabilitation of patients with respiratory failure treated with invasive mechanical ventilation | 2021 | Jin, Y. and Di, J. and Wang, X. |
| 464 | Wrong outcome | Early versusÂ late COVID-19 Home Health Care patient population: Shifting sociodemographics and comparable outcomes | 2022 | Videon, T. M. and Rosati, R. J. and Landers, S. H. |
| 465 | Wrong outcome | Early warning score challenges and opportunities in the care of deteriorating patientsâ€© | 2018 | Petersen, J. A. |
| 466 | Wrong outcome | [Early Warning Scores at the nursing ward: what do you really want to know?] | 2020 | Bredie, S. J. H. and van Goor, H. |
| 467 | Wrong outcome | Early Warning Scores to Support Continuous Wireless Vital Sign Monitoring for Complication Prediction in Patients on Surgical Wards: Retrospective Observational Study | 2023 | van Rossum, M. C. and Bekhuis, R. E. M. and Wang, Y. and Hegeman, J. H. and Folbert, E. C. and Vollenbroek-Hutten, M. M. R. and Kalkman, C. J. and Kouwenhoven, E. A. and Hermens, H. J. |
| 468 | Wrong outcome | Early warning tools to identify children at risk of deterioration: a discussion | 2004 | Tume, L. and Bullock, I. |
| 469 | Wrong outcome | Early warning- and track and trigger systems for newborn infants: A review | 2017 | Mortensen, Nicolay and Augustsson, Johan Henrik and Ulriksen, Jorunn and Hinna, Unni Tveit and SchmÃ¶izer, Georg M. and SolevagÃ¥, Anne Lee |
| 470 | Wrong outcome | ED "hold" patients: is their care also being held? | 2000 | Sobie, J. M. and Gaves, D. and Tringali, A. |
| 471 | Wrong outcome | Educational and organisational interventions used to improve the management of hypertension in primary care: A systematic review | 2005 | Fahey, T. and Schroeder, K. and Ebrahim, S. |
| 472 | Wrong outcome | An educational implementation of a cancer pain algorithm for ambulatory care | 2000 | Du Pen, A. R. and Du Pen, S. and Hansberry, J. and Miller-Kraybill, B. and Millen, J. and Everly, R. and Hansen, N. and Syrjala, K. |
| 473 | Wrong outcome | Effect and Significance of High-Quality Nursing on Blood Glucose, Pregnancy Outcome, and Neonatal Complications of Patients with Gestational Diabetes Mellitus | 2022 | Zhong, W. and Li, C. and Liu, J. and Zhou, J. and Xiao, Z. and Li, C. and Wu, H. |
| 474 | Wrong outcome | The effect of "nurse Companionship" on anxiety and vital sign changes of cesarean section candidates: A randomized control trial | 2020 | Roshangar, F. and Lotfi, M. and Aghazadeh, A. M. and Asghari, E. and Aghaei, E. |
| 475 | Wrong outcome | Effect of 1â€‰+â€‰N Extended Nursing Service on Functional Recovery of Colostomy Patients | 2022 | Feng, C. and Lv, C. and Zhang, X. and Guo, Y. and Li, X. |
| 476 | Wrong outcome | Effect of a comprehensive geriatric assessment nursing intervention model on older patients with diabetes and hypertension | 2024 | Bao, D. Y. and Wu, L. Y. and Cheng, Q. Y. |
| 477 | Wrong outcome | Effect of a Coordinated Community and Chronic Care Model Team Intervention vs Usual Care on Systolic Blood Pressure in Patients With Stroke or Transient Ischemic Attack: The SUCCEED Randomized Clinical Trial | 2021 | Towfighi, A. and Cheng, E. M. and Ayala-Rivera, M. and Barry, F. and McCreath, H. and Ganz, D. A. and Lee, M. L. and Sanossian, N. and Mehta, B. and Dutta, T. and Razmara, A. and Bryg, R. and Song, S. S. and Willis, P. and Wu, S. and Ramirez, M. and Richards, A. and Jackson, N. and Wacksman, J. and Mittman, B. and Tran, J. and Johnson, R. R. and Ediss, C. and Sivers-Teixeira, T. and Shaby, B. and Montoya, A. L. and Corrales, M. and Mojarro-Huang, E. and Castro, M. and Gomez, P. and MuÃ±oz, C. and Garcia, D. and Moreno, L. and Fernandez, M. and Lopez, E. and Valdez, S. and Haber, H. R. and Hill, V. A. and Rao, N. M. and Martinez, B. and Hudson, L. and Valle, N. P. and Vickrey, B. G. |
| 478 | Wrong population | The Effect of a Light-Dark Cycle on Premature Infants in the Neonatal Intensive Care Unit: A Randomized Controlled Study | 2024 | Olgun, A. B. and YÃ¼ksel, D. and YardÄ±mcÄ±, F. |
| 479 | Wrong outcome | The effect of a managerial-based intervention on the occurrence of out-of-range-measurements and mortality in intensive care units | 2004 | Fidler, V. and Nap, R. and Miranda, D. R. |
| 480 | Wrong outcome | The Effect of a Nurse-Driven Program Utilizing Implantable Pulmonary Artery Pressure Monitoring to Reduce Hospitalizations in Low-Socioeconomic Urban Patients with Heart Failure | 2020 | Alcain, Charina and Aziz-Smith, Rahmana and Besser, Stephanie and Chua, Rhys and Spencer, Kirk and Tabit, Corey |
| 481 | Wrong outcome | The effect of a randomized trial of home telemonitoring on medical costs, 30-day readmissions, mortality, and health-related quality of life in a cohort of community-dwelling heart failure patients | 2014 | Blum, K. and Gottlieb, S. S. |
| 482 | Wrong outcome | Effect of a staffing strategy based on voluntary increase in working hours on quality of patient care in a hospital in KwaZulu-Natal | 2009 | McIntosh, J. and Stellenberg, E. L. |
| 483 | Wrong outcome | The effect of a web-based educational program on nursing practice in recognising and responding to deteriorating ward patients: A qualitative evaluation study | 2017 | Liaw, Sok Ying and Ping Lim, Eunice Ya and Wong, Lai Fun and Yin Ho, Jasmine Tze and Mordiffi, Siti Zubaidah and Leng Ang, Sophia Bee and Chua, Wei Ling and Ang, Emily Neo Kim |
| 484 | Wrong outcome | Effect of a Web-Based Management Guide on Risk Factors in Patients with Type 2 Diabetes and Diabetic Kidney Disease: A JADE Randomized Clinical Trial | 2022 | Chan, J. C. N. and Thewjitcharoen, Y. and Nguyen, T. K. and Tan, A. and Chia, Y. C. and Hwu, C. M. and Jian, D. and Himathongkam, T. and Wong, K. L. and Choi, Y. M. and Mirasol, R. and Mohamed, M. and Kong, A. P. S. and Ma, R. C. W. and Chow, E. Y. K. and Ozaki, R. and Lau, V. and Fu, A. W. C. and Hong, E. G. and Yoon, K. H. and Tsang, C. C. and Lau, E. S. H. and Lim, L. L. and Luk, A. O. Y. |
| 485 | Wrong outcome | Effect of a Wireless Vital Sign Monitoring System on the Rapid Response System in the General Ward | 2022 | Han, W. H. and Sohn, D. K. and Hwangbo, Y. and Park, H. J. and Kim, M. and Choi, Y. and Shin, I. W. and Lee, J. M. and Jeon, H. and Ryu, K. C. and Yoon, T. and Kim, J. H. |
| 486 | Wrong outcome | The effect of audio-visual video with korotkoff sounds on anxiety levels and blood pressure measurement skills of nursing students: A randomized controlled study | 2023 | Ãœlker, T. and Korkut, S. |
| 487 | Wrong population | The Effect of Birthing Ball Exercises on Labor Pain and Labor Outcome Among Primigraviade Parturient Mothers at a Tertiary Care Hospital | 2023 | Jha, S. and Vyas, H. and Nebhinani, M. and Singh, P. and T, D. |
| 488 | Wrong outcome | Effect of bundle set interventions on physiologic alarms and alarm fatigue in an intensive care unit: A quality improvement project | 2021 | Seifert, Micah and Tola, Denise H. and Thompson, Julie and McGugan, Lynn and Smallheer, Benjamin |
| 489 | Wrong outcome | Effect of cardiovascular biofeedback on nursing staff stress: a randomized controlled clinical trial | 2023 | Macedo, A. B. T. and Vega, E. A. U. and Antoniolli, L. and Pinheiro, J. M. G. and Tavares, J. P. and Souza, S. B. C. |
| 490 | Wrong outcome | The effect of community-based health management on the health of the elderly: a randomized controlled trial from China | 2012 | Chao, Jianqian and Wang, Yimin and Xu, Hui and Yu, Qing and Jiang, Lili and Tian, Lin and Xie, Wenyuan and Liu, Pei |
| 491 | Wrong outcome | Effect of Continuous Infusion vs Bolus Dose of Hydrocortisone in Septic Shock: A Prospective Randomized Study | 2024 | Salhotra, R. and Sharahudeen, A. and Tyagi, A. and Rautela, R. S. and Kemprai, R. |
| 492 | Wrong outcome | The effect of different times of day for exercise on blood glucose fluctuations | 2024 | Niu, W. C. and Liu, C. and Liu, K. and Fang, W. J. and Liu, X. Q. and Liang, X. L. and Yuan, H. P. and Jia, H. M. and Peng, H. F. and Jiang, H. W. and Jia, Z. M. |
| 493 | Wrong outcome | Effect of early progressive mobilization for stroke patients with mechanical ventilation in the neurointensive care unit | 2022 | Xiaolong, Y. and Lei, C. and Xin, Q. and Wenjin, C. and Na, W. and Weiqun, S. |
| 494 | Wrong outcome | Effect of education based on the Common-Sense Model of Self-Regulation on blood pressure and self-management of hypertensive patients: A clinical trial study | 2023 | Kordvarkane, Z. and Oshvandi, K. and Mohammadi, Y. and Azizi, A. |
| 495 | Wrong outcome | The effect of emergency nursing on the mental health and limb function recovery of myocardial infarction patients | 2021 | Wang, Y. and Tang, Z. and Dong, A. and Cai, J. and Le, P. |
| 496 | Wrong outcome | The effect of event recording home infant apnea/heart rate monitoring in the greater Los Angeles area | 1991 | Schlose, T. and Den Blyker, R. P. and McHattie, J. and Van Zitter, P. and Martin, K. and King, K. |
| 497 | Wrong outcome | Effect of facilitated tucking with the nurse and a simulated hand on physiological pain index during vein puncture on premature infants | 2018 | Salmani, N. and Karjoo, Z. and Dehghani, K. and Sadeghnia, A. |
| 498 | Wrong outcome | The Effect of Foot Bath on Physiological Parameters and Anxiety in Patients With Acute Stroke: A Randomized Controlled Trial | 2023 | Seidi, Jamal and Gheshlagh, Reza Ghanei and Nourifard, Yaser and Dehvan, Fazel |
| 499 | Wrong outcome | Effect of high-quality nursing on postoperative recovery, adverse reactions and degree of pain of patients undergoing abdominal surgery under general anesthesia | 2020 | Liu, S. and Xia, Y. and Shen, J. |
| 500 | Wrong outcome | Effect of hospitalization on conventional and 24-hour blood pressure | 1995 | Fotherby, M. D. and Critchley, D. and Potter, J. F. |
| 501 | Wrong outcome | Effect of hygiene interventions on the thermal stability of extremely low-birth-weight newborns in the first two weeks of life | 2005 | Montes Bueno, T. and De La Fuente Calle, P. and Iglesias Diz, A. and Bescos Calvo, C. and QuÃ­lez Cervera, P. and Madero Jarabo, R. and GarcÃ­a-Alix PÃ©rez, A. and Quero JimÃ©nez, J. |
| 502 | Wrong outcome | Effect of hypertension on the long-term prognosis of children with primary focal segmental glomerulosclerosis-a retrospective cohort study | 2023 | Huang, L. and Peng, W. |
| 503 | Wrong outcome | Effect of Implementing a Commercial Electronic Early Warning System on Outcomes of Hospitalized Patients | 2023 | Singh, S. and Laud, P. W. and Crotty, B. H. and Nanchal, R. S. and Hanson, R. and Penlesky, A. C. and Fletcher, K. E. and Stadler, M. E. and Dong, Y. and Nattinger, A. B. |
| 504 | Wrong outcome | Effect of implementing decision support to activate a rapid response system by automated screening of verified vital sign data: A retrospective database study | 2022 | Jerng, J. S. and Chen, L. C. and Chen, S. Y. and Kuo, L. C. and Tsan, C. Y. and Hsieh, P. Y. and Chen, C. M. and Chuang, P. Y. and Huang, H. F. and Huang, S. F. |
| 505 | Wrong outcome | Effect of innovative nursing practice on stress response of patients undergoing laparoscopic cholecystectomy | 2020 | Chen, G. X. and Lou, Y. J. |
| 506 | Wrong outcome | Effect of intraoperative temperature level on patient outcome in elderly patients with strangulated small bowel obstruction | 2023 | Wu, J. and Xu, L. and Li, X. |
| 507 | Wrong outcome | Effect of Intravenous Ketorolac on Postoperative Pain in Mandibular Fracture Surgery; A Randomized, Double-Blind, Placebo-Controlled Trial | 2017 | Eftekharian, H. R. and Ilkhani Pak, H. |
| 508 | Wrong outcome | Effect of intravenous ketorolac on postoperative pain in mandibular fracture surgery; A randomized, double-blind, placebo-controlled trial | 2017 | Eftekharian, H. R. and Pak, H. I. |
| 509 | Wrong outcome | Effect of local refrigeration prior to venipuncture on pain related responses in school age children | 2006 | Movahedi, A. F. and Rostami, S. and Salsali, M. and Keikhaee, B. and Moradi, A. |
| 510 | Wrong population | The Effect of Lullabies and Classical Music on Preterm Neonates' Cerebral Oxygenation, Vital Signs, and Comfort During Orogastric Tube Feeding: A Randomized Controlled Trial | 2024 | BaÄŸli, E. and KÃ¼Ã§Ã¼koÄŸlu, S. and Soylu, H. |
| 511 | Wrong outcome | Effect of massage therapy on preterm neonate's body temperature | 2021 | Nyaga, E. and Esamai, F. and Kyololo, O. |
| 512 | Wrong outcome | Effect of meditation on adrenaline, norepinephrine and blood pressure control of elderly hypertensive patients in community | 2021 | Shuwen, X. and Rong, L. and Jian, S. |
| 513 | Wrong outcome | The effect of melody on the physiological responses of heel sticks pain in neonates | 2015 | Marofi, M. and Nikobakht, F. and Badiee, Z. and Golchin, M. |
| 514 | Wrong outcome | Effect of motivational interviewing in hypertensive patients (MIdNIgHT): Study protocol for a randomized controlled trial | 2019 | Silveira, L. C. J. and Aliti, G. B. and Da Silva, E. M. and Pimentel, R. P. and Gus, M. and Rabelo-Silva, E. R. |
| 515 | Wrong outcome | Effect of night duties on blood pressure of healthy female nursing staff | 2021 | Chaitra, N. D. and Chaitra, M. S. and Veeraiah, S. |
| 516 | Wrong outcome | Effect of nurse-led home-based biofeedback intervention on the blood pressure levels among patients with hypertension: Pretest-posttest study | 2020 | Elavally, S. and Ramamurthy, M. T. and Subash, J. and Meleveedu, R. and Venkatasalu, M. R. |
| 517 | Wrong outcome | Effect of Nursing in Operating Room Combined with Intraoperative Heat Preservation Intervention on Prevention of Incision Infection and Improvement of Hemodynamics in Patients with Anterior Cruciate Ligament Injury and Reconstruction under Knee Arthroscopy | 2022 | Dai, J. and Li, Y. |
| 518 | Wrong outcome | The effect of personal space on blood pressure in the Turkish woman | 2018 | Cetinkaya-Uslusoy, E. and Tasci-Duran, E. |
| 519 | Wrong outcome | Effect of posture and positive end expiratory pressure on central venous pressure in patients with mechanical ventilation | 2007 | Wang, B. and Kang, Y. and Jin, X. D. and Qian, Z. C. and Dong, L. |
| 520 | Wrong outcome | The Effect of Precolonoscopy Lavender Inhalation on Patient Anxiety and Comfort: A Randomized, Controlled, Double-Blinded, Single-Center Study | 2023 | Sayilan, S. and Sayilan, A. A. and Mert, S. and Ã–ztekin, S. D. and Baydemir, C. |
| 521 | Wrong outcome | The effect of self-management support on knowledge level, treatment compliance and self-care management in patients with hypertension | 2022 | Kurt, Duygu |
| 522 | Wrong outcome | The effect of telehomecare on heart failure self care | 2010 | Bowles, K. H. and Riegel, B. and Weiner, M. G. and Glick, H. and Naylor, M. D. |
| 523 | Wrong outcome | The effect of the multimodal intervention on blood pressure in patients with first ischemic stroke: A randomized controlled trial | 2023 | Jullmusi, O. and Yunibhand, J. and Jitpanya, C. |
| 524 | Wrong outcome | The effect of thermoregulation quality improvement initiatives on the admission temperature of premature/very low birth-weight infants in neonatal intensive care units: A systematic review | 2020 | Donnellan, D. and Moore, Z. and Patton, D. and O'Connor, T. and Nugent, L. |
| 525 | Wrong outcome | The effect of transportation between the recovery room and intensive care unit on postoperative acoustic tumor patients | 1988 | Hamm, C. W. and Robertson, J. H. and Robertson, J. T. and Hathaway, D. K. and Tolley, E. |
| 526 | Wrong population | Effect of Two Bathing Methods on Physiologic Parameters in Pediatric Intensive Care | 2022 | Ã–z, Ã– and Uysal, G. and DÃ¼zkaya, D. S. |
| 527 | Wrong outcome | The Effect of Video Call with Family Members on Physiological Parameters of Critically Ill Patients in Intensive Care Unit: A Quasi-experimental Study | 2023 | Uysal, N. and VaizoÄŸlu, D. |
| 528 | Wrong outcome | Effect of voluntary breathing exercises on stable coronary artery disease in heart rate variability and rate-pressure product: a study protocol for a single-blind, prospective, randomized controlled trial | 2020 | Wu, Q. and Liu, L. and Jiang, X. and Hu, Y. Y. and Liang, Q. S. and He, Z. S. and Xue, Y. and Zhu, W. and Tang, Z. X. and Hou, Y. Y. and Zhao, Q. and Wang, X. H. |
| 529 | Wrong outcome | EFFECT OF WARM HUMIDIFIED OXYGEN ON THERMOREGULATION OF POST-OPERATIVE PATIENTS WITH ABDOMINAL SURGERY IN THE SELECTED HOSPITALS OF PUNE | 2023 | Karande, Jyoti Badade and Ganapathy, Meena |
| 530 | Wrong population | The effect of waterbirth on neonatal mortality and morbidity: a systematic review and meta-analysis | 2015 | Davies, R. and Davis, D. and Pearce, M. and Wong, N. |
| 531 | Wrong outcome | Effectiveness and cost-effectiveness of a virtual multidisciplinary stroke care clinic for community-dwelling stroke survivors and caregivers: a randomised controlled trial protocol | 2019 | Chau, J. P. C. and Lo, S. H. S. and Lee, V. W. Y. and Choi, K. C. and Shum, E. W. C. and Hung, Z. S. S. and Mok, V. C. T. and Siow, E. K. C. and Ching, J. Y. L. and Lam, S. K. Y. and Yeung, J. H. M. and Li, S. H. and Lau, A. Y. L. |
| 532 | Wrong outcome | Effectiveness and Practicality of eKTANG as a Digital Treatment for Diabetes and Relevant Influence Factors | 2022 | Lu, X. and Guo, D. and Feng, L. and Zhou, Y. and Zhang, C. and Li, J. and Jiang, Y. |
| 533 | Wrong outcome | The effectiveness of a thermal mattress in stabilizing and maintaining body temperature during the transport of very low-birth weight newborns | 2001 | L'Herault, J. and Petroff, L. and Jeffrey, J. |
| 534 | Wrong outcome | The effectiveness of a WeChat-based multimodal nursing program for women with breast cancer: A randomized controlled trial protocol | 2020 | Zhao, N. and Yin, F. and Wu, X. and Zhong, Y. |
| 535 | Wrong outcome | The effectiveness of a WeChat-based multimodal nursing program for women with breast cancer: A randomized controlled trial protocol | 2020 | Na, Zhao and Fang, Yin and Xiaofang, Wu and Yuxia, Zhong and Zhao, Na and Yin, Fang and Wu, Xiaofang and Zhong, Yuxia |
| 536 | Wrong outcome | Effectiveness of an Analytics-Based Intervention for Reducing Sleep Interruption in Hospitalized Patients: A Randomized Clinical Trial | 2022 | Najafi, N. and Robinson, A. and Pletcher, M. J. and Patel, S. |
| 537 | Wrong outcome | Effectiveness of application of a manual for improvement of alarms management by nurses in Intensive Care Units | 2021 | Yousefinya, Amirhossein and Torabizadeh, Camellia and Zand, Farid and Rakhshan, Mahnaz and Fararooei, Mohammad |
| 538 | Wrong outcome | Effectiveness of bed position versus chair position on reliability and validity of cardiac index in postoperative cardiothoracic surgery adult patients: A systematic review protocol | 2013 | Tartavoulle, T. and Manning, J. and Fowler, L. H. |
| 539 | Wrong outcome | Effectiveness of blood pressure educational and evaluation program for the improvement of measurement accuracy among nurses | 2013 | Rabbia, F. and Testa, E. and Rabbia, S. and PraticÃ², S. and Colasanto, C. and Montersino, F. and Berra, E. and Covella, M. and Fulcheri, C. and Di Monaco, S. and Buffolo, F. and Totaro, S. and Veglio, F. |
| 540 | Wrong outcome | Effectiveness of Goal-Setting Telephone Follow-Up on Health Behaviors of Patients with Ischemic Stroke: A Randomized Controlled Trial | 2016 | Wan, L. H. and Zhang, X. P. and Mo, M. M. and Xiong, X. N. and Ou, C. L. and You, L. M. and Chen, S. X. and Zhang, M. |
| 541 | Wrong outcome | Effectiveness of Implementing Modified Early Warning System and Rapid Response Team for General Ward Inpatients | 2024 | Liaw, W. J. and Wu, T. J. and Huang, L. H. and Chen, C. S. and Tsai, M. C. and Lin, I. C. and Liao, Y. H. and Shen, W. C. |
| 542 | Wrong outcome | Effectiveness of Instruction Program on Nurses' Knowledgeabout Clinical Devices Alarm in Intensive Care Unit | 2021 | Obeid, A. S. and Hudabakerhassan |
| 543 | Wrong outcome | Effectiveness of nurse-led disease management programs on health outcomes and health service utilization in adult patients with chronic obstructive pulmonary disease: A systematic review protocol | 2013 | Poon Chung Leung, Henry and Chan Sau, Man and Yu Sau, Fung |
| 544 | Wrong outcome | Effectiveness of nurse-led disease management programs on health outcomes and health service utilization in adult patients with chronic obstructive pulmonary disease: A systematic review protocol | 2013 | Henry, P. C. L. and Man, C. S. and Fung, Y. S. |
| 545 | Wrong outcome | Effectiveness of pharmacist's intervention in the management of cardiovascular diseases | 2018 | Omboni, S. and Caserini, M. |
| 546 | Wrong outcome | Effectiveness of remote patient monitoring after discharge of hospitalized patients with heart failure the better effectiveness after transition-heart failure (BEAT-HF) randomized clinical trial | 2016 | Ong, M. K. and Romano, P. S. and Edgington, S. and Aronow, H. U. and Auerbach, A. D. and Black, J. T. and De Marco, T. and Escarce, J. J. and Evangelista, L. S. and Hanna, B. and Ganiats, T. G. and Greenberg, B. H. and Greenfield, S. and Kaplan, S. H. and Kimchi, A. and Liu, H. and Lombardo, D. and Mangione, C. M. and Sadeghi, B. and Sadeghi, B. and Sarrafzadeh, M. and Tong, K. and Fonarow, G. C. and Davidson, B. and Ghasemzadeh, H. and Gropper, M. and Mourad, M. and Ahmadpour, A. and Davila, W. and Engel, S. and Jacolbia, R. and Lee, H. and Linares, L. and Michel, E. and Weyrich, M. S. and Zellmer, E. and Esbati-Mashayekhi, L. and Haddad, E. and Haskins, M. and Larson, T. and Pratt, K. and Ansorie, H. and Aoki, K. and Baron, R. and Brinker, E. and Carroll, M. and Contasti, A. and Fekete, A. and Guzman, V. and Larsen, L. and Martinez, L. and Myers, S. and Schimmel, M. and Schnell-Heringer, A. and Taylor, A. and Tooley, T. and Van Den Brande, G. and Zaharias, E. and Billimek, J. and Castaneda, R. and Reyes, M. E. D. and Fine, D. and Lo, T. and Luu, X. and Ochoa, S. and Perez, M. and Rincon, D. and Sillas, F. and Uy, V. and Wang, E. and Xu, H. and Yala, S. and Yan, T. |
| 547 | Wrong outcome | Effectiveness of Targeted Nursing Measures to Relieve Swollen Limb Pain after Extremity Fracture | 2024 | Wang, X. and Xu, H. and Wu, X. |
| 548 | Wrong outcome | Effects of a bedbath on mixed venous oxygen saturation and heart rate in coronary artery bypass graft patients | 1994 | Atkins, P. J. and Hapshe, E. and Riegel, B. |
| 549 | Wrong outcome | Effects of a diuretic adjustment algorithm protocol on heart failure admissions: A randomized clinical trial | 2021 | FeijÃ³, M. K. and Ruschel, K. B. and Bernardes, D. and Ferro, E. B. and Rohde, L. E. and Biolo, A. and Rabelo da Silva, E. R. |
| 550 | Wrong outcome | Effects of a nurse-led heart failure clinic on hospital readmission and mortality in Hong Kong | 2016 | Cheng, H. Y. and Chair, S. Y. and Wang, Q. and Sit, J. W. and Wong, E. M. and Tang, S. W. |
| 551 | Wrong outcome | Effects of a Technology-Assisted Integrated Diabetes Care Program on Cardiometabolic Risk Factors among Patients with Type 2 Diabetes in the Asia-Pacific Region: The JADE Program Randomized Clinical Trial | 2021 | Lim, L. L. and Lau, E. S. H. and Fu, A. W. C. and Ray, S. and Hung, Y. J. and Tan, A. T. B. and Chamnan, P. and Sheu, W. H. H. and Chawla, M. S. and Chia, Y. C. and Chuang, L. M. and Nguyen, D. C. and Sosale, A. and Saboo, B. D. and Phadke, U. and Kesavadev, J. and Goh, S. Y. and Gera, N. and Huyen Vu, T. T. and Ma, R. C. W. and Lau, V. and Luk, A. O. Y. and Kong, A. P. S. and Chan, J. C. N. |
| 552 | Wrong population | The effects of an educational programme about preeclampsia on women's awareness: a randomised control trial | 2020 | Alnuaimi, K. and Abuidhail, J. and Abuzaid, H. |
| 553 | Wrong outcome | Effects of anesthesia recovery nursing combined with heat-preservation nursing on the state of stress and recovery agitation of general anesthesia patients during surgery | 2020 | Chen, N. and Li, G. and Yao, B. and Wang, D. and Mao, X. |
| 554 | Wrong outcome | The effects of different positions on saturation and vital signs in patients | 2021 | Alan, Nurten and Khorshid, Leyla |
| 555 | Wrong outcome | EFFECTS OF FUNCTIONAL TRAINING ON POSTOPERATIVE ANTERIOR CRUCIATE INJURY IN ATHLETES HOSPITALIZED | 2022 | Zhang, F. and Wang, C. and Chen, X. and Li, H. |
| 556 | Wrong population | The effects of giving pacifiers to premature infants and making them listen to lullabies on their transition period for total oral feeding and sucking success | 2012 | Yildiz, A. and Arikan, D. |
| 557 | Wrong outcome | Effects of growth trajectory of shock index within 24 h on the prognosis of patients with sepsis | 2022 | Xu, F. and Zhang, L. and Huang, T. and Han, D. and Yang, R. and Zheng, S. and Feng, A. and Huang, L. and Yin, H. and Lyu, J. |
| 558 | Wrong outcome | The effects of home monitoring by public health nurse on individuals' diabetes control | 2006 | Kitis, Y. and Emiroglu, O. N. |
| 559 | Wrong outcome | The effects of implementation cardiac rehabilitation program using a mobile application on activity tolerance, fatigue, and dyspnea in patients with myocardial infraction; a randomized clinical trial study | 2021 | Etemadifar, S. and Davoodvand, S. and Tahmasebian, S. and Sedehi, M. and Esmaeili, Z. |
| 560 | Wrong population | Effects of Kangaroo Mother Care on Physiological Parameters of Low-Birth-Weight Neonates | 2023 | Mateen, A. and Awan, N. N. and Gul, S. S. and Adeel, B. and Razzak, S. and Mushtaq, S. |
| 561 | Wrong outcome | The effects of leg/body position on transcutaneous oxygen measurements after lower-extremity arterial revascularization | 2008 | Rich, K. |
| 562 | Wrong outcome | Effects of light intensity on the physiological parameters of the premature infant | 2001 | Peng, N. and Mao, H. and Chen, Y. and Chang, Y. |
| 563 | Wrong outcome | Effects of methylprednisolone infusions on vital signs in children with headaches | 2013 | Heidrich, E. and Greene, G. and Weberding, J. and Lin, L. and McGee, S. |
| 564 | Wrong outcome | Effects of Music Intervention on State Anxiety and Physiological Indices in Patients Undergoing Mechanical Ventilation in the Intensive Care Unit | 2017 | Lee, C. H. and Lee, C. Y. and Hsu, M. Y. and Lai, C. L. and Sung, Y. H. and Lin, C. Y. and Lin, L. Y. |
| 565 | Wrong outcome | Effects of Noise on Vital Signs and Anxiety Levels of Patients Hospitalized in the General Surgery Intensive Care Unit | 2021 | Serap, Gungor and Gursel, Oztunc |
| 566 | Wrong outcome | Effects of normal saline on endotracheal suctioning | 2002 | AkgÃ¼l, S. and Akyolcu, N. |
| 567 | Wrong outcome | Effects of occupied and unoccupied bed making on myocardial work in healthy subjects | 1991 | Futrell, A. G. and Forst, S. and Harrell, J. S. and Adams, L. F. |
| 568 | Wrong outcome | Effects of Positioning on Respiration Rate, Heart Rate, and Oxygen Saturation in Preterm Infants During Feeding: A Cross-over Design | 2018 | Busakorn, Punthmatharith and Jananya, Mora |
| 569 | Wrong outcome | Effects of progressive muscle relaxation exercises on the vital signs and fatigue in kidney transplant patients: a randomized controlled trial | 2024 | Uzun YaÄŸÄ±z, Åž and AvcÄ± IÅŸÄ±k, S. |
| 570 | Wrong outcome | Effects of technology-enabled blood pressure monitoring in primary care: A quasi-experimental trial | 2024 | Teo, V. H. and Teo, S. H. and Burkill, S. M. and Wang, Y. and Chew, E. A. and Ng, D. W. and Tang, W. E. and Koh, G. C. |
| 571 | Wrong outcome | Effects of the nursing practice environment, nurse staffing, patient surveillance and escalation of care on patient mortality: A multi-source quantitative study | 2024 | Al-Ghraiybah, T. and Lago, L. and Fernandez, R. and Sim, J. |
| 572 | Wrong outcome | Effects of Transport on Oral Temperature of Postsurgical Patients Transported from PACU to Nursing Units | 2020 | Dureault, Kathy and Winokur, Elizabeth J. and Rutledge, Dana N. |
| 573 | Wrong outcome | The effects on rehospitalization rate of transitional care using information communication technology in patients with heart failure: A scoping review | 2023 | Qi, K. and Koike, T. and Yasuda, Y. and Tayama, S. and Wati, I. |
| 574 | Wrong outcome | Efficacy and Cost-Effectiveness Analysis of Evidence-Based Nursing Interventions to Maintain Tissue Integrity to Prevent Pressure Ulcers and Incontinence-Associated Dermatitis | 2018 | AvÅŸar, P. and KaradaÄŸ, A. |
| 575 | Wrong outcome | The efficacy and safety of a chest pain protocol for short stay unit patients: A one year follow-up | 2015 | Lee, G. and Dix, S. and Mitra, B. and Coleridge, J. and Cameron, P. |
| 576 | Wrong outcome | Efficacy of an enhanced recovery nursing plan as a rooming-in practice for women with preeclampsia post-cesarean section | 2024 | Li, J. and Zhao, W. Y. and Zhuang, Y. and Gu, N. and Wang, M. Q. and Zheng, Y. N. and Wang, J. X. |
| 577 | Wrong outcome | Efficacy of aromatherapy with Lavandula angustifolia oil on postoperative pain after cardiac surgery: A randomized clinical trial | 2024 | Silva, L. C. D. M. A. and dos Santos, K. V. G. and dos Santos, J. J. D. S. and Camara, R. P. D. P. O. A. and Bezerra e Silva, S. Y. and Silva, H. M. M. D. and Ribeiro, K. R. B. and Dantas, D. V. and Dantas, R. A. N. |
| 578 | Wrong outcome | Efficacy of frequent blood pressure and heart rate monitoring for early identification of bleeding following percutaneous coronary intervention | 2012 | Mert, Hatice and Seren Intepeler, Seyda and Bengu, Nergiz and Baturlar, Zuhal and Istan, Pakize and Ozcelik, Ebru |
| 579 | Wrong outcome | Efficacy of Phase II Remote Home Rehabilitation in Patients with Acute Myocardial Infarction after Percutaneous Coronary Intervention | 2022 | Li, Z. and Hui, Z. and Zheng, Y. and Yu, J. and Zhang, J. |
| 580 | Wrong population | Efficacy of Warm Showers on Labor Pain and Birth Experiences During the First Labor Stage | 2013 | Lee, S. L. and Liu, C. Y. and Lu, Y. Y. and Gau, M. L. |
| 581 | Wrong outcome | Efficiency of warming devices in extubated postoperative patients | 1994 | Weyland, W. and Fritz, U. and Fabian, S. and Jaeger, H. and Crozier, T. and Kieztmann, D. and Braun, U. |
| 582 | Wrong outcome | eHealth-Generated Patient Data in an Outpatient Setting after Hematopoietic Stem Cell Transplantation: A Scoping Review | 2022 | Van Opstal, J. and Zhao, A. T. and Kaplan, S. J. and Sung, A. D. and Schoemans, H. |
| 583 | Wrong outcome | eHOME for an integrated care approach for drug-related problems in homeliving older people | 2018 | Dijkstra, Nienke and Hiddink, Eric and Sino, Carolien |
| 584 | Wrong outcome | Electronic "Facility Board" Provides Easy Look at Patient Status | 2016 | Helwick, Caroline |
| 585 | Wrong outcome | Electronic Alert Signal for Early Detection of Tissue Injuries in Patients: An Innovative Pressure Sensor Mattress | 2023 | Mamom, J. and Rungroungdouyboon, B. and Daovisan, H. and Sri-Ngernyuang, C. |
| 586 | Wrong outcome | ELECTRONIC MONITORING OF VITAL SIGNS | 1965 | Geroge, J. H. |
| 587 | Wrong outcome | Electronic recording of transfusion-related patient observations: a comparison of two bedside systems | 2017 | Staples, S. and Noel, S. and Watkinson, P. and Murphy, M. F. |
| 588 | Wrong outcome | Emergency department care-related causal factors of in-patient deterioration | 2022 | Nassief, K. and Azer, M. and Watts, M. and Tuala, E. and McLennan, P. and Curtis, K. |
| 589 | Wrong outcome | Emergency department waiting room nurses in practice: An observational study | 2018 | Innes, Kelli and Elliott, Doug and Plummer, Virginia and Jackson, Debra |
| 590 | Wrong population | Emergency Nursing Based on PEWS can Improve the Condition of Children with Acute Asthma | 2024 | Wang, L. and Zheng, S. and Wang, Q. and Ma, J. and Zhang, S. and Ma, J. and Ma, Y. and Chang, C. and Cui, Y. |
| 591 | Wrong outcome | Emergency rescue of a patient with hemorrhagic shock caused by superior mesenteric artery rupture: A case report | 2024 | Lin, X. P. and Guo, X. L. and Tian, H. F. and Wu, Z. R. and Yang, W. J. and Pan, H. Y. |
| 592 | Wrong outcome | An empirical approach to estimating the effect of e-health on medical expenditure | 2010 | Akematsu, Y. and Tsuji, M. |
| 593 | Wrong outcome | Employer-sponsored wellness programs for hypertension and dyslipidemia in a 2-hospital health system | 2019 | Misher, A. and Brown, J. and Maguire, C. and Schnibben, A. P. |
| 594 | Wrong outcome | Employment of telemedicine in nursing homes: Clinical requirement analysis, system development and first test results | 2020 | Ohligs, M. and Stocklassa, S. and Rossaint, R. and Czaplik, M. and Follmann, A. |
| 595 | Wrong outcome | End digit preference in blood pressure measurement in a hypertension specialty clinic in southwest Nigeria | 2012 | Ayodele, O. E. and Sanya, E. O. and Okunola, O. O. and Akintunde, A. A. |
| 596 | Wrong outcome | End-of-Life Care and Quality of Dying in 23 Acute Geriatric Hospital Wards in Flanders, Belgium | 2017 | Verhofstede, Rebecca and Smets, Tinne and Cohen, Joachim and Eecloo, Kim and Costantini, Massimo and Van Den Noortgate, Nele and Deliens, Luc |
| 597 | Wrong outcome | Energy expenditure, heart rate, work pace, and their associations with perceived workload among female hospital nurses working 12-hour day shift | 2009 | Chen, J. |
| 598 | Wrong outcome | Engaging Frontline Providers Prevents Hypothermia and Improves Communication in the Postoperative Neonate | 2021 | Guidash, J. C. and Berman, L. and Panagos, P. G. and Sullivan, K. M. |
| 599 | Wrong outcome | Engaging nurses in clinical research | 2001 | FitzGerald, M. and McCutcheon, H. and Court, A. and Athanasiadis, K. |
| 600 | Wrong outcome | Engaging staff to improve quality and safety in an austere medical environment: a case-control study in two Sierra Leonean hospitals | 2015 | Rosen, Michael A. and Chima, Adaora M. and Sampson, John B. and Jackson, Eric V., Jr. and Koka, Rahul and Marx, Megan K. and Kamara, Thaim B. and Ogbuagu, Onyebuchi U. and Lee, Benjamin H. |
| 601 | Wrong outcome | Enhanced peri-operative care to improve outcomes for high-risk surgical patients in Brazil: a single-centre before-and-after cohort study | 2022 | Stahlschmidt, A. and Passos, S. C. and Cardoso, G. R. and Schuh, G. J. and Gutierrez, C. S. and Castro, S. M. J. and Caumo, W. and Pearse, R. M. and Stefani, L. C. |
| 602 | Wrong outcome | Enhanced Telehealth Home-Monitoring Intervention for Vulnerable and Frail Patients after Cardiac Surgery (THE-FACS Pilot Intervention Study) | 2022 | Sarkar, S. and MacLeod, J. and Hassan, A. and Brunt, K. R. and Palmer, K. and LÃ©garÃ©, J. F. |
| 603 | Wrong outcome | ENHANCING PATIENT SAFETY THROUGH MEDICATION RECONCILIATION IN SMALL HOSPITAL SETTINGS: A PROSPECTIVE ANALYSIS | 2024 | Hadi, A. E. and Rahman, W. U. and Sarkar, N. U. and Ismail, M. A. M. and Ullah, S. and Joy, A. A. and Tariq, A. |
| 604 | Wrong outcome | Environmental factors related to sleep latency among inpatients in rehabilitation wards according to functional independence measure cognitive scores | 2022 | Fukui, Sakiko and Ohama, Etsuko and Hattori, Satoshi |
| 605 | Wrong outcome | Epidural and intrathecal opioids for postoperative pain management in Europe - A 17-nation questionnaire study of selected hospitals | 1996 | Rawal, N. and Allvin, R. and Neumark, J. and Sosnowski, M. and KrÃ¸ner, K. and Nuutinen, L. and Bonnet, F. and Hempel, V. and Vadaloukas, A. and Hirlekar, G. and Assaf, R. and Capogna, G. and Hasenbos, M. and BjÃ¸rgo, S. and Campos, R. and BaÃ±os, J. E. and CaÃ±ellas, M. and Buchser, E. and Wheatley, R. |
| 606 | Wrong outcome | Epidural, Inadvertent Subdural, and Combined Epiduralâ€“Subdural Anesthesia in Lumbar Spine Surgery: A Retrospective Analysis | 2024 | Kang, S. Y. and Cho, H. S. and Yi, J. and Jung, S. C. and Kim, H. S. and Jang, I. T. and Kang, H. |
| 607 | Wrong outcome | Epilepsy in the elderly: Prognosis | 1986 | Luhdorf, K. and Jensen, L. K. and Plesner, A. M. |
| 608 | Wrong outcome | Equipment used for safe mobilization of the ICU patient | 2013 | Asher, A. |
| 609 | Wrong outcome | Equivalence of temperature measurement methods in the adult hematology/oncology population | 2015 | Mason, T. M. and Reich, R. R. and Carroll, M. E. and Lalau, J. and Smith, S. and Boyington, A. R. |
| 610 | Wrong outcome | Escalation Pathways of Remote Patient Monitoring Programs for COVID-19 Patients in Canada and the United States: A Rapid Review | 2024 | Hicks, N. and Zhan, J. and Brual, J. and Abejirinde, I. O. and Alfred, M. |
| 611 | Wrong outcome | Establishing a nurse-based, anesthesiologist-supervised inpatient acute pain service: Experience of 4,617 patients | 2004 | Shapiro, A. and Zohar, E. and Kantor, M. and Memrod, J. and Fredman, B. |
| 612 | Wrong outcome | Establishing a transplant coordinator-led living kidney donor follow-up clinic | 2003 | Lumsdaine, J. A. and Wigmore, S. J. and Wooton, D. and Stewart, C. and Akyol, M. and Forsythe, J. L. R. |
| 613 | Wrong outcome | Estimating geriatric patient's body weight using the knee height caliper and mid-arm circumference in Hong Kong Chinese | 2004 | Jung, M. Y. and Chan, M. S. and Chow, V. S. and Chan, Y. T. and Leung, P. F. and Leung, E. M. and Lau, T. Y. and Man, C. W. and Lau, J. T. and Wong, E. M. |
| 614 | Wrong outcome | Estimation of fluid status changes in critically ill patients: Fluid balance chart or electronic bed weight? | 2012 | Schneider, Antoine G. and Baldwin, Ian and Freitag, Elke and Glassford, Neil and Bellomo, Rinaldo |
| 615 | Wrong outcome | ESTRATEGIA EDUCATIVA PARA EL CUIDADO DOMICILIARIO DE LOS BEBES PREMATUROS: MADRES USUARIAS DEL PROGRAMA MADRE CANGURO DE TUNJA, COLOMBIA | 2013 | Araque Salazar, Sonia and Ariza RiaÃ±o, Nelly Esperanza and Valderrama Sanabria, Mery Luz |
| 616 | Wrong outcome | An ethnographic observational study to evaluate and optimize the use of respiratory acoustic monitoring in children receiving postoperative opioid infusions | 2016 | GÃ¶rges, M. and West, N. C. and Christopher, N. A. and Koch, J. L. and Brodie, S. M. and Lowlaavar, N. and Lauder, G. R. and Ansermino, J. M. |
| 617 | Wrong outcome | The EU project "United4Health": Results and experiences from automatic health status assessment in a Norwegian telemedicine trial system | 2019 | Gerdes, Martin and Gallefoss, Frode and Fensli, Rune Werner |
| 618 | Wrong population | European survey on Paediatric Early Warning Systems, and other processes used to aid the recognition and response to children's deterioration on hospital wards | 2024 | Gawronski, O. and Briassoulis, G. and El Ghannudi, Z. and Ilia, S. and SÃ¡nchez-MartÃ­n, M. and Chiusolo, F. and Jensen, C. S. and Manning, J. C. and Valla, F. V. and Pavelescu, C. and Dall'Oglio, I. and Coad, J. and Sefton, G. |
| 619 | Wrong outcome | Evaluating a novel, integrative dashboard for health professionalsâ€™ performance in managing deteriorating patients: quality improvement project | 2022 | Alhmoud, B. and Melley, D. and Khan, N. and Bonnici, T. and Patel, R. and Banerjee, A. |
| 620 | Wrong outcome | Evaluating changes in ambient ozone and respiratory-related healthcare utilization in the Washington, DC metropolitan area | 2020 | Fuller, C. H. and Jones, J. W. and Roblin, D. W. |
| 621 | Wrong outcome | EVALUATING CRITICAL CARE OUTREACH AND THE EARLY WARNING SCORE TOOL -- THE WARD NURSE'S VIEWPOINT | 2013 | Salt, Lynn |
| 622 | Wrong outcome | Evaluating culture practices used to identify infection in patients with brain injury | 2012 | Hillier, Robin and Everett, Brenda |
| 623 | Wrong outcome | Evaluating the documentation of vital signs following implementation of a new comprehensive newborn monitoring chart in 19 hospitals in Kenya: A time series analysis | 2023 | Muinga, N. and Tuti, T. and Mwaniki, P. and Gicheha, E. and Paton, C. and BeÅˆovÃ¡, L. and English, M. |
| 624 | Wrong outcome | Evaluating the Effectiveness of a Training on Ergonomic Risks of Measuring Blood Pressure | 2024 | DÑ–rgar, E. and Olgun, N. |
| 625 | Wrong outcome | Evaluating the understanding of nurses regarding pain management in neonatal units and special neonatal units of Qamar Monir Bani Haeshem Hospital in Khoy, Iran, in 2016 | 2017 | Zinalpoor, S. and Sakhaei, S. and Sadagheyani, H. E. and Mollabashi, L. M. and Motaaref, H. |
| 626 | Wrong outcome | Evaluating the validity and reliability of the V-scale instrument (Turkish version) used to determine nurses' attitudes towards vital sign monitoring | 2018 | ErtuÄŸ, N. |
| 627 | Wrong outcome | Evaluation and follow-up of the quality of medical care offered to intoxicated patients in an Emergency Department | 2007 | NoguÃ©-Xarau, S. and AmigÃ³-TadÃ­n, M. and SÃ¡nchez-SÃ¡nchez, M. and SalmerÃ³n Bargo, J. M. |
| 628 | Wrong outcome | Evaluation of a continuous epidural analgesia program for postoperative pain in children | 2007 | Ellis, J. A. and Martelli, B. and LaMontagne, C. and Splinter, W. |
| 629 | Wrong outcome | Evaluation of a MS specialist nurse programme | 2006 | Forbes, A. and While, A. and Mathes, L. and Griffiths, P. |
| 630 | Wrong outcome | Evaluation of a National Broadband Network-enabled Telehealth trial for older people with chronic disease | 2016 | Nancarrow, Susan and Banbury, Annie and Buckley, Jennene |
| 631 | Wrong outcome | Evaluation of a new respiratory monitoring tool "Early Warning ScoreO2" for patients admitted at the emergency department with dyspnea | 2020 | Viglino, Damien and L'Her, Erwan and Maltais, FranÃ§ois and Maignan, Maxime and Lellouche, FranÃ§ois |
| 632 | Wrong outcome | Evaluation of a new smartphone optical blood pressure application (OptiBPâ„¢) in the post-anesthesia care unit: a method comparison study against the non-invasive automatic oscillometric brachial cuff as the reference method | 2022 | Desebbe, O. and El Hilali, M. and Kouz, K. and Alexander, B. and Karam, L. and Chirnoaga, D. and Knebel, J. F. and Degott, J. and Schoettker, P. and Michard, F. and Saugel, B. and Vincent, J. L. and Joosten, A. |
| 633 | Wrong population | Evaluation of a new tool - "Step by step with my baby" - to support parental involvement in the care of preterm infants | 2024 | Zores, C. and Gibier, C. and Haumesser, L. and Meyer, N. and Poirot, S. and Briot, C. and Langlet, C. and Dillenseger, L. and Kuhn, P. |
| 634 | Wrong outcome | Evaluation of a Nurse-Driven Fluid Management Protocol to Improve Outcomes in Critically Ill Patients | 2023 | Barstow, Loraine and Tola, Denise H. and Smallheer, Benjamin |
| 635 | Wrong outcome | Evaluation of a radial artery cannulation training program for intensive care nurses: A descriptive, explorative study | 2011 | Chee, Bee C. and Baldwin, Ian C. and Shahwan-Akl, Lina and Fealy, Nigel G. and Heland, Melodie J. and Rogan, John J. |
| 636 | Wrong outcome | Evaluation of a Web Application for Nursing Records of Multiple Trauma Patients in an Emergency Department | 2022 | Phaken, C. and Pearkao, C. and Potisopha, W. and Angkasith, P. |
| 637 | Wrong outcome | Evaluation of a wireless, portable, wearable multi-parameter vital signs monitor in hospitalized neurological and neurosurgical patients | 2018 | Weller, Robert S. and Foard, Kristina L. and Harwood, Timothy N. |
| 638 | Wrong population | An evaluation of Acute Care of at-Risk Newborns (ACoRN), a Canadian education program, in Chinese neonatal nurseries | 2020 | Aziz, Khalid and Ma, Xiaolu and Lockyer, Jocelyn and McMillan, Douglas and Ye, Xiang Y. and Du, Lizhong and Lee, Shoo K. and Singhal, Nalini |
| 639 | Wrong outcome | Evaluation of an integrated intensive care unit monitoring display by critical care fellow physicians | 2012 | GÃ¶rges, M. and Westenskow, D. R. and Markewitz, B. A. |
| 640 | Wrong outcome | An evaluation of body temperature measurement | 1983 | Ilsley, A. H. and Rutten, A. J. and Runciman, W. B. |
| 641 | Wrong outcome | An evaluation of body temperature measurement | 1983 | Ilsley, A. H. and Runciman, W. B. |
| 642 | Wrong outcome | Evaluation of clinical application effect of wireless temperature and pulse measuring system | 2023 | Dan, K. and Guangfei, C. and Wensu, W. and Jiahui, L. and Yuan, G. and Ling, G. |
| 643 | Wrong outcome | Evaluation of Nonpharmacologic Interventions and Sleep Outcomes in Hospitalized Medical and Surgical Patients: A Nonrandomized Controlled Trial | 2022 | van den Ende, Eva S. and Merten, Hanneke and Van der Roest, Lisanne and Toussaint, Belle and van Rijn, Quirine and Keesenberg, Marjolein and Lodders, Anne M. and van Veldhuizen, Kim and Vos, Iris E. and Hoekstra, Sophie and Nanayakkara, Prabath W. B. |
| 644 | Wrong outcome | Evaluation of nursing care -- using conjoint analysis | 2006 | Anezaki, H. and Aso, Y. and Ohkusa, Y. |
| 645 | Wrong outcome | Evaluation of patients' sleep by nurses in an ICU | 2016 | Ritmala-Castren, M. and Virtanen, I. and Vahlberg, T. and Leivo, S. and Kaukonen, K. M. and Leino-Kilpi, H. |
| 646 | Wrong outcome | Evaluation of physiological work demands and low back neuromuscular fatigue on nurses working in geriatric wards | 2001 | Hui, L. and Ng, G. Y. and Yeung, S. S. and Hui-Chan, C. W. |
| 647 | Wrong outcome | Evaluation of Postoperative Warming Care Protocol for Thermal Comfort and Temperature Management Immediately After Surgery: Nonrandomized Controlled Trial | 2023 | Kameda, Norihiro and Okada, Shinobu |
| 648 | Wrong outcome | Evaluation of skills and knowledge on orthostatic blood pressure measurements in elderly patients | 2002 | Vloet, L. C. and Smits, R. and Frederiks, C. M. and Hoefnagels, W. H. and Jansen, R. W. |
| 649 | Wrong outcome | Evaluation of Temporal Artery And Disposable Digital Oral Thermometers in Acutely Ill Patients | 2014 | Counts, Diane and Acosta, Mary and Holbrook, Holly and Foos, Eileen and Hays-Ponder, Kimberly and Macairan, Olga and Thomas, Linda and Whitsett, Maryse and Williams, Lori and Twiss, Elizabeth J. |
| 650 | Wrong outcome | Evaluation of the ARI program: a health facility survey in Simbu, Papua, New Guinea | 1993 | Brewster, D. R. and Pyakalyia, T. and Hiawalyer, G. and O'Connell, D. L. |
| 651 | Wrong outcome | Evaluation of the Cadi ThermoSENSOR wireless skin-contact thermometer against ear and axillary temperatures in children | 2010 | Ng, K. G. and Wong, S. T. and Lim, S. M. and Goh, Z. |
| 652 | Wrong outcome | Evaluation of the causal effects between dopamine infusion changeover and fluctuations in mean arterial pressure in neonates | 2020 | Kirupakaran, K. and de Sousa, P. and Le Roux, C. and Redwood, L. and Rabe, H. and Patel, B. A. |
| 653 | Wrong outcome | Evaluation of the Effect of a Critical Care Follow-up Program on Patient Outcomes | 2017 | Hang Mui, So |
| 654 | Wrong population | Evaluation of the effect of sound intensity on vital signs in neonatal intensive care unit | 2020 | BaÅŸaranoÄŸlu, M. and Karaman, S. and SÃ¶nmez, B. and Tuncer, O. |
| 655 | Wrong outcome | Evaluation of the effectiveness of a segmented alternating shift pattern based on wearable vital signs monitoring devices during COVID-19: a cross-sectional study | 2024 | Xie, M. and Zhang, Z. and Jin, R. and Chen, X. and Liu, Z. and Ma, J. and Qiao, W. |
| 656 | Wrong outcome | Evaluation of the effectiveness of the Pastormaster method for disinfection of legionella in a hospital water distribution system | 2005 | PeirÃ³ Callizo, E. F. and DarpÃ³n Sierra, J. and Santos Pombo, J. M. and Ezpeleta Baquedano, C. and PÃ©rez Huerta, B. |
| 657 | Wrong outcome | Evaluation of the nurse-assisted eHealth intervention 'eHealth@Hospital-2-Home' on self-care by patients with heart failure and colorectal cancer post-hospital discharge: protocol for a randomised controlled trial | 2024 | Storm, M. and Morken, I. M. and Austin, R. C. and Nordfonn, O. and Wathne, H. B. and Urstad, K. H. and Karlsen, B. and Dalen, I. and Gjeilo, K. H. and Richardson, A. and Elwyn, G. and Bru, E. and SÃ¸reide, J. A. and KÃ¸rner, H. and Mo, R. and StrÃ¶mberg, A. and LurÃ¥s, H. and HusebÃ¸, A. M. L. |
| 658 | Wrong outcome | Evaluation of the safety and efficacy of deep sedation for electrophysiology procedures administered in the absence of an anesthetist | 1997 | Geiger, M. J. and Wase, A. and Kearney, M. M. and Brandon, M. J. and Kent, V. and Newby, K. H. and Natale, A. |
| 659 | Wrong outcome | An evaluation of trauma team response in a major Trauma Hospital in 100 patients with predominantly minor injuries | 2000 | Lu, W. H. and Kolkman, K. and Seger, M. and Sugrue, M. |
| 660 | Wrong outcome | Evidence-based new service package vs. routine service package for smoking cessation to prevent high risk patients from cardiovascular diseases (CVD): study protocol for randomized controlled trial | 2013 | Aung, M. N. and Yuasa, M. and Lorga, T. and Moolphate, S. and Fukuda, H. and Kitajima, T. and Yokokawa, H. and Minematsu, K. and Tanimura, S. and Hiratsuka, Y. and Ono, K. and Naunboonruang, P. and Thinuan, P. and Kawai, S. and Suya, Y. and Chumvicharana, S. and Marui, E. |
| 661 | Wrong outcome | Evidence-practice gaps in initial neuro-protective nursing care: A mixed methods study of Thai patients with moderate or severe traumatic brain injury | 2021 | Promlek, K. and Currey, J. and Damkliang, J. and Considine, J. |
| 662 | Wrong outcome | An Examination of the Nursing Records of Cerebrovascular Disease Patients in Intensive Care | 2017 | Gencturk, Nuran and Ay, Fatma and Demirci, Åženay and Acamur, Zehra and IzdeÅŸ, Seval and Bulut, Aygul |
| 663 | Wrong outcome | Expectations of Continuous Vital Signs Monitoring for Recognizing Complications After Esophagectomy: Interview Study Among Nurses and Surgeons | 2021 | van Rossum, M. and Leenen, J. and Kingma, F. and Breteler, M. and van Hillegersberg, R. and Ruurda, J. and Kouwenhoven, E. and van Det, M. and Luyer, M. and Nieuwenhuijzen, G. and Kalkman, C. and Hermens, H. |
| 664 | Wrong outcome | Experience Caring for a Severe COVID-19 Patient With ARDS in the Intensive Care Unit | 2020 | Wang, Y. P. and Chuang, P. Y. and Gone, S. I. and Tseng, C. Y. |
| 665 | Wrong outcome | The experience of setting up a resident-managed Acute Pain Service: a descriptive study | 2016 | Borracci, T. and Prencipe, D. and Masotti, A. and Nella, A. and Tuccinardi, G. and Margiacchi, L. and Villa, G. and Pinelli, F. and Romagnoli, S. and De Gaudio, A. R. and Zagli, G. |
| 666 | Wrong outcome | Experience of treating batches of exertional heat stroke patients in military training | 2020 | Jiao, W. and Yuxiang, Z. |
| 667 | Wrong outcome | [Experience of treating batches of exertional heat stroke patients in military training] | 2020 | Wang, J. and Zhang, Y. |
| 668 | Wrong outcome | Experience with planned and coordinated care using telemedicine | 2007 | Taylor, D. M. and Capamagian, L. |
| 669 | Wrong outcome | The experiences of nurses implementing the Modified Early Warning Score and a 24-hour on-call Mobile Intensive Care Nurse: An exploratory study | 2016 | Stafseth, S. K. and GrÃ¸nbeck, S. and Lien, T. and Randen, I. and Lerdal, A. |
| 670 | Wrong outcome | [Experiences with scoring systems SAPS II and NEMS for registration of activities in an intensive care unit] | 2001 | Haagensen, R. and Jamtli, B. and Moen, H. and Stokland, O. |
| 671 | Wrong outcome | Exploration of assistance and rehabilitation possibilities for neurosurgical patients with late complications after craniocerebral injuries based on one patient case | 2012 | BiaÅ‚kowska, J. and Sowa, M. and Maksymowicz, W. |
| 672 | Wrong outcome | An exploratory data quality analysis of time series physiologic signals using a large-scale intensive care unit database | 2021 | Afshar, A. S. and Li, Y. and Chen, Z. and Chen, Y. and Lee, J. H. and Irani, D. and Crank, A. and Singh, D. and Kanter, M. and Faraday, N. and Kharrazi, H. |
| 673 | Wrong outcome | Exploring Nursing Care for Patients With COVID-19 Using International Classification for Nursing Practice-Based Nursing Records | 2024 | Sung, S. and Jung, H. and Kim, Y. |
| 674 | Wrong outcome | Exploring Nursing Care for Patients With COVID-19 Using International Classification for Nursing Practiceâ€“Based Nursing Records | 2024 | Sumi, Sung and Hyesil, Jung and Youlim, Kim |
| 675 | Wrong population | Exploring physiological stability of infants in Kangaroo Mother Care position versus placed in transport incubator during neonatal ground ambulance transport in Sweden | 2022 | van den Berg, Johannes and Jakobsson, Ulf and Selander, Bo and Lundqvist, Pia |
| 676 | Wrong outcome | Exploring the Frequency of Blood Pressure Documentation in Emergency Departments | 2014 | Miltner, Rebecca S. and Johnson, Kimberly D. and Deierhoi, Rhiannon |
| 677 | Wrong outcome | Exploring the guidelines for the management of severe head injury | 2000 | Iacono, L. A. |
| 678 | Wrong outcome | Exploring the knowledge and skills for effective family caregiving in elderly home care: a qualitative study | 2024 | Hailu, G. N. and Abdelkader, M. and Asfaw, F. and Meles, H. A. |
| 679 | Wrong outcome | Exploring the Outcomes and Satisfaction of Automated Physiological Monitoring Systems Among Nurses | 2021 | Cheng, Y. C. and Lee, T. T. and Hwang, Y. T. and Chan, P. T. and Mills, M. E. |
| 680 | Wrong outcome | Exploring unplanned ICU admissions: a systematic review | 2011 | Vlayen, Annemie and Verelst, Sandra and Bekkering, Geertruida E. and Schrooten, Ward and Hellings, Johan and Claes, NerÃ©e |
| 681 | Wrong population | Extracorporeal membrane oxygenation therapy in children with acute fulminant myocarditis | 2015 | Zhou, H. Q. and Chen, B. and Ning, B. T. and Zhang, H. Y. |
| 682 | Wrong outcome | Facilitators and barriers to optimal home blood pressure management in patients with hypertensive disorders of pregnancy in a tertiary care facility in Abuja, Nigeria: a qualitative research study | 2023 | Mahmoud, Z. and Orji, A. A. and Okoye, C. F. and Ameh, F. O. and Jamro-Comer, E. and Isah, A. and Ekele, B. and Akaba, G. and Ojji, D. B. and Huffman, M. D. |
| 683 | Wrong outcome | Factors affecting the occurrence of pressure injuries among patients receiving targeted temperature management after cardiac arrest | 2023 | Ahn, S. and An, M. and Yoo, S. H. and Park, H. |
| 684 | Wrong outcome | Factors associated to the development of hypothermia in the intraoperative period | 2009 | Poveda, V. B. and GalvÃ£o, C. M. and Santos, C. B. |
| 685 | Wrong outcome | Factors associated with hospital length of stay in patients admitted with suspected malaria in Kenya: secondary analysis of a cross-sectional survey | 2022 | Machini, B. and Achia, T. N. O. and Kipruto, H. and Amboko, B. and Chesang, J. |
| 686 | Wrong outcome | Factors associated with the frequency of respiratory rate measurement by hospital nurses: a multicentre cross-sectional study | 2022 | Takayama, Atsushi and Takeshima, Taro and Nagamine, Takahiko |
| 687 | Wrong outcome | Factors Influencing Clinical Deterioration in Persons with Sepsis | 2017 | Patiporn, Bunyaphatkun and Siriorn, Sindhu and Davidson, Patricia M. and Ketsarin, Utriyaprasit and Chukiat, Viwatwongkasem and Wittaya, Chartbunchachai |
| 688 | Wrong outcome | Factors influencing partograph recording among skilled birth attendants at Mzuzu Central Hospital, Northern Malawi | 2021 | Kaunda, Prisca and Leshabari, Selbada and Mwale, Charles Masulani |
| 689 | Wrong outcome | Factors influencing the activation of the rapid response system for clinically deteriorating patients by frontline ward clinicians: a systematic review | 2017 | Chua, W. L. and See, M. T. A. and Legio-Quigley, H. and Jones, D. and Tee, A. and Liaw, S. Y. |
| 690 | Wrong outcome | Factors influencing the confidence in core clinical skills among hospital nurses | 2015 | Yang, Y. O. and Kim, M. and Park, K. Y. and Yang, J. H. |
| 691 | Wrong outcome | Factors Influencing the Quality of Standardized Treatment for Patients with Post-Cardiac Arrest Syndrome | 2017 | Lu, J. and Liu, L. and Zhu, J. and Guo, X. |
| 692 | Wrong outcome | Factors influencing the quality of vital sign data in electronic health records: A qualitative study | 2018 | Stevenson, J. E. and Israelsson, J. and Petersson, G. and Bath, P. A. |
| 693 | Wrong outcome | Factors influencing when intensive care unit nurses go to the bedside to investigate patient related alarms: A descriptive qualitative study | 2017 | Despins, L. A. |
| 694 | Wrong outcome | Factors predicting the development of pressure ulcers in an at-risk population who receive standardized preventive care: secondary analyses of a multicentre randomised controlled trial | 2015 | Demarre, L. and Verhaeghe, S. and Van Hecke, A. and Clays, E. and Grypdonck, M. and Beeckman, D. |
| 695 | Wrong outcome | The Factors that Affect the Frequency of Vital Sign Monitoring in the Emergency Department | 2014 | Johnson, Kimberly D. and Winkelman, Chris and Burant, Christopher J. and Dolansky, Mary and Totten, Vicken |
| 696 | Wrong outcome | Factors that influence nursesâ€™ assessment of patient acuity and response to acute deterioration | 2018 | Dalton, Mark and Harrison, John and Malin, Anitra and Leavey, Conan |
| 697 | Wrong outcome | Failure events in transition of care for surgical patients | 2014 | Helling, T. S. and Martin, L. C. and Martin, M. and Mitchell, M. E. |
| 698 | Wrong outcome | Failure to detect ward hypoxaemia and hypotension: contributions of insufficient assessment frequency and patient arousal during nursing assessments | 2021 | Saab, Remie and Wu, Bernie P. and Rivas, Eva and Chiu, Andrew and Lozovoskiy, Sofia and Ma, Chao and Yang, Dongsheng and Turan, Alparslan and Sessler, Daniel I. |
| 699 | Wrong outcome | Failure to Rescue as the Conceptual Basis for Nursing Clinical Peer Review | 2014 | Thielen, Jackie |
| 700 | Wrong outcome | Failure to rescue: A quality indicator for postoperative care | 2021 | Rosero, E. B. and Romito, B. T. and Joshi, G. P. |
| 701 | Wrong outcome | Fall prevention in hospitalized patients: Evaluation through the nursing outcomes classification/NOC | 2020 | de Freitas Luzia, M. and Vidor, I. D. and da Silva, Acfe and de FÃ¡tima Lucena, A. |
| 702 | Wrong outcome | Falls in the Nursing Home: Are they preventable? | 2005 | Vu, M. Q. and Weintraub, N. and Rubenstein, L. Z. |
| 703 | Wrong outcome | [Falls of patients in hospital: testing of a notification system and statistical monitoring] | 2010 | Bollini, G. and Lolli, A. and Cattin, P. and Zampieri, P. and Lamberti, M. |
| 704 | Wrong outcome | Family supplemented patient monitoring after surgery (SMARTER): a pilot stepped-wedge cluster-randomised trial | 2024 | Hewitt-Smith, A. and Bulamba, F. and Patel, A. and Nanimambi, J. and Adong, L. R. and Emacu, B. and Kabaleta, M. and Khanyalano, J. and Maiga, A. H. and Mugume, C. and Nakibuule, J. and Nandyose, L. and Sejja, M. and Weere, W. and Stephens, T. and Pearse, R. M. |
| 705 | Wrong population | A far-view intensive care unit monitoring display enables faster triage | 2011 | GÃ¶rges, M. and KÃ¼ck, K. and Koch, S. H. and Agutter, J. and Westenskow, D. R. |
| 706 | Wrong outcome | FBG-based smart bed system for healthcare applications | 2009 | Hao, J. and Jayachandran, M. and Kng, P. L. and Foo, S. F. and Aung Aung, P. W. and Cai, Z. |
| 707 | Wrong outcome | Feasibility and usability of patch-based continuous cardiac rhythm monitoring in comparison with traditional telemetry in noncritically ill hospitalized patients | 2019 | Amuthan, R. and Burkle, A. and Mould, S. and Tote, J. and Loy, M. and Kirkwood, D. and Meyer, J. and Pengel, S. and Hamilton, A. C. and Cantillon, D. J. |
| 708 | Wrong outcome | Feasibility evaluation of Smart Stretcher to improve patient safety during transfers | 2011 | Ohashi, K. and Kurihara, Y. and Watanabe, K. and Ohno-Machado, L. and Tanaka, H. and Ohashi, K. and Kurihara, Y. and Watanabe, K. and Ohno-Machado, L. and Tanaka, H. |
| 709 | Wrong outcome | Feasibility of continuous glucose monitoring in critically Ill emergency department patients | 2012 | Lee, J. H. and Kim, K. and Jo, Y. H. and Rhee, J. E. and Lee, J. C. and Kim, K. S. and Kwon, W. Y. and Suh, G. J. and Kim, H. C. and Yoon, H. I. and Park, S. H. |
| 710 | Wrong outcome | Feasibility of continuous monitoring of vital signs in surgical patients on a general ward: an observational cohort study | 2021 | Leenen, J. P. L. and Dijkman, E. M. and van Dijk, J. D. and van Westreenen, H. L. and Kalkman, C. and Schoonhoven, L. and Patijn, G. A. |
| 711 | Wrong outcome | Feasibility of the implementation of a technic of extra-corporeal CO2 removal (ECCO2R) in an intensive care unit which doesnâ€™t use ECMO and its real utilization | 2015 | Amilien, V. and Ponthus, J. P. and Ngasseu, P. and Barsam, E. and Lehericey, P. and Tchir, M. and Bezian, E. and Georger, J. F. |
| 712 | Wrong outcome | A feasibility study to investigate the acceptability and potential effectiveness of a telecare service for older people with chronic obstructive pulmonary disease | 2012 | Chau, J. P. and Lee, D. T. and Yu, D. S. and Chow, A. Y. and Yu, W. C. and Chair, S. Y. and Lai, A. S. and Chick, Y. L. |
| 713 | Wrong outcome | Feasibility study: home telemonitoring for patients with lung cancer in a mountainous rural area | 2014 | Petitte, T. M. and Narsavage, G. L. and Chen, Y. J. and Coole, C. and Forth, T. and Frick, K. D. |
| 714 | Wrong outcome | Feasibility Study: Home Telemonitoring for Patients With Lung Cancer in a Mountainous Rural Area | 2014 | Petitte, Trisha M. and Narsavage, Georgia L. and Chen, Yea-Jyh and Coole, Charles and Forth, Tara and Frick, Kevin D. |
| 715 | Wrong outcome | Feasibility, acceptability, and appropriateness of a mobile health stroke intervention among Ghanaian health workers | 2022 | Amuasi, J. and Agbogbatey, M. K. and Sarfo, F. S. and Beyuo, A. and Duah, K. and Agasiya, P. and Arthur, A. and Appiah, L. and Nguah, S. B. and Bockarie, A. and Ayisi-Boateng, N. K. and Boateng, K. G. A. and Adusei-Mensah, N. and Akpalu, A. and Ovbiagele, B. |
| 716 | Wrong outcome | Features of primary care associated with variations in process and outcome of care of people with diabetes | 2001 | Khunti, K. and Ganguli, S. and Baker, R. and Lowy, A. |
| 717 | Wrong outcome | Femoral Nerve Injury during Neuroborreliosis -- Nursing Care. Case Report | 2022 | Michalak, Monika and Frysiak, GraÅ¼yna and Kazimierska-ZajÄ…c, Magdalena |
| 718 | Wrong outcome | Fever and standard monitoring parameters of ICU patients: a descriptive study | 2007 | Kiekkas, P. and Brokalaki, H. and Manolis, E. and Askotiri, P. and Karga, M. and Baltopoulos, G. I. |
| 719 | Wrong outcome | Fever management audit: Australian nurses' antipyretic usage | 2003 | Edwards, H. E. and Courtney, M. D. and Wilson, J. E. and Monaghan, S. J. and Walsh, A. M. |
| 720 | Wrong outcome | The fifth vital sign: cornerstone of a new pain management strategy | 2002 | Berdine, H. J. |
| 721 | Wrong outcome | Financial and Clinical Impact of Virtual Care During the COVID-19 Pandemic: Difference-in-Differences Analysis | 2023 | Walter, R. J. and Schwab, S. D. and Wilkes, M. and Yourk, D. and Zahradka, N. and Pugmire, J. and Wolfberg, A. and Merritt, A. and Boster, J. and Loudermilk, K. and Hipp, S. J. and Morris, M. J. |
| 722 | Wrong outcome | Findings of a Naloxone Database and its Utilization to Improve Safety and Education in a Tertiary Care Medical Center | 2016 | Rosenfeld, D. M. and Betcher, J. A. and Shah, R. A. and Chang, Y. H. and Cheng, M. R. and Cubillo, E. I. and Griffin, J. M. and Trentman, T. L. |
| 723 | Wrong outcome | First Steps in Improving Blood Pressure Control Among Primary Care Hypertensive Veterans Utilizing Quality Improvement Tools | 2017 | Breaux-Shropshire, Tonya L. and Huie, Rebecca and Shropshire, Toneyell S. and Wyatt, Annette and Shropshire, Angele T. and Estrada, Carlos A. and Patrician, Patricia |
| 724 | Wrong outcome | A five-year audit of nursing observations on ward patients | 2008 | Smith, S. |
| 725 | Wrong outcome | Flexible monitoring in the management of patient care process: a pilot study | 2000 | Banks, J. and McArthur, J. and Gordon, G. |
| 726 | Wrong outcome | A flexible system for vital signs monitoring in hospital general care wards based on the integration of UNIX-based workstations, standard networks and portable vital signs monitors | 1991 | Welch, J. P. and Sims, N. and Ford-Carlton, P. and Moon, J. B. and West, K. and Honore, G. and Colquitt, N. |
| 727 | Wrong outcome | Fluctuations in vital signs and behavioural responses of brain surgery patients in the Intensive Care Unit: are they valid indicators of pain? | 2014 | Kapoustina, O. and Echegaray-Benites, C. and GÃ©linas, C. |
| 728 | Wrong outcome | Fluid optimisation using a peripherally inserted central catheter (PICC) following proximal femoral fracture: lessons learnt from a feasibility study | 2009 | Tutton, E. and Gray, B. |
| 729 | Wrong outcome | Focus on quality. Developing hypothermia indicators | 1996 | Bush, P. and O'Donohue, M. and Black, K. and Cavanagh, E. and Nugent, D. and Rybak, S. and Seabrook, W. |
| 730 | Wrong outcome | Follow-up 90 Days after Stroke and TIA in the Stroke Quality Monitoring Project Rheinland-Pfalz | 2018 | Grau, A. J. and Eicke, M. and Burmeister, C. and Hardt, R. and Schmitt, E. and Dienlin, S. |
| 731 | Wrong outcome | Food intakes in in-hospital patients | 2003 | Zazzo, J. F. |
| 732 | Wrong outcome | For the enquiring mind... Do you know of any new education or research programs that pediatric nurses and technologists are involved in? | 1999 | Korus, M. |
| 733 | Wrong outcome | Forced air warmers: Policies for safe use | 2016 | Deinlein, Amanda D. |
| 734 | Wrong outcome | Free flap monitoring using skin temperature strip indicators: Adjunct to clinical examination | 2008 | Chiu, E. S. and Altman, A. and Allen, R. J. |
| 735 | Wrong outcome | The frequency and timing of respiratory depression in 1524 postoperative patients treated with systemic or neuraxial morphine | 2005 | Shapiro, A. and Zohar, E. and Zaslansky, R. and Hoppenstein, D. and Shabat, S. and Fredman, B. |
| 736 | Wrong outcome | The frequency of errors of blood pressure measurement among nurses in the hospitals affiliated to Shiraz University of Medical Sciences, 2014 | 2015 | Dokoohaki, R. and Raeiskarimian, F. and Rahgosha, A. and Sharifi, M. |
| 737 | Wrong outcome | Frequency of vital sign assessment and clinical deterioration in an Australian emergency department | 2016 | Lambe, Katherine and Currey, Judy and Considine, Julie |
| 738 | Wrong population | Frequency, duration and cause of ventilator alarms on a neonatal intensive care unit | 2018 | Belteki, G. and Morley, C. J. |
| 739 | Wrong outcome | From compute to care: Lessons learned from deploying an early warning system into clinical practice | 2022 | Pou-Prom, C. and Murray, J. and Kuzulugil, S. and Mamdani, M. and Verma, A. A. |
| 740 | Wrong outcome | From Nonadherence to Adherence | 2020 | Kuypers, D. R. J. |
| 741 | Wrong outcome | From nursing care sheets to nursing diagnoses | 2005 | Domingo Pozo, M. and GÃ³mez Robles, F. J. and Torres Figueiras, M. and GabaldÃ³n Bravo, E. M. |
| 742 | Wrong outcome | Front line nurses' experiences with deteriorating ward patients: a qualitative study | 2013 | Chua, W. L. and Mackey, S. and Ng, E. K. and Liaw, S. Y. |
| 743 | Wrong outcome | Functional Dementia Care Unit: organisational innovation for cognitive impairment prevention | 2016 | RodrÃ­guez, Daniel and Rey, Antoni and Vargas, Elena and UreÃ±a, Montse and Civit, Carme and Medarde, Elena |
| 744 | Wrong outcome | A fundamental conflict of care: Nurses' accounts of balancing patients' sleep with taking vital sign observations at night | 2018 | Hope, J. and Recio-Saucedo, A. and Fogg, C. and Griffiths, P. and Smith, G. B. and Westwood, G. and Schmidt, P. E. |
| 745 | Wrong outcome | Further Analysis on Solution Treatment for Diabetes of Patients at Hospitals in Vietnam | 2021 | Binh, V. T. and Huy, D. T. N. |
| 746 | Wrong outcome | [The future patient monitoring in the bed ward] | 2022 | Meyhoff, C. S. and Aasvang, E. K. |
| 747 | Wrong outcome | Fuzzy Guided Autonomous Nursing Robot through Wireless Beacon Network | 2022 | Narayanan, K. L. and Krishnan, R. S. and Son, L. H. and Tung, N. T. and Julie, E. G. and Robinson, Y. H. and Kumar, R. and Gerogiannis, V. C. |
| 748 | Wrong outcome | Gastric outlet obstruction: A case report | 2020 | Ghungrud, D. and Tembhare, V. and Sakharkar, S. and Patil, M. |
| 749 | Foreign Language | Geburtshilfliche Analgesie in deutschen Kliniken. Remifentanil als Alternative zur Regionalanalgesie | 2011 | Schnabel, A. and Hahn, N. and Muellenbach, R. and Frambach, T. and Hoenig, A. and Roewer, N. and Kranke, P. and Schnabel, A. and Hahn, N. and Muellenbach, R. and Frambach, T. and Hoenig, A. and Roewer, N. and Kranke, P. |
| 750 | Wrong outcome | General ward nurses detection and response to clinical deterioration in three hospitals at the Kenyan coast: a convergent parallel mixed methods study | 2024 | Mbuthia, N. and Kagwanja, N. and Ngari, M. and Boga, M. |
| 751 | Wrong outcome | Glucose as the Fifth Vital Sign: A Randomized Controlled Trial of Continuous Glucose Monitoring in a Non-ICU Hospital Setting | 2020 | Fortmann, A. L. and Spierling Bagsic, S. R. and Talavera, L. and Garcia, I. M. and Sandoval, H. and Hottinger, A. and Philis-Tsimikas, A. |
| 752 | Wrong outcome | A good idea badly implemented? Revised National Early Warning Score 2 in community settings | 2019 | Nazarko, Linda |
| 753 | Wrong outcome | A guide to establishing a hyperthermic intraperitoneal chemotherapy program in gynecologic oncology | 2020 | Chambers, L. M. and Costales, A. B. and Crean-Tate, K. and Kuznicki, M. and Morton, M. and Horowitz, M. and Jagielo, T. and Rose, P. G. and Michener, C. and Vargas, R. and Debernardo, R. |
| 754 | Wrong population | Guideline No. 431: Postpartum Hemorrhage and Hemorrhagic Shock | 2022 | Robinson, D. and Basso, M. and Chan, C. and Duckitt, K. and Lett, R. |
| 755 | Foreign Language | Habilidades e competÃªncias da equipe de enfermagem na tÃ©cnica de mensuraÃ§Ã£o da pressÃ£o arterial | 2024 | Fabiana, Rezer and Junior Paulino, Machacal and Wladimir Rodrigues, Faustino |
| 756 | Wrong outcome | The handling of sodium-nitroprusside in high blood pressure emergencies | 2009 | Calil, A. M. and Paranhos, W. Y. |
| 757 | Wrong outcome | Hands-on pulse monitoring sees cardiac arrests cut by two thirds | 2008 | Parish, C. |
| 758 | Wrong outcome | Harnessing Nursing Expertise and LEAN Methodology to Champion Change to Arterial Blood Pressure Monitoring | 2016 | McLellan, Lorna and Grin, Sarah |
| 759 | Background Article | Has not passed urine: but is the bladder full? | 2005 | Carrington, F. |
| 760 | Wrong outcome | Hazardous post anesthesia care unit (PACU): reality or myth? A case study | 1999 | Edwards, M. L. |
| 761 | Wrong outcome | Health care provider outcomes during and shortly after acute care restructuring in Newfoundland and Labrador | 2005 | Way, C. and Gregory, D. and Doyle, M. and Twells, L. and Barrett, B. and Parfrey, P. |
| 762 | Wrong outcome | A health partnership to reduce neonatal mortality in four hospitals in Rwanda | 2017 | Ntigurirwa, P. and Mellor, K. and Langer, D. and Evans, M. and Robertson, E. and Tuyisenge, L. and Groves, A. and Lissauer, T. |
| 763 | Wrong outcome | Health professionals' expectations versus experiences of internet-based telemonitoring: survey among heart failure clinics | 2013 | de Vries, A. E. and van der Wal, M. H. and Nieuwenhuis, M. M. and de Jong, R. M. and van Dijk, R. B. and Jaarsma, T. and Hillege, H. L. |
| 764 | Wrong outcome | Health Services and Delivery Research | 2015 | Benn, J. and Arnold, G. and Dâ€™Lima, D. and Wei, I. and Moore, J. and Aleva, F. and Smith, A. and Bottle, A. and Brett, S. |
| 765 | Wrong outcome | Healthcare Monitoring Based on Cloud Computing: Case Study (Corona Virus) | 2022 | Abdul-Rahaim, L. A. and Ali, S. M. and Mohmmed, T. |
| 766 | Wrong outcome | Healthcare provider's perspectives on home blood pressure management in Peru and Cameroon: Findings from the BPMONITOR study | 2023 | Al-Rousan, T. and Awad, M. and Amalia Pesantes, M. and Kandula, N. R. and Huffman, M. D. and Jaime Miranda, J. and Vidal-Perez, R. and Dzudie, A. and Anderson, C. A. M. |
| 767 | Wrong outcome | Healthcare system barriers and facilitators to hypertension management in Ghana | 2024 | Byiringiro, S. and Hinneh, T. and Chepkorir, J. and Tomiwa, T. and Commodore-Mensah, Y. and Marsteller, J. and Sarfo, F. S. and Saylor, M. A. and Assibey, S. and Himmelfarb, C. R. |
| 768 | Wrong outcome | Healthcare-seeking behaviour and management of type 2 diabetes: From Ugandan traditional healersâ€™ perspective | 2016 | Atwine, F. and Hjelm, K. |
| 769 | Wrong outcome | Heart failure units in Spain: State of the art | 2007 | Zamora, E. and LupÃ³n, J. |
| 770 | Wrong population | HEAT RETENTION HEAD WRAP FOR REWARMING INFANTS UNDERGOING CARDIOPULMONARY BYPASS SURGERY | 2015 | Sakakeeny, Karen H. and Connor, Jean Anne and del Nido, Pedro J. and Odegard, Kirsten and Degrazia, Michele |
| 771 | Wrong outcome | Heliox: a new treatment for life-threatening asthma | 1997 | Browne-Heitschmidt, M. G. and Cassidy, J. B. |
| 772 | Wrong outcome | Help to spot deterioration | 2015 | Lewis, Robin |
| 773 | Wrong outcome | The helpful or hindering effects of in-hospital patient monitor alarms on nurses: a qualitative analysis | 2012 | Varpio, Lara and Kuziemsky, Craig and MacDonald, Charlotte and King, W. James |
| 774 | Background Article | [Hemodynamic Monitoring 2.0 - What is Possible on Normal Wards?] | 2022 | Schiewe, R. and Bein, B. |
| 775 | Wrong outcome | HerzMobil, an Integrated and Collaborative Telemonitoring-Based Disease Management Program for Patients With Heart Failure: A Feasibility Study Paving the Way to Routine Care | 2018 | Ammenwerth, E. and Modre-Osprian, R. and Fetz, B. and Gstrein, S. and Krestan, S. and DÃ¶rler, J. and Kastner, P. and Welte, S. and Rissbacher, C. and PÃ¶lzl, G. |
| 776 | Wrong outcome | Hidden Obligatory Fluid Intake in Critical Care Patients | 2017 | Bashir, M. U. and Tawil, A. and Mani, V. R. and Farooq, U. and M, A. DeVita |
| 777 | Wrong outcome | High blood pressure control program - Educational sessions: Part I | 1984 | Marnalse, R. and Bohanek, B. and Kopp, C. |
| 778 | Wrong outcome | High-altitude mountain telemedicine | 2022 | Martinelli, M. and Moroni, D. and Bastiani, L. and Mrakic-Sposta, S. and Giardini, G. and Pratali, L. |
| 779 | Wrong population | The High-Risk Postpartum Transitional Program: A Multidisciplinary Approach to Caring for Postpartum Woman With Hypertensive Disease | 2014 | Bittle, Marianne D. and Scalise, Laura and Green, Dana and Srinivas, Sindhu and Hirshberg, Adi and Chandrasekaran, Suchitra |
| 780 | Foreign Language | HIPERTENSCO ARTERIAL EM PROFISSIONAIS QUE ATUAM EM SERVIEOS DE ATENDIMENTO PRO-HOSPITALAR | 2011 | Cesira Cavagioni, Luciane and Geraldo Pierin, Angela Maria |
| 781 | Wrong outcome | A home based intervention reduced the frequency of hospital readmissions and out of hospital deaths after discharge [commentary on Stewart S, Pearson S, Luke CG, et al. Effects of home-based intervention on unplanned readmissions and out-of-hospital deaths. J AM GERIATR SOC 1998 Feb;46:174-80] | 1998 | Shuster, G. F. |
| 782 | Wrong outcome | Home blood pressure telemonitoring in the 21st century | 2018 | Parati, G. and Dolan, E. and McManus, R. J. and Omboni, S. |
| 783 | Wrong outcome | Home care by outreach nursing for chronic obstructive pulmonary disease | 2001 | Smith, B. and Appleton, S. and Adams, R. and Southcott, A. and Ruffin, R. |
| 784 | Wrong outcome | Home intravenous antibiotherapy and the proper use of elastomeric pumps: Systematic review of the literature and proposals for improved use | 2021 | Diamantis, S. and Dawudi, Y. and Cassard, B. and Longuet, P. and Lesprit, P. and Gauzit, R. |
| 785 | Wrong outcome | Home monitoring program reduces interstage mortality after the modified Norwood procedure | 2014 | Siehr, S. L. and Norris, J. K. and Bushnell, J. A. and Ramamoorthy, C. and Reddy, V. M. and Hanley, F. L. and Wright, G. E. |
| 786 | Wrong outcome | Home monitoring reduced short stay admissions in suspected COVID-19 patients: COVID-box project | 2021 | Dirikgil, E. and Roos, R. and Groeneveld, G. H. and Heringhaus, C. and Silven, A. V. and Petrus, A. H. J. and Villalobos-Quesada, M. and Tsonaka, R. and van der Boog, P. J. M. and Rabelink, T. J. and Bos, W. J. W. and Chavannes, N. H. and Atsma, D. E. and Teng, Y. K. O. |
| 787 | Wrong outcome | Home telehealth for patients with chronic obstructive pulmonary disease (COPD): an evidence-based analysis | 2012 | Franek, J. |
| 788 | Wrong outcome | Home telehealth reduces healthcare costs | 2004 | Noel, H. C. and Vogel, D. C. and Erdos, J. J. and Cornwall, D. and Levin, F. |
| 789 | Wrong outcome | Home telehealthcare: findings from a pilot study in North-east London | 2006 | Procter, S. and Single, A. |
| 790 | Wrong outcome | Home-based initiatives for acute management of COVID-19 patients needing oxygen: differences across The Netherlands | 2023 | Boeijen, J. A. and van de Pol, A. C. and van Uum, R. T. and Smit, K. and Ahmad, A. and van Rijswijk, E. and van Apeldoorn, M. J. and van Thiel, E. and de Graaf, N. and Menkveld, R. M. and Mantingh, M. R. and Geertman, S. and Couzijn, N. and van Groenendael, L. and Schers, H. and Bont, J. and Bonten, T. N. and Rutten, F. H. and Zwart, D. L. M. |
| 791 | Wrong outcome | [Home-based telemonitoring of simple vital signs to reduce hospitalization in heart failure patients: real-world data from a community-based hospital] | 2011 | Palmieri, V. and Pezzullo, S. and Lubrano, V. and Bettella, S. and Olandese, M. and Sorrentino, C. and Russo, C. and Celentano, A. |
| 792 | Wrong outcome | Home-based telesurveillance and rehabilitation after stroke: a real-life study | 2016 | Bernocchi, P. and Vanoglio, F. and Baratti, D. and Morini, R. and Rocchi, S. and Luisa, A. and Scalvini, S. |
| 793 | Wrong outcome | Homecare safety and medication management: a scoping review of the quantitative and qualitative evidence | 2013 | Godfrey, Christina M. and Harrison, Margaret B. and Lang, Ariella and Macdonald, Marilyn and Leung, Tina and Swab, Michelle |
| 794 | Wrong outcome | Hong Kong general ward nurses' experiences of transitional care for patients discharged from the intensive care unit: An inductive thematic analysis | 2023 | Yau, Y. C. and Christensen, M. |
| 795 | Wrong outcome | Hospital acquired infections. I. Surveillance in a university hospital | 1976 | Wenzel, R. P. and Osterman, C. A. and Hunting andGwaltney Jr, K. J. J. M. |
| 796 | Wrong outcome | Hospital Epidemics Tracker (HEpiTracker): Description and pilot study of a mobile app to track COVID-19 in hospital workers | 2020 | Soriano, J. B. and FernÃ¡ndez, E. and de Astorza, Ã and PÃ©rez de Llano, L. A. and FernÃ¡ndez-Villar, A. and Carnicer-Pont, D. and AlcÃ¡zar-Navarrete, B. and GarcÃ­a, A. and Morales, A. and Lobo, M. and Maroto, M. and Ferreras, E. and Soriano, C. and Del Rio-Bermudez, C. and Vega-Piris, L. and BasagaÃ±a, X. and Muncunill, J. and Cosio, B. G. and Lumbreras, S. and Catalina, C. and Alzaga, J. M. and GÃ³mez QuilÃ³n, D. and Valdivia, C. A. and de Lara, C. and Ancochea, J. |
| 797 | Wrong outcome | Hospital sphygmomanometer use: an audit | 1995 | Carney, S. L. and Gillies, A. H. and Smith, A. J. and Smitham, S. |
| 798 | Wrong outcome | Hospital-acquired infections. I. Surveillance in a university hospital | 1976 | Wenzel, R. P. and Osterman, C. A. and Hunting, K. J. and Gwaltney, J. M., Jr. |
| 799 | Wrong outcome | Hospital-Based Electrocardiographic Monitoring: The Good, the Not So Good, and Untapped Potential | 2024 | Pelter, Michele M. |
| 800 | Wrong outcome | Hospital-based intervention to enhance hypertension diagnosis in Kalasin hospital, Thailand, 2017-2019: A pre-post pilot intervention study | 2020 | Yueayai, K. and Moran, A. E. and Pratipanwat, P. and Chaisongkram, S. and Anosri, L. and Thitichai, P. |
| 801 | Wrong outcome | Hospital-Level Care at Home for Patients With Acute Respiratory Disease: A Descriptive Analysis | 2023 | Hernandez, C. and Tukpah, A. C. and Mitchell, H. M. and Rosario, N. A. and Boxer, R. B. and Morris, C. A. and Schnipper, J. L. and Levine, D. M. |
| 802 | Wrong outcome | Hospital-Level Care at Home for Patients With Acute Respiratory Disease: A Descriptive Analysis | 2023 | Hernandez, C. and Tukpah, A. M. C. and Mitchell, H. M. and Rosario, N. A. and Boxer, R. B. and Morris, C. A. and Schnipper, J. L. and Levine, D. M. |
| 803 | Wrong outcome | Hospitalization and suicide | 2010 | Nimmagadda, Janaki and Farooqui, Azra and Mohyuddin, Farooq and Postolache, Teodor T. |
| 804 | Wrong outcome | Hospitalization reduction by an asthma tele-medicine system | 2000 | Kokubu, F. and Nakajima, S. and Ito, K. and Makino, S. and Kitamura, S. and Fukuchi, Y. and Mano, K. and Sano, Y. and Inoue, H. and Morita, Y. and Fukuda, K. and Akiyama, K. and Adachi, M. and Miyamoto, T. |
| 805 | Wrong outcome | Hot water immersion therapy with a thermal isolator in patient with marine envenomation | 2011 | Lau, K. K. and Chan, C. K. and Tse, M. L. and Lau, F. L. |
| 806 | Wrong outcome | How a University Hospital improves its prevention performance: Results of two biennial studies 2004-2006 | 2010 | Blanquet, M. and Grondin, M. A. and Noirfalise, C. and Gerbaud, L. |
| 807 | Wrong outcome | How do nurses use early warning system vital signs observation charts in rural, remote and regional health care facilities: A scoping review | 2023 | Augutis, Wendy and Flenady, Tracy and LeÂ Lagadec, Danielle and Jefford, Elaine |
| 808 | Wrong outcome | How do nurses use the early warning score in their practice? A case study from an acute medical unit | 2019 | Foley, Claire and Dowling, Maura |
| 809 | Wrong outcome | How helpful are early warning scores? | 2014 | Carberry, Martin |
| 810 | Wrong outcome | How is norepinephrine used in intensive care? A field study | 2007 | Blazejewski, S. and Raymond, N. and Lagnaoui, R. and Winnock, S. and Cochard, J. F. and Petit, L. and Dosque, J. P. and Fillatreau, C. and Moore, N. |
| 811 | Wrong outcome | How long do nurses take to measure patientsâ€™ vital signs? | 2022 | Dallâ€™Ora, Chiara and Saville, Christina |
| 812 | Wrong outcome | How Much do We Know About Pulse Oximeters Used in Every Field from Home Care to Critical Care? A Descriptive Study | 2023 | Keti, E. P. and DinÃ§er, N. Ãœ |
| 813 | Wrong outcome | How registered nurses are measuring respiratory rates in adult acute care health settings: An integrative review | 2023 | Palmer, J. H. and James, S. and Wadsworth, D. and Gordon, C. J. and Craft, J. |
| 814 | Wrong outcome | How reliable are self-assessments using mobile technology in healthcare? The effects of technology identity and self-efficacy | 2019 | Reychav, Iris and Beeri, Roni and Balapour, Ali and Raban, Daphne Ruth and Sabherwal, Rajiv and Azuri, Joseph |
| 815 | Wrong outcome | How should we measure blood pressure in the doctor's office? | 2001 | Gerin, W. and Marion, R. M. and Friedman, R. and James, G. D. and Bovbjerg, D. H. and Pickering, T. G. |
| 816 | Wrong outcome | How to avoid catastrophic events on the ward | 2016 | Bein, B. and Seewald, S. and GrÃ¤sner, J. T. |
| 817 | Wrong outcome | [How to implement a complete apheresis program within a hemodialysis unit] | 2019 | Maurizi-Balzan, J. and Jouve, T. and Naciri-Bennani, H. and Noble, J. and Tanoukhi, K. and Motte, L. and Malvezzi, P. and Rostaing, L. |
| 818 | Wrong outcome | How to measure and record vital signs to ensure detection of deteriorating patients | 2009 | Boulanger, C. and Toghill, M. |
| 819 | Wrong outcome | How visiting nurses detect symptoms of disease progression in patients with chronic heart failure | 2020 | Taniguchi, C. and Okada, A. and Seto, N. and Shimizu, Y. |
| 820 | Wrong outcome | How Well Are Pulses Measured? Practice-Based Evidence from an Observational Study of Acutely Ill Medical Patients During Hospital Admission | 2017 | Opio, M. O. and Kellett, J. |
| 821 | Wrong outcome | How Well Do Neurochecks Perform After Stroke? | 2021 | De Leon Benedetti, A. M. and Bhatia, R. and Ancheta, S. R. and Romano, J. G. and Koch, S. |
| 822 | Wrong outcome | Human factors approach to evaluate the user interface of physiologic monitoring | 2015 | Fidler, R. and Bond, R. and Finlay, D. and Guldenring, D. and Gallagher, A. and Pelter, M. and Drew, B. and Hu, X. |
| 823 | Wrong outcome | Hypertension healthcare professional beliefs and behaviour regarding patient medication adherence: a survey conducted among European Society of Hypertension Centres of Excellence | 2021 | Burnier, M. and Prejbisz, A. and Weber, T. and Azizi, M. and Cunha, V. and Versmissen, J. and Gupta, P. and Vaclavik, J. and Januszewicz, A. and Persu, A. and Kreutz, R. |
| 824 | Wrong outcome | Hypertension in professionals working in prehospital care services | 2011 | Cavagioni, Luciane Cesira and Pierin, Angela Maria Geraldo |
| 825 | Wrong population | Hypertensive disorders of pregnancy: Maternal profile and role of the midwife in the department of health of gandia | 2019 | SÃ¡nchez-LÃ³pez, M. I. and Llabata-Carabal, P. and GarrigÃ³s-Almerich, E. |
| 826 | Wrong outcome | Hypertensive Emergencies: Common Presentations and Pharmacological Interventions | 2023 | El Hussein, M. T. and Dolynny, A. |
| 827 | Wrong population | Hypothermia detection in low birth weight neonates using a novel bracelet device | 2019 | Tanigasalam, Vasanthan and Vishnu Bhat, B. and Adhisivam, B. and Balachander, Bharathi and Kumar, Harichandra |
| 828 | Wrong population | Iatrogenic Hypermagnesemia in a Patient With Preeclampsia Caused by Misinterpretation of the Magnesium Reporting Unit Following Magnesium Sulfate Administration | 2022 | Omer, M. S. and Latif, S. and Grisson, R. |
| 829 | Wrong outcome | Iatrogenic severe hypoglycemia in a non-diabetic patient under general anesthesia: Case report | 2019 | Khorasanizadeh, S. and Behnaz, F. and Dehkordy, M. E. and Teymourian, H. and Kouzekanani, H. |
| 830 | Wrong outcome | The ICEBERG: A score and visual representation to track the severity of traumatic brain injury: Design principles and preliminary results | 2022 | VallÃ©e, Fabrice and Nougue, HÃ©lÃ¨ne and Cartailler, JÃ©rome and KoundÃ©, Paul Robert and Mebazaa, Alexandre and Gayat, Etienne and Azouvi, Philippe and Mateo, Joaquim |
| 831 | Wrong outcome | Ideal Site for Skin Temperature Probe Placement on Infants in the NICU: A Review of Literature | 2017 | Joseph, R. A. and Derstine, S. and Killian, M. |
| 832 | Wrong outcome | Identification of Predictors for Clinical Deterioration in Patients with COVID-19 via Electronic Nursing Records: Retrospective Observational Study | 2024 | Sung, S. and Kim, Y. and Kim, S. H. and Jung, H. |
| 833 | Wrong outcome | Identification of thresholds for accuracy comparisons of heart rate and respiratory rate in neonates | 2021 | Coleman, J. and Ginsburg, A. S. and Macharia, W. M. and Ochieng, R. and Zhou, G. and Dunsmuir, D. and Karlen, W. and Ansermino, J. M. |
| 834 | Wrong outcome | Identification of warning signs for prevention of in-hospital cardiorespiratory arrest | 2019 | Tessorolo Souza, Beatriz and Barbosa Teixeira Lopes, Maria Carolina and Pinto Okuno, Meiry Fernanda and Assayag Batista, Ruth Ester and Teixeira de GÃ³is, AÃ©cio FlÃ¡vio and Vancini Campanharo, CÃ¡ssia Regina |
| 835 | Wrong outcome | The illness severity of ward remaining patients reviewed by the medical emergency team: A retrospective cohort study | 2023 | Batterbury, A. and Douglas, C. and Coyer, F. |
| 836 | Wrong outcome | The impact of a clinical information system in an intensive care unit | 2008 | Donati, A. and Gabbanelli, V. and Pantanetti, S. and Carletti, P. and Principi, T. and Marini, B. and Nataloni, S. and Sambo, G. and Pelaia, P. and Donati, Abele and Gabbanelli, Vincenzo and Pantanetti, Simona and Carletti, Paola and Principi, Tiziana and Marini, Benedetto and Nataloni, Simonetta and Sambo, Gisella and Pelaia, Paolo |
| 837 | Wrong outcome | The Impact of a Machine Learning Early Warning Score on Hospital Mortality: A Multicenter Clinical Intervention Trial | 2022 | Winslow, C. J. and Edelson, D. P. and Churpek, M. M. and Taneja, M. and Shah, N. S. and Datta, A. and Wang, C. H. and Ravichandran, U. and McNulty, P. and Kharasch, M. and Halasyamani, L. K. |
| 838 | Wrong outcome | Impact of a modified early warning score on nurses' recognition and response to clinical deterioration | 2021 | Warren, Talecia and Moore, Leslie C. and Roberts, Sterling and Darby, Laura |
| 839 | Wrong outcome | Impact of a telemedicine system on work burden and mental health of healthcare providers working with COVID-19: a multicenter pre-post prospective study | 2022 | Kagiyama, N. and Komatsu, T. and Nishikawa, M. and Hiki, M. and Kobayashi, M. and Matsuzawa, W. and Daida, H. and Minamino, T. and Naito, T. and Sugita, M. and Miyazaki, K. and Anan, H. and Kasai, T. |
| 840 | Wrong outcome | The impact of a Web-based educational program on the recognition and management of deteriorating patients | 2017 | Liaw, S. Y. and Chng, D. Y. J. and Wong, L. F. and Ho, J. T. Y. and Mordiffi, S. Z. and Cooper, S. and Chua, W. L. and Ang, E. N. K. |
| 841 | Wrong outcome | The impact of an admission unit on failure- and late-to-rescue rates in the emergency department | 2007 | Koran, Z. |
| 842 | Wrong outcome | The Impact of an Electronic Patient Bedside Observation and Handover System on Clinical Practice: Mixed-Methods Evaluation | 2019 | Lang, A. and Simmonds, M. and Pinchin, J. and Sharples, S. and Dunn, L. and Clarke, S. and Bennett, O. and Wood, S. and Swinscoe, C. |
| 843 | Wrong population | The impact of an intervention to improve intrapartum maternal vital sign monitoring and reduce alarm fatigue | 2023 | Kern-Goldberger, A. R. and Nicholls, E. M. and Plastino, N. and Srinivas, S. K. |
| 844 | Wrong population | The impact of capnography monitoring among children and adolescents in the postanesthesia care unit: a randomized controlled trial | 2017 | Langhan, M. L. and Li, F. Y. and Lichtor, J. L. |
| 845 | Wrong outcome | Impact of clinical alarms on patient safety from nursesâ€™ perspective | 2022 | Alsuyayfi, S. and Alanazi, A. |
| 846 | Wrong outcome | The Impact of Comprehensive Nursing and Warming Measures on Emergence Agitation and Maternal-Neonatal Safety in Women Undergoing General Anesthesia for Cesarean Section | 2024 | Zhuang, H. and Li, W. and Xue, X. and Wang, H. and Li, S. and Zhong, Y. |
| 847 | Wrong outcome | The impact of continuous versus intermittent vital signs monitoring in hospitals: A systematic review and narrative synthesis | 2018 | Downey, C. L. and Chapman, S. and Randell, R. and Brown, J. M. and Jayne, D. G. |
| 848 | Wrong outcome | Impact of different intervention models on adherence to secondary prevention therapies in patients with acute coronary syndrome | 2018 | Liu, J. and Wang, W. and Liu, J. and Wang, Y. and Qi, Y. and Sun, J. and Zhao, D. |
| 849 | Wrong outcome | Impact of foot reflexology massage on the patientsâ€™ physiological indicators without trauma with loss of consciousness in the intensive care unit | 2017 | Sheikh, S. and Yaghoubinia, F. and Navidian, A. |
| 850 | Wrong outcome | Impact of goal-directed perioperative fluid management in high-risk surgical procedures: a literature review | 2013 | Trinooson, C. D. and Gold, M. E. |
| 851 | Wrong outcome | The Impact of Head-Worn Displays on Strategic Alarm Management and Situation Awareness | 2019 | Pascale, Michael T. and Sanderson, Penelope and Liu, David and Mohamed, Ismail and Brecknell, Birgit and Loeb, Robert G. |
| 852 | Wrong outcome | Impact of health technology assessments. Some experiences of SBU | 2002 | Britton, M. and Jonsson, E. |
| 853 | Wrong outcome | Impact of home-based monitoring on the care of patients with congestive heart failure | 2006 | Myers, S. and Grant, R. W. and Lugn, N. E. and Holbert, B. and Kvedar, J. C. |
| 854 | Wrong outcome | The impact of interprofessional collaboration on nursesâ€™ satisfaction and comfort with intranasal fentanyl | 2013 | Moadebi, Susanne and Kwan, Fiona and Stackhouse, Sherry and Reddekopp, Lisa |
| 855 | Wrong outcome | Impact of nursing education and a monitoring tool on outcomes in traumatic brain injury | 2020 | Gamble, M. and Luggya, T. S. and Mabweijano, J. and Nabulime, J. and Mowafi, H. |
| 856 | Wrong outcome | The Impact of Nursing Interventions on the Treatment Outcomes of Renal Cell Carcinoma with Postoperative Interleukin-2 and Recombinant Human Interferon | 2024 | Guo, L. and Zhao, L. Q. and Chen, J. Y. and Li, M. |
| 857 | Wrong outcome | Impact of Nursing Interventions Targeting Vital Signs and Complication Risk on Perioperative Parameters and Complication Rates in Aortic Dissection Patients | 2024 | Su, H. and Guo, H. and Zhang, Q. and Sun, L. and Zhang, L. |
| 858 | Wrong population | The impact of paediatric early warning systems | 2012 | Naddy, Catherine |
| 859 | Wrong outcome | The impact of preoperative interview and prospective nursing on perioperative psychological stress and postoperative complications in patients undergoing TACE intervention for hepatocellular carcinoma | 2024 | Gao, L. and Chen, W. and Qin, S. and Yang, X. |
| 860 | Wrong outcome | Impact of professionalism in nursing on in-hospital bedside monitoring practice | 2013 | Bunkenborg, G. and Samuelson, K. and Akeson, J. and Poulsen, I. |
| 861 | Wrong outcome | The impact of public hospitals' privatization on nurse staffing | 2021 | Ramamonjiarivelo, Zo and Hearld, Larry and Weech-Maldonado, Robert |
| 862 | Wrong outcome | Impact of pulse oximetry surveillance on rescue events and intensive care unit transfers: A before-and-after concurrence study | 2010 | Taenzer, A. H. and Pyke, J. B. and McGrath, S. P. and Blike, G. T. |
| 863 | Wrong outcome | Impact of Remote Patient Monitoring on Length of Stay for Patients with COVID-19 | 2023 | Patel, H. and Hassell, A. and Keniston, A. and Davis, C. |
| 864 | Wrong outcome | The impact of surgical ward nurses practising respiratory assessment on positive patient outcomes | 2007 | Duff, B. and Gardiner, G. and Barnes, M. |
| 865 | Wrong outcome | [Impact of the Covid-19 pandemic on the health of nurses in a university hospital] | 2021 | Lucas, D. and Brient, S. and Eveillard, B. M. and Gressier, A. and LeGrand, T. and Dewitte, J. D. and LoddÃ©, B. and Pougnet, R. |
| 866 | Wrong outcome | The impact of the medical emergency team on the resuscitation practice of critical care nurses | 2011 | Santiano, N. and Young, L. and Baramy, L. S. and Cabrera, R. and May, E. and Wegener, R. and Butt, D. and Parr, M. |
| 867 | Wrong outcome | The impact of the medical emergency team on the resuscitation practice of critical care nurses | 2011 | Santiano, N. and Young, L. and Baramy, L. S. and Cabrera, R. and May, E. and Wegener, R. and Butt, D. and Parr, M. and Armstrong, K. and Brillante, V. and Brennan, K. and Chan, C. and Clare, M. and Curley, L. and Hedges, S. and Murphy, J. and Nicholson, M. and Parker, S. and Shunker, S. A. and Stevenson, J. and Twadell, N. and Sanchez, D. and Sutevski, V. and Quach, M. and Whelan, M. |
| 868 | Wrong outcome | Impact of the new generation coronary care unit | 1976 | O'Rourke, M. F. and Walsh, B. and Fletcher, M. and Crowley, A. |
| 869 | Wrong outcome | Impact of using data from electronic protocols in nursing performance management: A qualitative interview study | 2019 | Hope, J. and Griffiths, P. and Schmidt, P. E. and Recio-Saucedo, A. and Smith, G. B. |
| 870 | Wrong outcome | Impact of using data from electronic protocols in nursing performance management: A qualitative interview study | 2019 | Hope, Joanna and Griffiths, Peter and Schmidt, Paul E. and Recioâ€Saucedo, Alejandra and Smith, Gary B. |
| 871 | Wrong outcome | Impact of wearable wireless continuous vital sign monitoring in abdominal surgical patients: before-after study | 2024 | Leenen, J. P. L. and Ardesch, V. and Kalkman, C. J. and Schoonhoven, L. and Patijn, G. A. |
| 872 | Wrong outcome | The impact of web-based and face-to-face simulation on patient deterioration and patient safety: protocol for a multi-site multi-method design | 2016 | Cooper, Simon J. and Kinsman, Leigh and Chung, Catherine and Cant, Robyn and Boyle, Jayne and Bull, Loretta and Cameron, Amanda and Connell, Cliff and Kim, Jeong-Ah and McInnes, Denise and McKay, Angela and Nankervis, Katrina and Penz, Erika and Rotter, Thomas |
| 873 | Wrong outcome | Impedance cardiography the next vital sign technology? | 2003 | Van De Water, J. M. and Miller, T. W. and Vogel, R. L. and Mount, B. E. and Dalton, M. L. |
| 874 | Wrong outcome | Impedance cardiography: the next vital sign technology? | 2003 | Van De Water, J. M. and Miller, T. W. and Vogel, R. L. and Mount, B. E. and Dalton, M. L. and Van De Water, Joseph M. and Miller, Timothy W. and Vogel, Robert L. and Mount, Bruce E. and Dalton, Martin L. |
| 875 | Wrong outcome | Imperfect implementation of an early warning scoring system in a Danish teaching hospital: a cross-sectional study | 2013 | Niegsch, M. and Fabritius, M. L. and AnhÃ¸j, J. |
| 876 | Wrong population | Implementation and evaluation of obstetric early warning systems in tertiary care hospitals in Nigeria | 2022 | Umar, A. and Ibrahim, S. and Liman, I. and Chama, C. and Ijaiya, M. and Mathai, M. and Ameh, C. |
| 877 | Wrong outcome | Implementation and impact on length of stay of a post-discharge remote patient monitoring program for acutely hospitalized COVID-19 pneumonia patients | 2022 | Kuo, S. and Aledia, A. and O'Connell, R. and Rudkin, S. and Dangodara, A. A. and Amin, A. N. |
| 878 | Wrong outcome | Implementation and outcomes of a pharmacist-managed clinical video telehealth anticoagulation clinic | 2015 | Singh, L. G. and Accursi, M. and Korch Black, K. |
| 879 | Wrong outcome | Implementation of a flexible visiting policy in intensive care unit: A randomized clinical trial | 2020 | Akbari, Reihane and Karimi Moonaghi, Hossein and Mazloum, Seyed Reza and Bagheri Moghaddam, Ahmad |
| 880 | Wrong population | Implementation of a Follow-Up System for Pediatric Sepsis Survivors in a Large Academic Pediatric Intensive Care Unit | 2021 | Fitzgerald, J. C. and Kelly, N. A. and Hickey, C. and Balamuth, F. and Thomas, N. H. and Hogan, A. and Stack, N. J. and Trimarchi, T. and Weiss, S. L. |
| 881 | Wrong outcome | IMPLEMENTATION OF A NURSE DRIVEN MOBILITY PROGRAM: PREVENTING FALLS AND FUNCTIONAL DECLINE IN ONCOLOGY...47th Annual Oncology Nursing Society Congress, April 27â€“May 1, 2022, Anaheim, CA | 2022 | Payne, Michelle |
| 882 | Wrong outcome | [Implementation of a patient data management system. Effects on intensive care documentation] | 2013 | Castellanos, I. and Ganslandt, T. and Prokosch, H. U. and SchÃ¼ttler, J. and BÃ¼rkle, T. |
| 883 | Wrong population | Implementation of a Pediatric Orthopaedic Bundle to Reduce Surgical Site Infections | 2017 | Schriefer, Jan and Sanders, James and Michels, Julie and Wolcott, Kori and Ruddy, Connor and Hanson, Jenna |
| 884 | Wrong outcome | Implementation of a pharmacist-managed heart failure medication titration clinic | 2013 | Martinez, Amanda S. and Saef, Jerold and Paszczuk, Anna and Bhatt-Chugani, Hetal |
| 885 | Wrong outcome | The implementation of a real time early warning system using machine learning in an Australian hospital to improve patient outcomes | 2023 | Bassin, L. and Raubenheimer, J. and Bell, D. |
| 886 | Wrong outcome | IMPLEMENTATION OF A SAFE AND FEASIBLE MOBILIZATION PROTOCOL FOR PATIENTS POD 0 TRANSCATHETER CARDIAC VALVE PROCEDURES | 2018 | J, Ward and Z, Hernandez and D, Freund |
| 887 | Wrong outcome | Implementation of a universal postpartum blood pressure monitoring program: feasibility and outcomes | 2022 | Hacker, F. M. and Jeyabalan, A. and Quinn, B. and Hauspurg, A. |
| 888 | Wrong outcome | Implementation of an easy-to-follow standardized chart for free flaps clinical monitoring during the start of a head and neck microsurgery unit in a tertiary hospital | 2019 | MegÃ­as Barrera, J. and Gorina Faz, M. and Artajona GarcÃ­a, M. and Costa Garcia, A. and Navarrete PiÃ±ero, M. |
| 889 | Wrong outcome | Implementation of an interprofessional model for the management of postpartum hypertension | 2024 | Safri, A. A. and Kopcza, B. T. and Kaplon, S. C. and Norman, K. E. and Brien, K. O. and Falinski, J. P. and Brien, M. E. O. and Yarrington, C. D. |
| 890 | Wrong outcome | Implementation of continuous temperature monitoring during perioperative care: a feasibility study | 2022 | Munday, J. and Sturgess, D. and Oishi, S. and Bendeich, J. and Kearney, A. and Douglas, C. |
| 891 | Wrong outcome | Implementation of Electronic Medical Record in Vascular and Interventional Radiology | 2019 | Othman, K. and Bashir, O. and Arabi, M. |
| 892 | Wrong outcome | Implementation of foot thermometry plus mHealth to prevent diabetic foot ulcers: study protocol for a randomized controlled trial | 2016 | Lazo-Porras, M. and Bernabe-Ortiz, A. and Sacksteder, K. A. and Gilman, R. H. and Malaga, G. and Armstrong, D. G. and Miranda, J. J. |
| 893 | Wrong outcome | Implementation of quality measures to reduce surgical site infection in colorectal patients | 2008 | Wick, E. C. and Gibbs, L. and Indorf, L. A. and Varma, M. G. and Garcia-Aguilar, J. |
| 894 | Wrong outcome | The Implementation of Targeted Temperature Management: An Evidence-Based Guideline from the Neurocritical Care Society | 2017 | Madden, L. K. and Hill, M. and May, T. L. and Human, T. and Guanci, M. M. and Jacobi, J. and Moreda, M. V. and Badjatia, N. |
| 895 | Wrong outcome | Implementing a Goal-Directed Care Bundle after Acute Intracerebral Haemorrhage: Process Evaluation for the Third INTEnsive Care Bundle with Blood Pressure Reduction in Acute Cerebral Haemorrhage Trial Study in China | 2022 | Ouyang, M. and Anderson, C. S. and Song, L. and Jan, S. and Sun, L. and Cheng, G. and Chu, H. and Hu, X. and Ma, L. and Chen, X. and You, C. and Liu, H. |
| 896 | Wrong outcome | Implementing a Protocol to Improve Self-Care Behaviors in Adult Patients With Heart Failure | 2018 | Capilouto, Sari and Brewer, Erica M. and Crawford, Wynne |
| 897 | Wrong outcome | Implementing a screening algorithm for early recognition of sepsis in hospitalized children: a quality improvement project | 2022 | Feinstein, Y. and Kogan, S. and Dreiher, J. and Noham, A. and Harosh, S. and Lecht, J. and Sror, T. and Cohen, N. and Bar-Yosef, E. and Hershkowitz, E. and Lazar, I. and Schonmann, Y. and Greenberg, D. and Danino, D. |
| 898 | Wrong outcome | Implementing Accelerated Rehabilitation Nursing to Facilitate Intestinal Function Recovery Following Cytoreductive Surgery for Gynecological Ovarian Cancer | 2024 | Shen, Q. and Jiang, T. |
| 899 | Wrong outcome | Implementing the National Early Warning Score 2 into pre-registration nurse education | 2020 | Butler, Z. A. |
| 900 | Wrong outcome | Implementing the Patient Safety First intervention to reduce harm from deterioration | 2009 | Beamish, S. and Wardell, D. |
| 901 | Wrong outcome | Implementing Triage-Bot: Supporting the Current Practice for Triage Nurses | 2024 | Sears, K. and Belbin, S. and Rashno, E. and Sharma, D. and Woo, K. and Zulkernine, F. and Neagu, C. D. and Amani, B. and Alaca, F. |
| 902 | Wrong outcome | Improved clinical management but not patient outcome in women with postpartum haemorrhage-An observational study of practical obstetric team training | 2018 | BaldvinsdÃ³ttir, T. and Blomberg, M. and Lilliecreutz, C. |
| 903 | Wrong outcome | Improved Practices for Safe Administration of Intravenous Bolus Morphine in a Pediatric Setting | 2011 | Ellis, Jacqueline and Martelli, Brenda and Lamontagne, Christine and Pascuet, Elena and Taillefer, Louise and Gaboury, Isabelle and Vaillancourt, RÃ©gis |
| 904 | Wrong outcome | Improvements in patient monitoring in the intensive care unit: Survey study | 2020 | Poncette, A. S. and Mosch, L. and Spies, C. and Schmieding, M. and SchiefenhÃ¶vel, F. and Krampe, H. and Balzer, F. |
| 905 | Wrong outcome | Improving detection of patient deterioration in the general hospital ward environment | 2018 | Vincent, J. L. and Einav, S. and Pearse, R. and Jaber, S. and Kranke, P. and Overdyk, F. J. and Whitaker, D. K. and Gordo, F. and Dahan, A. and Hoeft, A. |
| 906 | Wrong outcome | Improving detection of patient deterioration in the general hospital ward environment | 2018 | Vincent, Jean-Louis and Einav, Sharon and Pearse, Rupert and Jaber, Samir and Kranke, Peter and Overdyk, Frank J. and Whitaker, David K. and Gordo, Federico and Dahan, Albert and Hoeft, Andreas |
| 907 | Wrong outcome | Improving diagnosis of early complications (<1Â week) through continuous vital sign monitoring following oncological gastrointestinal surgical procedures | 2024 | Lockhorst, E. W. and van Noordenne, M. and Klouwens, L. and Govaert, K. M. and de Bruijn, E. and Verhoef, C. and Gobardhan, P. D. and Schreinemakers, J. M. J. |
| 908 | Wrong outcome | Improving Electronic Patient Portal Use in Groups with Low Utilization at a Family Medicine Practice | 2022 | Saif, Nadia and Blackstone, Sarah R. and Sebring, Amanda and Compton, Rebekah |
| 909 | Wrong outcome | Improving heart failure care and guideline-directed medical therapy through proactive remote patient monitoring-home telehealth and pharmacy integration | 2022 | Lynch, K. A. and Ganz, D. A. and Saliba, D. and Chang, D. S. and De Peralta, S. S. |
| 910 | Wrong outcome | Improving integrated care for frail elderly patients throught ict - the veneto region experience | 2017 | Gris, Stefano and Sampognaro, Erika and Forestiero, Antonella and Saccavini, Claudio and Mancin, Silvia |
| 911 | Wrong population | Improving Long-Term Sustainability for a Pediatric Acute Care Emergency Event Debriefing Process | 2023 | Bohman, Annie and Hanks, Jacqueline and Carr, Amanda |
| 912 | Wrong outcome | IMPROVING NURSE RESPONSIVENESS ON INPATIENT ONCOLOGY UNITS...47th Annual Oncology Nursing Society Congress, April 27â€“May 1, 2022, Anaheim, CA | 2022 | Lutz, Brianna and Crouch, Laurie and Sanchis, Desiree |
| 913 | Wrong outcome | Improving Patient Safety and Clinician Workflow in the General Care Setting With Enhanced Surveillance Monitoring | 2019 | McGrath, S. P. and Perreard, I. M. and Garland, M. D. and Converse, K. A. and Mackenzie, T. A. |
| 914 | Wrong outcome | Improving patient safety through the use of nursing surveillance | 2017 | Giuliano, K. K. |
| 915 | Wrong outcome | Improving quality in resource poor settings: Observational study from rural Rwanda | 2009 | Kotagal, M. and Lee, P. and Habiyakare, C. and Dusabe, R. and Kanama, P. and Epino, H. M. and Rich, M. L. and Farmer, P. E. |
| 916 | Wrong outcome | Improving rates of metabolic monitoring on an inpatient psychiatric ward | 2020 | Michael, S. and MacDonald, K. |
| 917 | Wrong outcome | In-Hospital Cardiopulmonary Arrest: The Role of Clinical Nurses as First Responders | 2023 | Kipourgos, George and Papakonstantopoulos, Stavros and Albani, Eleni and Tzenalis, Anastasios |
| 918 | Wrong outcome | In-hospital resuscitation: recognising and responding to adults in cardiac arrest | 2016 | Simpson, Elizabeth |
| 919 | Wrong outcome | (In)accuracy of blood pressure measurement in 14 Italian hospitals | 2012 | Manzoli, L. and Simonetti, V. and D'Errico, M. M. and De Vito, C. and Flacco, M. E. and Forni, C. and La Torre, G. and Liguori, G. and Messina, G. and Mezzetti, A. and Panella, M. and Pizzi, C. and Siliquini, R. and Villari, P. and Cicolini, G. |
| 920 | Wrong outcome | The inaccuracy of automatic devices taking postural measurements in the emergency department | 2011 | Dind, A. and Short, A. and Ekholm, J. and Holdgate, A. |
| 921 | Wrong outcome | Inadvertent Perioperative Hypothermia Risks and Postoperative Complications: A Retrospective Study | 2019 | Akers, J. L. and Dupnick, A. C. and Hillman, E. L. and Bauer, A. G. and Kinker, L. M. and Hagedorn Wonder, A. |
| 922 | Wrong outcome | Incidence, severity and detection of blood pressure and heart rate perturbations in postoperative ward patients after noncardiac surgery | 2023 | Khanna, A. K. and O'Connell, N. S. and Ahuja, S. and Saha, A. K. and Harris, L. and Cusson, B. D. and Faris, A. and Huffman, C. S. and Vallabhajosyula, S. and Clark, C. J. and Segal, S. and Wells, B. J. and Kirkendall, E. S. and Sessler, D. I. |
| 923 | Wrong outcome | Increasing Supportive Care for Patients With COVID-19-Related Respiratory Deterioration in Non-ICU Settings | 2023 | Stellpflug, C. L. and Olson, S. D. and Balko, B. A. and Mrowka, V. G. and Focht, G. D. and Elmer, J. L. |
| 924 | Wrong outcome | Increasing vigilance on the medical/surgical floor to improve patient safety | 2007 | Jacobs, J. L. and Apatov, N. and Glei, M. |
| 925 | Wrong population | Incubators versus mothers' arms: body temperature conservation in very-low-birth-weight premature infants | 2001 | Mellien, A. C. |
| 926 | Foreign Language | Indagine sulle conoscenze infermieristiche del sistema di monitoraggio invasivo della Pressione Arteriosa in UnitÃ  di Terapia Intensiva | 2023 | Proietti, Stefano and Angelelli, Francesca and Lorenzetti, Claudia and Carelli, Simone |
| 927 | Wrong outcome | [Index of Caring Complexity as a management tool in a surgical setting: an observational study] | 2014 | Simonetti, V. and Comparcini, D. and Buccolini, M. and Cicolini, G. |
| 928 | Wrong outcome | Indicators for monitoring care improvement of emergency transfer of perinatal after the evaluation of the emergency transport of perinatal patients in the Trento provincie | 1999 | Cologna, M. and Pederzini, F. and Benedetti, D. and Lorenzi, S. and Faes, L. |
| 929 | Wrong outcome | Indicators of patient deterioration in poorly resourced private hospitals: Which vital sign to watch? A retrospective case-control study | 2024 | Le Lagadec, M. D. and Dwyer, T. and Browne, M. |
| 930 | Wrong outcome | An indirect method for the continuous monitoring of the respiration of the non intubated seriously ill patient | 1976 | Tahl, H. U. and Kraemer, M. |
| 931 | Wrong outcome | [INDUCED HYPOTHERMIA AFTER CARDIAC ARREST] | 2014 | Aguilera Torrico, E. and Zapata Monfort, E. and Nunell, M. C. and Cabello Ledesma, V. and MartÃ­nez Estalella, G. |
| 932 | Wrong population | Infant exposure to cigarette smoke | 1996 | Stepans, M. E. F. |
| 933 | Wrong outcome | Infectious Diseases Society of America/American Thoracic Society Consensus Guidelines on the management of community-acquired pneumonia in adults | 2007 | Mandell, L. A. and Wunderink, R. G. and Anzueto, A. and Bartlett, J. G. and Campbell, G. D. and Dean, N. C. and Dowell, S. F. and File Jr, T. M. and Musher, D. M. and Niederman, M. S. and Torres, A. and Whitney, C. G. |
| 934 | Wrong outcome | Influence of "Hospital-Community-Family" Integrated Management on Blood Pressure, Quality of Life, Anxiety and Depression in Hypertensive Patients | 2022 | Shi, W. and Cheng, L. and Li, Y. |
| 935 | Wrong population | Influence of Afferent nursing positions on vital signs of premature infants receiving nasogastric feeding and its safety analyst | 2015 | Wang, Guishu |
| 936 | Wrong population | The influence of auditory experience on the behavior of preterm newborns | 2000 | Philbin, M. K. |
| 937 | Wrong outcome | The influence of domestic overload on the association between job strain and ambulatory blood pressure among female nursing workers | 2013 | Portela, L. F. and Rotenberg, L. and Almeida, A. L. and Landsbergis, P. and Griep, R. H. |
| 938 | Wrong outcome | Influence of individual nursing care on postoperative early recovery and negative emotions in primary liver cancer patients | 2018 | Li, S. and Zhou, Y. |
| 939 | Wrong population | Influence of kangaroo care and traditional nursing care on premature physiologic parameters | 2000 | Yin, Y. and Wang, R. and Lee, M. M. and Yuh, Y. |
| 940 | Wrong outcome | Influence of Obesity on Heart Rate Variability in Nurses with Age and Shift Type as Moderators | 2021 | Chang, W. P. and Wang, C. H. and Lin, Y. K. |
| 941 | Wrong population | Influence of whole nursing intervention on rehabilitation of children after thoracoscopic minimally invasive Nuss operation | 2014 | Xu, Min and Liu, Xia and Ding, Yuefeng |
| 942 | Wrong outcome | Information Technology-Based Management of Clinically Healthy COVID-19 Patients: Lessons From a Living and Treatment Support Center Operated by Seoul National University Hospital | 2020 | Bae, Y. S. and Kim, K. H. and Choi, S. W. and Ko, T. and Jeong, C. W. and Cho, B. and Kim, M. S. and Kang, E. |
| 943 | Wrong outcome | Informing the Design of "Lifestyle Monitoring" Technology for the Detection of Health Deterioration in Long-Term Conditions: A Qualitative Study of People Living With Heart Failure | 2017 | Hargreaves, S. and Hawley, M. S. and Haywood, A. and Enderby, P. M. |
| 944 | Wrong outcome | The initial attempt at home hemodialysis in mainland China | 2022 | Ni, Z. and Zhou, Y. and Lu, R. and Shen, J. and Gu, L. and Mou, S. and Zhao, L. and Zhang, H. and Zhang, B. and Fang, Y. and Fang, W. and Wang, Q. and Zhang, W. and Zhang, J. and Li, W. |
| 945 | Wrong outcome | Initial psychometric evaluation of the physical health attitude scale and a survey of mental health nurses | 2020 | Ã–zaslan, Z. and Bilgin, H. and Uysal YalÃ§Ä±n, S. and Haddad, M. |
| 946 | Wrong outcome | Injectable Amoxicillin Versus Injectable Ampicillin Plus Gentamicin in the Treatment of Severe Pneumonia in Children Aged 2 to 59 Months: Protocol for an Open-Label Randomized Controlled Trial | 2020 | Shahrin, L. and Chisti, M. J. and Shahid, Asmsb and Rahman, Asmmh and Islam, M. Z. and Afroze, F. and Huq, S. and Ahmed, T. |
| 947 | Wrong outcome | Innovation in the transport of cytotoxic drugs by a pneumatic transport system | 2017 | Baillie, C. and Desplanques, M. and Delbey, S. and Sakji, I. and Feutry, F. |
| 948 | Wrong population | Instrument for classifying dependency in neonatal intensive care: analysis of agreement and reliability | 2024 | Lopes, P. M. B. and Torres, F. B. G. and Massaneiro, T. J. and Hino, A. A. and Carmona, E. V. and Cubas, M. R. |
| 949 | Wrong outcome | An integrated care pathway designing hospital care at home for elderly with an acute respiratory infection...22nd International Conference on Integrated Care, May 23-25, 2022, Odense, Denmark | 2022 | Pepping, Rianne |
| 950 | Wrong outcome | An integrated, cost-effective efficient chain to deliver botulinum toxin in optimal conditions | 2019 | Beldarrain, Marian Gomez and IbaÃ±ez, Amaia Santos and Fernandez, Maria Carmen Maldonado and Bengoechea, Maria Jose Martinez and Garcia-Monco, Juan Carlos |
| 951 | Wrong outcome | Integrating acute and subacute care through dynamic adaption of care intensity: The Integrated General Hospital Pilot in Singapore | 2019 | Ting, Jeanette and Gollamudi, Satya and Gim Gee, Teng and See Meng, Khoo |
| 952 | Wrong outcome | Integrating Care in Complex Cardiac Care: the Tech Touch | 2016 | Stellato, Kira and Radini, Donatella and Pellizzari, Mara and Pordenon, Marta and Pletti, Luciano and Humar, Franco and Apuzzo, Matteo and Di Lenarda, Andrea and Fragiacomo, Emanuela and Quadri, Nicola Delli |
| 953 | Wrong outcome | Integrating hospital and community care: using a community virtual ward model to deliver combined specialist and generalist care to patients with severe chronic respiratory disease in their homes | 2022 | Cushen, B. and Madden, A. and Long, D. and Whelan, Y. and O'Brien, M. E. and Carroll, D. and O'Flynn, D. and Forde, M. and Pye, V. and Grogan, L. and Casey, M. and Farrell, K. and Costello, R. W. and Lewis, C. |
| 954 | Wrong outcome | Integrating Structured and Unstructured EHR Data for Predicting Mortality by Machine Learning and Latent Dirichlet Allocation Method | 2023 | Chiu, C. C. and Wu, C. M. and Chien, T. N. and Kao, L. J. and Li, C. and Chu, C. M. |
| 955 | Wrong outcome | Integration of Remote Symptom and Biometric Monitoring into the Care of Adult Patients with Cancer Receiving Chemotherapy-A Decentralized Feasibility Pilot Study | 2023 | Offodile, A. C. and Delgado, D. and Lin, Y. L. and Geyen, D. and Miller, C. J. and Jain, S. and Finder, J. P. and Shete, S. and Fossella, F. V. and Overman, M. J. and Peterson, S. K. |
| 956 | Wrong outcome | Intelligent infusion controller with a physiological information feedback function | 2020 | Li, J. and Dong, P. and Lai, Y. and Qin, X. and Bai, J. and Wang, H. and Wan, Z. and Ji, Z. and Shi, X. |
| 957 | Wrong outcome | An Intelligent Medical Isolation Observation Management System Based on the Internet of Things | 2022 | Sun, W. and Wang, C. and Sun, J. and Miao, Z. and Ling, F. and Wu, G. |
| 958 | Wrong outcome | [Intelligent Nursing System] | 2021 | Chen, B. and Lu, L. |
| 959 | Wrong outcome | Intensive care of a patient undergoing combined multi-organ cluster ("larynx-trachea-thyroid-hypopharynx-esophagus") transplantation: A case report | 2024 | Shen, J. and Liu, H. and Zhang, Y. and Xu, Y. and Du, A. and Tian, Y. |
| 960 | Wrong outcome | Intensive care unit telemedicine: Review and consensus recommendations | 2007 | Cummings, J. and Krsek, C. and Vermoch, K. and Matuszewski, K. |
| 961 | Wrong outcome | Intensive care unit without walls: seeking patient safety by improving the efficiency of the system | 2014 | Gordo, F. and Abella, A. |
| 962 | Wrong outcome | Intensive Nursing Care by an Electronic Followup System to Promote Secondary Prevention After Percutaneous Coronary Intervention: A RANDOMIZED TRIAL | 2014 | Hu, Xin and Zhu, Xiuqin and Gao, Lei |
| 963 | Wrong outcome | Inter-hospital transfers of acutely ill adults in Scotland | 2010 | Fried, M. J. and Bruce, J. and Colquhoun, R. and Smith, G. |
| 964 | Wrong outcome | Inter-Observer Agreement in Measuring Respiratory Rate | 2015 | Nielsen, L. G. and Folkestad, L. and Brodersen, J. B. and Brabrand, M. |
| 965 | Wrong outcome | Inter-rater reliability of the Silverman and Andersen index-a measure of respiratory distress in preterm infants | 2023 | Brenne, H. and Follestad, T. and Bergseng, H. and Eriksen, B. H. and SÃ¸raunet, K. and Grunewaldt, K. H. |
| 966 | Wrong outcome | Interactive telemedicine: effects on professional practice and health care outcomes | 2015 | Flodgren, G. and Rachas, A. and Farmer, A. J. and Inzitari, M. and Shepperd, S. |
| 967 | Wrong outcome | Internet of things (IoT) in hospitals and healthcare industry for better operations and quality of care | 2018 | Panditi, D. D. and Badri Narayanan, M. K. |
| 968 | Wrong outcome | Interobserver and intraobserver reliability in the collection of emergency medical services data | 1980 | Herrmann, N. and Cayten, C. G. and Senior, J. and Staroscik, R. and Walsh, S. and Woll, M. |
| 969 | Wrong outcome | Interobserver concordance (reliability) in the measurement of vital signs at the National Institute of Respiratory Diseases Ismael CosÃ­o Villegas | 2006 | CastaÃ±Ã³n-MejÃ­a, A. and Santos-MartÃ­nez, L. E. and JimÃ©nez, A. and Monroy, M. D. C. |
| 970 | Wrong outcome | Interpretation of continuously measured vital signs data of COVID-19 patients by nurses and physicians at the general ward: A mixed methods study | 2023 | van Goor, H. M. R. and Breteler, M. J. M. and Schoonhoven, L. and Kalkman, C. J. and van Loon, K. and Kaasjager, K. A. H. |
| 971 | Wrong outcome | An intervention including the national early warning score improves patient monitoring practice and reduces mortality: A cluster randomized controlled trial | 2019 | Haegdorens, F. and Monsieurs, K. G. and De Meester, K. and Van Bogaert, P. |
| 972 | Wrong outcome | Interventions to improve medication adherence among Chinese patients with hypertension: a systematic review and meta-analysis of randomized controlled trails | 2018 | Xu, R. and Xie, X. and Li, S. and Chen, X. and Wang, S. and Hu, C. and Lv, X. |
| 973 | Wrong outcome | Intra- and inter-operator variability of refractometric total proteins measurement of canine plasma | 2022 | Venier, F. and Jamont, W. and McLennan, K. and Rosa, C. |
| 974 | Wrong outcome | Intra-hospital transfer anxiety of patients in the neurosurgery intensive care unit: A prospective cohort study | 2023 | Ortahisar, B. K. and Uslu, Y. |
| 975 | Wrong outcome | [Intracranial Pressure Monitor Based on Wireless] | 2017 | Han, H. and Zhang, Y. and Qian, C. and Wang, H. and Qian, Z. and Li, W. |
| 976 | Wrong outcome | Intraoperative progress reports decrease family members' anxiety | 1996 | Leske, J. S. |
| 977 | Wrong outcome | An Intraoperative Telemedicine Program to Improve Perioperative Quality Measures: The ACTFAST-3 Randomized Clinical Trial | 2023 | King, C. R. and Gregory, S. and Fritz, B. A. and Budelier, T. P. and Ben Abdallah, A. and Kronzer, A. and Helsten, D. L. and Torres, B. and McKinnon, S. and Goswami, S. and Mehta, D. and Higo, O. and Kerby, P. and Henrichs, B. and Wildes, T. S. and Politi, M. C. and Abraham, J. and Avidan, M. S. and Kannampallil, T. |
| 978 | Wrong population | Intrapartal care documented in a Swedish maternity unit and considered in relation to World Health Organization recommendations for care in normal birth | 2006 | Sandin-BojÃ¶, A. K. and Larsson, B. W. and Axelsson, O. and Hall-Lord, M. L. |
| 979 | Wrong outcome | Intraprofessional collaboration between enrolled and registered nurses in the care of clinically deteriorating ward patients: AÂ qualitative study | 2022 | Chua, W. L. and Rahim, Nrba and McKenna, L. and Ho, J. T. Y. and Liaw, S. Y. |
| 980 | Wrong outcome | Intravenous immunoglobulin administration: an evaluation of vital monitoring | 1994 | Camp-Sorrell, D. and Wujcik, D. |
| 981 | Wrong outcome | Intravenous lidocaine for cancer pain without electrocardiographic monitoring: a retrospective review | 2015 | Peixoto, R. D. and Hawley, P. |
| 982 | Wrong outcome | Introducing capnography monitoring into an endoscopy service | 2016 | Egan, Elaine |
| 983 | Wrong outcome | Introducing epidural fentanyl for on-ward pain relief after major surgery | 1996 | SalomÃ¤ki, T. E. and Kokki, H. and Turunen, M. and Havukainen, U. and Nuutinen, L. S. |
| 984 | Wrong outcome | Introduction of Clinical Practice Guidelines for Emergency Patients | 2018 | Yi-Maun, Subeq and Wai-Mau, Choi and Cian-Huei, Shih and Ru-Ping, L. E. E. |
| 985 | Wrong outcome | [Introduction of Clinical Practice Guidelines for Emergency Patients] | 2018 | Subeq, Y. M. and Choi, W. M. and Shih, C. H. and Lee, R. P. |
| 986 | Wrong outcome | Investigating the prevalence of oxygen desaturation in post-anesthesia care unit and preoperative predicting factors | 2017 | Sajedi, P. and Sharifi-Dorcheh, F. and Hessam, F. |
| 987 | Wrong outcome | Investigation and analysis of the development status of critical care medicine in Xinjiang Uygur Autonomous Region in 2019 | 2020 | Xiang, L. and Huxitaer, G. and Yi, W. and Xiaoli, H. and Xinxin, D. and Xiangyou, Y. |
| 988 | Wrong outcome | [Investigation and analysis of the development status of critical care medicine in Xinjiang Uygur Autonomous Region in 2019] | 2020 | Li, X. and Gulibanumu, H. and Wang, Y. and Hua, X. and Du, X. and Yu, X. |
| 989 | Wrong outcome | Investigation of the effects of chest physiotherapy in different positions on the heart and the respiratory system after coronary artery bypass surgery | 2015 | Guner, S. I. and Korkmaz, F. D. |
| 990 | Wrong outcome | Investigation of the Relationship Between Socio-Demographic Characteristics, Metabolic Variables, and Treatment Adherence in Individuals with Diabetes | 2022 | Aslan, Gamze YÄ±ldÄ±z and Tekir, Ã–zlem and YÄ±ldÄ±z, Hicran |
| 991 | Wrong outcome | Investigation on monitoring index of death risk in inpatients with chronic non-communicable diseases | 2018 | Yan, Yechao and Zhang, Lin and Gu, Yu |
| 992 | Wrong outcome | IoT based Saline Monitoring System | 2022 | Selvi, M. C. and Bhuvaneswari, T. and Priyadarshini, R. N. and Chitralekha, R. and Ramya, R. |
| 993 | Wrong outcome | Is "really conscious" sedation with solely an opioid an alternative to every day used sedation regimes for colonoscopies in a teaching hospital? Midazolam/fentanyl, propofol/alfentanil, or alfentanil only for colonoscopy: a randomized trial | 2014 | Eberl, S. and Polderman, J. A. and Preckel, B. and Kalkman, C. J. and Fockens, P. and Hollmann, M. W. |
| 994 | Wrong outcome | Is clinical assessment of the circulation reliable in postoperative cardiac surgical patients? | 2002 | Linton, R. A. and Linton, N. W. and Kelly, F. |
| 995 | Wrong outcome | Is Modified Early Warning Score associated with clinical outcomes of patients admitted to a university internal medicine ward? | 2023 | Saba, A. and Nunes, Mdpt |
| 996 | Wrong outcome | Is nurse staffing associated with critical deterioration events on acute and critical care pediatric wards? A literature review | 2023 | Genna, C. and Thekkan, K. R. and Raymakers-Janssen, Pama and Gawronski, O. |
| 997 | Wrong outcome | Is nurse staffing associated with critical deterioration events on acute and critical care pediatric wards? A literature review | 2023 | Genna, C. and Thekkan, K. R. and Raymakers-Janssen, P. A. M. A. and Gawronski, O. |
| 998 | Wrong outcome | Is the Modified Early Warning Score able to enhance clinical observation to detect deteriorating patients earlier in an Accident & Emergency Department? | 2015 | Shuk-Ngor, So and Chi-Wai, Ong and Lai-Yee, Wong and Chung, Josephine Y. M. and Graham, Colin A. |
| 999 | Wrong outcome | Iv NTG: monitoring vital signs hourly versus every two hours | 1990 | Ardire, L. |
| 1000 | Wrong outcome | Job strain and physiological stress responses in nurses and nurse's aides: predictors of daily blood pressure variability | 2003 | Brown, D. E. and James, G. D. and Nordloh, L. and Jones, A. A. and Brown, Daniel E. and James, Gary D. and Nordloh, Lea and Jones, Amy A. |
| 1001 | Wrong outcome | John M. Eisenberg Patient Safety and Quality Awards: use of the modified early warning score decreases code blue events | 2009 | Maupin, J. M. and Roth, D. J. and Krapes, J. M. |
| 1002 | Wrong outcome | The Kaiser Permanente Northern California Advance Alert Monitor Program: An Automated Early Warning System for Adults at Risk for In-Hospital Clinical Deterioration | 2022 | Martinez, Vanessa A. and Betts, Robin K. and Scruth, Elizabeth A. and Buckley, Jacqueline D. and Cadiz, Vilma R. and Bertrand, Linda D. and Paulson, Shirley S. and Dummett, Brian Alex and Abhyankar, Stella S. and Reyes, Vivian M. and Hatton, Joeffrey R. and Sulit, Reynaldo and Liu, Vincent X. |
| 1003 | Wrong population | Keep Calm and Carry On: Reducing Elective Early-Term Deliveries | 2014 | Sincore, Tammy J. and Levine, Linda C. |
| 1004 | Wrong outcome | Keeping record of the postoperative nursing care of patients | 2002 | Roets, L. and Aucamp, M. C. and de Beer, H. and Niemand, M. |
| 1005 | Wrong population | Ketamine sedation for the reduction of children's fractures in the emergency department | 2000 | McCarty, E. C. and Mencio, G. A. and Walker, L. A. and Green, N. E. |
| 1006 | Wrong outcome | Ketamine-midazolam versus meperidine-midazolam for painful procedures in pediatric oncology patients | 1997 | Marx, C. M. and Stein, J. and Tyler, M. K. and Nieder, M. L. and Shurin, S. B. and Blumer, J. L. and Marx, C. M. and Stein, J. and Tyler, M. K. and Nieder, M. L. and Shurin, S. B. and Blumer, J. L. |
| 1007 | Wrong outcome | Knowledge and practices of blood pressure measurement among final year students, house officers, and resident dental surgeons in a dental hospital, South West Nigeria | 2020 | Ibiyemi, O. and Ogunbodede, O. and Gbolahan, O. O. and Ogah, O. S. |
| 1008 | Wrong outcome | [Knowledge and practices of hypertension in nursing staff of the Hospital Gabriel TourÃ© and Point G] | 2014 | Menta, I. and Diall, I. B. and Coulibaly, S. and Bah, O. and SangarÃ©, I. and SidibÃ©, N. and Kone, O. D. and TraorÃ©, D. and Camara, Y. and TourÃ©, K. and Diarra, A. and KÃ©ita, L. and TraorÃ©, A. and Sanogo, K. M. |
| 1009 | Wrong outcome | [Knowledge and practices regarding stroke at a university hospital: Part 1. Education of the nursing staff: priorities for the treatment of cerebral infarction] | 1997 | AndrÃ©, C. and Costa, M. F. and Raggio, R. and Vermelho, L. L. and Novis, S. A. |
| 1010 | Wrong population | Knowledge and skills of pre-eclampsia management among healthcare providers working in antenatal clinics in Zanzibar | 2022 | Seif, S. A. and Rashid, S. A. |
| 1011 | Wrong outcome | Lack of treatment continuance: An obstacle for controlling blood pressure | 2005 | Leibovitz, E. and Hertsog, D. and Oren, S. and Gavish, D. |
| 1012 | Wrong outcome | Lack of validation of the Dixtal (DX 2020) upper arm blood pressure monitor, in oscillometric mode, for clinical use in an intensive care unit, according to the European Society of Hypertension-International Protocol revision 2010 | 2013 | Gothardo, A. C. L. O. and Savioli, A. F. and Santos, D. S. and Lamas, J. L. T. |
| 1013 | Wrong outcome | Late Rescue Collaborative: Reducing Non-ICU Arrests | 2020 | Dean, Nathan P. and Ghebremariam, Emanuel and Szeles, Rosemary and Levin, Amanda and Colyer, Jessica and Steinhorn, Robin H. |
| 1014 | Wrong population | Lean Design of the Pediatric Intensive Care Unit Patient Room for Efficient and Safe Care Delivery | 2022 | Lu, Yuqian and Bishop, Naomi B. and Zadeh, Rana |
| 1015 | Wrong outcome | Lessons from 30 years' data of Korean end-stage renal disease registry, 1985-2015 | 2015 | Jin, D. C. and Yun, S. R. and Lee, S. W. and Han, S. W. and Kim, W. and Park, J. and Kim, Y. K. |
| 1016 | Wrong outcome | Lessons learned as a research assistant studying ambulatory blood pressure in elderly Japanese stroke patients | 2003 | Nishimura, C. and Takahashi, R. and Miyamoto, S. and Saito, T. and Kanemaru, A. and Liehr, P. R. |
| 1017 | Wrong outcome | Lessons Learned from Telemonitoring in an Outpatient Bariatric Surgery Pathway-Secondary Outcomes of a Patient Preference Clinical Trial | 2023 | van Ede, E. S. and Scheerhoorn, J. and Schonck, Fmjf and van der Stam, J. A. and Buise, M. P. and Nienhuijs, S. W. and Bouwman, R. A. |
| 1018 | Wrong outcome | Letter to the editor: efficacy of different methods of combination regimen administrations including dexamethasone, intravenous immunoglobulin, and interferon-beta to treat critically ill COVID-19 patients: a structured summary of a study protocol for a randomized controlled trial | 2020 | Abdolahi, N. and Kaheh, E. and Golsha, R. and Khodabakhshi, B. and Norouzi, A. and Khandashpoor, M. and Besharat, S. and Tavassoli, S. and Livani, S. and Azimi, S. A. and Gharib, M. H. and Peivandi, B. and Fazel, A. and Shirzad-Aski, H. and Roshandel, G. |
| 1019 | Wrong outcome | Life-Sustaining Treatment Decision in Palliative Care Based on Electronic Health Records Analysis | 2023 | Kim, S. and Lim, A. and Jang, H. and Jeon, M. |
| 1020 | Wrong outcome | Lifestyle interventions reduce cardiovascular risk in patients with coronary artery disease: a randomized clinical trial | 2014 | Saffi, M. A. and Polanczyk, C. A. and Rabelo-Silva, E. R. |
| 1021 | Wrong outcome | Limits of electronical monitoring - The value of clinical signs | 2003 | Langgartner, J. and Klebl, F. and SchÃ¶lmerich, J. and Reng, C. M. |
| 1022 | Wrong outcome | Linezolid desensitization for a patient with multiple medication hypersensitivity reactions | 2013 | Bagwell, A. D. and Stollings, J. L. and White, K. D. and Fadugba, O. O. and Choi, J. J. |
| 1023 | Wrong outcome | Linking hospital and residential aged care: a nurse-led vascular-geriatric model of care | 2021 | Schasser, Suzy and Monaro, Susan and West, Sandra |
| 1024 | Wrong outcome | Long term monitoring of a pressure ulcer risk patient using thermal images | 2017 | Bennett, S. L. and Goubran, R. and Knoefel, F. |
| 1025 | Wrong outcome | Long-term adherence to a local guideline on postoperative body temperature measurement: mixed methods analysis | 2012 | Storm-Versloot, Marja N. and Knops, Anouk M. and Ubbink, Dirk T. and Goossens, Astrid and Legemate, Dink A. and Vermeulen, Hester |
| 1026 | Wrong outcome | Long-Term Effect of Home Blood Pressure Self-Monitoring Plus Medication Self-Titration for Patients with Hypertension: A Secondary Analysis of the ADAMPA Randomized Clinical Trial | 2024 | MartÃ­nez-IbÃ¡Ã±ez, P. and Marco-Moreno, I. and GarcÃ­a-Sempere, A. and PeirÃ³, S. and MartÃ­nez-IbÃ¡Ã±ez, L. and Barreira-Franch, I. and Bellot-Pujalte, L. and Avelino-Hidalgo, E. and Escrig-Veses, M. and BÃ³veda-GarcÃ­a, M. and Calleja-Del-Ser, M. and Robles-CabaniÃ±as, C. and Hurtado, I. and RodrÃ­guez-Bernal, C. L. and GimÃ©nez-Loreiro, M. and SanfÃ©lix-Gimeno, G. and SanfÃ©lix-GenovÃ©s, J. |
| 1027 | Wrong outcome | Long-term monitoring of arterial pO2 in burned patients | 1984 | Nilsson, E. and Arnander, C. |
| 1028 | Wrong outcome | A longitudinal study of nonvitamin, nonmineral supplement use: prevalence, associations, and survival in an aging population | 2007 | Knudtson, M. D. and Klein, R. and Lee, K. E. and Reinke, J. O. and Danforth, L. G. and Wealti, A. M. and Moore, E. and Klein, B. E. K. and Knudtson, Michael D. and Klein, Ronald and Lee, Kristine E. and Reinke, Jennifer O. and Danforth, Lorraine G. and Wealti, Angela M. and Moore, Emily and Klein, Barbara E. K. |
| 1029 | Wrong outcome | Low airloss hydrotherapy versus standard care for incontinent hospitalized patients | 1998 | Bennett, R. G. and Baran, P. J. and DeVone, L. V. and Bacetti, H. and Kristo, B. and Tayback, M. and Greenough, W. B., 3rd |
| 1030 | Wrong outcome | Low airloss hydrotherapy versus standard care for incontinent hospitalized patients | 1998 | Bennett, R. G. and Baran, P. J. and DeVone, L. and Bacetti, H. and Kristo, B. and Tayback, M. and Greenough Iii, W. B. |
| 1031 | Wrong outcome | Low cardiac output syndrome in critically ill child, nursing considerations | 2014 | Garzon, J. X. R. |
| 1032 | Wrong outcome | Low compliance to a vital sign safety protocol on general hospital wards: A retrospective cohort study | 2021 | Eddahchouri, Y. and Koeneman, M. and Plokker, M. and Brouwer, E. and van de Belt, T. H. and van Goor, H. and Bredie, S. J. |
| 1033 | Wrong outcome | Low compliance to a vital sign safety protocol on general hospital wards: A retrospective cohort study | 2021 | Eddahchouri, Yassin and Koeneman, Mats and Plokker, Manon and Brouwer, Egbert and van de Belt, Tom H. and van Goor, Harry and Bredie, Sebastian J. H. |
| 1034 | Wrong outcome | Low-Cost Technologies that can be Integrated into Medical Education in Emerging Areas | 2022 | ÈšoÈ›a, P. and Vaida, M. F. |
| 1035 | Wrong outcome | Lower incidence of unexpected in-hospital death after interprofessional implementation of a bedside track-and-trigger system | 2014 | Bunkenborg, G. and Samuelson, K. and Poulsen, I. and Ladelund, S. and Ã…keson, J. |
| 1036 | Wrong outcome | m-Health: Lessons Learned by m-Experiences | 2018 | Bravo, J. and HervÃ¡s, R. and Fontecha, J. and GonzÃ¡lez, I. |
| 1037 | Wrong outcome | Machine Learning-Based Prediction Models of Mortality for Intensive Care Unit Patients Using Nursing Records | 2024 | Kim, Y. and Kim, Y. and Choi, M. |
| 1038 | Wrong outcome | Magnitude and associated factors of diabetes mellitus and hypertension among adult HIV-positive individuals receiving highly active antiretroviral therapy at Jugal Hospital, Harar, Ethiopia | 2018 | Ataro, Z. and Ashenafi, W. and Fayera, J. and Abdosh, T. |
| 1039 | Wrong outcome | The main Optimal Post rTpa-Iv Monitoring in Ischemic Stroke Trial (OPTIMISTmain): Protocol for a Pragmatic, Stepped Wedge, Cluster Randomized Controlled Trial | 2023 | Ouyang, M. and Faigle, R. and Wang, X. and Johnson, B. and Summers, D. and Khatri, P. and Billot, L. and Liu, H. and Malavera, A. and MuÃ±oz-Venturelli, P. and GonzÃ¡lez, F. and Urrutia, F. and Day, D. and Song, L. and Sui, Y. and Delcourt, C. and Robinson, T. and Durham, A. C. and Ebraimo, A. and Zaidi, W. A. W. and Jan, S. and Lindley, R. I. and Urrutia, V. C. and Anderson, C. S. |
| 1040 | Wrong outcome | Maintaining a safe environment in emergency department waiting rooms | 2024 | Robinson, S. |
| 1041 | Wrong outcome | Management of abnormal observations in the emergency department: A review | 2019 | Trajkovska, Aleksandra and Farooq, Munawar and Richardson, Drew |
| 1042 | Wrong outcome | Management of aneurysmal subarachnoid hemorrhage | 1995 | Miller, J. and Diringer, M. |
| 1043 | Wrong outcome | Management of Fever, Hyperglycemia, and Dysphagia in an Acute Stroke Unit | 2016 | Kenny, T. and Barr, C. and Laver, K. |
| 1044 | Wrong outcome | Managing diabetes care using an integrated regional e-health approach | 2006 | Harno, K. and Kauppinen-MÃ¤kelin, R. and SyrjÃ¤lÃ¤inen, J. |
| 1045 | Wrong outcome | Managing diabetes in the hospital setting: a nurse-patient partnership | 2003 | Walker, R. |
| 1046 | Wrong outcome | Managing epistaxis in A&E | 1999 | Bird, D. |
| 1047 | Wrong outcome | Managing pressure relief in a special needs nursing scheme | 1997 | Knowles, C. |
| 1048 | Wrong outcome | Mandatory early warning scoring-implementation evaluated with a mixed-methods approach | 2016 | Bunkenborg, Gitte and Poulsen, Ingrid and Samuelson, Karin and Ladelund, Steen and Akeson, Jonas |
| 1049 | Wrong population | Maternal cardiac autonomic function and fetal heart rate in preeclamptic compared to normotensive pregnancies | 2005 | Swansburg, M. L. and Brown, C. A. and Hains, S. M. and Smith, G. N. and Kisilevsky, B. S. |
| 1050 | Wrong outcome | Maternal hypothermia during elective caesarean delivery: A prospective observational study | 2024 | Brodshaug, I. and Reine, E. and Raeder, J. |
| 1051 | Wrong outcome | Meal-Monitoring Systems Using Weight and Temperature Sensors for Elder Residents in Long-Term Care Facilities | 2022 | Hu, Y. and Joo, J. E. and Choi, E. and Yoo, L. and Jung, D. and Shin, J. H. and Kim, J. H. and Park, S. M. |
| 1052 | Wrong outcome | The mean prehospital machine; accurate prehospital non-invasive blood pressure measurement in the critically ill patient | 2010 | Muecke, S. and Bersten, A. and Plummer, J. and Muecke, Sandy and Bersten, Andrew and Plummer, John |
| 1053 | Wrong outcome | Measurement of body temperature in adult patients: Comparative study of accuracy, reliability and validity of different devices | 2011 | Rubia-Rubia, J. and Arias, A. and Sierra, A. and Aguirre-Jaime, A. |
| 1054 | Wrong outcome | Measuring adherence among nurses one year after training in applying the Modified Early Warning Score and Situation-Background-Assessment-Recommendation instruments | 2011 | Ludikhuize, J. and de Jonge, E. and Goossens, A. |
| 1055 | Wrong outcome | Measuring central venous pressure | 2007 | Cole, E. |
| 1056 | Wrong outcome | Measuring patient anxiety in coronary care... part 1 | 1992 | Elliott, D. |
| 1057 | Wrong outcome | Measuring the 'fifth vital sign' in cataract surgery patients -- is it necessary? | 2006 | Henry, C. and Navarro, V. and Jun, A. and Annaberdyev, S. |
| 1058 | Wrong outcome | Measuring the Effects of Sharing Mobile Health Data During Diabetes Consultations: Protocol for a Mixed Method Study | 2020 | Bradway, M. and Giordanengo, A. and Joakimsen, R. and Hansen, A. H. and GrÃ¸ttland, A. and Hartvigsen, G. and Randine, P. and Ã…rsand, E. |
| 1059 | Wrong outcome | Measuring the modified early warning score and the Rothman index: advantages of utilizing the electronic medical record in an early warning system | 2014 | Finlay, G. D. and Rothman, M. J. and Smith, R. A. |
| 1060 | Wrong outcome | Mechanism of paracetamol-induced hypotension in critically ill patients: A prospective observational cross-over study | 2013 | KrajÄovÃ¡, A. and MatouÅ¡ek, V. and DuÅ¡ka, F. |
| 1061 | Wrong outcome | Medical Care of Patient with HIV-Associated Kaposiâ€™s Sarcoma: Case Report | 2022 | GenÃ§, Z. and Yildirim, D. and AkyÃ¼z Ã–zdemir, F. and Tekin, S. |
| 1062 | Wrong outcome | Medical device-related pressure injuries in paediatric patients: An incidence study in a children's hospital | 2024 | BaÅŸbakkal, Z. and Yilmaz, H. B. and GÃ¼mÃ¼ÅŸ, M. and Belli, M. and ErÃ§elik, Z. E. |
| 1063 | Wrong outcome | Medication error reporting in Tehran: a survey | 2014 | Hajibabaee, F. and Joolaee, S. and Peyravi, H. and Alijany-Renany, H. and Bahrani, N. and Haghani, H. |
| 1064 | Wrong outcome | MEDICINES OPTIMISATION FOR PATIENTS IN A NURSING HOME...European Association of Hospital Pharmacists (EAHP) 28th Congress, March 20-22, 2024, Bordeaux, France | 2024 | Soler, F. M. Ferrer and MartÃ­nez, C. M. Cuadros and SÃ¡nchez, P. LÃ³pez and PÃ©rez, M. V. Peraza and Nieves, J. J. MÃ¡rquez |
| 1065 | Wrong outcome | Memory-efficient low-compute segmentation algorithms for bladder-monitoring smart ultrasound devices | 2023 | Song, Z. and Asiedu, M. and Wang, S. and Li, Q. and Ozturk, A. and Mittal, V. and Schoen, S., Jr. and Ramaswamy, S. and Pierce, T. T. and Samir, A. E. and Eldar, Y. C. and Chandrakasan, A. and Kumar, V. |
| 1066 | Wrong outcome | The mercury sphygmomanometer should be abandoned before it is proscribed | 2000 | Markandu, N. D. and Whitcher, F. and Arnold, A. and Carney, C. |
| 1067 | Wrong outcome | Metabolic control in insulin-treated type 2-diabetic patients aged over 80 years and without participation in a structured diabetic teaching program | 2005 | Sedlak, M. and Raml, A. and Schmekal, B. and Grafinger, P. and Biesenbach, G. |
| 1068 | Wrong outcome | Metabolic Side Effects in Patients Using Atypical Antipsychotic Medications During Hospitalization | 2018 | DikeÃ§, GÃ¼l and Arabaci, Leyla Baysan and Uzunoglu, GÃ¼lÃ§in BÃ¶lÃ¼k and Mizrak, Selin Demet |
| 1069 | Wrong outcome | Methodological investigation of measuring nasopharyngeal temperature as noninvasive brain temperature analogue in the neonate | 2001 | Ko, H. K. and Flemmer, A. and Haberl, C. and Simbruner, G. |
| 1070 | Wrong outcome | Methods of sedation for auditory brainstem response testing | 1996 | Reich, D. S. and Wiatrak, B. J. |
| 1071 | Wrong outcome | mHealth App to Facilitate Remote Care for Patients With COVID-19: Rapid Development of the DrCovid+ App | 2023 | Tan, J. P. Y. and Tan, M. W. J. and Towle, R. M. and Lee, J. S. W. and Lei, X. and Liu, Y. and Goh, R. S. M. and Chee Ping, F. T. and Tan, T. C. and Ting, D. S. W. and Lee, C. E. and Low, L. L. |
| 1072 | Wrong outcome | Midazolam versus propofol for long-term sedation in the ICU: A randomized prospective comparison | 1997 | Weinbroum, A. A. and Halpern, P. and Rudick, V. and Sorkine, P. and Freedman, M. and Geller, E. |
| 1073 | Wrong outcome | [The missed care in Nursing Homes: a pilot study] | 2018 | Basso, I. and Bonaudo, M. and Dimonte, V. and Campagna, S. |
| 1074 | Wrong outcome | Missed nursing care, non-nursing tasks, staffing adequacy, and job satisfaction among nurses in a teaching hospital in Egypt | 2021 | Hammad, M. and Guirguis, W. and Mosallam, R. |
| 1075 | Wrong outcome | A mixed methods thematic review: Healthâ€related decisionâ€making by the older person | 2018 | King, Lindy and Harrington, Ann and Linedale, Ecushla and Tanner, Elizabeth |
| 1076 | Wrong outcome | A mixed-methods investigation of health professionals' perceptions of a physiological track and trigger system | 2016 | Lydon, S. and Byrne, D. and Offiah, G. and Gleeson, L. and O'Connor, P. |
| 1077 | Wrong outcome | A mobile care system with alert mechanism | 2007 | Lee, R. G. and Chen, K. C. and Hsiao, C. C. and Tseng, C. L. |
| 1078 | Wrong population | Mobile Health-Collected Biophysical Markers in Children with Serious Illness-Related Pain | 2021 | Ajayi, T. A. and Salongo, L. and Zang, Y. and Wineinger, N. and Steinhubl, S. |
| 1079 | Wrong outcome | Monitoring a pulse in adults | 2018 | Allan, Jaden and Sheppard, Karen |
| 1080 | Wrong population | Monitoring and nursing for children with obstructive sleep apnea syndrome in the recovery room after general anesthesia | 2020 | Pang, C. and Niu, J. and Zhu, L. and Zhu, H. and Hu, X. and Zhang, X. and Cheng, S. |
| 1081 | Wrong outcome | Monitoring cardiorespiratory instability: Current approaches and implications for nursing practice | 2016 | Bose, E. and Hoffman, L. and Hravnak, M. |
| 1082 | Wrong outcome | Monitoring for intradialytic hypotension: An audit of nursing practice | 2021 | McIntyre, David and Havas, Kathryn and Bonner, Ann |
| 1083 | Wrong population | Monitoring hypertension in pregnancy. Home self-testing of blood pressure levels | 1991 | Kennedy, S. |
| 1084 | Wrong outcome | Monitoring of Chronic Disease in the community: Australian Telehealth Study on Organisational Challenges and Economic Impact | 2016 | Jayasena, Rajiv and Cellar, Branko and Sparks, Ross and Varnfield, Marlien and Li, Jane and Nepal, Surya |
| 1085 | Wrong population | The monitoring of critically ill neonates | 1983 | McIntosh, N. |
| 1086 | Wrong outcome | Monitoring of Diabetic Foot Syndrome Treatment: Some New Perspectives | 2011 | Foltynski, P. and Wojcicki, J. M. and Ladyzynski, P. and Migalska-Musial, K. and Rosinski, G. and Krzymien, J. and Karnafel, W. |
| 1087 | Wrong outcome | Monitoring of elderly housebound and mobile diabetic patients in 31 Leicestershire practices: a comparative study | 1999 | Farooqi, A. and Sorrie, R. |
| 1088 | Wrong outcome | Monitoring of nursing care for peripheral intravenous access in hospitalized children | 2023 | SvÄ›tnickÃ¡, G. and JaroÅ¡ovÃ¡, D. |
| 1089 | Wrong outcome | [Monitoring of patient-controlled analgesia by the nursing staff] | 2001 | Geiss, C. and MÃ¤rkert, D. and Koppert, W. and Griessinger, N. and Sittl, R. |
| 1090 | Wrong outcome | Monitoring of quality assurance of Emergency Medical Technicians (EMTs) in the emergency department | 2002 | Hu, S. C. and Yen, D. H. T. and Kao, W. F. |
| 1091 | Wrong population | Monitoring premature infants in car seats: implementing the American Academy of Pediatrics policy in a community hospital | 1993 | Bass, J. L. and Mehta, K. A. and Camara, J. |
| 1092 | Wrong outcome | Monitoring temperature in transit; pitfalls of plastic, solid-bottom trays | 2015 | Taurasi, Ray |
| 1093 | Wrong outcome | Monitoring vital signs: development of a modified early warning scoring (MEWS) system for general wards in a developing country | 2014 | Kyriacos, U. and Jelsma, J. and James, M. and Jordan, S. |
| 1094 | Wrong outcome | Monitorization of Autonomic Stress Response of Nurse Students in Hospital Clinical Simulation | 2021 | BeltrÃ¡n-Velasco, A. I. and SÃ¡nchez-Conde, P. and Ramos-Campo, D. J. and Clemente-SuÃ¡rez, V. J. |
| 1095 | Wrong outcome | Morbidity prediction using pre- and intraoperative data | 1979 | Schneider, A. J. L. and Knoke, J. D. and Zollinger Jr, R. M. |
| 1096 | Wrong outcome | Motor Activity Assessment Scale: A valid and reliable sedation scale for use with mechanically ventilated patients in an adult surgical intensive care unit | 1999 | Devlin, J. W. and Boleski, G. and Mlynarek, M. and Nerenz, D. R. and Peterson, E. and Jankowski, M. and Horst, H. M. and Zarowitz, B. J. |
| 1097 | Wrong outcome | The mountable unit stretcher extension rack (MOUNSTER) of the AZ-Vub, Brussels, Belgium | 2004 | Danschutter, D. |
| 1098 | Wrong outcome | A multi-layer monitoring system for clinical management of Congestive Heart Failure | 2015 | Guidi, G. and Pollonini, L. and Dacso, C. C. and Iadanza, E. |
| 1099 | Wrong outcome | Multi-parameter vital sign database to assist in alarm optimization for general care units | 2016 | Welch, J. and Kanter, B. and Skora, B. and McCombie, S. and Henry, I. and McCombie, D. and Kennedy, R. and Soller, B. |
| 1100 | Wrong outcome | A multicenter, cross-sectional quality improvement project: The perioperative implementation of a hypertension protocol by anesthesiologists | 2020 | Pfister, C. L. and Govender, S. and Dyer, R. A. and Rayner, B. and Flint, M. and Roodt, F. and Davids, J. and Nejthardt, M. B. and Swanevelder, J. L. and Chiu, C. J. E. and Cloete, E. and Koller, V. and Pretorius, T. and Fullerton, Z. and Roos, J. and van Zyl, R. and Biccard, B. M. |
| 1101 | Wrong outcome | A multicenter, multiyear study of the safety and clinical utility of esophagogastroduodenoscopy in 20 consecutive pregnant females with follow-up of fetal outcome | 1993 | Cappell, M. S. and Sidhom, O. |
| 1102 | Wrong outcome | Multicentre paired non-inferiority study of the cardiorespiratory monitoring performance of the wireless and non-adhesive BambiÂ® belt measuring diaphragm activity in neonates: Study protocol | 2022 | Scholten, A. W. J. and Zhan, Z. and Niemarkt, H. J. and Vervoorn, M. and Van Leuteren, R. W. and De Jongh, F. H. and Van Kaam, A. H. and Van Den Heuvel, E. R. and Jeroen Hutten, G. |
| 1103 | Wrong population | Multicentre validation of the bedside paediatric early warning system score: a severity of illness score to detect evolving critical illness in hospitalised children | 2011 | Parshuram, Christopher S. and Duncan, Heather P. and Joffe, Ari R. and Farrell, Catherine A. and Lacroix, Jacques R. and Middaugh, Kristen L. and Hutchison, James S. and Wensley, David and Blanchard, Nadeene and Beyene, Joseph and Parkin, Patricia C. |
| 1104 | Wrong outcome | Multidimensional reaction to therapeutic touch in a hospital setting | 1979 | Whitcher, S. J. and Fisher, J. D. |
| 1105 | Wrong outcome | Multidisciplinary Smartphone-Based Interventions to Empower Patients With Acute Coronary Syndromes: Qualitative Study on Health Care Providers' Perspectives | 2018 | Bashi, N. and Hassanzadeh, H. and Varnfield, M. and Wee, Y. and Walters, D. and Karunanithi, M. |
| 1106 | Wrong outcome | Multidisciplinary, three-dimensional and individualized comprehensive treatment for severe/critical COVID-19 | 2020 |  |
| 1107 | Wrong outcome | A multifactorial intervention to improve blood pressure control in co-existing diabetes and kidney disease: a feasibility randomized controlled trial | 2012 | Williams, Allison and Manias, Elizabeth and Walker, Rowan and Gorelik, Alexandra |
| 1108 | Wrong outcome | Multifunctional Nursing Beds Based on Intelligent Detection and Recovery | 2016 | Jiang, J. and Pan, X. and Jiang, X. and Yan, Z. |
| 1109 | Wrong outcome | A multimodal sensor dataset for continuous stress detection of nurses in a hospital | 2022 | Hosseini, S. and Gottumukkala, R. and Katragadda, S. and Bhupatiraju, R. T. and Ashkar, Z. and Borst, C. W. and Cochran, K. |
| 1110 | Wrong outcome | Multiparameter Intelligent Monitoring in Intensive Care II: a public-access intensive care unit database | 2011 | Saeed, M. and Villarroel, M. and Reisner, A. T. and Clifford, G. and Lehman, L. W. and Moody, G. and Heldt, T. and Kyaw, T. H. and Moody, B. and Mark, R. G. |
| 1111 | Wrong outcome | A MULTIPLE PATIENT MONITOR FOR INTENSIVE CARE OF RECOVERY ROOM | 1963 | Johnston, E. B. and Harper, R. B. |
| 1112 | Wrong outcome | Musculoskeletal Disorders and Psychosocial Stress at Work: A Comprehensive Study on Indian Nurses | 2024 | Chowdhury, Upasana and Das, Tama and Mazumder, Sahana and Gangopadhyay, Somnath |
| 1113 | Wrong outcome | Music to reduce stress in hospitalized patients | 2021 | Miller, C. R. and Patmon, F. L. and Knapp, H. |
| 1114 | Wrong outcome | Music to relieve pain and anxiety in cardiac catheterization: A systematic review and meta-analysis | 2024 | Santos, Kvgd and Dantas, Jkds and Fernandes, T. E. L. and Medeiros, K. S. and Sarmento, A. C. A. and Ribeiro, K. R. B. and Dantas, D. V. and Dantas, R. A. N. |
| 1115 | Wrong population | N-BiPAP vs n-CPAP in term neonate with respiratory distress syndrome | 2020 | Cimino, C. and Saporito, M. A. N. and Vitaliti, G. and Pavone, P. and Mauceri, L. and Gitto, E. and Corsello, G. and Lubrano, R. and Falsaperla, R. |
| 1116 | Wrong outcome | Narcotic use in the hospital: Reasonably safe? | 1992 | Whipple, J. K. and Ausman, R. K. and Quebbeman, E. J. |
| 1117 | Wrong population | A narrative review of thermoregulation techniques used by paediatric theatre staff during intra hospital transfer from paediatric theatres to the Neonatal Intensive Care Unit (NICU) | 2020 | Struzik, Sarah and Dow, Angela |
| 1118 | Wrong outcome | National Early Warning Score: A survey of registered nurses' perceptions, experiences and barriers | 2020 | SpÃ¥ngfors, M. and Molt, M. and Samuelson, K. |
| 1119 | Wrong outcome | National Survey: How Do We Approach the Patient at Risk of Clinical Deterioration outside the ICU in the Spanish Context? | 2022 | Clemente Vivancos, Ã and LeÃ³n Castelao, E. and Castellanos Ortega, Ã and Bodi Saera, M. and Gordo Vidal, F. and Martin Delgado, M. C. and Jorge-Soto, C. and Fernandez Mendez, F. and IgeÃ±o Cano, J. C. and Trenado Alvarez, J. and Caballero Lopez, J. and Parraga Ramirez, M. J. |
| 1120 | Wrong outcome | Near-infrared Spectroscopy in Transport With a Patient in Multi-factorial Shock | 2019 | Parker, J. and Walenta, T. and Turner-Nelson, K. |
| 1121 | Wrong population | The needs of young people with disabilities in transition from paediatric to adult services | 2005 | Chamberlain, M. A. and Kent, R. M. |
| 1122 | Wrong population | Neonatal axillary temperature measurements: a comparison of electronic thermometer predictive and monitor modes | 1999 | Fallis, W. M. and Christiani, P. |
| 1123 | Wrong population | Neonatal Cardiovascular, Respiratory, and Thermal Transition in the First Four Hours After Water Birth Compared to Water Labor and No Immersion | 2018 | Meyer, Shaunette Lin |
| 1124 | Wrong population | Neonatal CPAP for Respiratory Distress Across Malawi and Mortality | 2019 | Carns, J. and Kawaza, K. and Liaghati-Mobarhan, S. and Asibon, A. and Quinn, M. K. and Chalira, A. and Lufesi, N. and Molyneux, E. and Oden, M. and Richards-Kortum, R. |
| 1125 | Wrong outcome | The "Nephrology outpatient Triage": an organizational model for the ambulatory care of patients with advanced renal disease | 2018 | Soragna, G. and Bermond, F. and Fabbrini, L. and Rodofili, A. and Soragna, A. and Bauducco, M. and Panunzi, A. and Ramondetti, A. and Cerri, C. and Vitale, C. |
| 1126 | Wrong outcome | A new digital health tool for the telemonitoring of patients with scleroderma during iloprost administration: a feasibility and acceptability study | 2023 | Faggioli, P. and Zaccara, E. and Castelnovo, L. and Bompane, D. and Tamburello, A. and Lurati, A. and Laria, A. and Gangemi, D. and Giani, M. and Gnani, D. and Di Giorgi, M. and Iura, K. and Grandelis, F. and Piazza, R. and Piana, T. and Zizzo, G. and Mazzone, A. |
| 1127 | Wrong outcome | A new model for home care for COPD | 2004 | Alonso, A. |
| 1128 | Wrong outcome | A new soft tissue constructed with chitosan for wound dressings-incorporating nanoparticles for medical and nursing therapeutic efficacy | 2023 | Yang, M. and Wang, H. and Li, K. and Chen, Z. and Seamirumi, D. T. |
| 1129 | Wrong outcome | Newly qualified Saudi nurses' ability to recognize the deteriorating child in hospital | 2019 | Al-Thubaity, D. and Williamson, S. and Leavey, R. and Tume, L. N. |
| 1130 | Wrong outcome | The Next Frontier of Remote Patient Monitoring: Hospital at Home | 2023 | Whitehead, D. and Conley, J. |
| 1131 | Wrong outcome | NICE calls for tighter checks on vital signs | 2007 | Lomas, C. |
| 1132 | Wrong outcome | NIPH Systematic Reviews: Executive Summaries | 2009 | Holte, T. O. and Vandvik, P. O. and Elvsaas, IkÃ˜ and Norderhaug, I. N. |
| 1133 | Wrong outcome | No cause for alarm: Decreasing inappropriate pulse oximetry use in bronchiolitis | 2018 | Heneghan, M. and Hart, J. and Dewan, M. and Wu, K. and Hope, K. and Taylor, A. and Shaw, K. and Bamat, T. |
| 1134 | Wrong outcome | No need for pain | 2000 | Sayers, M. and Marando, R. and Fisher, S. and Aquila, A. and Morrison, B. and Dailey, T. |
| 1135 | Wrong outcome | [No response to antihypertensive therapy: consider non-adherence] | 2007 | Braam, R. L. and Van Uum, S. H. and Lenders, J. W. and Thien, T. |
| 1136 | Wrong population | Non-comorbid Respiratory Factor and Work of Breathing in Pediatric COVID-19 Patient: How is Their Synergistic Correlation with the Level of Care? | 2022 | Efendi, Defi and Kurniasari, Maria Dyah and Huda, Mega Hasanul and Farid, Raudha Ilmi and Rias, Yohanes Andy and Prawira, Yogi and Putri, Nina Dwi and Utami, Ayuni Rizka and Asmarini, Titik Ambar and Lestari, Pande Lilik and Mais, Pricilia and Babakal, Abram |
| 1137 | Wrong outcome | Non-contact physiological monitoring of post-operative patients in the intensive care unit | 2022 | Jorge, J. and Villarroel, M. and Tomlinson, H. and Gibson, O. and Darbyshire, J. L. and Ede, J. and Harford, M. and Young, J. D. and Tarassenko, L. and Watkinson, P. |
| 1138 | Wrong outcome | Non-contact screening system based for COVID-19 on XGBoost and logistic regression | 2022 | Dong, C. and Qiao, Y. and Shang, C. and Liao, X. and Yuan, X. and Cheng, Q. and Li, Y. and Zhang, J. and Wang, Y. and Chen, Y. and Ge, Q. and Bao, Y. |
| 1139 | Wrong outcome | Non-infectious hyperthermia in acute brain injury patients: Relationships to mortality, blood pressure, intracranial pressure and cerebral perfusion pressure | 2012 | Oh, Hyun Soo and Jeong, Hye Sun and Seo, Wha Sook |
| 1140 | Wrong outcome | Non-Invasive Core Temperature Monitoring System: Providing Consistent, Reliable Temperature Measurement in the Perianesthesia Patient...American Society of PeriAnesthesia Nurses 40th National Conference, 25-29 April, 2021 | 2021 | Brooks, Team Leader Cristina and Matulewicz, Team Member Sarah Bosserman |
| 1141 | Wrong outcome | Non-invasive monitoring of core body temperature rhythms over 72h in 10 bedridden elderly patients with disorders of consciousness in a Japanese hospital: A pilot study | 2013 | Matsumoto, M. and Sugama, J. and Okuwa, M. and Dai, M. and Matsuo, J. and Sanada, H. |
| 1142 | Wrong outcome | Non-invasive ventilation improves comfort in pediatric palliative care patients | 2014 | Bosch-Alcaraz, A. |
| 1143 | Wrong outcome | Non-invasive wearable seizure detection using long-short-term memory networks with transfer learning | 2021 | Nasseri, M. and Pal Attia, T. and Joseph, B. and Gregg, N. M. and Nurse, E. S. and Viana, P. F. and Schulze-Bonhage, A. and DÃ¼mpelmann, M. and Worrell, G. and Freestone, D. R. and Richardson, M. P. and Brinkmann, B. H. |
| 1144 | Wrong outcome | Noninvasive blood pressure monitoring | 2002 | Dobbin, K. R. |
| 1145 | Wrong population | Nonlinearity of heart rate in the neonate | 1995 | Allen, C. E. and Menke, J. A. and Hayes, J. |
| 1146 | Wrong outcome | [Not Available] | 2005 | de Jong, J. W. and Ekkerink, J. L. and Touma, I. and Koopmans, R. T. |
| 1147 | Wrong outcome | A Novel Method of Clinical Nursing under the Medical Internet of Things Technology | 2021 | Ou, T. and Cai, X. and Wang, M. and Guo, F. and Wu, B. |
| 1148 | Wrong outcome | A Novel Model of Patientâ€™s Health Monitoring System Using IoT Platform | 2022 | Lakshminarayanan, V. and Thirunavukkarasu, M. and Suganya, A. |
| 1149 | Wrong outcome | A Novel Non-contact Self-Injection-Locked Radar for Vital Sign Sensing and Body Movement Monitoring in COVID-19 Isolation Ward | 2020 | Tsai, C. Y. and Chang, N. C. and Fang, H. C. and Chen, Y. C. and Lee, S. S. |
| 1150 | Wrong outcome | A Novel Noninvasive Device to Assess Sympathetic Nervous System Function in Patients With Heart Failure | 2015 | Parry, M. and Nielson, C. A. and Muckle, F. and O'Keefe-McCarthy, S. and van Lien, R. and Meijer, J. H. |
| 1151 | Wrong outcome | A novel predictive analytics score reflecting accumulating disease burden-an investigation of the cumulative CoMET score | 2023 | Monfredi, O. and Andris, R. T. and Lake, D. E. and Moorman, J. R. |
| 1152 | Wrong outcome | Novel technologies can provide effective dressing and securement for peripheral arterial catheters: A pilot randomised controlled trial in the operating theatre and the intensive care unit | 2015 | Reynolds, H. and Taraporewalla, K. and Tower, M. and Mihala, G. and Tuffaha, H. W. and Fraser, J. F. and Rickard, C. M. |
| 1153 | Wrong outcome | Nurse Capacity to Rescue in an Empowered Work Environment | 2018 | Stacey Scime, Anastasia |
| 1154 | Wrong outcome | The nurse care of cardiovascular health in hypertense [sic] women | 2009 | de Assis, L. S. and Stipp, M. A. C. and Leite, J. L. and da Cunha, N. M. |
| 1155 | Wrong outcome | Nurse decision making in the prearrest period | 2010 | Gazarian, P. K. and Henneman, E. A. and Chandler, G. E. |
| 1156 | Wrong outcome | Nurse decision-making when managing noradrenaline in the intensive care unit: A naturalistic observational study | 2023 | Hunter, S. and Considine, J. and Manias, E. |
| 1157 | Wrong outcome | A Nurse Led Clinic's contribution to Patient Education and Promoting Self-care in Heart Failure Patients: A Systematic Review | 2017 | Walsh, Jacinta Clare |
| 1158 | Wrong outcome | Nurse led clinics controlled hypertension and hyperlipidaemia better than usual care in diabetes | 2004 | Carrier, J. A. K. |
| 1159 | Wrong outcome | Nurse productivity: using evidence to enhance nurses' use of time | 2024 | Morgan, S. |
| 1160 | Wrong population | Nurse Responses to Physiologic Monitor Alarms on a General Pediatric Unit | 2019 | Schondelmeyer, A. C. and Daraiseh, N. M. and Allison, B. and Acree, C. and Loechtenfeldt, A. M. and Timmons, K. M. and Mangeot, C. and Brady, P. W. |
| 1161 | Wrong outcome | Nurse-assessed metabolic monitoring: A file audit of risk factor prevalence and impact of an intervention to enhance measurement of waist circumference | 2014 | Rosenbaum, Simon and Nijjar, Sukh and Watkins, Andrew and Garwood, Natasha and Sherrington, Catherine and Tiedemann, Anne |
| 1162 | Wrong outcome | Nurse-coordinated multidisciplinary, family-based cardiovascular disease prevention programme (EUROACTION) for patients with coronary heart disease and asymptomatic individuals at high risk of cardiovascular disease: A paired, cluster-randomised controlled trial | 2008 | Wood, D. A. and Kotseva, K. and Connolly, S. and Jennings, C. and Mead, A. and Jones, J. et al |
| 1163 | Wrong outcome | A nurse-led coaching intervention with home telemonitoring for patients with heart failure: Protocol for a feasibility randomized clinical trial | 2024 | Basso, I. and Bassi, E. and Caristia, S. and Durante, A. and Vairo, C. and Patti, S. G. R. and Pirisi, M. and Campanini, M. and Invernizzi, M. and Bellan, M. and Dal Molin, A. and Caldera, F. and D'Amario, D. and Dell'Era, G. and Gomez, I. and Lazzati, A. and Massara, E. and Milanese, C. and Molon, A. and Petteneo, M. and Scaramuzzino, S. and Torgano, C. and Zumbo, P. |
| 1164 | Wrong outcome | Nurse-led disease management for hypertension control in a diverse urban community: a randomized trial | 2012 | Hebert, P. L. and Sisk, J. E. and Tuzzio, L. and Casabianca, J. M. and Pogue, V. A. and Wang, J. J. and Chen, Y. and Cowles, C. and McLaughlin, M. A. |
| 1165 | Wrong outcome | Nurse-led disease management for hypertension control in a diverse urban community: a randomized trial | 2012 | Hebert, P. L. and Sisk, J. E. and Tuzzio, L. and Casabianca, J. M. and Pogue, V. A. and Wang, J. J. and Chen, Y. and Cowles, C. and McLaughlin, M. A. and Hebert, Paul L. and Sisk, Jane E. and Tuzzio, Leah and Casabianca, Jodi M. and Pogue, Velvie A. and Wang, Jason J. and Chen, Yingchun and Cowles, Christine and McLaughlin, Mary Ann |
| 1166 | Wrong outcome | Nurse-led hypertension referral system in an emergency department for asymptomatic elevated blood pressure | 2012 | Tsoi, L. C. and Tung, C. C. and Wong, E. L. |
| 1167 | Wrong outcome | A nurse-led multicomponent intervention supported by advanced electronic health records to improve the acute management of stroke patients: A pre- and post-intervention study | 2021 | Dello, S. and Lemmens, R. and Demeestere, J. and Michiels, D. and Wellens, L. and Weltens, C. and Vanhaecht, K. and Bruyneel, L. |
| 1168 | Wrong outcome | Nurse-Led Vital Signs Monitoring Can Safely Identify Low-Risk Hematology-Oncology Patients For De-Escalation | 2020 | Tham, S. M. and Kasinathan, S. and Lui, P. L. and Lim, B. K. and Yap, R. A. P. and Montanez, F. B. and Ow, S. G. W. |
| 1169 | Wrong outcome | Nurse-managed protocols for early identification of sepsis: a scoping review | 2023 | de MendonÃ§a Henrique, Danielle and Costa, Beatriz da Silva Rodrigues and Fassarella, Cintia Silva and Camerini, Flavia Giron and Silva, Renata FlÃ¡via Abreu da and de Oliveira Silva, Julia Leonidia |
| 1170 | Wrong outcome | NURSES ON KNOWLEDGE VASOACTIVE DRUGS USED IN CRITICAL PATIENTS | 2016 | Mesquita Melo, Elizabeth and da Penha Oliveira Cavalcante, HerlÃªnia and Mota Marques, Aline and MagalhÃ£es Ferreira, Andreza Moura and Ferreira de Abreu, Maria Alana and Frota Lima, Violeta and Santos Garces, Thiago |
| 1171 | Wrong outcome | Nurses' 12-hour shifts and missed or delayed vital signs observations on hospital wards: retrospective observational study | 2019 | Dall'Ora, C. and Griffiths, P. and Redfern, O. and Recio-Saucedo, A. and Meredith, P. and Ball, J. |
| 1172 | Wrong outcome | Nurses' attitudes toward conventional and automated vital signs measurement methods | 1988 | Campbell-Heider, N. and Knapp, T. R. |
| 1173 | Wrong outcome | Nurses' experience with patient deterioration and rapid response teams | 2024 | Ruiz, C. and Golec, K. and Vonderheid, S. C. |
| 1174 | Wrong outcome | Nurses' knowledge of error in blood pressure measurement technique | 2002 | Armstrong, R. S. |
| 1175 | Wrong outcome | Nurses' knowledge of heart failure education principles | 2002 | Albert, N. M. and Collier, S. and Sumodi, V. and Wilkinson, S. and Hammel, J. P. and Vopat, L. and Willis, C. and Bittel, B. |
| 1176 | Wrong outcome | Nurses' knowledge of heart failure education topics as reported in a small midwestern community hospital | 2005 | Washburn, S. C. and Hornberger, C. A. and Klutman, A. and Skinner, L. |
| 1177 | Wrong outcome | Nurses' knowledge, perceived challenges, and recommended solutions regarding premature infant care: A mixed method study in the referral and tertiary hospitals in Dar es salaam, Tanzania | 2023 | Mwikali, M. and Salim, N. and Sylvester, I. and Munubhi, E. |
| 1178 | Wrong outcome | Nurses' perceptions of caring activities in nursing | 2021 | Akansel, N. and Watson, R. and Vatansever, N. and Ã–zdemir, A. |
| 1179 | Wrong outcome | Nurses' Perceptions of the Application of the Internet of Things in Healthcare Services in Indonesia: A Mixed Methods Study | 2021 | Lindayani, Linlin and Taryudi and Darmawati, Irma |
| 1180 | Wrong outcome | Nurses' practice of metabolic monitoring for patients on antipsychotics in Lesotho | 2024 | Matete, R. P. and Rathobei, L. M. |
| 1181 | Wrong outcome | Nurses' response to frequency and types of electrocardiography alarms in a non-critical care setting: A descriptive study | 2014 | Gazarian, Priscilla K. |
| 1182 | Wrong outcome | Nurses' responses to monitor alarms in an intensive care unit: An observational study | 2020 | Dursun Ergezen, F. and Kol, E. |
| 1183 | Wrong outcome | Nurses' role in recognising and responding to clinical deterioration in surgical patients | 2018 | Mohammmed Iddrisu, Suad and Hutchinson, Ana F. and Sungkar, Yasmin and Considine, Julie |
| 1184 | Wrong outcome | Nursing actions in the perioperative period and in preparing prostatectomy patients for discharge | 2013 | da Mata, Luciana Regina Ferreira and Ferreira, Taciana Caldas and de Carvalho, Emilia Campos |
| 1185 | Wrong outcome | Nursing and practices that speed up healing in heart surgery | 2015 | Koyuncu, A. and Eti Aslan, F. and Karabacak, U. and DemirkiliÃ§, U. |
| 1186 | Wrong outcome | Nursing assessment of continuous vital sign surveillance to improve patient safety on the medical/surgical unit | 2016 | Watkins, T. and Whisman, L. and Booker, P. |
| 1187 | Wrong outcome | Nursing assistance for spring coil occlusion for the treatment of intracranial giant internal carotid artery aneurysms | 2010 | Ma, Y. G. and Mao, Y. J. and Yuan, Y. L. and Hu, Y. Q. and Liu, J. and Xi, J. |
| 1188 | Wrong outcome | Nursing care activities based on documentation | 2019 | Asmirajanti, M. and Hamid, A. Y. S. and Hariyati, R. T. S. |
| 1189 | Wrong outcome | Nursing Care and Treatment of Ambulatory Patients With Percutaneously Placed Axillary Intra-aortic Balloon Pump Before Heart Transplant | 2019 | Macapagal, Frederick R. and McClellan, Emma and Macapagal, Rosario O. and Green, Lisa and Bonuel, Nena |
| 1190 | Wrong population | Nursing care for patients receiving percutaneous antegrade ureteral stent implantation for ureteral stricture | 2017 | Xu, Y. and Yang, R. and Gu, M. and Wang, Z. |
| 1191 | Wrong outcome | Nursing care for the severely obese patient | 1982 | Vaughan, M. S. |
| 1192 | Wrong outcome | Nursing Care in Intensive Care Unit of a Patient Infected With Balamuthia Mandrillaris After Renal Transplantation: A Case Report | 2024 | Qin, S. and Lu, X. and Li, L. and Huang, D. |
| 1193 | Wrong outcome | Nursing Care in Lebanon: A Nursing Perspective | 2011 | Badr, Lina Kurdahi and Abdallah, Bahia and Purdy, Isabell B. |
| 1194 | Wrong outcome | Nursing care of a patient with sarcoidosis. Case study using ICNPÂ® | 2019 | BrzeÅºnicki, Jakub and Grabowska, Hanna |
| 1195 | Wrong outcome | Nursing care of hospitalised patients receiving mechanical ventilation in intensive care units | 2014 | Mesquita Melo, Elizabeth and Santos Teixeira, Carlos and Terto de Oliveira, RogÃ©ria and Teixeira de Almeida, Diva and Eline Gomes Lacerda de Freitas Veras, Joelna and Marques Frota, Natasha and Borges Studart, Rita MÃ´nica |
| 1196 | Wrong outcome | Nursing care of patients with relapsed and refractory multiple myeloma treated with B-cell mature antigen-targeted universal chimeric antigen receptor T cells | 2023 | Dai, Y. and Tang, F. and Mao, Y. and He, N. and Yu, M. and Zhang, M. and Gu, S. and Lu, Y. and Shang, J. and Zhu, X. |
| 1197 | Wrong outcome | Nursing care of the ambulatory patient with a mechanical assist device | 1990 | Reedy, J. E. and Ruzevich, S. A. and Noedel, N. R. and Vitale, L. J. and Merkle, E. J. |
| 1198 | Wrong outcome | Nursing care of transradial angiography and intervention in a tertiary hospital in Shanghai: a best practice implementation project | 2016 | Li, Zhu and Jianming, Xu and Qibing, Wang and Fei, Xu and Jun, Chen |
| 1199 | Wrong outcome | Nursing care of transradial angiography and intervention in a tertiary hospital in shanghai: A best practice implementation project | 2016 | Zhu, L. and Xu, J. and Wang, Q. and Xu, F. and Chen, J. |
| 1200 | Wrong outcome | Nursing documentation prior to emergency admissions to the intensive care unit | 2011 | Jonsson, Thorsteinn and Jonsdottir, Helga and MÃ¶ller, Alma D. and Baldursdottir, LovÃ­sa |
| 1201 | Wrong outcome | Nursing evaluation during treatment with helmet continuous positive airway pressure in patients with respiratory failure due to COVID-19 pneumonia: A case series | 2022 | Privitera, D. and Capsoni, N. and Mazzone, A. and Airoldi, C. and Angaroni, L. and Pierotti, F. and Rocca, E. and Dal Molin, A. and Bellone, A. |
| 1202 | Wrong outcome | Nursing Experience in Allogeneic Hematopoietic Stem Cell Transplantation for Acute Lymphoblastic Leukemia | 2024 | Wu, X. and Chao, J. and Xu, Q. |
| 1203 | Wrong outcome | Nursing Experience of Awake Prone Position in a Severe COVID-19 Case | 2023 | Yi-Chien, Chen and Ling-Fen, Chen |
| 1204 | Wrong outcome | Nursing Experience of Caring for a Patient With COVID-19 During Isolation | 2020 | Tsang-Ching, H. S. U. and Chia-Chien, W. U. and Pei-Yu, L. A. I. and Ling-Shan, Syue and Yi-Yin, L. A. I. and Nai-Ying, K. O. |
| 1205 | Wrong outcome | [Nursing Experience of Caring for a Patient With COVID-19 During Isolation] | 2020 | Hsu, T. C. and Wu, C. C. and Lai, P. Y. and Syue, L. S. and Lai, Y. Y. and Ko, N. Y. |
| 1206 | Wrong outcome | Nursing implications of an early warning system implemented to reduce adverse events: a qualitative study | 2022 | Braun, E. J. and Singh, S. and Penlesky, A. C. and Strong, E. A. and Holt, J. M. and Fletcher, K. E. and Stadler, M. E. and Nattinger, A. B. and Crotty, B. H. |
| 1207 | Wrong outcome | Nursing in pre-hospital care for burn victims: a scoping review | 2021 | Medeiros da Silva, RosivÃ¢nia Ingrid and dos Santos Oliveira, Eloysa and Andrade Rocha, Rodrigo Rhuan and de Souza Costa, Thatiane Monick and Neves Dantas, Rodrigo Assis and Vieira Dantas, Daniele |
| 1208 | Wrong population | [Nursing intervention in a child diagnosed with ineffective airway clearance: a case report] | 2009 | Pereira-de-Melo, R. and Arrais-Sampaio, F. A. and de Oliveira-Lopes, M. V. |
| 1209 | Wrong outcome | Nursing interventions for patients with COVID-19: A medical record review and nursing interventions classification study | 2022 | Asghari, E. and Archibald, M. and Roshangar, F. |
| 1210 | Wrong outcome | Nursing interventions for the early detection of ward patients' clinical deterioration: an integrative review | 2017 | Moura Freitas, Catarina and PolÃ³nio Preto, EmÃ­dio and Fernandes Nascimento, Carla Alexandra |
| 1211 | Wrong outcome | Nursing Interventions to Prevent Secondary Injury in Critically Ill Patients with Traumatic Brain Injury: A Scoping Review | 2024 | Figueiredo, R. and Castro, C. and Fernandes, J. B. |
| 1212 | Wrong outcome | Nursing logs for blood transfusion | 2004 | Quero la Rosa, F. and Soria Grande, A. I. and Palencia Herranz, MÃ |
| 1213 | Wrong outcome | Nursing monitoring and management of free and pedicled flaps--outcomes of teaching sessions on flap care | 2010 | Khan, M. A. and Mohan, A. and Ahmed, W. and Rayatt, S. |
| 1214 | Wrong outcome | Nursing observations during the first 24 hours after a surgical procedure: what do we do? | 2005 | Zeitz, K. |
| 1215 | Wrong outcome | Nursing observations on ward patients at risk of critical illness | 2002 | Chellel, A. and Fraser, J. and Fender, V. and Higgs, D. and Buras-Rees, S. and Hook, L. and Mummery, L. and Cook, C. and Parsons, S. and Thomas, C. |
| 1216 | Wrong outcome | The nursing perspective on monitoring hemodynamics and oxygen transport | 2011 | Tucker, D. and Hazinski, M. F. |
| 1217 | Wrong population | [Nursing practice in maternity intensive care units. Severe pre-eclampsia in a primigravida] | 2015 | Carmona-Guirado, A. J. and EscaÃ±o-Cardona, V. and GarcÃ­a-CaÃ±edo, F. J. |
| 1218 | Wrong outcome | The nursing practice of taking level 1 patient observations | 2006 | Wheatley, I. |
| 1219 | Wrong outcome | Nursing research on patients with hypertensive intracerebral hemorrhage based on fuzzy neural network related algorithm under postural adjustment | 2020 | Qu, D. and Ding, C. |
| 1220 | Wrong Study Design | Nursing staff perspectives of continuous remote vital signs monitoring on surgical wards: Theory elicitation for a realist evaluation | 2022 | Downey, C. and Brown, J. and Jayne, D. and Randell, R. |
| 1221 | Wrong outcome | Nursing strategies for the mechanically ventilated patient | 2023 | Meitner, C. and Feuerstein, R. A. and Steele, A. M. |
| 1222 | Wrong outcome | Nursing stress and patient care: real-time investigation of the effect of nursing tasks and demands on psychological stress, physiological stress, and job performance: study protocol | 2013 | Farquharson, B. and Bell, C. and Johnston, D. and Jones, M. and Schofield, P. and Allan, J. and Ricketts, I. and Morrison, K. and Johnston, M. |
| 1223 | Wrong outcome | Nursing surveillance moderates the relationship between staffing levels and pediatric postoperative serious adverse events: A nested case-control study | 2013 | Voepel-Lewis, Terri and Pechlavanidis, Elsa and Burke, Constance |
| 1224 | Wrong outcome | Nursing's renaissance: an innovative continuum of care takes nurses back to their roots | 1993 | Hey, M. |
| 1225 | Wrong outcome | [Observation of critically ill patients] | 2009 | Fuhrmann, L. and Hesselfeldt, R. and Lippert, A. and Perner, A. and Ostergaard, D. |
| 1226 | Wrong population | An observational study of monitoring of vital signs in children admitted to Kenyan hospitals: an insight into the quality of nursing care? | 2018 | Ogero, M. and Ayieko, P. and Makone, B. and Julius, T. and Malla, L. and Oliwa, J. and Irimu, G. and English, M. |
| 1227 | Wrong outcome | An observational study of older patient specialling in acute hospital settings | 2020 | Cook, Jacquelene and Palesy, Debra and Lapkin, Samuel and Chenoweth, Lynn |
| 1228 | Wrong outcome | Observational study using video recordings to explore the first hour after admission to a neonatal intensive care unit | 2021 | Aydon, L. and Gill, A. and Zimmer, M. and Sharp, M. and Woods, P. and Seeber, C. and Mooney, D. and Murdoch, J. |
| 1229 | Wrong outcome | Observations and monitoring: routine practices on the ward | 2010 | Oliver, A. and Powell, C. and Edwards, D. and Mason, B. |
| 1230 | Wrong outcome | Observations and vital signs: ritual or vital for the monitoring of postoperative patients? | 2006 | Zeitz, K. and McCutcheon, H. |
| 1231 | Wrong outcome | Observations using antiendotoxin antibody (E5) as adjuvant therapy in humans with suspected, serious, Gram-negative sepsis | 1992 | Greenberg, R. N. and Wilson, K. M. and Kunz, A. Y. and Wedel, N. I. and Gorelick, K. J. |
| 1232 | Wrong outcome | [Obstetric analgesia in German clinics. Remifentanil as alternative to regional analgesia] | 2011 | Schnabel, A. and Hahn, N. and Muellenbach, R. and Frambach, T. and Hoenig, A. and Roewer, N. and Kranke, P. |
| 1233 | Wrong outcome | Occurrence of hypothermia in a prehospital setting, southern Sweden | 2010 | KornfÃ¤lt, J. and Johansson, A. |
| 1234 | Wrong outcome | Open abdomen treatment following endovascular repair of ruptured abdominal aortic aneurysms | 2009 | Mayer, D. and Rancic, Z. and Meier, C. and Pfammatter, T. and Veith, F. J. and Lachat, M. |
| 1235 | Wrong outcome | The opinions of nurses on the care of the patients undergoing endovascular peripheral vascular intervention | 2018 | Durmaz Edeer, A. and MÃ¼ezziÌ‡Nler EvsiÌ‡Ne, N. and Kara, S. |
| 1236 | Wrong outcome | Opportunistic influenza vaccination in the home: broadening access in isolated times | 2021 | Nisbet, L. C. and Cobbledick, A. M. and Smith, T. E. and Bryant, P. A. and Lawrence, J. |
| 1237 | Wrong outcome | Optimal nursing strategy for patients receiving basilar artery angioplasty | 2014 | Sun, F. Z. and Chen, J. and Xiang, L. and Du, B. |
| 1238 | Wrong population | Optimising homeothermy in neonates: A systematic review and clinical guidelines from the French Neonatal Society | 2022 | Tourneux, P. and Thiriez, G. and Renesme, L. and Zores, C. and Sizun, J. and Kuhn, P. and Allen, A. and Audeoud, F. and Bouvard, C. and Brandicourt, A. and Caeymaex, L. and Duboz, M. A. and Evrard, A. and Fichtner, C. and Fischer-Fumeaux, C. and Girard, L. and Gonnaud, F. and HÃ¼ppi, P. and Knezovic, N. and Kuhn, P. and Laprugne-Garcia, E. and Legouais, S. and Mons, F. and Muller, J. B. and Picaud, J. C. and Pierrat, V. and Pladys, P. and Reynaud, A. and Renesme, L. and Rideau, A. and Sizun, J. and Souet, G. and Thiriez, G. and Tourneux, P. and Touzet, M. and Truffert, P. and Tscherning, C. and Zaoui, C. and Zana-Taieb, E. |
| 1239 | Wrong outcome | Optimizing intensive care in stroke: A European perspective. A report of an Ad Hoc Consensus Group Meeting | 1997 | Hacke, W. |
| 1240 | Wrong outcome | Optimizing the response to mental health deterioration: Nurses experiences of using a Mental Health Observation Response Chart | 2023 | Forster, John A. and Coventry, Alysia A. and Daniel, Catherine |
| 1241 | Wrong outcome | Oral midazolam with low dose ketamine, fentanyl, or ketoprofen for the prevention of emergence agitation after pediatric ambulatory surgery | 2008 | Shaban M, M. and Asida, S. M. |
| 1242 | Wrong outcome | Organisation, staffing and resources of critical care units in Kenya | 2023 | Mwangi, W. and Kaddu, R. and Njoki Muiru, C. and Simiyu, N. and Patel, V. and Sulemanji, D. and Otieno, D. and Okelo, S. and Chikophe, I. and Pisani, L. and Dona, D. P. G. and Beane, A. and Haniffa, R. and Misango, D. and Waweru-Siika, W. |
| 1243 | Wrong outcome | ORGANIZATION OF INTENSIVE CARE IN SLOVENIA | 2017 | Pareznik, R. |
| 1244 | Wrong outcome | Organizational aspects of implementation the technology of on-line dispensary observation in patients with arterial hypertension | 2020 | Strokolskaya, I. L. and Kilizhekova, D. V. and Makarov, S. A. |
| 1245 | Wrong outcome | OS035. Blood pressure measurement by health professionals, comparison with American Heart Association Technique | 2012 | Sahbaeiroy, F. and Pourzadi, M. and Hasani, M. M. and Khatibi, N. and Esmailpour, S. |
| 1246 | Wrong population | Outborn newborns drive birth asphyxia mortality rates-An 8 year analysis at a rural level two nursery in Uganda | 2023 | Hedstrom, A. and Nyonyintono, J. and Mubiri, P. and Namakula Mirembe, H. and Magnusson, B. and Nakakande, J. and MacGuffie, M. and Nsubuga, M. and Waiswa, P. and Nambuya, H. and Batra, M. |
| 1247 | Wrong outcome | Outcomes of an advanced practice nurse-led type-2 diabetes support group | 2011 | Partiprajak, Suphamas and Hanucharurnkul, Somchit and Piaseu, Noppawan and Brooten, Dorothy and Nityasuddhi, Dechavudh |
| 1248 | Wrong outcome | [Outcomes of home monitoring after palliative cardiac surgery in infants with congenital heart disease] | 2014 | Kim, S. W. and Uhm, J. Y. and Im, Y. M. and Yun, T. J. and Park, J. J. and Park, C. S. |
| 1249 | Wrong outcome | Outpatient cataract surgery: incident and procedural risk analysis do not support current clinical ophthalmology guidelines | 2015 | Koolwijk, J. and Fick, M. and Selles, C. and Turgut, G. and Noordergraaf, J. I. and Tukkers, F. S. and Noordergraaf, G. J. |
| 1250 | Wrong outcome | Outstanding Practice--Podium: Evaluation of Continuous Multiparameter Surveillance Monitoring, a Wearable Medical Device, on Code Blue/Treat Team Events for Medical-Surgical Floor Patients | 2017 | Judson, Tonya and Holyfield, Ada |
| 1251 | Wrong outcome | Ownership conversions and nursing home performance | 2008 | Grabowski, D. C. and Stevenson, D. G. |
| 1252 | Wrong outcome | Oxygen efficient respiratory Aid (OxEraTM) device: A safety study | 2022 | John, M. T. and van Blydenstein, S. A. and Omar, S. and Bruins, J. and Tshukutsoane, S. |
| 1253 | Wrong outcome | Oxygen saturation and hemodynamic response in critically ill, mechanically ventilated adults during intrahospital transport | 1995 | Evans, A. and Winslow, E. H. |
| 1254 | Wrong outcome | Oxygen therapy: professional compliance with national guidelines | 2014 | Nippers, Ingrid and Sutton, Andrew |
| 1255 | Wrong population | Oxygen treatment of ventilated critically ill children: Nursing assessment and cognitive processes in clinical decision-making | 2020 | Soini, Ulla Marie and AndenÃ¦s, Randi and Solberg, Marianne Trygg |
| 1256 | Wrong outcome | P015. Implementing Telemedicine Practices for Mechanical Circulatory Support Patients During COVID-19 Global Pandemic...American Association of Heart Failure Nurses, 17th Annual Meeting (Virtual), 17-18 June, 2021 | 2021 | Dinicola, Beth |
| 1257 | Wrong outcome | PACU-why hand washing is vital! | 2009 | Petty, W. C. |
| 1258 | Wrong population | The paediatric early warning score | 2007 | Duncan, H. P. |
| 1259 | Wrong population | Paediatric early warning systems: where do we go from here? | 2009 | McCabe, A. and Duncan, H. and Heward, Y. |
| 1260 | Wrong population | Paediatric retrieval - Aiming for the gold standard | 2001 | Davies, J. |
| 1261 | Wrong population | Pain and sedation management and monitoring in pediatric intensive care units across Europe: an ESPNIC survey | 2022 | Daverio, M. and von Borell, F. and Ramelet, A. S. and Sperotto, F. and Pokorna, P. and Brenner, S. and Mondardini, M. C. and Tibboel, D. and Amigoni, A. and Ista, E. and Kola, E. and Vittinghoff, M. and Duval, E. and PoliÄ‡, B. and Valla, F. and Neunhoeffer, F. and Konstantinos, T. and GyÃ¶rgyi, Z. and Tan, M. H. and Hasani, A. and Poluzioroviene, E. and Balmaks, R. and Afanetti, M. and Bentsen, G. and Bartkowska-Sniatkowska, A. and Camilo, C. and Simic, D. and LÃ³pez-FernÃ¡ndez, Y. M. and Mattsson, J. and Ã–zen, H. and Dmytriiev, D. and Manning, J. C. and TekgÃ¼Ã§, H. |
| 1262 | Wrong outcome | Pain as the fifth vital sign: use of the assessment scale by nurses in general hospital | 2010 | Bottega, F. H. and Fontana, R. T. |
| 1263 | Wrong outcome | Pain in the intensive care unit: Nursing issues | 2015 | de Lattre, S. and de Jong, A. and Gniadek, C. and Carr, J. and Tondut, G. and Conseil, M. and CissÃ©, M. and Jaber, S. and Chanques, G. |
| 1264 | Wrong outcome | Pain Level, Influencing Factors and Applied Nursing Interventions in Patients Undergoing GI Surgery | 2016 | Rizalar, Selda and Ozbas, Ayfer |
| 1265 | Wrong outcome | Palliative care for endâ€ofâ€life patients in a basic emergency service | 2018 | Pereira, Maria Eduarda Diniz and Barbosa, AntÃ³nio and Dixe, Maria dos Anjos |
| 1266 | Wrong outcome | Parameters of Intra Aortic Balloon Pumps in Patients Undergoing Open Heart Surgery | 2023 | Uygur, Feragat and Guzel, Hatice and Tuna, Arzu |
| 1267 | Wrong outcome | The parameters that cardiothoracic intensive care nurses use to assess the progress or deterioration of their patients | 1999 | Norrie, P. |
| 1268 | Wrong outcome | Parents' and carers' experiences of transitions and aftercare following a child's discharge from a pediatric intensive care unit to an inpatient ward setting: a qualitative systematic review protocol | 2016 | Suleman, Zainab and Manning, Joseph C. and Evans, Catrin |
| 1269 | Wrong outcome | [Path Analysis for Delirium on Patient Prognosis in Intensive Care Units] | 2019 | Lee, S. and Lee, S. M. |
| 1270 | Wrong outcome | Pathway for the management of survivors of out-of-hospital cardiac arrest | 2010 | Herzog, E. and Shapiro, J. and Aziz, E. F. and Chong, J. and Hong, M. K. and Wiener, D. and Lee, R. and Janis, G. and Azrieli, Y. and Velazquez, B. and Lacdao, L. and Mittal, S. |
| 1271 | Wrong outcome | Patient acceptability of wearable vital sign monitoring technologies in the acute care setting: A systematic review | 2019 | Sprogis, S. K. and Currey, J. and Considine, J. |
| 1272 | Wrong outcome | Patient and Clinician Perceptions of the Pulse Oximeter in a Remote Monitoring Setting for COVID-19: Qualitative Study | 2023 | Torres-Robles, A. and Allison, K. and Poon, S. K. and Shaw, M. and Hutchings, O. and Britton, W. J. and Wilson, A. and Baysari, M. |
| 1273 | Wrong outcome | Patient and nurse experience of vital-sign monitoring practices and preliminary views of wearable monitoring: Qualitative study in a surgical ward...Physiotherapy UK Virtual Conference, November 5-6, 2021 | 2022 | Areia, C. and King, E. and Young, L. and Ede, J. and Tarassenko, L. and Watkinson, P. and Vollam, S. |
| 1274 | Wrong outcome | Patient monitoring using infrastructure-oriented wireless LANs | 2006 | Varshney, U. |
| 1275 | Wrong outcome | Patient safety culture, missed Nursing care and its reasons in Obstetrics | 2021 | Silva, S. C. D. and Morais, B. X. and Munhoz, O. L. and Ongaro, J. D. and Urbanetto, J. S. and Magnago, Tsbs |
| 1276 | Wrong outcome | Patient safety culture, missed Nursing care and its reasons in Obstetrics | 2021 | da Silva, Silvana Cruz and Morais, Bruna Xavier and Munhoz, Oclaris Lopes and Dal Ongaro, Juliana and Urbanetto, Janete de Souza and Bosi de Souza Magnago, TÃ¢nia Solange |
| 1277 | Wrong outcome | Patient safety culture, missed Nursing care and its reasons in Obstetrics | 2021 | Silva, S. C. D. and Morais, B. X. and Munhoz, O. L. and Ongaro, J. D. and Urbanetto, J. S. and Magnago, T. S. B. S. |
| 1278 | Wrong outcome | Patient safety during transfers from critical care: developing and assessing a checklist | 2024 | Pearson, L. and Finney, A. |
| 1279 | Wrong outcome | Patient SafetyNet for the Evaluation of Postoperative Respiratory Status by Nurses: A Presurvey and Postsurvey Study | 2021 | Ishikawa, Masashi and Sakamoto, Atsuhiro |
| 1280 | Wrong outcome | Patient satisfaction survey in a teaching hospital in saudi arabia: preliminary results | 1995 | Al Umran, K. and Albar, A. and Ai-Awdah, S. and Ai-Jaber, S. and Wosornu, L. |
| 1281 | Wrong outcome | [Patient-controlled analgesia with piritramide for postoperative pain relief in general surgery: a prospective observational study.] | 1993 | Ure, B. M. and Ullmann, K. and Neugebauer, E. and Bende, J. and Troidl, H. |
| 1282 | Wrong outcome | Patients' and Providers' Perspectives on and Needs of Telemonitoring to Support Clinical Management and Self-care of People at High Risk for Preeclampsia: Qualitative Study | 2022 | Aquino, M. and Griffith, J. and Vattaparambil, T. and Munce, S. and Hladunewich, M. and Seto, E. |
| 1283 | Wrong outcome | Patients' experiences of postoperative intermediate care and standard surgical ward care after emergency abdominal surgery: a qualitative sub-study of the Incare trial | 2015 | Thomsen, T. and Vester-Andersen, M. and Nielsen, M. V. and Waldau, T. and MÃ¸ller, A. M. and Rosenberg, J. and MÃ¸ller, M. H. and Nystrup, K. B. and Esbensen, B. A. |
| 1284 | Wrong outcome | Patients' outcomes: intrahospital transportation and monitoring of critically ill patients by a specially trained ICU nursing staff | 1998 | Stearley, H. E. |
| 1285 | Wrong outcome | Patients' vital signs and the length of time between the monitoring of vital signs during times of emergency department crowding | 2011 | Johnson, Kimberly D. |
| 1286 | Wrong outcome | Patients'satisfaction with the rheumatology day care unit | 2011 | Barbosa, L. and Ramiro, S. and Roque, R. and GonÃ§alves, P. and da Silva, J. C. and Santos, M. J. |
| 1287 | Wrong population | A pediatric death audit in a large referral hospital in Malawi | 2018 | Fitzgerald, E. and Mlotha-Mitole, R. and Ciccone, E. J. and Tilly, A. E. and Montijo, J. M. and Lang, H. J. and Eckerle, M. |
| 1288 | Wrong population | Pediatric Early Warning Score Systems, Nurses Perspective â€“ A Focus Group Study | 2018 | Jensen, Claus Sixtus and Nielsen, Pia Bonde and Olesen, Hanne Vebert and Kirkegaard, Hans and Aagaard, Hanne |
| 1289 | Wrong outcome | Pediatric health care providers' knowledge of pulse oximetry | 2004 | Popovich, D. M. and Richiuso, N. and Danek, G. |
| 1290 | Wrong population | A pediatric hypertension center: Two year review of an outpatient diagnostic module | 1994 | Kania, P. and Gauthier, B. and Frank, R. and Florestan, M. A. and Trachtman, H. |
| 1291 | Wrong outcome | Pediatric Rapid Response Systems: Identification and Treatment of Deteriorating Children | 2015 | Levin, A. B. and Brady, P. and Duncan, H. P. and Davis, A. B. |
| 1292 | Wrong population | Pediatric vital signs monitoring in hospital wards: Recognition systems and factors influencing nurses' attitudes and practices | 2023 | Thekkan, K. R. and Genna, C. and Ferro, F. and Cecchetti, C. and Dall'Oglio, I. and Tiozzo, E. and Raponi, M. and Gawronski, O. |
| 1293 | Wrong outcome | Penetrating abdominal trauma | 1989 | Henneman, P. L. |
| 1294 | Wrong outcome | Perceived work ability and turnover intentions: A prospective study among Belgian healthcare workers | 2012 | Derycke, H. and Clays, E. and Vlerick, P. and D'Hoore, W. and Hasselhorn, H. M. and Braeckman, L. |
| 1295 | Wrong outcome | Perception of caring among nurses working in a tertiary hospital in Riyadh, Saudi Arabia | 2020 | Albougami, A. S. |
| 1296 | Wrong outcome | Performance and Acceptability of Health Literacy Measurements in Hospitalized Adults with Heart Failure...28th Annual Scientific Session, June 2-6, 2017, Baltimore, Maryland | 2016 | Mock, Margaret and Sethares, Kristen |
| 1297 | Wrong outcome | Performance of handheld electrocardiogram devices to detect atrial fibrillation in a cardiology and geriatric ward setting | 2017 | Desteghe, L. and Raymaekers, Z. and Lutin, M. and Vijgen, J. and Dilling-Boer, D. and Koopman, P. and Schurmans, J. and Vanduynhoven, P. and Dendale, P. and Heidbuchel, H. |
| 1298 | Wrong outcome | Performance of infrared ear and forehead thermometers: a comparative study in 205 febrile and afebrile children | 2013 | Hamilton, Patricia A. and Marcos, Lorenzo S. and Secic, Michelle |
| 1299 | Wrong outcome | Performance of non-contact infrared thermometer for detecting febrile children in hospital and ambulatory settings | 2011 | Chiappini, E. and Sollai, S. and Longhi, R. and Morandini, L. and Laghi, A. and Osio, C. E. and Persiani, M. and Picchi, R. and Lonati, S. and Bonsignori, F. and Mannelli, F. and Galli, L. and De Martino, M. |
| 1300 | Wrong outcome | Perioperative hypothermia prevention: development of simple principles and practice recommendations using a multidisciplinary consensus-based approach | 2023 | Munday, J. and Duff, J. and Wood, F. M. and Sturgess, D. and Ralph, N. and Ramis, M. A. |
| 1301 | Wrong outcome | Perioperative Nursing Care of a Patient Who Underwent Arthroscopic Shoulder Labral Surgery | 2020 | Shei-Tsung, Chen and Chun-Chen, Lin and Yun-Shan, Tseng |
| 1302 | Wrong outcome | Perioperative Peripheral Nerve Block Safety Education: A Quality Improvement Project...American Society of PeriAnesthesia Nurses 40th National Conference, 25-29 April, 2021 | 2021 | Zelaya, Pauline |
| 1303 | Wrong outcome | Peripheral perfusion and oxygenation in areas of risk of skin integrity impairment exposed to pressure patterns. A phase I trial (POTER Study) | 2018 | GarcÃ­a-Mayor, S. and Morilla-Herrera, J. C. and LupiÃ¡Ã±ez-PÃ©rez, I. and Kaknani Uttumchandani, S. and LeÃ³n Campos, Ã and Aranda-Gallardo, M. and Moya-SuÃ¡rez, A. B. and Morales-Asencio, J. M. |
| 1304 | Wrong outcome | Periprocedural management of 172 gastrointestinal endoscopies in patients with left ventricular assist devices | 2015 | Barbara, D. W. and Olsen, D. A. and Pulido, J. N. and Boilson, B. A. and Bruining, D. H. and Stulak, J. M. and Mauermann, W. J. |
| 1305 | Wrong outcome | Personal space intrusion and PTSD | 1996 | Brown, P. and Yantis, J. |
| 1306 | Wrong outcome | A Personalized and Interactive Web-Based Health Care Innovation to Advance the Quality of Life and Care of Patients With Heart Failure (ACQUIRE-HF): A Mixed Methods Feasibility Study | 2017 | Pedersen, S. S. and Schmidt, T. and Skovbakke, S. J. and Wiil, U. K. and Egstrup, K. and Smolderen, K. G. and Spertus, J. A. |
| 1307 | Wrong outcome | Pharmacist Hypertension Management Quality Review at an Ambulatory Care Clinic | 2022 | Chonko, K. and Axtell, S. and Mayzel, B. |
| 1308 | Wrong outcome | Pharmacist intervention program to enhance hypertension control: a randomised controlled trial | 2011 | Morgado, M. and Rolo, S. and Castelo-Branco, M. and Morgado, Manuel and Rolo, Sandra and Castelo-Branco, Miguel |
| 1309 | Wrong population | Pharmacokinetics of dexmedetomidine in postsurgical pediatric intensive care unit patients: preliminary study | 2007 | DÃ­az, S. M. and Rodarte, A. and Foley, J. and Capparelli, E. V. |
| 1310 | Wrong outcome | Pharmacological treatment of the arterial hypertension in coronary intensive care unit | 2010 | da Cunha, Gilmara Holanda and Lopes, Marcos VenÃcios de Oliveira and Leite, Ismenia OsÃ³rio |
| 1311 | Wrong outcome | Phone-Based Intervention under Nurse Guidance after Stroke (PINGS II) Study: Protocol for a Phase III Randomized Clinical Trial | 2021 | Sarfo, F. S. and Akpalu, A. and Bockarie, A. and Appiah, L. and Nguah, S. B. and Ayisi-Boateng, N. K. and Adamu, S. and Neizer, C. and Arthur, A. and Nyamekye, R. and Agyenim-Boateng, K. and Tagge, R. and Adusei-Mensah, N. and Ampofo, M. and Laryea, R. and Singh, A. and Amuasi, J. H. and Ovbiagele, B. |
| 1312 | Wrong outcome | Physical deterioration in an acute mental health unit: A quantitative retrospective analysis of medical emergencies | 2018 | Porter, Joanne E. and Cant, Robyn and Missen, Karen and Raymond, Anita and Churchill, Anne |
| 1313 | Wrong outcome | Physical hazard safety awareness among healthcare workers in Tanta university hospitals, Egypt | 2018 | El-Sallamy, R. M. and Kabbash, I. A. and El-Fatah, S. A. and El-Feky, A. |
| 1314 | Wrong outcome | Physical Healthcare of People with Serious Mental Illness: A Cross-Sectional Study of Nurses' Involvement, Views, and Current Practices | 2019 | YalÃ§Ä±n, Suna Uysal and Bilgin, HÃ¼lya and Ã–zaslan, Zeynep |
| 1315 | Wrong outcome | The physiologic cipher at altitude: Telemedicine and real-time monitoring of climbers on Mount Everest | 2000 | Satava, R. and Angood, P. B. and Harnett, B. and Macedonia, C. and Merrell, R. |
| 1316 | Wrong outcome | The physiologic effect of intrahospital transport on the adult critically ill patient | 2002 | Marshall, P. M. |
| 1317 | Wrong outcome | Physiological and behavioural response patterns at work among hospital nurses | 2011 | Chen, J. and Davis, L. S. and Davis, K. G. and Pan, W. and Daraiseh, N. M. |
| 1318 | Wrong outcome | Physiological and behavioural response patterns at work among hospital nurses | 2011 | Jie, Chen and Davis, L. Sue and Davis, Kermit G. and Wei, P. A. N. and Daraiseh, Nancy M. |
| 1319 | Wrong population | Physiological correlates of painful stimulation in preterm infants | 1993 | Gonsalves, S. and Mercer, J. |
| 1320 | Wrong outcome | The physiological effect on rescuers of doing 2min of uninterrupted chest compressions | 2007 | Riera, S. Q. and GonzÃ¡lez, B. S. and Alvarez, J. T. and FernÃ¡ndez Mdel, M. and Saura, J. M. |
| 1321 | Wrong outcome | Physiological observations of patients admitted from A&E | 2002 | Alcock, K. and Clancy, M. and Crouch, R. |
| 1322 | Wrong outcome | Physiological trajectory of patients pre and post ICU discharge | 2014 | Johnson, A. E. and Burgess, J. and Pimentel, M. A. and Clifton, D. A. and Young, J. D. and Watkinson, P. J. and Tarassenko, L. |
| 1323 | Wrong outcome | PICA infarction, Wallenberg Syndrome; Presentation of a case | 2010 | SÃ¡nchez-Camacho-Maroto, P. and Borrallo-LÃ³pez, J. A. and SanjuÃ¡n-MenÃ©ndez, E. and Cruz-DÃ­az, V. and Cepeda-Bautista, M. and Rodrigo-Gil, J. |
| 1324 | Wrong outcome | Pilot Mobile Phone Intervention in Promoting Type 2 Diabetes Management in an Urban Area in Ghana: A Randomized Controlled Trial | 2020 | Asante, Ernest and Bam, Victoria and Diji, Abigail Kusi-Amponsah and Lomotey, Alberta Yemotsoo and Owusu Boateng, Agnes and Sarfo-Kantanka, Osei and Oparebea Ansah, Eunice and Adjei, Dennis |
| 1325 | Wrong outcome | A pilot observational study of gait changes over time before and after an unplanned hospital visit in long-term care residents with dementia | 2023 | Nabavi, H. and Mehdizadeh, S. and Shum, L. C. and Flint, A. J. and Mansfield, A. and Taati, B. and Iaboni, A. |
| 1326 | Wrong outcome | Pilot Project: Heart Chargersâ€”A Successful Model for a Home-Based Physical Activity Program Utilizing Telemedicine for Fontan Patients | 2023 | Fernie, J. C. and Wylie, L. and SchÃ¤fer, M. and Carnegie, K. and Miyamoto, S. D. and Jacobsen, R. M. |
| 1327 | Wrong outcome | A Pilot Quality Improvement Project to Reduce Intraoperative MRI Hypothermia in Neurosurgical Patients | 2022 | Wong, B. J. and Rama, A. and Caruso, T. J. and Lee, C. K. and Wang, E. and Chen, M. |
| 1328 | Wrong outcome | A pilot randomised controlled trial of a Telehealth intervention in patients with chronic obstructive pulmonary disease: challenges of clinician-led data collection | 2014 | Bentley, C. L. and Mountain, G. A. and Thompson, J. and Fitzsimmons, D. A. and Lowrie, K. and Parker, S. G. and Hawley, M. S. |
| 1329 | Wrong outcome | Pilot research: Construction of emergency rescue database | 2018 | Yuzhuo, Z. and Junmei, W. and Fei, P. and Peiyao, L. and Lijing, J. and Kaiyuan, L. and Cong, F. and Tongbo, L. and Zhengbo, Z. and Desen, C. and Tanshi, L. |
| 1330 | Wrong outcome | [Pilot research: construction of emergency rescue database] | 2018 | Zhao, Y. and Wang, J. and Pan, F. and Li, P. and Jia, L. and Li, K. and Feng, C. and Liu, T. and Zhang, Z. and Cao, D. and Li, T. |
| 1331 | Wrong population | Pilot study of home phototherapy for neonatal jaundice monitored in maternity ward during the enforced Italy-wide COVID-19 national lockdown | 2022 | Zanardo, V. and Guerrini, P. and Sandri, A. and Ramon, C. M. and Severino, L. and Garani, G. and Mesirca, P. and Straface, G. |
| 1332 | Wrong outcome | A pilot study of nursing activities on the Chest Medicine Ward at KMCH | 1993 | Pan, S. M. and Chin, C. C. and Hwang, S. C. and Ko, C. C. |
| 1333 | Wrong outcome | A pilot study testing the feasibility of skin temperature monitoring to reduce recurrent foot ulcers in patients with diabetes--a randomized controlled trial | 2015 | Skafjeld, A. and Iversen, M. M. and Holme, I. and Ribu, L. and Hvaal, K. and Kilhovd, B. K. |
| 1334 | Wrong Study Design | A pilot study to investigate real-time digital alerting from wearable sensors in surgical patients | 2022 | Joshi, M. and Ashrafian, H. and Arora, S. and Sharabiani, M. and McAndrew, K. and Khan, S. N. and Cooke, G. S. and Darzi, A. |
| 1335 | Wrong outcome | The pilot, proof of concept REMOTE-COVID trial: remote monitoring use in suspected cases of COVID-19 (SARS-CoV 2) | 2021 | Iqbal, F. M. and Joshi, M. and Davies, G. and Khan, S. and Ashrafian, H. and Darzi, A. |
| 1336 | Wrong outcome | Planning a change project in mental health nursing | 2015 | Thorpe, Rebecca |
| 1337 | Wrong outcome | Poor prognosis for existing monitors in the intensive care unit | 1997 | Tsien, C. L. and Fackler, J. C. |
| 1338 | Wrong outcome | Post Discharge after Surgery Virtual Care with Remote Automated Monitoring Technology (PVC-RAM): protocol for a randomized controlled trial | 2021 | McGillion, M. H. and Parlow, J. and Borges, F. K. and Marcucci, M. and Jacka, M. and Adili, A. and Lalu, M. M. and Yang, H. and Patel, A. and O'Leary, S. and Tandon, V. and Hamilton, G. M. and Mrkobrada, M. and Ouellette, C. and Bird, M. and Ofori, S. and Conen, D. and Roshanov, P. S. and Harvey, V. and Guyatt, G. H. and Le Manach, Y. and Bangdiwala, S. I. and Arellano, R. and Scott, T. and Lounsbury, J. and Taylor, D. A. and Nenshi, R. and Forster, A. J. and Nagappa, M. and Lamy, A. and Peter, E. and Levesque, K. and Marosi, K. and Chaudhry, S. and Haider, S. and Deuchar, L. and LeBlanc, B. and McCartney, C. J. L. and Schemitsch, E. H. and Vincent, J. and Pettit, S. M. and Paul, J. and DuMerton, D. and Paulin, A. D. and Simunovic, M. and Williams, D. C. and Halman, S. and Schlachta, C. M. and Shelley, J. and Harlock, J. and Meyer, R. M. and Graham, M. and Shanthanna, H. and Parry, N. and Pichora, D. R. and Yousef, H. and Moloo, H. and Sehmbi, H. and Waggott, M. and Belley-Cote, E. P. and Whitlock, R. and Devereaux, P. J. |
| 1339 | Wrong outcome | Post operative capnostream monitoring in patients with obstructive sleep apnoea symptoms - Case series | 2016 | Rao Kadam, V. and Danesh, M. |
| 1340 | Wrong outcome | Post-discharge after surgery Virtual Care with Remote Automated Monitoring-1 (PVC-RAM-1) technology versus standard care: randomised controlled trial | 2021 | McGillion, M. H. and Parlow, J. and Borges, F. K. and Marcucci, M. and Jacka, M. and Adili, A. and Lalu, M. M. and Ouellette, C. and Bird, M. and Ofori, S. and Roshanov, P. S. and Patel, A. and Yang, H. and O'Leary, S. and Tandon, V. and Hamilton, G. M. and Mrkobrada, M. and Conen, D. and Harvey, V. and Lounsbury, J. and Mian, R. and Bangdiwala, S. I. and Arellano, R. and Scott, T. and Guyatt, G. H. and Gao, P. and Graham, M. and Nenshi, R. and Forster, A. J. and Nagappa, M. and Levesque, K. and Marosi, K. and Chaudhry, S. and Haider, S. and Deuchar, L. and LeBlanc, B. and McCartney, C. J. L. and Schemitsch, E. H. and Vincent, J. and Pettit, S. M. and DuMerton, D. and Paulin, A. D. and Simunovic, M. and Williams, D. C. and Halman, S. and Harlock, J. and Meyer, R. M. and Taylor, D. A. and Shanthanna, H. and Schlachta, C. M. and Parry, N. and Pichora, D. R. and Yousuf, H. and Peter, E. and Lamy, A. and Petch, J. and Moloo, H. and Sehmbi, H. and Waggott, M. and Shelley, J. and Belley-Cote, E. P. and Devereaux, P. J. |
| 1341 | Wrong outcome | Post-hospitalization remote monitoring for patients with heart failure or chronic obstructive pulmonary disease in an accountable care organization | 2024 | Harris, S. and Paynter, K. and Guinn, M. and Fox, J. and Moore, N. and Maddox, T. M. and Lyons, P. G. |
| 1342 | Wrong outcome | Postâ€craniotomy fever and its associated factors in patients with traumatic brain injury | 2022 | Huang, Juiâ€Hsia and Wang, Tsaeâ€Jyy and Wu, Shuâ€Fang and Liu, Chiehâ€Yu and Fan, Junâ€Yu |
| 1343 | Wrong population | Postanesthesia care by remote monitoring of vital signs in surgical wards | 2018 | Boer, C. and Touw, H. R. and Loer, S. A. |
| 1344 | Wrong outcome | Postimplementation Evaluation of a Machine Learning--Based Deterioration Risk Alert to Enhance Sepsis Outcome Improvements | 2020 | Linnen, Daniel T. and Xiao, Hu and Stephens, Caroline E. |
| 1345 | Wrong outcome | Postoperative care of the adult cardiac surgical patient | 2009 | Parnell, A. D. and Massey, N. J. |
| 1346 | Wrong outcome | Postoperative care of the adult cardiac surgical patient | 2012 | Rosser, J. H. and Parnell, A. D. and Massey, N. J. |
| 1347 | Wrong outcome | Postoperative care of the adult cardiac surgical patient | 2015 | Press, C. P. and Rosser, J. H. and Parnell, A. D. |
| 1348 | Wrong outcome | [Postoperative intensive care of biliary atresia patients treated with living donor liver transplantation] | 2011 | Deng, Y. H. and Guo, C. B. and Zhang, M. M. and Li, Y. C. |
| 1349 | Wrong outcome | Postoperative lower extremity bypass surveillance: beyond ankle arm blood pressures | 1995 | Foldes, M. S. |
| 1350 | Wrong population | Postoperative Recovery in the Youngest: Beyond Technology | 2024 | SjÃ¶berg, C. and Ringdal, M. and JildenstÃ¥l, P. |
| 1351 | Wrong outcome | Postoperative Remote Automated Monitoring and Virtual Hospital-to-Home Care System Following Cardiac and Major Vascular Surgery: User Testing Study | 2020 | McGillion, M. and Ouellette, C. and Good, A. and Bird, M. and Henry, S. and Clyne, W. and Turner, A. and Ritvo, P. and Ritvo, S. and Dvirnik, N. and Lamy, A. and Whitlock, R. and Lawton, C. and Walsh, J. and Paterson, K. and Duquette, J. and Sanchez Medeiros, K. and Elias, F. and Scott, T. and Mills, J. and Harrington, D. and Field, M. and Harsha, P. and Yang, S. and Peter, E. and Bhavnani, S. and Devereaux, P. J. |
| 1352 | Wrong outcome | Postoperative respiratory state assessment using the Integrated Pulmonary Index (IPI) and resultant nurse interventions in the post-anesthesia care unit: a randomized controlled trial | 2021 | Broens, S. J. L. and Prins, S. A. and de Kleer, D. and Niesters, M. and Dahan, A. and van Velzen, M. |
| 1353 | Wrong outcome | Postoperative temperature and infection in patients undergoing general surgery | 1989 | Payman, B. C. and Dampier, S. E. and Hawthorn, P. J. |
| 1354 | Wrong outcome | Postoperative ward monitoring - Why and what now? | 2019 | Khanna, Ashish K. and Ahuja, Sanchit and Weller, Robert S. and Harwood, Timothy N. |
| 1355 | Wrong outcome | [Postoperative warming therapy in the recovery room. A comparison of radiative and convective warmers] | 1994 | Weyland, W. and Fritz, U. and Fabian, S. and Jaeger, H. and Crozier, T. and Kietzmann, D. and Braun, U. |
| 1356 | Wrong population | Postpartum Health Services Requested by Mothers with Newborns Receiving Intensive Care | 2016 | Verbiest, S. and McClain, E. and Stuebe, A. and Menard, M. K. |
| 1357 | Wrong population | Postpartum Outcomes With Systematic Treatment and Management of Postpartum Hypertension | 2021 | Suresh, S. C. and Duncan, C. and Kaur, H. and Mueller, A. and Tung, A. and Perdigao, J. L. and Khosla, K. and Dhir, R. and Stewart, K. and Wallace, K. and Ahn, R. and Rana, S. |
| 1358 | Wrong population | A Postpartum Remote Hypertension Monitoring Protocol Implemented at the Hospital Level | 2019 | Hauspurg, A. and Lemon, L. S. and Quinn, B. A. and Binstock, A. and Larkin, J. and Beigi, R. H. and Watson, A. R. and Simhan, H. N. |
| 1359 | Wrong population | A Postpartum Remote Hypertension Monitoring Protocol Implemented at the Hospital Level | 2019 | Hauspurg, Alisse M. D. and Lemon, Lara S. PharmD PhD and Quinn, Beth A. R. N. and Binstock, Anna M. D. and Larkin, Jacob M. D. and Beigi, Richard H. M. D. and Watson, Andrew R. M. D. and Simhan, Hyagriv N. M. D. and Hauspurg, Alisse and Lemon, Lara S. and Quinn, Beth A. and Binstock, Anna and Larkin, Jacob and Beigi, Richard H. and Watson, Andrew R. and Simhan, Hyagriv N. |
| 1360 | Wrong outcome | Potential for remote vital sign monitoring to improve hospital patient sleep: A feasibility study | 2023 | Beaman, H. and Douglas, V. C. and Patel, K. and John Boscardin, W. and Youn, J. and LaHue, S. C. |
| 1361 | Wrong outcome | Power to the patients | 2012 | Pearce, L. |
| 1362 | Wrong population | PP104. Pregnancy-induced hypertension is a strong risk factor for hypertension just 5 years after delivery: A double cohort study at the National Center for Child Health and Development and Showa University Hospital, Tokyo | 2012 | Mito, A. and Arata, N. and Jwa, S. C. and Sakamoto, N. and Qiu, D. and Murashima, A. and Ichihara, A. and Matsuoka, R. and Sekizawa, A. and Ohya, Y. and Kitagawa, M. |
| 1363 | Wrong outcome | A practical comparison of temporal artery thermometry and axillary thermometry in neonates under different environments | 2016 | Sim, Ming Ann and Leow, Syen Yee and Hao, Ying and Yeo, Cheo Lian |
| 1364 | Wrong outcome | Practice corner. Practice tips from Columbia/HNA Cancer Centers... Elimination of frequent vital sign monitoring in patients receiving paclitaxel | 1998 | Thomas, M. |
| 1365 | Wrong outcome | Practices and Barriers towards Physical Assessment among Nurses Working in Intensive Care Units: Multicenter Cross-Sectional Study | 2021 | Liyew, B. and Tilahun, A. D. and Kassew, T. |
| 1366 | Wrong outcome | Pre-warming Surgical Patients...American Society of PeriAnesthsia nurses (ASPAN) National Conference, April 14-18, 2024, Orlando, Florida | 2024 | Durbin, Primary Investigator Ashley and Orr, Co-Investigator Casey |
| 1367 | Wrong outcome | Predicting early deterioration of admitted patients at the Intermediate Care Unit | 2018 | Plate, J. D. J. and Hietbrink, F. and Leenen, L. P. H. and Peelen, L. M. |
| 1368 | Wrong outcome | Predicting Intensive Care Unit Readmission with Machine Learning Using Electronic Health Record Data | 2018 | Rojas, Juan C. and Carey, Kyle A. and Edelson, Dana P. and Venable, Laura R. and Howell, Michael D. and Churpek, Matthew M. |
| 1369 | Wrong outcome | Predictive factors for bleeding-related re-exploration after cardiac surgery: A prospective cohort study | 2016 | Lopes, C. T. and Brunori, E. H. and Santos, V. B. and Moorhead, S. A. and Lopes Jde, L. and de Barros, A. L. |
| 1370 | Wrong outcome | Predictive factors of intensive care length of stay in liver transplant recipients | 2014 | Rowe, Lynn Ann |
| 1371 | Wrong population | Predictive Monitoring of Critical Cardiorespiratory Alarms in Neonates under Intensive Care | 2019 | Joshi, R. and Peng, Z. and Long, X. and Feijs, L. and Andriessen, P. and Van Pul, C. |
| 1372 | Wrong outcome | Predictive nursing helps improve treatment efficacy, treatment compliance, and quality of life in unstable angina pectoris patients | 2021 | Zhu, Y. and Duan, X. |
| 1373 | Wrong outcome | Predictive power of a single body temperature at different cutoff values for neonates in the nursery transferring to special care nursery | 2018 | Lee, En-Pei and Yu, Meng-Kung and Lee, Shu-Chun and Gao, Feng-Xia and Wu, Han-Ping |
| 1374 | Wrong outcome | The predictive value of the modified early warning score for admission to the intensive care unit in patients with a hematologic malignancy - A multicenter observational study | 2023 | van Mourik, N. and Oomen, J. J. and van Vught, L. A. and Biemond, B. J. and van den Bergh, W. M. and Blijlevens, N. M. A. and Vlaar, A. P. J. and MÃ¼ller, M. C. A. |
| 1375 | Wrong outcome | Prehospital Hemorrhage Assessment Criteria: A Concise Review | 2021 | Di Carlo, Sara and Cavallaro, Giuseppe and Palomeque, Kenia and Cardi, Maurizio and Sica, Giuseppe and Rossi, Piero and Sibio, Simone |
| 1376 | Wrong outcome | Preparation of simulation programs regarding excess-dose drug administration and acute-phase condition changes and its evaluation by students | 2011 | Tokunaga, J. and Takamura, N. and Ogata, K. and Setoguchi, N. and Matsuoka, T. and Sato, K. |
| 1377 | Wrong outcome | Preparedness of primary & secondary care health facilities for the management of non-communicable diseases in tribal population across 12 districts in India | 2022 | Kaur, P. and Borah, P. K. and Gaigaware, P. and Mohapatra, P. K. and NK, R. Das and Uike, P. V. and Tobgay, K. J. and Tushi, A. and Zorinsangi and Mazumdar, G. and Marak, B. and Pizi, D. and Chakma, T. and Sugunan, A. P. and Vijayachari, P. and Bhardwaj, R. R. and Arambam, P. C. and Kutum, T. and Sharma, A. and Pal, P. and Shanmugapriya, P. C. and Manivel, P. and Kaliyamoorthy, N. and Chakma, J. and Mathur, P. and Dhaliwal, R. S. and Mahanta, J. and Mehendale, S. M. |
| 1378 | Wrong outcome | Preparing for total power failure in the operating room | 2019 | Vetter, A. G. and Harman, R. J. and Stamper, M. J. and Titch, J. F. and Vacchiano, C. A. |
| 1379 | Wrong outcome | Preparing infection detection technology for hospital at home after lower limb external fixation | 2022 | Annadatha, S. and Hua, Q. and Fridberg, M. and LindstrÃ¸m Jensen, T. and Liu, J. and Kold, S. and Rahbek, O. and Shen, M. |
| 1380 | Wrong outcome | Presenting an efficient approach based on novel mapping for mortality prediction in intensive care unit cardiovascular patients | 2018 | Karimi Moridani, M. and Haghighi Bardineh, Y. |
| 1381 | Wrong outcome | Pressure monitoring in continuous renal replacement therapy | 2010 | Guirao Moya, A. and Esteban SÃ¡nchez, M. E. and FernÃ¡ndez Gaute, N. and Murga GonzÃ¡lez, A. and Vergara Diez, L. and MartÃ­nez GarcÃ­a, M. P. and DomÃ­nguez DomÃ­nguez, J. E. and Frade Mera, M. J. and Cruz Ramos, A. M. and Molano Ãlvarez, E. |
| 1382 | Wrong outcome | Preterm infant behavioral and heart rate responses to antenatal phenobarbital | 1999 | McCain, G. C. and Donovan, E. F. and Gartside, P. |
| 1383 | Wrong outcome | Preterm infants: behavioral responses to handling by the nursing team | 2012 | AldiÃ¢nia Carlos, Balbino and Maria Vera LÃºcia Moreira LeitÃ£o, Cardoso and Regina CÃ©lia Carvalho da, Silva and KÃ©sia Marques, Moraes |
| 1384 | Wrong outcome | Prevalence and characteristics of a group dementia patients in the final phase of dementia | 2005 | De Jong, J. W. D. and Ekkerink, J. L. P. and Touma, I. and Koopmans, R. T. C. M. |
| 1385 | Wrong outcome | The prevalence and course of neuropsychiatric symptoms in stroke patients impact functional recovery during in-hospital rehabilitation | 2022 | Suzuki, Akihito and Mutai, Hitoshi and Furukawa, Tomomi and Wakabayashi, Ayumi and Hanihara, Tokiji |
| 1386 | Wrong outcome | Prevalence and factors affecting home blood pressure documentation in routine clinical care: a retrospective study | 2010 | Kramer, M. H. and Breydo, E. and Shubina, M. and Babcock, K. and Einbinder, J. S. and Turchin, A. and Kramer, Michael H. and Breydo, Eugene and Shubina, Maria and Babcock, Kelly and Einbinder, Jonathan S. and Turchin, Alexander |
| 1387 | Wrong outcome | Prevalence of Anaesthesia Complications in Orthopaedic Surgeries and its Related Factors | 2023 | Sahoo, D. and Mishra, S. K. and Suresh Kumar Gupta, T. and Parida, S. K. and Das, A. and Panda, S. K. and Dhar, R. N. |
| 1388 | Wrong outcome | Prevalence of end-digit preference in recorded blood pressure by nurses: a comparison of measurements taken by mercury and electronic blood pressure-measuring devices | 2013 | Ayodele, O. E. and Akinyemi, S. O. and Akinboro, A. O. and Popoola, A. A. and Alao, C. A. |
| 1389 | Wrong outcome | Prevalence of High Blood Pressure and its Relationship with Body Weight Factors among Inpatients with Schizophrenia in Taiwan | 2012 | Lan, Y. L. and Chen, T. L. |
| 1390 | Wrong outcome | Preventing cardiopulmonary arrest via enhanced vital signs monitoring | 1995 | Hill, M. G. and Fieselmann, J. F. and Nobiling, H. E. and O'Neill, P. S. and Barry-Walker, J. and Dwyer, J. and Kobler, L. |
| 1391 | Wrong outcome | Preventing Perioperative Hypothermia...American Society of PeriAnesthesia Nurses, 42nd National Conference, April 28 - May 1, 2023, Denver, Colorado | 2023 | Gibbs, Cary |
| 1392 | Wrong outcome | Preventing unrecognized deterioration & improving outcomes of critically ill patients using the National Early Warning Score 2 in a high dependency unit in Bangladesh: A quality improvement project | 2023 | Anam, A. M. and Shareef, A. and Shumy, F. and Gerardus King, M. R. |
| 1393 | Wrong outcome | Prevention after stroke: A quality assurance study | 2021 | Hornnes, N. |
| 1394 | Wrong outcome | Prevention of deterioration in acutely ill patients in hospital | 2010 | Steen, C. |
| 1395 | Wrong outcome | Prevention of hospitalizations for heart failure with an interactive home monitoring program | 1998 | Shah, N. B. and Der, E. and Ruggerio, C. and Heidenreich, P. A. and Massie, B. M. |
| 1396 | Wrong outcome | Prevention of hypothermia in trauma victims - the HYPOTRAUM 2 study | 2021 | Lapostolle, F. and Garrigue, B. and Richard, O. and Weisslinger, L. and Chollet, C. and Lagadec, S. and Soulat, L. and Ricard-Hibon, A. and Hilaire-Schneider, C. and Debaty, G. and Mazur, V. and Vicaut, E. |
| 1397 | Wrong outcome | Preventive strategies for feeding intolerance among patients with severe traumatic brain injury: A cross-sectional survey | 2022 | Fang, Y. and Ma, Y. and He, H. and Chen, T. and Fu, J. and Zhu, J. |
| 1398 | Wrong population | Primary care issues for the healthy premature infant | 2006 | Kelly, M. M. |
| 1399 | Wrong outcome | Primary Exploration of Efficacy of Community-Family Management Mode under Internet-Based Mobile Terminal Monitoring in Elderly Patients with Stable Coronary Heart Disease | 2022 | Li, X. and Zheng, W. and Li, J. and Gao, Y. and Lin, Q. and Yang, J. and Huang, S. and Wang, D. and Wang, B. |
| 1400 | Wrong outcome | Prioritising Responses Of Nurses To deteriorating patient Observations (PRONTO): a pragmatic cluster randomised controlled trial evaluating the effectiveness of a facilitation intervention on recognition and response to clinical deterioration | 2022 | Bucknall, Tracey K. and Considine, Julie and Harvey, Gillian and Graham, Ian D. and Malone, Jo Rycroft and Mitchell, Imogen and Saultry, Bridey and Watts, Jennifer J. and Mohebbi, Mohammadreza and Mudiyanselage, Shalika Bohingamu and Lotfaliany, Mojtaba and Hutchinson, Alison |
| 1401 | Wrong outcome | Pro-Con Debate: Universal Versus Selective Continuous Monitoring of Postoperative Patients | 2024 | Blike, G. T. and McGrath, S. P. and Ochs Kinney, M. A. and Gali, B. |
| 1402 | Wrong outcome | A pro/con review comparing the use of mono- and multiplace hyperbaric chambers for critical care | 2015 | Lind, F. |
| 1403 | Wrong outcome | Proactive management of heart failure by digital health: is monitoring of invasive pulmonary artery pressure the Holy Grail? | 2020 | Mortara, A. and Margonato, D. |
| 1404 | Foreign Language | Problemi aperti nel passaggio del paziente tra la terapia intensiva e la terapia sub-intensiva: gestione del setting, vie aeree, respiro e circolo...Open issues related to the transfer of the patient from the intensive care unit to the high dependency unit: Management of clinical setting, airways... | 2017 | Cholewa, Agnieszka Katarzyna and Lucchini, Alberto and Caruso, Christian and Bambi, Stefano |
| 1405 | Wrong outcome | Problems identified in gaining non-expert consensus for a hypothetical wound assessment form | 2003 | Maylor, M. E. |
| 1406 | Wrong outcome | Procedural sedation of adult patients in the emergency department: a best practice implementation project | 2024 | Pickens, J. and Garbo, C. |
| 1407 | Wrong outcome | Proceedings of the 3rd IPLeiria's International Health Congress : Leiria, Portugal. 6-7 May 2016 | 2016 | TomÃ¡s, C. C. and Oliveira, E. and Sousa, D. and Uba-Chupel, M. and Furtado, G. and Rocha, C. and Teixeira, A. and Ferreira, P. and Alves, C. and Gisin, S. and Catarino, E. and Carvalho, N. and Coucelo, T. and Bonfim, L. and Silva, C. and Franco, D. and GonzÃ¡lez, J. A. and Jardim, H. G. and Silva, R. and Baixinho, C. L. and Presado MÂª, H. and Marques MÂª, F. and Cardoso, M. E. and Cunha, M. and Mendes, J. and Xavier, A. and Galhardo, A. and Couto, M. and Frade, J. G. and Nunes, C. and Mesquita, J. R. and Nascimento, M. S. and GonÃ§alves, G. and Castro, C. and MÃ¡rtires, A. and Monteiro MÂª, J. and Rainho, C. and Caballero, F. P. and Monago, F. M. and Guerrero, J. T. and Monago, R. M. and Trigo, A. P. and Gutierrez, M. L. and MilanÃ©s, G. M. and Reina, M. G. and Villanueva, A. G. and PiÃ±ero, A. S. and Aliseda, I. R. and Ramirez, F. B. and Ribeiro, A. and Quelhas, A. and Manso, C. and Caballero, F. P. and Guerrero, J. T. and Monago, F. M. and Santos, R. B. and Jimenez, N. R. and NuÃ±ez, C. G. and Gomez, I. R. and Fernandez MÂª, J. L. and Marquez, L. A. and Moreno, A. L. and Huertas MÂª, J. T. and Ramirez, F. B. and Seabra, D. and Salvador MÂª, C. and Braga, L. and Parreira, P. and Salgueiro-Oliveira, A. and Arreguy-Sena, C. and Oliveira, B. F. and Henriques MÂª, A. and Santos, J. and Lebre, S. and Marques, A. and Festas, C. and Rodrigues, S. and Ribeiro, A. and Lumini, J. and Figueiredo, A. G. and Hernandez-Martinez, F. J. and Campi, L. and Quintana-Montesdeoca MÂª, P. and Jimenez-Diaz, J. F. and Rodriguez-De-Vera, B. C. and Parente, A. and Mata MÂª, A. and Pereira, AmÂª and Fernandes, A. and BrÃ¡s, M. and Pinto MÂª, R. and Parreira, P. and Basto, M. L. and Rei, A. C. and MÃ³nico, L. M. and Sousa, G. and Morna, C. and Freitas, O. and Freitas, G. and Jardim, A. and Vasconcelos, R. and Horta, L. G. and Rosa, R. S. and Kranz, L. F. and Nugem, R. C. and Siqueira, M. S. and Bordin, R. and Kniess, R. and Lacerda, J. T. and Guedes, J. and Machado, I. and Almeida, S. and ZilhÃ£o, A. and Alves, H. and Ribeiro, Ã“ and Amaral, A. P. and Santos, A. and Monteiro, J. and Rocha MÂª, C. and Cruz, R. and Amaral, A. P. and LourenÃ§o, M. and Rocha MÂª, C. and Cruz, R. and Antunes, S. and MendonÃ§a, V. and Andrade, I. and OsÃ³rio, N. and Valado, A. and Caseiro, A. and Gabriel, A. and Martins, A. C. and Mendes, F. and Cabral, L. and Ferreira, M. and GonÃ§alves, A. and Luz, T. D. and Luz, L. and Martins, R. and Morgado, A. and Vale-Dias, M. L. and Porta-Nova, R. and Fleig, T. C. and Reuter Ã‰, M. and Froemming, M. B. and Guerreiro, S. L. and Carvalho, L. L. and Guedelha, D. and Coelho, P. and Pereira, A. and Calha, A. and Cordeiro, R. and GonÃ§alves, A. and Certo, A. and GalvÃ£o, A. and Mata MÂª, A. and Welter, A. and Pereira, E. and Ribeiro, S. and Kretzer, M. and JimÃ©nez-DÃ­az, J. F. |
| 1408 | Foreign Language | PROCES PIELÄ˜GNOWANIA PACJENTA Z PRZEWLEKÅYM ZAPALENIEM TRZUSTKI W UJÄ˜CIU MIÄ˜DZYNARODOWEJ KLASYFIKACJI PRAKTYKI PIELÄ˜GNIARSKIEJ ICNPÂ® | 2020 | Landowska, Paulina and Grabowska, Hanna |
| 1409 | Wrong outcome | Process Evaluation of a Randomised Controlled Trial for TeleClinical Care, a Smartphone-App Based Model of Care | 2021 | Indraratna, P. and Biswas, U. and Liu, H. and Redmond, S. J. and Yu, J. and Lovell, N. H. and Ooi, S. Y. |
| 1410 | Wrong outcome | Process Evaluation of an Ambulance-delivered Early Intensive Blood Pressure Lowering Stroke Trial: Design, Rationale, and Reflection | 2024 | Liu, R. and Chen, C. and Liu, F. and Lin, Y. and Chu, H. and Liu, H. and Anderson, C. S. and Yang, J. and Li, G. and Song, L. and Ouyang, M. |
| 1411 | Wrong outcome | Production of reliability proven physiological data for use in automated monitoring and diagnostic systems | 1991 | Rostron, D. W. and Hitchings, D. J. |
| 1412 | Wrong outcome | Productivity in nursing | 1977 | Friss, L. and White, M. |
| 1413 | Wrong outcome | Professional misconduct. The importance of measuring and recording vital signs correctly | 2006 | Castledine, G. |
| 1414 | Wrong outcome | The prognostic value of blood lactate levels relative to that of vital signs in the pre-hospital setting: a pilot study | 2008 | Jansen, T. C. and van Bommel, J. and Mulder, P. G. and Rommes, J. H. and Schieveld, S. J. and Bakker, J. |
| 1415 | Wrong outcome | Prognostic value of signs and symptoms in heart failure patients using remote telemonitoring | 2024 | Gingele, A. J. and Brandts, L. and Vossen, K. and Knackstedt, C. and Boyne, J. and Brunner-La Rocca, H. P. |
| 1416 | Wrong outcome | Project honeybee: Clinical applications for wearable biosensors | 2019 | Hartwell, L. and Ross, H. M. and La Belle, J. T. |
| 1417 | Wrong outcome | Project to Decrease the Incidence of Neonatal Hypothermia in the Newborn Center | 2019 | Kuan-Ying, Chen and Tzu-Yi, W. E. I. and Hsiao-Yu, Huang and Yun-Hsin, H. S. U. |
| 1418 | Wrong population | [Project to Decrease the Incidence of Neonatal Hypothermia in the Newborn Center] | 2019 | Chen, K. Y. and Wei, T. Y. and Huang, H. Y. and Hsu, Y. H. |
| 1419 | Wrong outcome | Prolonged field care (austere emergency care) principles in UK paramedic practice | 2023 | O'Kelly, Aebhric and Mallinson, Tom |
| 1420 | Wrong outcome | Prolonged recovery and delayed side effects of sedation for diagnostic imaging studies in children | 2000 | Malviya, S. and Voepel-Lewis, T. and Prochaska, G. and Tait, A. R. |
| 1421 | Wrong outcome | Promoting safer blood transfusion practice in hospital | 2007 | Parris, E. and Grant-Casey, J. |
| 1422 | Wrong outcome | Prophylactic Î²-blockade to prevent myocardial infarction perioperatively in high-risk patients who undergo general surgical procedures | 2003 | Taylor, R. C. and Pagliarello, G. |
| 1423 | Wrong outcome | Propofol anesthesia for invasive procedures in ambulatory and hospitalized children: experience in the pediatric intensive care unit | 1999 | Hertzog, J. H. and Campbell, J. K. and Dalton, H. J. and Hauser, G. J. |
| 1424 | Wrong outcome | [Propofol sedation administered by nurses for endoscopic procedures] | 2009 | Vilmann, P. and Hornslet, P. and Simmons, H. and Hammering, A. and Clementsen, P. |
| 1425 | Wrong outcome | Propranolol in infantile haemangioma: simplifying pretreatment monitoring | 2014 | El Ezzi, O. and Hohlfeld, J. and de Buys Roessingh, A. |
| 1426 | Wrong outcome | Prospective application of the interdisciplinary bedside rounding checklist 'TEMP' is associated with reduced infections and length of hospital stay | 2022 | Radhakrishnan, N. S. and Lukose, K. and Cartwright, R. and Sleiman, A. and Matey, N. and Lim, D. and LeGault, T. and Pollard, S. and Gravina, N. and Southwick, F. S. |
| 1427 | Wrong outcome | A prospective controlled trial of the effect of a multi-faceted intervention on early recognition and intervention in deteriorating hospital patients | 2010 | Mitchell, I. A. and McKay, H. and Van Leuvan, C. and Berry, R. and McCutcheon, C. and Avard, B. and Slater, N. and Neeman, T. and Lamberth, P. |
| 1428 | Wrong outcome | Prospective observational investigation of capnography and pulse oximetry monitoring after cesarean delivery with intrathecal morphine | 2019 | Weiniger, C. F. and Akdagli, S. and Turvall, E. and Deutsch, L. and Carvalho, B. |
| 1429 | Wrong outcome | Prospective study of tympanic temperature in patients undergoing haemopoietic transplant | 2010 | Meseguer-Liza, C. and Caravaca-HernÃ¡ndez, A. and Solano-Antolinos, J. and LÃ³pez-MartÃ­nez, S. and Meseguer-SÃ¡nchez, S. and SÃ¡nchez-MartÃ­nez, C. and Camargo-Portal, M. and Szendrei-Kovacs, J. |
| 1430 | Wrong outcome | A prospective validation of national early warning score in emergency intensive care unit patients at Beijing | 2015 | Liu, F. Y. and Qin, J. and Wang, R. X. and Fan, X. L. and Wang, J. and Sun, C. Y. and Cao, T. and Liang, X. |
| 1431 | Wrong outcome | Prospective, multi-center inverstigation on safety of critical patients in intensive care units | 2007 | Ma, P. L. and Xi, X. M. and Lin, H. Y. and Xu, Y. and Du, B. and Zhao, H. L. and Zhang, X. Y. Xiang-Yu and Zeng, L. |
| 1432 | Wrong outcome | [Prospective, multi-center investigation on safety of critical patients in intensive care units] | 2007 | Ma, P. L. and Xi, X. M. and Lin, H. Y. and Xu, Y. and Du, B. and Zhao, H. L. and Zhang, X. Y. and Zeng, L. |
| 1433 | Wrong outcome | A protocol for the development of a critical thinking assessment tool for nurses using a Delphi technique | 2017 | Jacob, E. and Duffield, C. and Jacob, D. |
| 1434 | Wrong outcome | Prototyping sensor network system for automatic vital signs collection. Evaluation of a location based automated assignment of measured vital signs to patients | 2013 | Kuroda, T. and Noma, H. and Naito, C. and Tada, M. and Yamanaka, H. and Takemura, T. and Nin, K. and Yoshihara, H. |
| 1435 | Wrong outcome | Psychometric properties of the Turkish version of the vital signs monitoring on pediatric wards scale (Ped-V scale) | 2023 | Demir KÃ¶sem, D. and BektaÅŸ, M. and Gawronski, O. |
| 1436 | Wrong outcome | [Psychosocial work factors associated to blood pressure and cardiovascular symptoms among Mexican nurses] | 2007 | JuÃ¡rez-GarcÃ­a, A. |
| 1437 | Wrong outcome | Public health nursing in Ireland: the general practitioners' view | 1992 | Hayes, C. H. and Hynes, M. and O'Herlihy, B. |
| 1438 | Wrong outcome | Pulse oximetry: an added criterion for discharge from the post-operative care unit | 1998 | Magboul, M. M. and Odugbesan, C. and el Dawlatly, A. A. and al Saud, S. |
| 1439 | Wrong outcome | The Purpose of Bedside Robots: Exploring the Needs of Inpatients and Healthcare Professionals | 2020 | Lee, Hyeongsuk and Piao, Meihua and Lee, Jisan and Byun, Ahjung and Kim, Jeongeun |
| 1440 | Wrong outcome | (QOL10) Corticosteroid Pulse Therapy--Hyperglycemia: Protocol for Capillary Blood Glucose Control...2020 Virtual Annual Meeting of the Consortium of Multiple Sclerosis Centers, May 26-29, 2020 | 2020 | Fernandes, Ivone R. and Sena, Ana C. and Paschoal, Maria A. and Franco, Adakson M. |
| 1441 | Wrong outcome | A qualitative description of nurses' problems to monitor and supervise vital signs in COVID-19 patients in isolation room | 2022 | Christina, Tri Yahya and Ismail, Suhartini and Erawati, Meira |
| 1442 | Wrong outcome | Qualitative evaluation of missed nursing care in neonatal intensive care units in a teaching hospital in Jordan | 2023 | Albsoul, R. A. and Alshyyab, M. A. and Albayyari, R. Y. and Alselaibi, D. H. and Flefil, S. A. and Jardaneh, L. H. and Dababseh, S. Y. F. and Al Odat, B. A. and Alkubaisi, F. A. and AlKhawaldeh, M. H. and FitzGerald, G. |
| 1443 | Wrong population | A qualitative exploration of the challenges providers experience during peripartum management of patients with a body mass index â‰¥ 50 kg/m2 and recommendations for improvement | 2024 | Kominiarek, M. A. and Lyleroehr, M. and Torres, J. |
| 1444 | Wrong outcome | A qualitative study examining the influences on situation awareness and the identification, mitigation and escalation of recognised patient risk | 2014 | Brady, P. W. and Goldenhar, L. M. |
| 1445 | Wrong outcome | A qualitative study exploring nurses' attitudes, confidence, and perceived barriers to implementing a traumatic brain injury nursing chart in Uganda | 2018 | Wynveen, L. and Gamble, M. and Nabulime, J. and Luggya, T. and Kalanzi, J. K. and Mowafi, H. |
| 1446 | Wrong population | A qualitative study of barriers and facilitators to pediatric early warning score (PEWS) implementation in a resource-limited setting | 2023 | Reuland, C. and Shi, G. and Deatras, M. and Ang, M. and Evangelista, P. P. G. and Shilkofski, N. |
| 1447 | Wrong outcome | A Quality Improvement Project to Improve the Utilization of an Intraoperative Rapid Response System | 2023 | Rama, A. and Qian, D. and Forbes, T. and Wang, E. and Knight, L. and Berg, M. and Caruso, T. J. |
| 1448 | Wrong outcome | Quality improvement project to increase screening and referral for biologic therapy for patients with uncontrolled asthma | 2022 | Davis, P. and Edie, A. H. and Rushton, S. and Cleven, K. |
| 1449 | Wrong outcome | Quality improvement strategies to improve inpatient management of small and sick newborns across All Babies Count supported hospitals in rural Rwanda | 2021 | Tuyisenge, D. and Byiringiro, S. and Manirakiza, M. L. and Mutsinzi, R. G. and Nshimyiryo, A. and Nyishime, M. and Hirschhorn, L. R. and Biziyaremye, F. and Gitera, J. and Beck, K. and Kirk, C. M. |
| 1450 | Wrong outcome | Quality management of nursing staff-based triage in an interdisciplinary emergency center: Development of an expanded advanced procedure for improving quality of the priority classification | 2013 | GrÃ¤ff, I. and SchÃ¶pfer, A. and Goldschmidt, B. and PlatzkÃ¶ster, C. and Glien, P. and Baumgarten, G. and Tenzer, D. |
| 1451 | Wrong population | Quality of care during childbirth at public health facilities in Bangladesh: a cross-sectional study using WHO/UNICEF 'Every Mother Every Newborn (EMEN)' standards | 2019 | Billah, S. M. and Chowdhury, M. A. K. and Khan, A. N. S. and Karim, F. and Hassan, A. and Zaka, N. and Arifeen, S. E. and Manu, A. |
| 1452 | Wrong outcome | Quality of care under casemix | 1994 | Duggan, J. M. |
| 1453 | Wrong outcome | Quality of post-anesthetic care in a hospital without a Post-Anesthetic Care Unit: A clinical audit | 2004 | Trevisan, P. and Gobber, G. |
| 1454 | Wrong outcome | A questionaire survey of present situation of ICU in Jiangsu province | 2006 | Li, G. M. and Wan, J. and Wang, J. Q. and Wang, X. and Xu, X. R. |
| 1455 | Wrong outcome | A questionaire survey on the current practices of respiratory care in intensive care unit in 30 provinces | 2009 | Li, J. and Zhan, Q. Y. and Liang, Z. A. and Du, M. L. and Dai, H. P. and Sun, B. and Yao, X. L. and Luo, Z. J. and Xia, J. G. and Wang, C. |
| 1456 | Wrong outcome | Quick change versus double pump while changing the infusion of inotropes: an experimental study | 2009 | de Barbieri, I. and Frigo, A. C. and Zampieron, A. |
| 1457 | Foreign Language | RabdomiÃ³lisis en un paciente con enfermedad de McArdle | 2021 | NafrÃ­a-Soria, H. and Moreno-EspaÃ±a, J. and SÃ¡nchez-Herrero, H. and GarcÃ­a-MenÃ©ndez, E. and Castillo, C. Moreno-Del and FernÃ¡ndez-Valle, I. |
| 1458 | Wrong outcome | Randomised controlled trial of patient controlled analgesia compared with nurse delivered analgesia in an emergency department | 2005 | Evans, E. and Turley, N. and Robinson, N. and Clancy, M. |
| 1459 | Wrong outcome | A randomized controlled trial of the effects of listening to non-commercial music on quality of nocturnal sleep and relaxation indices in patients in medical intensive care unit | 2013 | Su, C. P. and Lai, H. L. and Chang, E. T. and Yiin, L. M. and Perng, S. J. and Chen, P. W. |
| 1460 | Wrong outcome | A randomized trial of a supplemental alarm for critically low systolic blood pressure | 2015 | Panjasawatwong, K. and Sessler, D. I. and Stapelfeldt, W. H. and Mayers, D. B. and Mascha, E. J. and Yang, D. and Kurz, A. |
| 1461 | Wrong outcome | Rational selection of antibacterial drugs and postoperative nursing for gynecologic and obstetric surgery patients | 2018 | Hou, L. and Zhang, Y. and Luan, Y. and Xin, B. and Wang, C. |
| 1462 | Wrong outcome | RE-AUDIT OF ORTHOSTATIC BLOOD PRESSURE MEASUREMENT TO PREVENT FALLS AND FALL-RELATED COMPLICATIONS FOR GERIATRICS INPATIENTS FROM A SECONDARY CARE HOSPITAL...British Geriatrics Society Autumn Meeting, November 6-8, 2019, Leicester, England | 2020 | Sultana, Z. and Sarda, P. and Ang, S. Kim |
| 1463 | Wrong outcome | A Real-Time Wearable System for Monitoring Vital Signs of COVID-19 Patients in a Hospital Setting | 2021 | Santos, M. D. and Roman, C. and Pimentel, M. A. F. and Vollam, S. and Areia, C. and Young, L. and Watkinson, P. and Tarassenko, L. |
| 1464 | Wrong outcome | Recognising signs and symptoms of patient deterioration | 2012 | Felton, Michelle |
| 1465 | Wrong outcome | Recognizing acute patient deterioration...McColl A, Pesata F. When seconds matter: rapid response teams and nurse decision making. Nursing Management. February, 2016 | 2016 | Ruata, T. Annette |
| 1466 | Wrong outcome | Record review to explore the adequacy of post-operative vital signs monitoring using a local modified early warning score (mews) chart to evaluate outcomes | 2014 | Kyriacos, U. and Jelsma, J. and Jordan, S. |
| 1467 | Wrong outcome | Recording and interpretation of vital signs in a selected private hospital in the KwaZulu-Natal province of South Africa | 2020 | Graan, A. C. V. and Scrooby, B. and Bruin, Y. |
| 1468 | Wrong outcome | A recovery room-based acute pain service | 2007 | Leykin, Y. and Pellis, T. and Ambrosio, C. and Zanette, G. and Malisano, A. and Rapotec, A. and Casati, A. |
| 1469 | Wrong outcome | Recovery Room. Organization and clinical aspects | 2001 | Leykin, Y. and Costa, N. and Gullo, A. |
| 1470 | Wrong outcome | Recruiting patients to a digital self-management study whilst in hospital for a chronic obstructive pulmonary disease exacerbation: A feasibility analysis | 2021 | Whelan, M. and Biggs, C. and Areia, C. and King, E. and Lawson, B. and Newhouse, N. and Ding, X. and Velardo, C. and Bafadhel, M. and Tarassenko, L. and Watkinson, P. and Clifton, D. and Farmer, A. |
| 1471 | Wrong outcome | Reducing Morbidity and Mortality Rates from COVID-19, Influenza and Pneumococcal Illness in Nursing Homes and Long-Term Care Facilities by Vaccination and Comprehensive Infection Control Interventions | 2021 | Thomas, R. E. |
| 1472 | Wrong outcome | Reducing Sepsis Mortality | 2014 | Lopez-Bushneil, Kathy and Demaray, William S. and Jaco, Cathy |
| 1473 | Wrong outcome | Reduction of pain intensity for patients undergoing arterial sheath removal after coronary artery angioplasty: An interventional program | 2023 | Sallal, M. H. and Mousa, A. M. |
| 1474 | Wrong outcome | Reengineering the Discharge Transition Process of COVID-19 Patients Using Telemedicine, Remote Patient Monitoring, and Around-the-Clock Remote Patient Monitoring from the Emergency Department and Inpatient Units | 2021 | Kodama, R. and Arora, S. and Anand, S. and Choudhary, A. and Weingarten, J. and Francesco, N. and Chiricolo, G. and Silber, S. and Mehta, P. H. |
| 1475 | Wrong outcome | Referral challenges and outcomes of neonates received at Muhimbili National Hospital, Dar es Salaam, Tanzania | 2022 | Kiputa, M. and Salim, N. and Kunambi, P. P. and Massawe, A. |
| 1476 | Wrong outcome | [Refractory heart failure. Models of hospital, ambulatory, and home management] | 2002 | Oliva, F. and Alunni, G. |
| 1477 | Wrong outcome | Regional anaesthesia and propofol sedation for carotid endarterectomy | 2005 | Barringer, C. and Williams, J. M. and McCrirrick, A. and Earnshaw, J. J. |
| 1478 | Wrong outcome | Relationship between blood pressure, as measured by a nurse, by a doctor and under basal conditions, and micro-albuminuria and left ventricular mass | 1991 | De Blok, K. and Veerman, D. P. and Hoek, F. and De Koning, H. and Van Montfrans, G. A. |
| 1479 | Wrong outcome | The relationship between departmental culture and resuscitation-related moral distress among inpatient medical departments physicians and nurses | 2024 | Weill-Lotan, D. and Dekeyser-Ganz, F. and Benbenishty, J. |
| 1480 | Wrong outcome | The Relationship between Missed Nursing Care, Work Environment Conditions and Patient Safety Culture | 2023 | Amjad, Mahnaz Amiri and Jahani, Simin and Sayadi, Neda and Cheraghian, Bahman |
| 1481 | Wrong outcome | Relationship Between Nighttime Vital Sign Assessments and Acute Care Transfers in the Rehabilitation Inpatient | 2014 | Pellicane, Anthony J. |
| 1482 | Wrong outcome | Relationship Between Nursing Documentation and Patients' Mortality | 2013 | Collins, Sarah A. and Cato, Kenrick and Albers, David and Scott, Karen and Stetson, Peter D. and Bakken, Suzanne and Vawdrey, David K. |
| 1483 | Wrong outcome | The relationship between thermal comfort and light intensity with sleep quality and eye tiredness in shift work nurses | 2013 | Azmoon, Hiva and Dehghan, Habibollah and Akbari, Jafar and Souri, Shiva |
| 1484 | Wrong outcome | [Relationships among frequency domain and non-linear parameters from heart rate variability] | 2011 | Okamoto, Y. and Imai, R. and Yoshida, H. and Taeka, K. and Kuramoto, C. |
| 1485 | Wrong outcome | Relaxation training as a nursing intervention versus pro re nata medication | 1978 | Tamez, E. G. and Moore, M. J. and Brown, P. L. |
| 1486 | Wrong outcome | Reliability of a wearable wireless patch for continuous remote monitoring of vital signs in patients recovering from major surgery: a clinical validation study from the TRaCINg trial | 2019 | Downey, C. and Ng, S. and Jayne, D. and Wong, D. |
| 1487 | Wrong outcome | A remote monitoring and telephone nurse coaching intervention to reduce readmissions among patients with heart failure: study protocol for the Better Effectiveness After Transition - Heart Failure (BEAT-HF) randomized controlled trial | 2014 | Black, J. T. and Romano, P. S. and Sadeghi, B. and Auerbach, A. D. and Ganiats, T. G. and Greenfield, S. and Kaplan, S. H. and Ong, M. K. |
| 1488 | Wrong outcome | Remote monitoring for diabetes disorder: Pilot study using InDiaTel prototype | 2015 | Vivekanandan, S. and Devanand, M. |
| 1489 | Wrong outcome | Remote Monitoring of Critically-Ill Post-Surgical Patients: Lessons from a Biosensor Implementation Trial | 2021 | Restrepo, M. and Huffenberger, A. M. and Hanson, C. W., 3rd and Draugelis, M. and Laudanski, K. |
| 1490 | Wrong outcome | Remote Patient Monitoring Program for Hospital Discharged COVID-19 Patients | 2020 | Gordon, W. J. and Henderson, D. and DeSharone, A. and Fisher, H. N. and Judge, J. and Levine, D. M. and MacLean, L. and Sousa, D. and Su, M. Y. and Boxer, R. |
| 1491 | Wrong outcome | Remote vision-based digital patient monitoring of pulse and respiratory rates in acute medical wards | 2024 | Lewis, A. and Venugopal, B. and Gandhi, V. and Gibson, O. and Swanton, L. and Green, M. and Bowen, J. and Polkey, M. I. |
| 1492 | Wrong Study Design | Remote wireless vital signs monitoring on the ward for early detection of deteriorating patients: A case series | 2020 | Posthuma, L. M. and Downey, C. and Visscher, M. J. and Ghazali, D. A. and Joshi, M. and Ashrafian, H. and Khan, S. and Darzi, A. and Goldstone, J. and Preckel, B. |
| 1493 | Wrong outcome | Repeatability of SphygmoCor pulse wave analysis in assessing arterial wave reflection in pregnancy using applanation tonometry | 2014 | Crilly, M. A. and Orme, K. M. and Henderson, J. and Allan, A. J. and Bhattacharya, S. |
| 1494 | Wrong outcome | Reporting adverse events related to medical devices: A single center experience from a tertiary academic hospital | 2019 | Alsohime, F. and Temsah, M. H. and Hasan, G. and Al-Eyadhy, A. and Gulman, S. and Issa, H. and Alsohime, O. |
| 1495 | Wrong outcome | Reporting on implementation trials with null findings: the need for concurrent process evaluation reporting | 2022 | Sales, Anne |
| 1496 | Wrong outcome | Reporting on Neurological Decline as Identified by Hourly Neuroassessments | 2024 | Brazel, M. and Harris, J. and Carroll, D. and Davidson, J. and Levchak, P. J. and Malhotra, A. and LaBuzetta, J. N. |
| 1497 | Wrong outcome | Reproducibility of the measurement of plasma noradrenergic and dopaminergic metabolites in normal subjects | 1988 | Baker, N. J. and Adler, L. E. and Waldo, M. and Gerhardt, G. and Drebing, C. and Cox, B. and Berry, S. and Phillips, W. and Freedman, R. |
| 1498 | Wrong outcome | Resource Allocation: Stable Patients Remain Stable 12â€“24Â h Post-tPA | 2020 | Khan, S. and Soto, A. and Marsh, E. B. |
| 1499 | Wrong outcome | Respiratory assessment. Why don't nurses monitor the respiratory rates of patients? | 2006 | Hogan, J. |
| 1500 | Wrong outcome | Respiratory care practices and requirements for respiratory therapists in Beijing intensive care units | 2012 | Li, J. and Zhan, Q. Y. and Liang, Z. A. and Tu, M. L. and Sun, B. and Yao, X. L. and Luo, Z. J. and Xia, J. G. and Wang, C. |
| 1501 | Wrong outcome | Respiratory Care Practices and Requirements for Respiratory Therapists in Beijing Intensive Care Units | 2012 | Jie, Li and Qing Yuan, Zhan and Zong An, Liang and Mei Lien, Tu and Bing, Sun and Xiu Li, Yao and Zu Jin, Luo and Jin Gen, Xia and Chen, Wang |
| 1502 | Background Article | Respiratory rate 6: the benefits of continuous monitoring | 2018 | Dix, Ann |
| 1503 | Wrong outcome | Responding to the COVID-19 Outbreak in Singapore: Staff Protection and Staff Temperature and Sickness Surveillance Systems | 2020 | Htun, H. L. and Lim, D. W. and Kyaw, W. M. and Loh, W. N. J. and Lee, L. T. and Ang, B. and Chow, A. |
| 1504 | Wrong population | [Results of applying a paediatric early warning score system as a healthcare quality improvement plan] | 2016 | Rivero-MartÃ­n, M. J. and Prieto-MartÃ­nez, S. and GarcÃ­a-Solano, M. and Montilla-PÃ©rez, M. and Tena-MartÃ­n, E. and Ballesteros-GarcÃ­a, M. M. |
| 1505 | Wrong population | RETAIN: A Board Game That Improves Neonatal Resuscitation Knowledge Retention | 2019 | Cutumisu, M. and Patel, S. D. and Brown, M. R. G. and Fray, C. and von Hauff, P. and Jeffery, T. and SchmÃ¶lzer, G. M. |
| 1506 | Wrong Study Design | Retinopathy of prematurity screening examination and changes in vital signs | 2021 | Lahoti, S. and Jones, R. and Beck, K. D. and Padidam, S. and Apple, D. and Lin, X. and Quiram, P. and Young, R. C. and Desireddi, J. and Armitage Harper, C. |
| 1507 | Wrong outcome | A retrospective evaluation of the risk of bias in perioperative temperature metrics | 2019 | Freundlich, R. E. and Nelson, S. E. and Qiu, Y. and Ehrenfeld, J. M. and Sandberg, W. S. and Wanderer, J. P. |
| 1508 | Wrong outcome | A retrospective study of nursing diagnoses, outcomes, and interventions for patients admitted to a cardiology rehabilitation unit | 2011 | Zampieron, A. and Aldo, S. and Corso, M. |
| 1509 | Wrong outcome | A Retrospective Study of Nursing Diagnoses, Outcomes, and Interventions for Patients Admitted to a Cardiology Rehabilitation Unit | 2011 | Alessandra, Zampieron and Silla, Aldo and Marilisa, Corso |
| 1510 | Wrong outcome | A review of educational strategies to improve nurses' roles in recognizing and responding to deteriorating patients | 2011 | Liaw, S. Y. and Scherpbier, A. and Klainin-Yobas, P. and Rethans, J. J. |
| 1511 | Wrong outcome | A review of the use of insulin protocols to maintain normoglycaemia in high dependency patients | 2007 | Whitehorn, L. J. |
| 1512 | Wrong outcome | Review Title: Vital signs to monitor hospital patients: a systematic review | 2008 | Moola, Sandeep and Yifan, Xue and Lockwood, Craig and Schultz, Tim |
| 1513 | Wrong outcome | Review: Continuous Monitoring to Detect Failure to Rescue in Adult Postoperative Inpatients | 2018 | Verrillo, Sue Carol and Winters, Bradford D. |
| 1514 | Wrong outcome | Revised Winter-Tozer equation for normalized phenytoin concentrations in trauma and elderly patients with hypoalbuminemia | 1997 | Anderson, G. D. and Pak, C. and Doane, K. W. and Griffy, K. G. and Temkin, N. R. and Wilensky, A. J. and Winn, H. R. |
| 1515 | Wrong outcome | Rhabdomyolysis in a patient with McArdle's disease | 2021 | NafrÃ­a-Soria, H. and Moreno-EspaÃ±a, J. and SÃ¡nchez-Herrero, H. and GarcÃ­a-MenÃ©ndez, E. and Castillo, C. M. and FernÃ¡ndez-Valle, I. |
| 1516 | Wrong outcome | Risk determination after an acute myocardial infarction: review of 3 clinical risk prediction tools | 2012 | Scruth, E. A. and Page, K. and Cheng, E. and Campbell, M. and Worrall-Carter, L. |
| 1517 | Wrong outcome | Risk factors and outcomes associated with hospital admission for dehydration | 2008 | Wakefield, B. J. and Mentes, J. and Holman, J. E. and Culp, K. |
| 1518 | Wrong outcome | Risk factors for relaparotomy after cesarean section due to hemorrhage: a tertiary center experience | 2020 | Peker, Nurullah and Yavuz, Mustafa and AydÄ±n, Edip and Ege, Serhat and BademkÄ±ran, Muhammed Hanifi and Karacor, Talip |
| 1519 | Wrong outcome | A risk management audit: are we complying with the national guidelines for sedation by non-anaesthetists? | 1999 | Nicol, M. F. |
| 1520 | Wrong outcome | Risk of acute deterioration and care complexity individual factors associated with health outcomes in hospitalised patients with COVID-19: a multicentre cohort study | 2021 | Adamuz, J. and GonzÃ¡lez-Samartino, M. and JimÃ©nez-MartÃ­nez, E. and Tapia-PÃ©rez, M. and LÃ³pez-JimÃ©nez, M. M. and RodrÃ­guez-FernÃ¡ndez, H. and Castro-Navarro, T. and Zuriguel-PÃ©rez, E. and Carratala, J. and JuvÃ©-Udina, M. E. |
| 1521 | Wrong outcome | Risk predict model using multi-drug resistant organism infection from Neuro-ICU patients: a retrospective cohort study | 2023 | Jiang, H. and Pu, H. and Huang, N. |
| 1522 | Wrong outcome | Risks associated with removal of ventricular epicardial pacing wires after cardiac surgery | 1998 | Carroll, K. C. and Reeves, L. M. and Andersen, G. and Ray, F. M. and Clopton, P. L. and Shively, M. and Tarazi, R. Y. |
| 1523 | Wrong outcome | The role of capnography in endoscopy patients undergoing nurse-administered propofol sedation: A randomized study | 2013 | Slagelse, C. and Vilmann, P. and Hornslet, P. and Jorgensen, H. L. and Horsted, T. I. |
| 1524 | Wrong outcome | The Role of Health Care Quality in Hypertension Self-Management: A Qualitative Study of the Experience of Patients in a Public Hospital, North-West Ethiopia | 2021 | Hussien, M. and Muhye, A. and Abebe, F. and Ambaw, F. |
| 1525 | Wrong outcome | The role of intensive care nurses in cellular treatments during the COVIDâ€19 pandemic | 2024 | Caliskan, Figen and Ozdemir, Irem Nur and Zeydan, Ayten and Kandemir, Canan and Yilmaz, Rabia and Karaoz, Erdal and Adas, Gokhan Tolga |
| 1526 | Wrong outcome | ROLE OF MODIFIED EARLY WARNING SCORE IN EVALUATING MORTALITY IN POSTOPERATIVE PERIOD | 2023 | Nivedita and Mohammed, F. K. and Mohammad, N. A. |
| 1527 | Wrong outcome | The role of telemonitoring in caring for older people with long-term conditions | 2012 | Barrett, David |
| 1528 | Wrong outcome | The role of temperature in the detection and diagnosis of neutropenic sepsis in adult solid tumour cancer patients receiving chemotherapy | 2018 | Warnock, Clare and Totterdell, Peter and Tod, Angela Mary and Mead, Rachel and Gynn, Jamie-Lee and Hancock, Barry |
| 1529 | Wrong outcome | Role of the nurse in the multidisciplinary team approach to care of liver transplant patients | 1989 | Overman, J. A. and Cox, D. L. and Buchl, L. L. and Campion, J. K. and Raihle, P. C. and Sloan, T. R. |
| 1530 | Wrong outcome | The role of the radiology nurse | 1994 | Blevins, S. J. |
| 1531 | Wrong outcome | Routine Dyspnea Assessment on Unit Admission | 2013 | Baker, Kathy and Barsamian, Jennifer and Leone, Danielle and Donovan, Barbara C. and Williams, Donna and Carnevale, Kerry and Lansing, Robert and Banzett, Robert |
| 1532 | Wrong outcome | Rural Access to the Cancer Hospital at Home Care Model | 2024 | Nicholson, Bridget and Sloss, Elizabeth A. and Fausett, Angela and Davis, Chaz and Dumas, Kimberly and Littledike, Marcene and Mooney, Kathi |
| 1533 | Wrong population | Safe heat application for pediatric patients: A hot item | 2010 | Ebbinghaus, S. and Kobayashi, H. |
| 1534 | Wrong outcome | Safe transport combined with prospective nursing intervention in intra-hospital transport of emergency critically ill patients | 2016 | Jiang, X. X. and Wang, J. and Zhang, W. and Wang, X. J. and Meng, X. H. |
| 1535 | Wrong outcome | Safer and more efficient vital signs monitoring protocols to identify the deteriorating patients in the general hospital ward: an observational study | 2024 | Briggs, J. and Kostakis, I. and Meredith, P. and Dall'ora, C. and Darbyshire, J. and Gerry, S. and Griffiths, P. and Hope, J. and Jones, J. and Kovacs, C. and Lawrence, R. and Prytherch, D. and Watkinson, P. and Redfern, O. |
| 1536 | Wrong outcome | Safety analysis of proposed data-driven physiologic alarm parameters for hospitalized children | 2016 | Goel, Veena V. and Poole, Sarah F. and Longhurst, Christopher A. and Platchek, Terry S. and Pageler, Natalie M. and Sharek, Paul J. and Palma, Jonathan P. |
| 1537 | Wrong outcome | Safety of an ED High-Dose Opioid Protocol for Sickle Cell Disease Pain | 2015 | Tanabe, Paula and Martinovich, Zoran and Buckley, Barbara and Schmelzer, Annie and Paice, Judith A. |
| 1538 | Wrong outcome | Safety of assessment of patients with potential ischemic chest pain in an emergency department waiting room: A prospective comparative cohort study | 2010 | Scheuermeyer, F. X. and Christenson, J. and Innes, G. and Boychuk, B. and Yu, E. and Grafstein, E. |
| 1539 | Wrong outcome | The safety of automatic versus manual blood pressure cuffs for patients receiving thrombolytic therapy | 1998 | Saul, L. and Smith, J. and Mook, W. |
| 1540 | Wrong outcome | The safety of deep sedation with propofol controlled by the endoscopist in endoscopic retrograde cholangiopancreatography (ERCP): a prospective study in a tertiary hospital | 2018 | LuzÃ³n Solanas, L. and Ollero Domenche, L. and Sierra Moros, E. M. and Val PÃ©rez, J. and Soria San Teodoro, M. T. and GimÃ©nez JÃºlvez, T. and Uribarrena Amezaga, R. |
| 1541 | Wrong outcome | The safety of deep sedation with propofol controlled by the endoscopist in endoscopic retrograde cholangiopancreatography (ERCP): A prospective study in a tertiary hospital | 2018 | LuzÃ³n-Solanas, L. and Ollero-Domenche, L. and Sierra-Moros, E. M. and Val-PÃ©rez, J. and Soria-San-Teodoro, M. T. and GimÃ©nez-JÃºlvez, T. and Uribarrena-Amezaga, R. |
| 1542 | Wrong outcome | Safety of pain control with morphine: New (and old) aspects of morphine pharmacokinetics and pharmacodynamics | 2010 | Coetzee, J. F. |
| 1543 | Wrong outcome | The safety of passive hypothermia during assessment for hypoxic ischaemic encephalopathy | 2021 | Jayasinghe, D. and Wilcox, L. and Schoonakker, B. |
| 1544 | Wrong outcome | Safety of peripheral administration of phenylephrine in a neurologic intensive care unit: A pilot study | 2016 | Delgado, T. and Wolfe, B. and Davis, G. and Ansari, S. |
| 1545 | Wrong outcome | Safety of Prolonged Inhalation of Hydrogen Gas in Air in Healthy Adults | 2021 | Cole, A. R. and Sperotto, F. and DiNardo, J. A. and Carlisle, S. and Rivkin, M. J. and Sleeper, L. A. and Kheir, J. N. |
| 1546 | Wrong outcome | Sampling rate causes bias in APACHE II and SAPS II scores | 2000 | Suistomaa, M. and Kari, A. and Ruokonen, E. and Takala, J. |
| 1547 | Wrong outcome | Scoping Review of Early Intravenous Infiltration and Extravasation Detection Devices | 2023 | Kamada, Sneha and Mosier, Rebecca and El-Khalili, Taj and Triantis, Sophia and Yang, Robin |
| 1548 | Wrong outcome | A scoping review of real-time automated clinical deterioration alerts and evidence of impacts on hospitalised patient outcomes | 2022 | Blythe, R. and Parsons, R. and White, N. M. and Cook, D. and McPhail, S. |
| 1549 | Wrong Study Design | Screening for postoperative complications by continuous monitoring: protocol for the Biobeat-Postop cohort study | 2021 | Paternot, A. and Aegerter, P. and Martin, A. and Ouattara, J. and Ma, S. and Adjavon, S. and Trillat, B. and Alfonsi, P. and Fischler, M. and Le Guen, M. |
| 1550 | Wrong outcome | Screening for postoperative vital signs abnormalities, and particularly hemodynamic ones, by continuous monitoring: Protocol for the Biobeat-Postop cohort study | 2021 | Fischler, M. and Paternot, A. and Aegerter, P. and Martin, A. and Ouattara, J. and Ma, S. and Adjavon, S. and Trillat, B. and Alfonsi, P. and Le Guen, M. |
| 1551 | Wrong outcome | Sedation in adults receiving mechanical ventilation: Physiological and comfort outcomes | 2012 | Grap, M. J. and Munro, C. L. and Wetzel, P. A. and Best, A. M. and Ketchum, J. M. and Hamilton, V. A. and Arief, N. Y. and Pickler, R. and Sessler, C. N. |
| 1552 | Wrong outcome | The SedUROscopy Project: Sedation conducted by nurses supervised by the anaesthetist at a Lithotripsy and Endourology Unit in Spain | 2022 | Arguedas, Pilar and Cabello, Teresa and Escuder, Maite and MuÃ±oz, Maria Angeles and Orozco, Luisa and Sales, Elvira |
| 1553 | Wrong outcome | Selecting a Bedside Cognitive Vital Sign to Monitor Cognition in Hospital: Feasibility, Reliability, and Responsiveness of Logical Memory | 2019 | Nicholas, P. and O'Caoimh, R. and Gao, Y. and Habib, A. and Mross, T. K. and Clarnette, R. and Molloy, D. W. |
| 1554 | Wrong outcome | Selecting a bedside cognitive vital sign to monitor cognition in hospital: Feasibility, reliability, and responsiveness of logical memory | 2019 | Nicholas, P. and Oâ€™Caoimh, R. and Gao, Y. and Habib, A. and Mross, T. K. and Clarnette, R. and Molloy, D. W. |
| 1555 | Wrong outcome | Self-Performed Lung Ultrasound for Home Monitoring of a Patient Positive for Coronavirus Disease 2019 | 2020 | Pivetta, E. and Girard, E. and Locascio, F. and Lupia, E. and Martin, J. D. and Stone, M. |
| 1556 | Wrong Study Design | A self-powered spiral droplet triboelectric sensor for real-time monitoring of patient infusion in nursing wards | 2024 | Qian, L. and Wang, Y. and Qian, W. and Wang, Y. and Qian, J. |
| 1557 | Wrong outcome | Sequential audits of geriatric care: measuring change in structure and process and the contribution of clinical audit | 1998 | Brocklehurst, J. and Dickinson, E. and Windsor, J. |
| 1558 | Wrong outcome | A service evaluation of measuring fluid responsiveness in acutely unwell hypotensive patients outside of critical care | 2024 | Rossiter, Adam and Hilton, James Anthony and Fizza Haider, S. and Nasser, Syed M. T. and Boyer, Naomi and Cooper, Cath and Davis, Charlene and Marshall, Debbie and Skelding, Emma and Pike, Jennifer and Jarratt, Laura and Wood, Laura and Knight, Lucy and Holmes, Sophie and Cowman, Tamsin and Shepley, Elaine and Dubravac, Natalie and Gray, Wendy and Munday, Caz and Creagh-Brown, Ben |
| 1559 | Wrong outcome | Severe exacerbations of chronic obstructive pulmonary disease: Management with noninvasive ventilation on a general medicine ward | 2010 | Fiorino, S. and Detotto, E. and Battilana, M. and Bacchi-Reggiani, L. and Moretti, R. and Benfenati, F. and Caselli, A. and Marchi, S. and Testi, M. R. and Gallo, C. G. and Cuppini, A. and Kindt, G. and Moretti, M. |
| 1560 | Wrong outcome | Shift Work Disrupts Circadian Regulation of the Transcriptome in Hospital Nurses | 2019 | Resuehr, D. and Wu, G. and Johnson, R. L., Jr. and Young, M. E. and Hogenesch, J. B. and Gamble, K. L. |
| 1561 | Wrong outcome | Short and long-term outcomes from a multisession diabetes education program targeting low-income minority patients: a six-month follow up | 2013 | Ryan, J. G. and Jennings, T. and Vittoria, I. and Fedders, M. |
| 1562 | Wrong outcome | Short-Term Wearable Sensors for In-Hospital Medical and Surgical Patients: Mixed Methods Analysis of Patient Perspectives | 2021 | Joshi, M. and Archer, S. and Morbi, A. and Arora, S. and Kwasnicki, R. and Ashrafian, H. and Khan, S. and Cooke, G. and Darzi, A. |
| 1563 | Wrong outcome | Silence is golden--Developing an alarm management strategy for single-room patient care in a pediatric intensive care unit (PICU)...Canadian Association of Critical Care Nurses' Dynamics of Critical Care Conference 2019, September 16-18, 2019, Halifax, Nova Scotia | 2019 | Fudge, Hailey and Ellsmere, Barb and MacIntyre, Denise and Ryan, Mark and Soder, Chris and Abouelela, Marwan |
| 1564 | Wrong outcome | A simple non-physiological artifact filter for invasive arterial blood pressure monitoring: a study of 1852 trauma ICU patients | 2006 | Cao, H. and Norris, P. and Ozdas, A. and Jenkins, J. and Morris, J. A. |
| 1565 | Wrong outcome | [Simplified anesthesia protocol for cataract surgery under topical anesthesia: one year retrospective study] | 2013 | de Beketch, C. and Boissonnot, M. and Bernit, A. F. and Debaene, B. and Djabarouti, M. and Bouamama, N. and Dighiero, P. |
| 1566 | Wrong outcome | A single-pieced, fully air-driven, cuff-inserted pseudo-blood pressure generator for on-site pre-screening test of non-invasive blood pressure monitor by nurses | 2019 | Hwang, Y. J. and Kim, G. H. and Yun, S. U. and Nam, K. W. |
| 1567 | Wrong outcome | Skin temperature and vascular attributes as early warning signs of pressure injury | 2020 | Jiang, X. and Hou, X. and Dong, N. and Deng, H. and Wang, Y. and Ling, X. and Guo, H. and Zhang, L. and Cai, F. |
| 1568 | Wrong population | Skin-to-skin care alters regional ventilation in stable neonates | 2021 | Schinckel, N. F. and Hickey, L. and Perkins, E. J. and Pereira-Fantini, P. M. and Koeppenkastrop, S. and Stafford, I. and Dowse, G. and Tingay, D. G. |
| 1569 | Wrong outcome | Sleep in Aneurysmal Subarachnoid Hemorrhage Patients During Critical and Acute Care | 2021 | Fowler, Susan B. and Walker, Sarah and Jones, Timothy W. and Gell, Lauren |
| 1570 | Wrong outcome | Sleep in the Hospitalized Patient: Nurse and Patient Perceptions | 2016 | Vincensi, Barbara |
| 1571 | Wrong outcome | Sleep quality assessment of adults in care settings using non-wearable sleep trackers: Scoping review | 2024 | Yamakawa, M. and Kang, H. S. and Wang, H. and Konno, R. |
| 1572 | Wrong outcome | Sleep-disrupting effects of nocturnal nursing interventions in intensive care unit patients: A systematic review | 2021 | LocihovÃ¡, H. and Axmann, K. and Å½iakovÃ¡, K. |
| 1573 | Wrong outcome | Sleepiness in Spanish nursing staff - influence of chronotype and care unit in circadian rhythm impairment: research protocol | 2014 | Morenoâ€Casbas, MarÃ­a Teresa and Ruzafaâ€Martinez, Maria and Rol, Maria Angeles and Madrid, Juan Antonio and Serrano Pinto, Antonio and GonzÃ¡lezâ€MarÃ­a, Esther and Fuentelsazâ€Gallego, Carmen |
| 1574 | Wrong Study Design | A smart all-in-one device to measure vital signs in admitted patients | 2018 | Weenk, M. and van Goor, H. and van Acht, M. and Engelen, L. J. and van de Belt, T. H. and Bredie, S. J. H. |
| 1575 | Wrong outcome | A Smart Band for Automatic Supervision of Restrained Patients in a Hospital Environment | 2020 | MuÃ±iz, R. and DÃ­az, J. and MartÃ­nez, J. A. and NuÃ±o, F. and Bobes, J. and GarcÃ­a-Portilla, M. P. and SÃ¡iz, P. A. |
| 1576 | Wrong outcome | Smart respiratory monitoring: clinical development and validation of the IPIâ„¢ (Integrated Pulmonary Index) algorithm | 2017 | Ronen, M. and Weissbrod, R. and Overdyk, F. J. and Ajizian, S. |
| 1577 | Wrong outcome | A Smartphone-Based Model of Care to Support Patients With Cardiac Disease Transitioning From Hospital to the Community (TeleClinical Care): Pilot Randomized Controlled Trial | 2022 | Indraratna, P. and Biswas, U. and McVeigh, J. and Mamo, A. and Magdy, J. and Vickers, D. and Watkins, E. and Ziegl, A. and Liu, H. and Cholerton, N. and Li, J. and Holgate, K. and Fildes, J. and Gallagher, R. and Ferry, C. and Jan, S. and Briggs, N. and Schreier, G. and Redmond, S. J. and Loh, E. and Yu, J. and Lovell, N. H. and Ooi, S. Y. |
| 1578 | Wrong outcome | SMILE - Supporting multi-morbidity self-care through Integration, Learning and eHealth...21st International Conference on Integrated Care (Virtual), May 1-31, 2021 | 2022 | Burke, Mary |
| 1579 | Wrong outcome | The SMILe integrated care model in allogeneic SteM cell TransplantatIon faciLitated by eHealth: a protocol for a hybrid effectiveness-implementation randomised controlled trial | 2022 | De Geest, S. and Valenta, S. and Ribaut, J. and Gerull, S. and Mielke, J. and Simon, M. and Bartakova, J. and Kaier, K. and Eckstein, J. and Leppla, L. and Teynor, A. |
| 1580 | Wrong outcome | Snack and RelaxÂ® | 2016 | Markwell, Perpetua and Polivka, Barbara J. and Morris, Katrina and Ryan, Carol and Taylor, Annetra |
| 1581 | Wrong outcome | Society of Critical Care Medicine Guidelines on Recognizing and Responding to Clinical Deterioration Outside the ICU: 2023 | 2024 | Honarmand, K. and Wax, R. S. and Penoyer, D. and Lighthall, G. and Danesh, V. and Rochwerg, B. and Cheatham, M. L. and Davis, D. P. and DeVita, M. and Downar, J. and Edelson, D. and Fox-Robichaud, A. and Fujitani, S. and Fuller, R. M. and Haskell, H. and Inada-Kim, M. and Jones, D. and Kumar, A. and Olsen, K. M. and Rowley, D. D. and Welch, J. and Baldisseri, M. R. and Kellett, J. and Knowles, H. and Shipley, J. K. and Kolb, P. and Wax, S. P. and Hecht, J. D. and Sebat, F. |
| 1582 | Wrong outcome | [Sociodemographic and clinical profile of a nursing team with high blood pressure] | 2011 | CustÃ³dio, I. L. and Lima, F. E. and Almeida, M. I. and Silva Lde, F. and Monteiro, A. R. |
| 1583 | Wrong outcome | SSSH: Responsive soothing bassinet feasibility study for infants with congenital heart disease after cardiac surgery | 2023 | Lysaught, S. and Erickson, L. and Marshall, J. and Feldman, K. |
| 1584 | Wrong outcome | Stability of etoposide solutions in disposable infusion devices for day hospital cancer practices | 2014 | Klasen, A. and Kessari, R. and Mercier, L. and Valade, C. and Grill, J. and Desmaris, R. and Paci, A. |
| 1585 | Wrong outcome | Staff knowledge of orthostatic vital signs measurement | 2022 | Schell, Kathleen and Lyons, Denise L. |
| 1586 | Wrong outcome | Staff perspectives on the influence of patient characteristics on alarm management in the intensive care unit: a cross-sectional survey study | 2023 | Balzer, F. and Agha-Mir-Salim, L. and Ziemert, N. and Schmieding, M. and Mosch, L. and Prendke, M. and Wunderlich, M. M. and Memmert, B. and Spies, C. and Poncette, A. S. |
| 1587 | Wrong population | A standard protocol for blood pressure measurement in the newborn | 1997 | Nwankwo, M. U. and Lorenz, J. M. and Gardiner, J. C. |
| 1588 | Wrong outcome | Standardization of approaches to early detection of risks for clinical deterioration in children treated in hospitals for infectious diseases | 2023 | Solodovnikova, O. N. and Dyagileva, A. Yu and Erovichenkov, A. A. and Troshansky, D. V. and Gosteva, O. M. and Nurpeisova, A. K. and Khlypovka, Y. N. and Tsygankov, A. E. and Shakaryan, A. K. and Ipatov, N. S. and Tyurin, I. N. and Protsenko, D. N. |
| 1589 | Wrong outcome | Standardized Skin Flap Warming Effectively Improves Flap Survival without Obstructing Temperature Monitoring after DIEP | 2022 | Wang, Y. and Wu, G. and Chu, C. and Li, X. and Zou, Q. and Cao, Y. and Zhu, L. |
| 1590 | Wrong outcome | Standards of practice in UK emergency departments before, during and after conscious sedation | 2008 | Adams, S. T. and Woods, C. and Lyall, H. and Higson, M. and Adams, S. T. and Woods, C. and Lyall, H. and Higson, M. |
| 1591 | Wrong outcome | Starting out: I learned a more professional approach to observations | 2008 | Thompson, L. |
| 1592 | Wrong population | The state of India's neonatal units in the mid-nineties | 1997 | Singh, M. and Paul, V. K. and Deorari, A. K. |
| 1593 | Wrong outcome | Stevens-Johnson Syndrome/Toxic epidermal necrolysis complicated with fulminant type 1 diabetes mellitus: a case report and literature review | 2024 | Zhang, X. and Huang, D. and Lou, D. and Si, X. and Mao, J. |
| 1594 | Wrong outcome | Strategies for the Facilitation of Self-Leadership Among Ward Nurses in a Nurse-Led Critical Care Outreach Service | 2023 | Prinsloo, C. |
| 1595 | Wrong outcome | Stratification of patient blood testing pathways through predicting deterioration in renal and hepatic function...British Oncology Pharmacy Association (BOPA) 25th Annual Symposium, October 7-9, 2022, Liverpool, United Kingdom | 2023 | Chambers, Pinkie and Watson, Matthew and Sebastian, Masento and Buroyne, Rebecca and Al Moubayed, Noura |
| 1596 | Wrong outcome | Strengthening the afferent limb of rapid response systems: an educational intervention using web-based learning for early recognition and responding to deteriorating patients | 2016 | Liaw, S. Y. and Wong, L. F. and Ang, S. B. and Ho, J. T. and Siau, C. and Ang, E. N. |
| 1597 | Wrong outcome | Strengthening the afferent limb of rapid response systems: an educational intervention using web-based learning for early recognition and responding to deteriorating patients | 2016 | Sok Ying, Liaw and Lai Fun, Wong and Bee Leng Ang, Sophia and Tze Yin Ho, Jasmine and Chiang, Siau and Neo Kim Ang, Emily |
| 1598 | Wrong outcome | Strengthening the afferent limb of rapid response systems: An educational intervention using web-based learning for early recognition and responding to deteriorating patients | 2016 | Liaw, S. Y. and Wong, L. F. and Ang, S. B. L. and Ho, J. T. Y. and Siau, C. and Ang, E. N. K. |
| 1599 | Wrong outcome | Stress in telephone helpline nurses: research protocol for a study of theoretical determinants, physiological aspects and behavioural consequences | 2009 | Allan, J. and Farquharson, B. and Choudhary, C. and Johnston, D. W. and Jones, M. C. and Johnston, M. |
| 1600 | Wrong outcome | A Stress Relief App Intervention for Newly Employed Nursing Staff: Quasi-Experimental Design | 2019 | Chang, I. C. and Cheng, W. C. and Kung, W. C. |
| 1601 | Wrong outcome | Stressor Combat Strategies and Motivating Factors Among Health Care Service Providers During COVID-19 Pandemic | 2021 | Srivastava, A. and Srivastava, S. and Upadhyay, R. and Gupta, R. and Jakhar, K. and Pandey, R. |
| 1602 | Wrong outcome | Stressors in intensive cardiac care units: Patients' perceptions | 2022 | Coelho, A. C. and Santos, V. B. and de Barros, Albl |
| 1603 | Wrong outcome | Stroke patients' perceptions of home blood pressure monitoring: a qualitative study | 2011 | Ovaisi, S. and Ibison, J. and Leontowitsch, M. and Cloud, G. and Oakeshott, P. and Kerry, S. |
| 1604 | Wrong outcome | Stroke, part 1: opening the window of opportunity for treating acute ischemic stroke | 2011 | Mink, J. and Miller, J. |
| 1605 | Wrong population | The STRONGkids nutritional risk screening tool can be used by paediatric nurses to identify hospitalised children at risk | 2014 | Moeeni, V. and Walls, T. and Day, A. S. |
| 1606 | Wrong outcome | The study of dietary patterns and their relationship to anthropometry in female nurses | 2017 | Abashzadeh, K. and Siassi, F. and Qorbani, M. and Koohdani, F. and Farasati, N. and Sotoudeh, G. |
| 1607 | Wrong outcome | Study of the relationship between vital signs monitoring and riker sedation-agitation scale | 2016 | Kalhori, R. P. and Jalali, A. and Mirzaei, M. |
| 1608 | Wrong outcome | A study of using dexmedetomidine in ventilator bundle treatment in an ICU | 2015 | Song, R. and Li, J. and Dong, C. and Yang, J. |
| 1609 | Wrong outcome | A study of using dexmedetomidine in ventilator bundle treatment in an ICU | 2015 | Ruixia, S. and Junyan, L. and Chenming, D. and Jing, Y. |
| 1610 | Wrong outcome | A study on bio-medical waste segregation monitoring in a tertiary care hospital at Telangana | 2020 | Shinde, S. S. and Marivina, K. B. and Shalini |
| 1611 | Wrong outcome | A study on the nature of interactions between direct-care staff and persons with developmental disabilities in institutional care | 2002 | Chan, J. S. L. and Yau, M. K. S. |
| 1612 | Wrong outcome | Study protocol for multicentre randomized controlled trial of HeLP (Heat Loss Prevention) in the delivery room | 2013 | Vohra, S. and Reilly, M. and Rac, V. E. and Bhaloo, Z. and Zayack, D. and Wimmer, J. and Vincer, M. and Ferrelli, K. and Kiss, A. and Soll, R. and Dunn, M. |
| 1613 | Wrong outcome | Study Protocol: A randomized controlled trial evaluating the effect of family-based behavioral treatment of childhood and adolescent obesity-The FABO-study | 2016 | SkjÃ¥kÃ¸degÃ¥rd, H. F. and Danielsen, Y. S. and Morken, M. and Linde, S. F. and Kolko, R. P. and Balantekin, K. N. and Wilfley, D. E. and JÃºlÃ­usson, P. B. |
| 1614 | Wrong outcome | Study shows how computerization affects nursing activities in ICU | 1977 | Tolbert, S. H. and Pertuz, A. E. |
| 1615 | Wrong outcome | Studying the effects of BIS monitoring (depth of anesthesia) in early extubation of patients candidated for non-emergency CABG | 2017 | Kamali, A. and Rostami, A. and Modir, H. and Shokrpour, M. |
| 1616 | Wrong outcome | Success of a multidisciplinary heart failure clinic for initiation and up-titration of key therapeutic agents | 2005 | Jain, A. and Mills, P. and Nunn, L. M. and Butler, J. and Luddington, L. and Ross, V. and Cliffe, P. and Ranjadayalan, K. and Timmis, A. D. |
| 1617 | Wrong outcome | Successful implementation of round-the-clock care in a virtual ward during the COVID-19 pandemic | 2022 | Wells, Emily and Taylor, Jessie Lever and Wilkes, Matt and Prosser-Snelling, Ed |
| 1618 | Wrong outcome | SUCCESSFUL UPTAKE OF A TELEHEALTH REMOTE MONITORING AND PEER SUPPORT PROGRAM FOR OSTOMATES...Scientific and Clinical Abstracts From WOCNext 2020 Reimagined, June 5-7, 2020 | 2020 | Fearn, Robert and Mehta, Saahil and Belk, Rebekka and Dinh, Binh |
| 1619 | Wrong outcome | [Suicidal fall from height after restraint or accident?] | 2015 | WÃ¶llner, K. and Ortmann, J. and Kernbach-Wighton, G. and Madea, B. |
| 1620 | Wrong outcome | Supply-side barriers to maternal health care utilization at health sub-centers in India | 2016 | Singh, A. |
| 1621 | Wrong outcome | SUPPORTIVE ONCOLOGY CARE AT HOME INTERVENTION FOR PATIENTS WITH PANCREATIC CANCER...47th Annual Oncology Nursing Society Congress, April 27â€“May 1, 2022, Anaheim, CA | 2022 | Smith, Melissa and Nipp, Ryan and Shulman, Eliza and El-Jawahri, Areej and Brown, Patricia and Bergeron-Noa, Marcy |
| 1622 | Wrong outcome | Surgical High Dependency Admissions after Elective Laparoscopic Colorectal Resections: Is It Truly Necessary? | 2024 | Tan, J. K. H. and Koh, W. L. and Peh, C. H. and Lee, A. W. X. and Lau, J. and Chee, C. and Tan, K. K. |
| 1623 | Wrong outcome | Surveillance and monitoring of blood pressure and electrocardiogram in a stroke unit | 2011 | GÃ³mez, Luisa GÃ³mez and Prada, Isabel Curto |
| 1624 | Wrong outcome | Surveillance nursing diagnoses, ongoing assessment and outcomes on in-patients who suffered a cardiorespiratory arrest | 2017 | Maria-EulÃ lia, JuvÃ©-Udina and NÃºria, Fabrellas-PadrÃ©s and Jordi, Adamuz-TomÃ¡s and SÃ²nia, Cadenas-GonzÃ¡lez and Maribel, Gonzalez-Samartino and Laura de la Cueva, Ariza and Pilar, Delgado-Hito |
| 1625 | Wrong outcome | Surveillance of high-risk early postsurgical patients for real-time detection of complications using wireless monitoring (SHEPHERD study): results of a randomized multicenter stepped wedge cluster trial | 2023 | Posthuma, L. M. and Breteler, M. J. M. and Lirk, P. B. and Nieveen van Dijkum, E. J. and Visscher, M. J. and Breel, J. S. and Wensing, Cagl and Schenk, J. and Vlaskamp, L. B. and van Rossum, M. C. and Ruurda, J. P. and Dijkgraaf, M. G. W. and Hollmann, M. W. and Kalkman, C. J. and Preckel, B. |
| 1626 | Wrong outcome | A survey of first year student nurses' experiences of learning blood pressure measurement | 2009 | Baillie, L. and Curzio, J. |
| 1627 | Wrong population | A survey of healthcare professionals' experiences with the Paediatric Early Warning Score (PEWS) | 2018 | SÃ¸nning, Kjersti and Nyrud, Cathrine and Ravn, Ingrid Helen |
| 1628 | Wrong outcome | A survey of hospital outpatient services for chronic diseases in Gauteng | 2000 | Kalk, W. J. and Veriawa, Y. and Osler, C. |
| 1629 | Wrong population | A survey of procedural sedation and analgesia practices in pediatric oncology centers in India | 2012 | Arora, R. S. and Kulkarni, K. P. and Alston, R. D. |
| 1630 | Wrong outcome | A survey of the perceptions and knowledge of anaesthesia and anaesthetists among Grade 12 learners in four Johannesburg districts | 2023 | Talane, P. A. and Mamoojee, A. and Madima, N. |
| 1631 | Wrong outcome | Survey of wastage from intravenous admixture in US hospitals | 1993 | Birdwell, S. W. and Meyer, G. E. and Scheckelhoff, D. J. and Giambrone, C. S. and Iteen, S. A. |
| 1632 | Wrong population | Survey on monitoring analgesia and sedation in the Italian pediatric intensive care units | 2017 | Ta Bacco, B. and Tacconi, C. and Amigoni, A. |
| 1633 | Wrong outcome | Sustained effectiveness of a primary-team-based rapid response system | 2012 | Howell, M. D. and Ngo, L. and Folcarelli, P. and Yang, J. and Mottley, L. and Marcantonio, E. R. and Sands, K. E. and Moorman, D. and Aronson, M. D. |
| 1634 | Wrong outcome | Swift take-up of standardised early warning system across NHS trusts | 2013 | Sprinks, Jennifer |
| 1635 | Wrong outcome | Symposium on intensive care: 1. Monitoring of the critically ill surgical patient | 1978 | Allardyce, D. B. |
| 1636 | Wrong outcome | Synthetic cannabinoid use in a case series of patients with psychosis presenting to acute psychiatric settings: Clinical presentation and management issues | 2018 | Bonaccorso, S. and Metastasio, A. and Ricciardi, A. and Stewart, N. and Jamal, L. and Rujully, N. U. D. and Theleritis, C. and Ferracuti, S. and Ducci, G. and Schifano, F. |
| 1637 | Wrong outcome | A SYSTEMATIC REVIEW AND META-ANALYSIS OF THE EVALUATION OF THE CORRELATION BETWEEN THE VARIOUS PARAMETERS MONITORED DURING CARDIOTHORACIC SURGERY, VARIOUS DRUGS, AND POST-OPERATIVE RECOVERY | 2023 | Lu, G. and Guo, Y. and Zhang, H. |
| 1638 | Wrong outcome | A Systematic Review of Early Warning Systems' Effects on Nurses' Clinical Performance and Adverse Events Among Deteriorating Ward Patients | 2020 | Lee, J. R. and Kim, E. M. and Kim, S. A. and Oh, E. G. |
| 1639 | Wrong outcome | A systematic review of the effects of home blood pressure monitoring on medication adherence | 2006 | Ogedegbe, G. and Schoenthaler, A. |
| 1640 | Wrong outcome | A systems approach to immediate evaluation and management of hyperacute stroke | 1997 | Levine, S. R. |
| 1641 | Wrong outcome | Targeted Temperature Modulation in the Neuroscience Patient | 2016 | Wilson, M. and Della Penna, A. |
| 1642 | Wrong outcome | TASC (Telehealth After Stroke Care): a study protocol for a randomized controlled feasibility trial of telehealth-enabled multidisciplinary stroke care in an underserved urban setting | 2022 | Naqvi, I. A. and Cheung, Y. K. and Strobino, K. and Li, H. and Tom, S. E. and Husaini, Z. and Williams, O. A. and Marshall, R. S. and Arcia, A. and Kronish, I. M. and Elkind, M. S. V. |
| 1643 | Wrong outcome | Taste, temperature, and presentation predict satisfaction with foodservices in a Canadian continuing-care hospital | 1997 | O'Hara P, A. and Harper, D. W. and Kangas, M. and Dubeau, J. and Borsutzky, C. and Lemire, N. |
| 1644 | Wrong population | Teaching paediatric ward teams to recognise and manage the deteriorating child | 2014 | Tume, Lyvonne N. and Sefton, Gerri and Arrowsmith, Pete |
| 1645 | Wrong outcome | Technical article: Overview of hospital-based data capture systems that acquire continuous ECG and physiologic data | 2024 | Pelter, M. M. and Prasad, P. A. and Mortara, D. W. and Badilini, F. |
| 1646 | Wrong outcome | Technological aided assessment of the acutely ill patient â€“ The case of postoperative complications | 2017 | Haahr-RaunkjÃ¦r, C. and Meyhoff, C. S. and SÃ¸rensen, H. B. D. and Olsen, R. M. and Aasvang, E. K. |
| 1647 | Wrong outcome | Technology and the issues facing nursing assessment | 2015 | Ansell, Helen and Meyer, Alannah and Thompson, Shona |
| 1648 | Wrong outcome | Technology-supported apprenticeship in the management of hypertension: A randomized controlled trial | 2014 | Moore, J. O. and Marshall, M. A. and Judge, D. C. and Moss, F. H. and Gilroy, S. J. and Crocker, J. B. and Zusman, R. M. |
| 1649 | Wrong outcome | Tele-homecare for chronically-ill patients: Improved outcomes and new developments | 2004 | Robinson, S. and Stroetmann, K. and Stroetmann, V. |
| 1650 | Wrong outcome | Tele-Rapid Response Team (Tele-RRT): The effect of implementing patient safety network system on outcomes of medical patients-A before and after cohort study | 2022 | Balshi, A. N. and Al-Odat, M. A. and Alharthy, A. M. and Alshaya, R. A. and Alenzi, H. M. and Dambung, A. S. and Mhawish, H. and Altamimi, S. M. and Aletreby, W. T. |
| 1651 | Wrong outcome | Telehealth and Telecare: A Real-Life Integrated Experience in the COVID-19 Pandemic | 2022 | Bernocchi, P. and Bonometti, F. and Serlini, M. and Assoni, G. and Zanardini, M. and Pasotti, E. and Guerrini, S. and Scalvini, S. |
| 1652 | Wrong outcome | Telehealth remote monitoring for community-dwelling older adults with chronic obstructive pulmonary disease | 2013 | De San Miguel, K. and Smith, J. and Lewin, G. |
| 1653 | Wrong outcome | Telehealth with remote blood pressure monitoring for postpartum hypertension: A prospective single-cohort feasibility study | 2019 | Hoppe, K. K. and Williams, M. and Thomas, N. and Zella, J. B. and Drewry, A. and Kim, K. and Havighurst, T. and Johnson, H. M. |
| 1654 | Wrong outcome | Telehome monitoring reduced readmissions and improved quality of life in heart failure or angina | 2008 | Thompson, D. R. |
| 1655 | Wrong outcome | Telemedicine and Telehealth in Nursing Homes: An Integrative Review | 2021 | Groom, L. L. and McCarthy, M. M. and Stimpfel, A. W. and Brody, A. A. |
| 1656 | Wrong outcome | Telemedicine diabetes consultations are cost-effective, and effects on essential diabetes treatment parameters are similar to conventional treatment: 7-year results from the Svendborg Telemedicine Diabetes Project | 2013 | Levin, K. and Madsen, J. R. and Petersen, I. and Wanscher, C. E. and Hangaard, J. |
| 1657 | Wrong outcome | Telemedicine for Facio-Scapulo-Humeral Muscular Dystrophy: A multidisciplinary approach to improve quality of life and reduce hospitalization rate? | 2018 | Portaro, S. and CalabrÃ², R. S. and Bramanti, P. and Silvestri, G. and Torrisi, M. and Conti-Nibali, V. and Caliri, S. and Lunetta, C. and Alagna, B. and Naro, A. and Bramanti, A. |
| 1658 | Wrong outcome | Telemedicine for recently discharged older patients | 2010 | Cardozo, L. and Steinberg, J. |
| 1659 | Wrong outcome | Telemedicine Intensive Care Unit (Tele-ICU) Implementation During COVID-19: A Scoping Review | 2022 | Kemp Van Ee, S. and McKelvey, H. and Williams, T. and Shao, B. and Lin, W. T. and Luu, J. and Sunny, D. and Kumar, S. and Narayan, S. and Urdaneta, A. and Perez, L. and Schwab, H. and Riegle, S. and Jacobs, R. J. |
| 1660 | Wrong outcome | Telemedicine With Wearable Technologies in Patients Undergoing Hematopoietic Cell Transplantation and Chimeric Antigen Receptor T-Cell Therapy (TEL-HEMATO Study): Prospective Noninterventional Single-Center Study | 2024 | Hurtado, L. and Gonzalez Concepcion, M. and Flix-Valle, A. and Ruiz-Romeo, M. and Gonzalez-Rodriguez, S. and PeÃ±a, M. and Paviglianiti, A. and Pera Jambrina, M. A. and Sureda, A. and Ochoa-Arnedo, C. and Mussetti, A. |
| 1661 | Wrong outcome | Telemedicine-assisted home support for patients with advanced chronic obstructive pulmonary disease: Preliminary results after nine-month follow-up | 2005 | Vontetsianos, Th and Giovas, P. and Katsaras, Th and Rigopoulou, A. and Mpirmpa, G. and Giaboudakis, P. and Koyrelea, S. and Kontopyrgias, G. and Tsoulkas, B. |
| 1662 | Wrong outcome | Telemetry-based vital sign monitoring for ambulatory hospital patients | 2009 | Orphanidou, C. and Clifton, D. and Khan, S. and Smith, M. and Feldmar, J. and Tarassenko, L. |
| 1663 | Wrong population | Telemonitoring of high-risk neonates discharged from SNCU using a novel device: a pilot study | 2019 | Madireddy, A. and Lingaldinna, S. |
| 1664 | Wrong population | Telephone transmission of fetal heart rate monitor data. The experience at the University of Connecticut Health Center | 1986 | Vintzileos, A. M. and Montgomery, J. T. and Nochimson, D. J. and Campbell, W. A. and Weinbaum, P. J. and Blanchfield, M. P. and Blechner, J. N. |
| 1665 | Wrong outcome | Temperature -- the forgotten vital sign | 2005 | Smith, J. J. and Bland, S. A. and Mullett, S. |
| 1666 | Wrong outcome | Temperature measurement in critically ill orally intubated adults: a comparison of pulmonary artery core, tympanic, and oral methods | 1999 | Giuliano, K. K. and Scott, S. S. and Elliot, S. and Giuliano, A. J. |
| 1667 | Wrong population | Temperature monitoring in newborns: a comparison of thermometry and measurement sites | 2004 | Smith, L. S. |
| 1668 | Wrong outcome | Temperature monitoring of nonanaesthetised patients in a cardiac catheterisation laboratory | 2016 | Kennedy, Wendy and Conway, Aaron |
| 1669 | Wrong outcome | Temperature Variability during Delirium in ICU Patients: An Observational Study | 2013 | van der Kooi, A. W. and Kappen, T. H. and Raijmakers, R. J. and Zaal, I. J. and Slooter, A. J. C. |
| 1670 | Wrong outcome | Terminal digit bias in a specialty hypertension faculty practice | 2003 | Thavarajah, S. and White, W. B. and Mansoor, G. A. |
| 1671 | Wrong outcome | Testing effectiveness of the revised Cape Town modified early warning and SBAR systems: a pilot pragmatic parallel group randomised controlled trial | 2019 | Kyriacos, U. and Burger, D. and Jordan, S. |
| 1672 | Wrong outcome | Testing physiologic monitor alarm customization software to reduce alarm rates and improve nursesâ€™ experience of alarms in a medical intensive care unit | 2018 | Ruppel, H. and De Vaux, L. and Cooper, D. and Kunz, S. and Duller, B. and Funk, M. |
| 1673 | Wrong outcome | Testing the Feasibility of Sensor-Based Home Health Monitoring (TEC4Home) to Support the Convalescence of Patients With Heart Failure: Pre-Post Study | 2021 | Ho, K. and Novak Lauscher, H. and Cordeiro, J. and Hawkins, N. and Scheuermeyer, F. and Mitton, C. and Wong, H. and McGavin, C. and Ross, D. and Apantaku, G. and Karim, M. E. and Bhullar, A. and Abu-Laban, R. and Nixon, S. and Smith, T. |
| 1674 | Wrong outcome | The Thai Anesthesia Incidents Study (THAI Study) of anesthetic outcomes : II anesthetic profiles and adverse events | 2005 | Charuluxananan, S. and Punjasawadwong, Y. and Suraseranivongse, S. and Srisawasdi, S. and Kyokong, O. and Chinachoti, T. and Chanchayanon, T. and Rungreungvanich, M. and Thienthong, S. and Sirinan, C. and Rodanant, O. |
| 1675 | Wrong outcome | Therapeutic communication between nurses and patients from a hospital unit | 2010 | Negreiros, P. L. and Fernandes, M. O. and Macedo-Costa, K. N. F. and da Silva, G. R. F. |
| 1676 | Wrong outcome | Therapeutic Effect and Prognosis of PiCCO in the Treatment of Myocardial Injury Complicated with Septic Shock | 2022 | Lu, X. and Zhai, H. and Dong, Y. and Su, F. and Xie, Y. and Wang, Y. and Wang, L. and Li, J. and Xu, P. |
| 1677 | Wrong outcome | The therapeutic effect of irbesartan combined with nursing care in the treatment of patients with essential hypertension | 2021 | Teng, X. and Guo, K. and Gu, Y. and Liu, J. and Yang, X. and Ma, X. and Lv, W. |
| 1678 | Wrong outcome | Therapeutic hypothermia in the ICU: The nursing aspect | 2017 | Papageorgiou, D. and Tsikritsaki, K. and Javed, F. and Stathopoulos, T. and Ntinou, E. and Mavrogenis, A. F. and Koukoulitsios, G. |
| 1679 | Wrong outcome | Therapeutic Hypothermia Post--Cardiac Arrest | 2014 | Muhammad Manasia, Roshan Jan and Husain, Shahid Javed and Hooda, Khairunnissa and Imran, Mehrunnissa and Bailey, Carolyn |
| 1680 | Wrong outcome | 'There were more wires than him': the potential for wireless patient monitoring in neonatal intensive care | 2017 | Bonner, O. and Beardsall, K. and Crilly, N. and Lasenby, J. |
| 1681 | Wrong outcome | "They can rest at home": an observational study of patients' quality of sleep in an Australian hospital | 2018 | Delaney, L. J. and Currie, M. J. and Huang, H. C. and Lopez, V. and Van Haren, F. |
| 1682 | Wrong outcome | Thirty-six critical cases of emergency helicopter transferring between hospitals | 2021 | Yi, L. and Xiaoxia, L. and Huimin, Z. and Guang, Z. and Zhian, L. and Guojun, W. and Da, L. and Xiaowen, Z. and Jianfeng, Z. and Haojun, F. |
| 1683 | Wrong outcome | [Thirty-six critical cases of emergency helicopter transferring between hospitals] | 2021 | Li, Y. and Liao, X. and Zhao, H. and Zeng, G. and Ling, Z. and Wu, G. and Liu, D. and Zheng, X. and Zhang, J. and Fan, H. |
| 1684 | Wrong outcome | Thoracic impedance pneumography in propofol-sedated patients undergoing percutaneous endoscopic gastrostomy (PEG) placement in gastrointestinal endoscopy: A prospective, randomized trial | 2024 | Michael, F. A. and Hessz, D. and Graf, C. and Zimmer, C. and Nour, S. and Jung, M. and Kloka, J. and Knabe, M. and Welsch, C. and Blumenstein, I. and Dultz, G. and Finkelmeier, F. and Walter, D. and Mihm, U. and Lingwal, N. and Zeuzem, S. and Bojunga, J. and Friedrich-Rust, M. |
[truncated: 31,080 more chars]
