# Supplementary material for: Nurses’ experiences with inhospital continuous monitoring of vital signs in general wards: A systematic review
Source: PLOS Digit Health. 2025 Aug 22;4(8):e0000949. doi: 10.1371/journal.pdig.0000949 (PMC12373230; doi:10.1371/journal.pdig.0000949)
Supplement: S3 Text — (DOCX) [file pdig.0000949.s003.docx]

**Supplemental 3: Synthesis of findings**

1. **Emotional and practical advantages of working with continuous monitoring of vital signs on general wards.**

| Findings | Categories |
| --- | --- |
| CM increases the feelings of safety. (Weenk 2020)(unequivocal) | Patients feel saver knowing they are continuously monitored. |
| Nurses perceived that patients felt safer when they were monitored continuously (kooij)(unequivocal) |  |
| Nurses observed improved patient comfort and satisfaction using CM. (Becking-Verhaar 2023)(Credible) |  |
| CM provides a feeling of trust. (Especially during the night). (Van Noort, 2024)(Credible) | Continuous monitoring provides a feeling of trust for nurses. |
| Staff described becoming more selective in their use of the system over time, balancing the perception of increased patient safety with the drawbacks of the system.(Buss 2023)(Unequivocal) |  |
| Nurses perceived CM as an extra set of eyes. (Van Noort, 2024)(Unequivocal) |  |
| This was described as particularly useful when nursing staff had several unwell patients in their case load, to aid prioritization.(Buss 2023) (Unequivocal) |  |
| Most nurses experiences direct and continuous insight into their patients’ vital signs, enabling them to detect trends. (Becking-Verhaar 2023).(Credible) |  |
| Benefits to patient safety, it improves confidence in managing and prioritizing their caseload. (Areia)(Unequivocal) |  |
| Early recognition and intervention in deteriorating patients is the most critical advantage of CM. (Becking-Verhaar 2023) (unequivocal) | Continuous monitoring helps nurses detect deterioration in an earlier stage. |
| Nurses could recognise deterioration in patients’ vital signs at an early stage and prevent or alleviate adverse events through timely recognition and intervention. (Becking-Verhaar 2023) (unequivocal) |  |
| Positive experiences with regards to early detection of deterioration. (kooij) (unequivocal) |  |
| Nurses are able to detect deviations of vital signs earlier using regular trend analysis and recognized the importance of vital sign trends over the intermittent vital sign manual measurements, because of the insight in the periods between intermittent measurements, especially during the night. (Leenen 2022(2)) (credible) |  |
| When witnessing deviating trends and taking action as a result, the added value of CM had become clearer afterward. (Leenen 2023) (unequivocal) |  |
| Attitudes changed over time from sceptical to enthusiastic. CM became an essential part of care which facilitated them in anticipating patients’ clinical deterioration. (Van Noort, 2024) (unequivocal) |  |
| Earlier detection can result in earlier interventions. (Weenk 2020) (credible) |  |
| Changes in vital signs more quickly identified. (Areia) (unequivocal) |  |
| Clinical decision support is helpful for trend assessment, especially the D-EWS scores which were closely related to their conventional way of interpreting vital values with the MEWS system. (Leenen 2022(2)) (unequivocal) |  |
| CM enables them to act earlier on deviating vital signs than when using intermittent monitoring alone. (Leenen 2022(2)) (credible) |  |
| CM may contribute to earlier detection of clinical deterioration by better insight into the vital sign trends and thus increase the safety of care. (Leenen 2022(1)) (unequivocal) |  |
| CM enabled them to better monitor the effect of interventions in vital signs. (Leenen 2022(2)) (unequivocal) |  |
| CM saved time only when the device functioned adequately. Otherwise it is more time-consuming. (Becking-Verhaar 2023) (unequivocal) | Under the right circumstances, continuous monitoring can be time saving for the nurses, especially in shifts with high patient to nurse ratio. |
| Those who trusted the observations reported that the AMS assisted with managing their caseload as efficiently as possible by allowing them to assess observations without entering the patient's room.(Buss 2023)(Unequivocal) |  |
| CM saves time as it eliminated the need for measuring vital signs manually during routine rounds. (kooij) (Credible) |  |
| CM is especially useful during night shifts because of the higher patient-to-nurse ratio and the desire not to wake the patient (Leenen 2022(1)) (unequivocal) |  |
| Nurses hope CM can reduce workload of current routine manual measuring and registering vital signs, allowing them to be more productive and have more dedicated time for patient care. (Leenen 2022(1)) (unequivocal) |  |
| Prioritization of CM depends on the caseload during the shift. (Leenen 2023) (unequivocal) |  |
| Workload varied by type of shift, day shifts higher then night shifts. Actual intervention fidelity was not better during evenings or night. (Leenen 2023) (unequivocal) |  |
| CM can save time. (Weenk 2020) (unequivocal) |  |
| Staff described the system as valuable for watching the vital-signs trends of patients they were concerned about, while enabling them to continue with their clinical duties.(Buss 2023) (Unequivocal) |  |
| Benefits of CM during busy periods. May save time while increasing efficiency in prioritizing patients, identifying trends and detecting deterioration. (Areia) (Credible) |  |

1. **Practical disadvantages of working with continuous monitoring of vital signs.**

| Findings | Categories |
| --- | --- |
| Impact on reducing interactions between patient and nurses. Nurses value to visually observe the patient. (Areia) (unequivocal) | Patient related disadvantages, like mobility, interaction between nurse and patient, and distress in patients. |
| They emphasized the need for contact with patients to ensure safety and appropriate care delivery.(Buss 2023)(Unequivocal) |  |
| Nurses are afraid that interaction between patient and HCP would be reduced. (Weenk 2020) (unequivocal) |  |
| Design of the device can hinder patients during daily activities (too many cables, battery too big, stickers too tight). (Becking-Verhaar 2023) (unequivocal) |  |
| Opposite opinions about practicality of the device (Weenk 2020) (unequivocal) |  |
| Patients can become worried by being able to see their own vital signs. (Weenk 2020) (unequivocal) |  |
| Patients could become restless or obsessed with seeing their vital signs. (Becking-Verhaar 2023) (unequivocal) |  |
| Continuous monitoring (CM) could cause distress by making patients aware of their condition. (Areia) (unequivocal) |  |
| Battery too big, hampered the patient in daily activities. (Van Noort, 2024) (unequivocal) |  |
| Negative impact on mobility (Areia) (unequivocal) |  |
| Over-monitoring, measuring vital signs when the clinical added value was not clear, did provoke a tired feeling towards CM. (Van Noort, 2024) (unequivocal) | Over-monitoring of patients and causing a data overload of measurements by continuously monitoring to many patients. |
| Less need for assessing the vital sing trends in patient with an uncomplicated course. (Leenen 2023) (unequivocal) |  |
| While the remote monitoring was perceived to assist the nursing staff, there were also limitations associated with the system use, so careful con sideration had to be made about which patients would benefit from it the most.(Buss 2023)(Unequivocal) |  |
| Nurses do not feel de need to change current situation (MEWS). They are satisfied with current monitoring (MEWS) (kooij) (unequivocal) |  |
| Nurses consider there should be a clear rationale to measure vital signs at a high frequency. Patients with high risk of clinical deterioration have the best benefits of CM. (Leenen 2022(1)) (unequivocal) |  |
| Not all patients require CM, prioritize according to patient condition, staff levels and own clinical judgement. (Areia) (Credible) (unequivocal) |  |
| Overload of data due to CM. It enables nurses to make trend analysis, but this was still far from optimal. (Van Noort, 2024) (unequivocal) |  |
| CM can generate an overload of information. (Weenk 2020) (unequivocal) |  |
| One of the challenges clinical staff faced was the decision of who should be set up on the virtual monitoring system, given the limited number of devices available. (Buss, 2023) (Unequivocal) |  |
| CM would cost more time and increase workload. (Weenk 2020) (unequivocal) | Continuous monitoring can be a time consuming intervention |
| Increased workload, in case of deteriorating vital signs, nurses needed to check the patients and perform extra check-ups. (kooij) (unequivocal) |  |
| Duration of the intervention relates to the additional time involved with using it, for example to attach and activate the sensor. (kooij) (unequivocal) |  |
| Time-consuming elements are: assigning and connecting patients to the devices, calibrating devices, replacing parts and troubleshooting. (Mainly during day-shift) (Becking-Verhaar 2023) (Credible) |  |
| Many nurses identified this as challenging within their practice, requiring prioritization of vital signs observations within their workload. (Buss 2023)(Unequivocal) |  |
| Full range of vital signs is needed to measure an EWS, measuring more vital signs provides a more complete insight in the clinical status of the patient. Nurses miss measurements. (Leenen 2022(1)) (unequivocal) | Limitations of the sensor used in vital signs monitoring, like limited measurements and non-reliable measurements. |
| Staff recognized ongoing benefits of the system on a ward where most patients were in side-rooms and therefore not easily visi ble. The system was used as an adjunct to regular manual observation measurements which included blood pressure and temperature, which the system did not offer. In this way, staff described being able to get a quick overview of the well-being of their patients.(Buss 2023)(Unequivocal) |  |
| They reported the system was less useful for some groups of patients, including those with cold peripheries (this impeded the oxygen saturation monitoring); patients who were con fused or restless; and those who were anxious about their oxygenation levels.(Buss 2023)(Unequivocal) |  |
| Blood pressure not always a reliable estimation of the parameter. (Van Noort, 2024) (Credible) |  |
| Nurses occasionally doubted the measured vital signs’ reliability. (Becking-Verhaar 2023) (Credible) |  |
| Nurses were not satisfied with the quality of the sensor. (kooij) (unequivocal) |  |
| Device is not able to measure all the vital signs. (like core temperature) (Weenk 2020) (Credible) |  |
| Not usable for patients with pacemaker, CT scan, or when patient is taking a shower.(Kooij) (Credible) |  |
| Removing the sensor when performing diagnostics for the prevention of interference was considered a barrier. (ill patients) (Leenen 2023) (unequivocal) |  |
| Sensor needs to be able to measure more vital signs than only HR and ReR to result in time-saving benefits(Leenen 2023) (Credible) |  |
| Lack of available evidence to substantiate the use of CM with a limited number of vital signs in their patient population. (kooij) (unequivocal) |  |

1. **Important aspects regarding the implementation of continuous monitoring.**

| Findings | Categories |
| --- | --- |
| Nurses are afraid the ward would become like an ICU.(CM can lead to reluctance in transfer to the ICU) (Weenk 2020) (credible) | Continuous monitoring is not a substitute for an intensive care admission. |
| Cm should not lead to earlier discharge of patients from the ICU. Reason 1: Nurses fear that this might result in a higher workload and unsafe nursing care. Reason is the inability to respond to alarms immediately. (Leenen 2022(1)) (unequivocal) |  |
| Reason 2: high workload because of lower nurse-patient ratio. Also they believe not to have the technical nursing skills an knowledge of CM that ICU patients would need. (Leenen 2022(1)) (unequivocal) |  |
| General ward is organized different then an ICU, therefore CM is different. A clear definition of what CM of vital signs a general wards is, and what kind of boundaries are determined, would enhance clarity as to the expectations towards nurses. (Van Noort, 2024) (unequivocal) |  |
| Implementation: Facilitating factor to execute a task with a colleague. (kooij) (credible) | Aspects regarding the implementation process |
| Although there was local agreement that with the use of the AMS, the protocolized frequency of blood pressure monitoring could be reduced to lower the required room entries, many of the staff interviewed described discomfort with this.(Buss 2023)(Unequivocal) |  |
| Implementation: Nurses are positive about both formal communication (meetings) and informal communication with colleagues. (kooij) (unequivocal) |  |
| There was a formally appointed internal implementation leader, positive. (kooij) (unequivocal) |  |
| Personal characteristics affecting implementation such as younger age, experience with the intervention task will be beneficial, for example to execute tasks correctly and at a more rapid pace. (kooij) (credible) |  |
| Key-users were present for practical support (nurses with specific involvement in the project). (kooij) (unequivocal) |  |
| Nurses prefer higher volume of patients with CM instead of a few. As a result they were working with two different work processes. (Leenen 2022(1)) (unequivocal) |  |
| Communication and education about the technology and work process to all stakeholders was important. (Leenen 2022(2)) (credible) |  |
| Communication and education about the technology and work process to all stakeholders was important. (Leenen 2022(2)) (credible) |  |
| Perceptions about the possibility to test the intervention and whether they felt safe to try the intervention and make mistakes varied (kooij) (unequivocal) | Nurses perceived training and on-the-job coaching as crucial elements for effective utilization of continuous monitoring in their daily practice. |
| Dedicated project team and technical support are perceived important. (kooij) (credible) |  |
| Access to a manual and training about CM was perceived helpful. (kooij) (credible) |  |
| Evaluation of the implementation is perceived important, it provides insights into the status of the project. (kooij) (unequivocal) |  |
| Receiving training and education is conditional to acquire knowledge of the system and to be able to start with CM. (Leenen 2022(1)) (unequivocal) |  |
| Timing of training and dosage of the amount of information is considered important, preferably shortly before the start of the implementation and repeated regularly during implementation to keep their acquired knowledge up to date. (Leenen 2022(1)) (unequivocal) |  |
| Coaching by the project leader and key-users is considered supportive for learning on the job. (Leenen 2022(1)) (unequivocal) |  |
| Skills are best learned at the bedside. (Leenen 2022(1)) (unequivocal) |  |
| Nurses think it will be helpful to learn to interpret vital sign trends when a patient deteriorates while having CM. (Leenen 2022(1)) (unequivocal) |  |
| Importance of experiencing an adverse event when continuous monitoring was applied. (Leenen 2022(2)) (unequivocal) |  |
| Teaching-on-the-job by the researcher was desirable for adoption of the technology. (Leenen 2022(2)) (credible) (unequivocal) |  |
| Practical experience was convenient for their adoption and acceptability of the intervention. (Leenen 2022(2)) (credible) |  |
| Analysis of trends required experience because they were only used to interpret absolute values of the intermitting measurements of vital signs. (Leenen 2022(2)) (credible) |  |
| Vital signs values and trends must be measured reliably and the technology must not be defective. (Leenen 2022(2)) (credible) | The importance of a properly working technical infrastructure. |
| With patient safety being the foremost thought of the clinical staff interviewed, trust in the observations was shown to really be a vital component underlying how clinical staff utilized the AMS in practice(Buss 2023)(Unequivocal) |  |
| Doubts also arose due to problems connecting patients to ViSi Mobile and calibrating the device. (Becking-Verhaar 2023) (credible) |  |
| Nurses mention the importance of automated integration of continuous vital sign data in de EMR. (Leenen 2022(2)) (unequivocal) |  |
| Nurses preferred a CM system technically integrated into their existing mobile devices without restrictions in the range of the wireless connection. (Leenen 2022(1)) (unequivocal) |  |
| Nurses favour integration of vital sign trends into the EMR allowing more effective documentation, evaluation and productivity. (Leenen 2022(1)) (credible) |  |
| Pairing of the sensor with the software platform is a barriers for regular daily use (it uses a separate web-based application rather than via the regularly used phone). (Leenen 2023) (unequivocal) |  |
| Negative about technical infrastructure to support CM. Bad Wi-Fi connection and lack of interoperability with existing systems. (kooij) (unequivocal) |  |

1. **False alarms and the importance of clinical assessment**

| Findings | Categories |
| --- | --- |
| Nurses place trends in the perspective of their clinical assessment. (Leenen 2022(1)) (unequivocal) | Nurses stress the need for their own clinical assessment besides continuous monitoring and relying entirely on alarms. |
| It is necessary to take clinical status and context factors into account when assessing the vital sign trend, rather then just acting solely on the trend data. (Leenen 2022(2)) (unequivocal) |  |
| Most clinical staff interviewed highlighted the importance of hav ng confidence in what observations the system was displaying.(Buss 2023)(Unequivocal) |  |
| Importance of their clinical bedside assessment. (this is more then only vital sign measurement) (Leenen 2023) (unequivocal) |  |
| Trends were often a confirmation of their clinical perspective of the patient rather than it prompting them to reconsider their assessment. (Leenen 2023) (unequivocal) |  |
| Nurses do not fully trust the accuracy of the technology without physically assessing the patient. (discrepancies between what they observed and what the trend indicated) (Leenen 2023) (unequivocal) |  |
| Longer use of CM affects their alertness to changes. Critical note towards the possibility of only analysing trends, because small changes may be overlooked. (Van Noort, 2024) (unequivocal) |  |
| it would not eliminate the need and value of bedside nursing assessments during rounds. (Leenen 2023) (Credible) |  |
| Clinical view is still needed in addition to CM. (kooij) (credible) |  |
| They now understand all types of and reasons for alarms and that they handled the continuous data availability of vital signs efficiently. (Van Noort, 2024) (unequivocal) | Nurses describe strategies to reduce false alarms. |
| There is no added value of alarms if trend analysis was carried out according to the protocol used in this study. (Leenen 2022(2)) (unequivocal) |  |
| Nurses suggest user-adjustable alarm settings to decrease false alarm rate and prevent alarm fatigue. (Leenen 2022(1)) (unequivocal) |  |
| Adjust alarm setting to reduce noise and minimize false alarms (areia) (unequivocal) |  |
| Specific alarm strategies for deviating trends could be an alternative to timely detect deterioration. (Leenen 2023) (credible) |  |
| Noise from alarms lead to frustration and anxiety (Areia) (unequivocal) | Nurses experience feelings of uncertainty and agitation caused by the technical downsides of the specific monitoring systems used for continuous monitoring |
| Feeling of agitation about the alarms, potentially related to the extra workload caused by the need to respond to the alarms. (Leenen 2022(1)) (unequivocal) |  |
| The system generated too many and too many false alarms. These alarms were experienced as disruptive and caused feelings of uncertainty and lead to irritation. (Leenen 2022(1)) (unequivocal) |  |
| Feelings of uncertainty raised by alarms because of having doubts about their own clinical experience by receiving multiple and frequent alarms. (Leenen 2022(1)) (unequivocal) |  |
| Alarms are only desirable when they are fully reliable and not generating frequent false alarms and an alarm should require immediate follow-up by the nurse, such as taking extra vital sign measurements or notifying a doctor. (Leenen 2022(2)) (unequivocal) |  |
| Nurses are not able to continuously watch the vital signs, as they were not all the time in one of the rooms where the dashboards were available. (Van Noort, 2024) (unequivocal) |  |
| Incorrect cables led to measurement errors and false alarms. (Van Noort, 2024) (credible) |  |
| Nurses do not get a warning in case of abnormal vital signs of other patients, this gives a feeling of uncertainty. (Van Noort, 2024) (unequivocal) |  |
| Safety concerns related to changing alarm thresholds (minimize adjusting or guidance by doctor needed) (Areia) (unequivocal) |  |
| Safety concerns related to changing alarm thresholds (alarms not returned to their baseline settings before attaching to other patient. (Areia) (unequivocal) |  |
