# Supplementary material for: Nurses’ experiences with inhospital continuous monitoring of vital signs in general wards: A systematic review
Source: PLOS Digit Health. 2025 Aug 22;4(8):e0000949. doi: 10.1371/journal.pdig.0000949 (PMC12373230; doi:10.1371/journal.pdig.0000949)
Supplement: S4 Data — (DOCX) [file pdig.0000949.s004.docx]

**Supplementary File 4: All extracted data from al included studies**

| **Findings** | **Illustration from publication (Page number)** | **Evidence** | | | **Name data extractor**  **Date data extraction** |
| --- | --- | --- | --- | --- | --- |
|  |  | **Unequivocal** | **Credible** | **Unsupported** |  |
| Continuous monitoring (CM) could cause distress by making patients aware of their condition. (Areia, 2022) | 'Although we do try and reassure them that it’s because we are taking very good care of them and, you know, we want to see any signs of anything we can sort out. I think for patients it can seem quite a big deal and it tells them that perhaps they are sicker perhaps than they thought they were.' (815) | X |  |  | Berte van Zeist-de Jonge  17-03-2024 |
| Impact on reducing interactions between patient and nurses. Nurses value to visually observe the patient. (Areia, 2022) | 'I believe intuition is a great thing as well so experience and intuition. And you’ll be like this patient's not right. Although they could be triggering maybe a zero but you just know in you, you know because you know the patient, you just know there’s just something not right the patient could deteriorate after that' (816) | X |  |  | Berte van Zeist-de Jonge  17-03-2024 |
| Benefits to patient safety, it improves confidence in managing and prioritizing their caseload. (Areia, 2022) | 'It does feel safer, that's what I would say. And I just feel like, sort of that I am doing a better job, like, if I dunno, if you are picking up things quicker, you can solve anything that can go wrong quicker and you are more efficient. Um, if an alarm is going off when you are not there and the doctor sees it, then I feel like, it's their moral code they would do something (laughs) you would hope.' (816) | X |  |  | Berte van Zeist-de Jonge  17-03-2024 |
| Changes in vital signs more quickly identified. (Areia, 2022) | 'I guess you're more likely to pick up on things that are changing quite slowly if someone's blood pressure drops a little bit and you're doing it more thoroughly and more frequently you'd be able to act on that sooner rather than if you left it four hours and then saw a mas sive drop and you could intercept and do something about it.' (816) | X |  |  | Berte van Zeist-de Jonge  17-03-2024 |
| Negative impact on mobility (Areia, 2022) | 'They feel like they can't move with it on. Not that they want to lie there, it just makes it harder for them to mobilise and do things for themselves so, in some in stances it does actually increase our workload … it kind of ties the patient down and it affects other areas of their care' (816) | X |  |  | Berte van Zeist-de Jonge  17-03-2024 |
| Concern for confused patients, wires have the potential to increase risk of falls. (Areia, 2022) |  |  |  | X | Berte van Zeist-de Jonge  17-03-2024 |
| Noise from CM alarms leads to frustration and anxiety. (Areia, 2022) | 'I think it is very disconcerting for relatives. Apart from anything else,, as the nurses and doctors know they alarm frequently. If they lose the signal, because somebody has moved, or something like that, or the parameters haven't been set up in a certain way, then it will alarm constantly. And I think that relative perception of those alarms going off is that their relative is, is deteriorating and I think it can be quite panicking for them to hear beep beep beep when their relative is on the monitor.' (816) | X |  |  | Berte van Zeist-de Jonge  17-03-2024 |
| Adjusting alarm settings to reduce noise and minimize false alarms (Areia, 2022) | 'Well for example you have some patients who are known as COPD and they don't need to have satura tions of oxygen at around 94% so we normally set those alarms according to the patients' normal rates. For a pa tient who's got AF obviously and we know he's got AF as a basic we disconnect the alarm of the AF detector because it's just going to jump all the time and things like that.' (817) | X |  |  | Berte van Zeist-de Jonge  17-03-2024 |
| Safety concerns related to changing alarm thresholds (minimize adjusting or guidance by doctor needed) (Areia, 2022) | '…and what you would do, is in collaboration with the surgeon is find a range for target saturations and they might not be the 95- 100% that most people are. They might have a totally different set of parameters 88- 92 for example. (817) | X |  |  | Berte van Zeist-de Jonge  17-03-2024 |
| Safety concerns related to changing alarm thresholds (alarms not returned to their baseline settings before attaching to other patient. (Areia, 2022) | '… if you start fiddling around with the alarms you might change the alarms for this patient and not change them back when you've taken that monitoring off so the next patient that comes along may be sick and you haven't noticed it because the alarm hasn't gone off because it's been reset' (817) | X |  |  | Berte van Zeist-de Jonge  17-03-2024 |
| Benefits of CM during busy periods. May save time while increasing efficiency in prioritizing patients, identifying trends and detecting deterioration. (Areia, 2022) | 'Just because when you are busy doing other things you can make sure that, just from a glance that nothing has changed. That is the main thing. Especially with post- op patients, and you have got a really poorly pancreatitis patients that are acutely ill.' (817) |  | X |  | Berte van Zeist-de Jonge  17-03-2024 |
| Not all patients require CM, prioritize according to patient condition, staff levels and own clinical judgement. (Areia, 2022) | 'A patient which is physically fine … doesn't need a constant monitor, Just need to check regularly to make sure that everything is still going fine, … because most of the patients don't need the monitoring, sometimes they are [scoring] a 1 or a 2 it's their normal blood pressure, their normal heart rate which may be a bit higher or lower than normal, so basically you will have to take continuous monitoring for the patients who truly need it' (817) |  | X |  | Berte van Zeist-de Jonge  17-03-2024 |
| General tolerance is variable and linked to a number of factors including condition, severity, independence and expectations. (Areia, 2022) |  |  |  | X | Berte van Zeist-de Jonge  17-03-2024 |
| Approaches to encourage patients to wear CM, including reassurance, negotiating rest periods and explaining the purpose of monitoring. (Areia, 2022) | 'They sometimes like it removed. So then you have to compromise, and say “Ok, well we'll take it off for half an hour but I need to pop it back on”. Explain the need behind it and they are usually OK with it.' (817)  'Sometimes confused patients don't tolerate it. They don't understand it and they find it annoying and stuff and that's quite hard for them and so try and one- to- one them as much as possible to keep them calmer and understand so they don't rip it all off' (817) | X |  |  | Berte van Zeist-de Jonge  17-03-2024 |
| Most nurses experiences direct and continuous insight into their patients’ vital signs, enabling them to detect trends and compare and interpret current and previous measurements. (Becking-Verhaar 2023) | Last week, we had a successful resuscitation because CM showed a low heart rate. (6) |  | X |  | Berte van Zeist-de Jonge  22-03-2024 |
| Nurses could recognise deterioration in patients’ vital signs at an early stage and prevent or alleviate adverse events through timely recognition and intervention. (Becking-Verhaar 2023) | Last week, we had a successful resuscitation because CM showed a low heart rate.(6) | X |  |  | Berte van Zeist-de Jonge  22-03-2024 |
| Early recognition and intervention in deteriorating patients is the most critical advantage of CM. (Becking-Verhaar 2023) | This allows you to respond/act appropriately and in a timely manner when the device shows abnormal values and is not acted upon until the next monitoring moment. (6) | X |  |  | Berte van Zeist-de Jonge  22-03-2024 |
| CM saves time because it is easier and quicker to load vital signs into the files using CM than input them manually. Most notable in the evening and night shifts. (Becking-Verhaar 2023) | Onderbouwd met percentages. |  | X |  | Berte van Zeist-de Jonge  22-03-2024 |
| Time-consuming elements are: assigning and connecting patients to the devices, calibrating devices, replacing parts and troubleshooting. (Mainly during day-shift)  (Becking-Verhaar 2023) |  |  | X |  | Berte van Zeist-de Jonge  22-03-2024 |
| CM saved time only when the device functioned adequately. Otherwise it is more time-consuming.  (Becking-Verhaar 2023) | In the day and evening shift, it does give you some gain, but usually only 5-10 min because it almost never happens that all three to six are functioning. In the evening shift, maybe sometimes a little more time gain, because you have more patients there and especially when they all function. On the night shift, the morning round, it certainly saves a lot of time. But also only when they are functioning; otherwise, you still have to calibrate them or manually measure the controls. (7) | X |  |  | Berte van Zeist-de Jonge  22-03-2024 |
| Nurses observed improved patient comfort and satisfaction using CM.  (Becking-Verhaar 2023) | We don’t need to disturb patients as often, especially at night. Patients sleep better because of this, I think. (8) |  | X |  | Berte van Zeist-de Jonge  22-03-2024 |
| Design of the device can hinder patients during daily activities (too many cables, battery too big, stickers too tight).  (Becking-Verhaar 2023) | I find it user friendly, but patients find the battery on the arm too big, the stickers too tight on the skin, the cables inconvenient when washing, dressing and showering. And when sleeping, the chest unit bothers them. (8) | X |  |  | Berte van Zeist-de Jonge  22-03-2024 |
| Patients could become restless or obsessed with seeing their vital signs.  (Becking-Verhaar 2023) | Some patients get restless form the data they can see, the beeping of the device, let alone the many cords, adhesives and the fairly heavy device that was attached to the body. (8) | X |  |  | Berte van Zeist-de Jonge  22-03-2024 |
| Inconveniences when using the device: Low battery power, problem-solving difficulties, chest cables and ECG stickers that inadvertently detach too quickly, problems calibrating the device and patient-related factors (Becking-Verhaar 2023) |  |  |  | X | Berte van Zeist-de Jonge  22-03-2024 |
| The respondents would like to see the following improvements: softer, smaller and lighter materials and equipment (e.g., batteries), fewer cables, more robust cables in the thumb and chest sensors, less sensitivity to daylight in the thumb sensor and improved adherence and covering patches to shield the thumb sensor from daylight, better ECG-sticker adhesion, more hygienic thumb patches and longer battery life (Becking-Verhaar 2023) |  |  |  | X | Berte van Zeist-de Jonge  22-03-2024 |
| Nurses occasionally doubted the measured vital signs’ reliability. Doubts also arose due to problems connecting patients to ViSi Mobile and calibrating the device.  (Becking-Verhaar 2023) | Also the saturation often deviates or is not present. Why should I rely on the rest? (8) |  | X |  | Berte van Zeist-de Jonge  22-03-2024 |
| Most of the respondents commented that improved internet connectivity is a requirement. Therefore, respondents would like to calibrate and assign patients more easily and quickly by optimising internet connectivity. (Becking-Verhaar 2023) |  |  |  | X | Berte van Zeist-de Jonge  22-03-2024 |
| Several nurses mentioned training as one of the top three improvements that should be prioritised when working with ViSi Mobile. (Becking-Verhaar 2023) |  |  |  | X | Berte van Zeist-de Jonge  22-03-2024 |
| Nurses wanted to know what to do when ViSi Mobile did not work and how to identify malfunctions. (Becking-Verhaar 2023) | To my understanding, many patients aren’t connected because we, ourselves as nurses, can’t resolve the error messages. (9) | X |  |  | Berte van Zeist-de Jonge  22-03-2024 |
| Respondents were divided about how they would like to receive training. Most  respondents preferred a presentation, and others preferred e-learning or a combination of the two. Several nurses desired the ability to re-read instructions or training materials after training and the ability to ask questions during presentations. A minority wanted a paper reference manual. (Becking-Verhaar 2023) |  |  |  | X | Berte van Zeist-de Jonge  22-03-2024 |
| Common wishes were centralising materials storage, a mobile container with the necessary equipment to take into patients’ rooms and arrangements to optimise material and stock management and corresponding responsibilities. Respondents also desired sufficient functioning equipment and materials to replace broken parts. (Becking-Verhaar 2023) |  |  |  | X | Berte van Zeist-de Jonge  22-03-2024 |
| One of the challenges clinical staff faced was the decision of who should be set up on the virtual monitoring system, given the limited number of devices available. (Buss, 2023) | I think the monitoring system actually came at a handy time … we actually had a lot of COVID patients and at the time we were not really sure how to use, how to monitor these patients in terms of like how do we go in there frequently? Or you know how many times should we monitor them per day? We actually used it for all COVID patients that were isolating on high flow oxygen. Participant 001 (Ward manager) (p.2455) | x |  |  | Berte van Zeist-de Jonge  24-09-2024 |
| Many nurses identified this as challenging within their practice, requiring prioritization of vital signs observations within their workload. (Buss, 2023) | we would escalate somebody to use the system if on our general observations we noted that there was a deterioration, or we felt there was likely to be a deterioration. Participant 007 (Nurse) (p.2456) | x |  |  | Berte van Zeist-de Jonge  24-09-2024 |
| Staff described the system as valuable for watching the vital-signs trends of patients they were concerned about, while enabling them to continue with their clinical duties. (Buss, 2023) | It helped us to work without too much exposure, but I personally think it was very good if the patient also has like cardiac problems who need to be monitored more in our ward as we don't have those monitors. Participant 003 (Doctor) (p.2456) | x |  |  | Berte van Zeist-de Jonge  24-09-2024 |
| This was described as particularly useful when nursing staff had several unwell patients in their case load, to aid prioritization. (Buss, 2023) | The changes would prompt us to gown up and go in and see the patient so really, really helpful. Participant 014 (Consultant) (p.2456) | x |  |  | Berte van Zeist-de Jonge  24-09-2024 |
| Staff described becoming more selective in their use of the system over time, balancing the perception of increased patient safety with the drawbacks of the system. (Buss, 2023) | there were some patients for who it was useful but it was a double edged sword and so patients who were anxious about their oxygen levels, I found were watching the number on their wrist go up and down and that was clearly having a bit of an impact on them. Participant 006 (Nurse) (p.2456) | x |  |  | Berte van Zeist-de Jonge  24-09-2024 |
| They reported the system was less useful for some groups of patients, including those with cold peripheries (this impeded the oxygen saturation monitoring); patients who were con fused or restless; and those who were anxious about their oxygenation levels. (Buss, 2023) | the patients who are confused and restless or got cold hands or who is just fidgety and they just want to take it off … you find the monitoring on the floor or on the table because they just want it off. Participant 011 (Nurse) (p.2456) | x |  |  | Berte van Zeist-de Jonge  24-09-2024 |
| While the remote monitoring was perceived to assist the nursing staff, there were also limitations associated with the system use, so careful con sideration had to be made about which patients would benefit from it the most. (Buss, 2023) |  |  | x |  | Berte van Zeist-de Jonge  24-09-2024 |
| It was clear most of the clinical staff wished to ensure accuracy of the system when coming onto shift. Five nurses described how they would measure accuracy by comparing the virtual monitoring observations with their usual ward monitoring system. (Buss, 2023) | For me when I'm starting my shift and I have a patient on the device I will go in with the obs[ervation] ma chines as well just to double check to know what their reading is, and I don't see any difference. Participant 005 (Nurse) | x |  |  | Berte van Zeist-de Jonge  24-09-2024 |
| Most clinical staff interviewed highlighted the importance of hav ng confidence in what observations the system was displaying. (Buss, 2023) | Sometimes I go into the room, and I have the obs[er vation] machine with me and the monitor will be on and I check the sats [oxygen saturations] and every thing is accurate. Participant 004 (Healthcare assistant) | x |  |  | Berte van Zeist-de Jonge  24-09-2024 |
| Most clinical staff interviewed highlighted the importance of having confidence in what observations the system was displaying. There were times where a discrepancy arose, particularly with the saturation probe numbers. Interviewed nurses described wide variation in how they handled these discrepancies. (Buss, 2023) | Even though there was a discrepancy between satu rations it wasn't a significant difference so you could still get the readings and if the sats went down you could be pretty sure their sats were going down. Participant 008 (Nurse) Even though there was a discrepancy between satu rations it wasn't a significant difference so you could still get the readings and if the sats went down you could be pretty sure their sats were going down. Participant 008 (Nurse) | x |  |  | Berte van Zeist-de Jonge  24-09-2024 |
| With patient safety being the foremost thought of the clinical staff interviewed, trust in the observations was shown to really be a vital component underlying how clinical staff utilized the AMS in practice. (Buss, 2023) | The saturation we noticed it was a little bit lower than what other machines were reading so that was an other reason some people did not use them. Participant 007 (Nurse) if you get a false positive then the patient needs ob servation so it is better when they under read. Participant 008 (Nurse) | x |  |  | Berte van Zeist-de Jonge  24-09-2024 |
| Although there was local agreement that with the use of the AMS, the protocolized frequency of blood pressure monitoring could be reduced to lower the required room entries, many of the staff interviewed described discomfort with this. (Buss, 2023) | I think also weighing on our shoulders we're completely accountable for our patient care and I don't know, even if I'm told not to do something for my own benefit so that I know how my patient is I'm going to want to know exactly how they are. Participant 007 (Nurse) | x |  |  | Berte van Zeist-de Jonge  24-09-2024 |
| They emphasized the need for contact with patients to ensure safety and appropriate care delivery. (Buss, 2023) | I think because of the frequency of observations and the fact it was recording so frequently it took a lit tle bit of pressure off if you had multiple unwell pa tients yourself and you felt that you were keeping a closer eye on them because it was not practical to be dipping in and out of rooms when you've got six sick patients and that's a high ratio of patients to one that's not practical and you can't get PPE [Personal Protective Equipment] on, get in the room and do the observations, come out, act on them and go into the next one so it's really reassuring to be able to walk past, have a quick look at other patients and go okay prioritise or not. Participant 007 (Nurse | x |  |  | Berte van Zeist-de Jonge  24-09-2024 |
| Those who trusted the observations reported that the AMS assisted with managing their caseload as efficiently as possible by allowing them to assess observations without entering the patient's room. (Buss, 2023) | instead of going in and out every half hourly or hourly we can document and everything so it's really helpful in saving our time. Participant 012 (Nurse) | x |  |  | Berte van Zeist-de Jonge  24-09-2024 |
| When trying to manage high caseloads and rapidly deteriorating patients, most nurses reported that the AMS allowed for reas surance of patient stability and assisted with prioritization of pa tients. (Buss, 2023) | sometimes you forget and you need to do obs[erva tions] on this patient but you are so busy and if you can just glance at the big screen and the patient is okay … I can go to that patient later and I attend to some other patient who maybe needs pain medication. Participant 005 (Nurse) | x |  |  | Berte van Zeist-de Jonge  24-09-2024 |
| The use of the AMS evolved so that instead of predominantly being used as a way to reduce patient contact, it also informed the management of their workload. (Buss, 2023) | Yes, absolutely I think it's great to have it available just because of the practicalities of side room nursing and not even to do with COVID and PPE, it's just having the patient observed when you're not there, it's an other set of eyes on them really isn't it. Participant 007 (Nurse) | x |  |  | Berte van Zeist-de Jonge  24-09-2024 |
| Staff recognized ongoing benefits of the system on a ward where most patients were in side-rooms and therefore not easily visible. The system was used as an adjunct to regular manual observation measurements which included blood pressure and temperature, which the system did not offer. In this way, staff described being able to get a quick overview of the well-being of their patients. (Buss, 2023) | you couldn't really go into the room all the time you're trying to also you know you're trying to avoid going into the room all the time but then again you can ac tually sit down virtually, and you can actually observe and see how the patients are actually doing. Without you going there all the time. Participant 001 (Ward manager) | x |  |  | Berte van Zeist-de Jonge  24-09-2024 |
| Lack of available evidence to substantiate the use of CM with a limited number of vital signs in their patient population. (kooij, 2022) | We need to gain trust in the idea that heart rate and respiratory rate together provides sufficient information to conduct interventions. This is still difficult for me. (5) | X |  |  | Berte van Zeist-de Jonge  18-03-2024 |
| Measurements of vital signs by the sensor did often not correspond with measurements by another monitoring device used in daily practice. (kooij, 2022) |  |  |  | X | Berte van Zeist-de Jonge  18-03-2024 |
| Technical issues (kooij, 2022) |  |  |  | X | Berte van Zeist-de Jonge  18-03-2024 |
| Positive experiences with regards to early detection of deterioration. (kooij, 2022) | You have a continuous view on the patient. I think that is most important, you can detect early deterioration. (5) | X |  |  | Berte van Zeist-de Jonge  18-03-2024 |
| Advantages of CM patient safety, early discharge, (kooij, 2022) |  |  |  | X | Berte van Zeist-de Jonge  18-03-2024 |
| CM saves time as it eliminated the need for measuring vital signs manually during routine rounds. (kooij, 2022) |  |  | X |  | Berte van Zeist-de Jonge  18-03-2024 |
| Pilot of working with CM made possible to gain experience with CM. (positive) (kooij, 2022) | We conducted a pilot on the nursing ward.. I think for a certain number of patients. Based on that pilot we wanted to see if it would be meaningful. |  | X |  | Berte van Zeist-de Jonge  18-03-2024 |
| Pilot setting led to additional tasks and duplications in registration due to the use of multiple systems. (kooij, 2022) |  |  |  | X | Berte van Zeist-de Jonge  18-03-2024 |
| High complexity as strong negative influence on implementation. (kooij, 2022) |  | X |  |  | Berte van Zeist-de Jonge  18-03-2024 |
| Duration of the intervention relates to the additional time involved with using it, for example to attach and activate the sensor. (kooij, 2022) | First, we had to open the system, search for the patient in the system. That will already take approximately 5 minutes, so it takes extra time. (5) | X |  |  | Berte van Zeist-de Jonge  18-03-2024 |
| Nurses were not satisfied with the quality of the sensor. (kooij, 2022) | Our target population was sweating a lot after surgery, and we noticed the sensor would come off.. (5) | X |  |  | Berte van Zeist-de Jonge  18-03-2024 |
| Not satisfied with the quality of the system and data availability. (kooij, 2022) |  |  | X |  | Berte van Zeist-de Jonge  18-03-2024 |
| Nurses perceived that patients felt safer when they were monitored continuously (kooij, 2022) | There were also patients that felt safe: So you monitor my values 24 hours per day. So even if you are not in my room, you monitor me. That gave patients a feeling of safety. (6) | X |  |  | Berte van Zeist-de Jonge  18-03-2024 |
| Nurses perceived that patients were not burdened by the sensor. (kooij, 2022) |  |  | X |  | Berte van Zeist-de Jonge  18-03-2024 |
| Implementation: Facilitating factor to execute a task with a colleague. (kooij, 2022) | During the planned meetings we could get together and share experiences, we also had frequent mail contact but the moments together were the most pleasant. (6) |  | X |  | Berte van Zeist-de Jonge  18-03-2024 |
| Implementation: Nurses are positive about both formal communication (meetings) and informal communication with colleagues. (kooij, 2022) | During the planned meetings we could get together and share experiences, we also had frequent mail contact but the moments together were the most pleasant. (6) | X |  |  | Berte van Zeist-de Jonge  18-03-2024 |
| Nurses do not feel de need to change current situation (MEWS). They are satisfied with current monitoring (MEWS) (kooij, 2022) | These check-ups, the MEWS, are really useful during acute situations. You can really compare with other check-ups or with deteriorating patients, so I am used to working with the MEWS and I think it is quite nice. (6) | X |  |  | Berte van Zeist-de Jonge  18-03-2024 |
| Increased workload, in case of deteriorating vital signs, nurses needed to check the patients and perform extra check-ups. (kooij, 2022) | So at some point you could see a deviation in a patient, which you couldn’t see with your clinical view alone, but to really be sure how the patient was doing you still had to go and take the measurements. So that was an additional task. (6/7) | X |  |  | Berte van Zeist-de Jonge  18-03-2024 |
| Sensor limitations: could not measure blood pressure. Not usable for patients with pacemaker, CT scan, or when patient is taking a shower. (kooij, 2022) |  |  | X |  | Berte van Zeist-de Jonge  18-03-2024 |
| Clinical view is still needed in addition to CM. (kooij, 2022) |  |  | X |  | Berte van Zeist-de Jonge  18-03-2024 |
| The aim of CM is to detect deterioration and the prospect of early discharge with CM in the home setting. (kooij, 2022) | Eventually, the goal is to discharge a patient early and to monitor them at home. (7) |  | X |  | Berte van Zeist-de Jonge  18-03-2024 |
| Perceptions about the possibility to test the intervention and whether they felt safe to try the intervention and make mistakes varied (kooij, 2022) | It was a pilot and it was no direct risk for the patient. We also performed the normal checks, so you had a good view of the patient and patient safety was not at risk (7) | X |  |  | Berte van Zeist-de Jonge  18-03-2024 |
| There were sufficient additional resources such as a dedicated project team and technical support. (kooij, 2022) | There was a project team with supervisors and researchers and somebody form the technical department. (7) |  | X |  | Berte van Zeist-de Jonge  18-03-2024 |
| Access to a manual and training about CM was perceived helpful. (kooij, 2022) | The manual was changed frequently, with new tips and things. That was very useful! (8) |  | X |  | Berte van Zeist-de Jonge  18-03-2024 |
| Nurses were predominantly positive about continuous monitoring on the nursing ward. (kooij, 2022) | I think it is a very nice development. When I see it in practice, I think it could be possible. There are a lot of patients that could just ga home. (8) |  | X |  | Berte van Zeist-de Jonge  18-03-2024 |
| Personal characteristics affecting implementation such as younger age, experience with the intervention task will be beneficial, for example to execute tasks correctly and at a more rapid pace. (kooij, 2022) | The more often you do it, the easier it will become and you will get into a routine. (8) |  | X |  | Berte van Zeist-de Jonge  18-03-2024 |
| There was a formally appointed internal implementation leader, positive. (kooij, 2022) | The project leader was accessible, and visible on the nursing ward. I think that is important especially at the start, that somebody is always available to answer your questions. (8) | X |  |  | Berte van Zeist-de Jonge  18-03-2024 |
| Key-users were present for practical support (nurses with specific involvement in the project). (kooij, 2022) | We had key-users who helped at attaching and connecting the sensor. (9) | X |  |  | Berte van Zeist-de Jonge  18-03-2024 |
| Nurses were positive about the evaluation of the implementation. They used meetings or evaluation forms. It provided insights into the status of the project. (kooij, 2022) | We discussed it each day in the daily evaluation. How it is going, is the connection working, are the check-ups good, do you notice differences, do you feel positively of negatively about it. A lot of attention was paid to it. (9) | X |  |  | Berte van Zeist-de Jonge  18-03-2024 |
| Negative about technical infrastructure to support CM. Bad Wi-Fi connection and lack of interoperability with existing systems. (kooij, 2022) | The wi-fi connection was a problem. Sometimes the sensor did not connect and we had to restart the whole system. So that was the reason it did not work out. | X |  |  | Berte van Zeist-de Jonge  18-03-2024 |
| Nurses suggest they need additional parameters for CM (blood pressure or oxygen saturation). (kooij, 2022) |  |  |  | X | Berte van Zeist-de Jonge  18-03-2024 |
| Interoperability with existing systems is perceived important. This could reduce workload by eliminating the need to manually register the measurements. (kooij, 2022) |  |  | X |  | Berte van Zeist-de Jonge  18-03-2024 |
| Properly working and reliable technology. (kooij, 2022) |  |  | X |  | Berte van Zeist-de Jonge  18-03-2024 |
| Receiving training and education is conditional to acquire knowledge of the system and to be able to start with CM. (Leenen 2022(1)) | In the beginning I had to get used to it for a while and I still felt insecure about some aspects of continuous monitoring. But it helped that we just started doing it and having an involved project leader and key users. There was always an opportunity to ask questions and she was also often present in the department, so that you just become really confident in working with it. (4) | X |  |  | Berte van Zeist-de Jonge  19-03-2024 |
| Timing of training and dosage of the amount of information is considered important, preferably shortly before the start of the implementation and repeated regularly during implementation to keep their acquired knowledge up to date. (Leenen 2022(1)) | I think that you should also give proper education and training beforehand. But also providing extra training for the people who find if difficult in advance. For Example, by setting up a personal coaching plan for the nurse. So, you really have to spend time on one-on-one guidance in the first period, so that nurses feel heard (…) To be able to ask questions about your patient with CM to a colleague who knows te system well, that will get you going. (4) | X |  |  | Berte van Zeist-de Jonge  19-03-2024 |
| Feelings of insecurity in using the system. (Leenen 2022(1)) | In the beginning I had to get used to it for a while and I still felt insecure about some aspects of continuous monitoring. But it helped that we just started doing it and having an involved project leader and key users. There was always an opportunity to ask questions and she was also often present in the department, so that you just become really confident in working with it. (4) | X |  |  | Berte van Zeist-de Jonge  19-03-2024 |
| Coaching by the project leader and key-users is considered supportive for learning on the job. (Leenen 2022(1)) | To be able to ask questions about your patient with CM to a colleague who knows te system well, that will get you going. (4) | X |  |  | Berte van Zeist-de Jonge  19-03-2024 |
| Skills are best learned at the bedside. (Leenen 2022(1)) | I really think it would be difficult to work with CM. Because you really need the experience in real-life practice, with real patients, if you want to be able to work with this new device properly. (4) | X |  |  | Berte van Zeist-de Jonge  19-03-2024 |
| Training before the start of CM does not work without applying the new knowledge at the bedside. (Leenen 2022(1)) | To be honest, we had training before the start, but that did not really take root at the time. At the start of the implementation, I really think it would be difficult to work with CM. (4) | X |  |  | Berte van Zeist-de Jonge  19-03-2024 |
| Nurses prefer higher volume of patients with CM instead of a few. As a result they were working with two different work processes. (Leenen 2022(1)) | Yes, CM is something that if you want to perform well, I think you really should do it structurally. And I mean, just really work with the system every day with every patient. Not only with some of your patients. Then you will easily learn the system during a few shifts, just in your daily work. (5) | X |  |  | Berte van Zeist-de Jonge  19-03-2024 |
| Varying perspectives of interpreting vital sign trends. 1, able to asses properly, 2, difficult because of the lack of knowledge about normal vital sign trends. (Leenen 2022(1)) | I think it is quite hard in the beginning, because you do not know what a vital sign trend should look like. Especially when taking the patient status, activity and missing data in the trend into account. Those factors are important to consider when assessing the trend. (5) | X |  |  | Berte van Zeist-de Jonge  19-03-2024 |
| Clear protocol for interpreting vital sign trends is considered useful by nurses. (Leenen 2022(1)) | Nowadays we work with the EWS. Those are recognizable and guiding in our follow-up actions, like calling a physician when a score is 5. The trends and thresholds did not provide such clear follow-up. Also because CM still does not measure all the vital signs to generate a proper EWS. (5) | X |  |  | Berte van Zeist-de Jonge  19-03-2024 |
| Full range of vital signs is needed to measure an EWS, measuring more vital signs provides a more complete insight in the clinical status of the patient. Nurses miss measurements. (Leenen 2022(1)) | Nowadays we work with the EWS. Those are recognizable and guiding in our follow-up actions, like calling a physician when a score is 5. The trends and thresholds did not provide such clear follow-up. Also because CM still does not measure all the vital signs to generate a proper EWS. (5) | X |  |  | Berte van Zeist-de Jonge  19-03-2024 |
| Collaboration with physicians vitally important for successful interpreting the trends and the follow-up. (Leenen 2022(1)) | Besides trend assessment by us as nurses, physicians must be involved. They need to know how to act based on deviating trends. Eventually, they are responsible for the medical policy following the trend. (5) | X |  |  | Berte van Zeist-de Jonge  19-03-2024 |
| Nurses place trends in the perspective of their clinical assessment. (Leenen 2022(1)) | Yes, I think I should see CM as a helpful tool. I don’t see it as a substitute for me as a nurse, like: Oh, that one patient has a wireless VSM and I an blindly rely on those measurements. But your own clinical assessment of the patient besides vital signs remains most important. (5) | X |  |  | Berte van Zeist-de Jonge  19-03-2024 |
| Nurses think it will be helpful to learn to interpret vital sign trends when a patient deteriorates while having CM. (Leenen 2022(1)) | I think it is helpful if you cared for a patient that had an acute clinical deterioration. Then you possibly have a clear picture of such a deviating vital sign trend in combination with the clinical status of the patient. (6) | X |  |  | Berte van Zeist-de Jonge  19-03-2024 |
| CM may contribute to earlier detection of clinical deterioration by better insight into the vital sign trends and thus increase the safety of care. (Leenen 2022(1)) | I think it can offer a lot for us and patients, especially if you are able to detect the complications earlier. By the insight in trends you may detect clinical deterioration earlier between the routine measurements. (6) | X |  |  | Berte van Zeist-de Jonge  19-03-2024 |
| Nurses consider there should be a clear rationale to measure vital signs at a high frequency. Patients with high risk of clinical deterioration have the best benefits of CM. (Leenen 2022(1)) | I would not see much added value for low-complexity care. These patients already have a low risk of complications and so clinical deterioration of vital signs. For example, consider an appendectomy. (6) | X |  |  | Berte van Zeist-de Jonge  19-03-2024 |
| Costs of implementation of CM systems should be in proportion to the benefits for patient care. (Leenen 2022(1)) | If the wearable sensor is very expensive, it is worth considering whether the investment is worth it for the particular patient group. I do not think it is effective to apply on those low-complex care patients. (6) | X |  |  | Berte van Zeist-de Jonge  19-03-2024 |
| CM is especially useful during night shifts because of the higher patient-to-nurse ratio and the desire not to wake the patient (Leenen 2022(1)) | During the night shift you have a direct insight and an overview whether each patient is still breathing or showing abnormalities in vital signs. This is really helpful when you nearly have a half ward of patients to take care of (6) | X |  |  | Berte van Zeist-de Jonge  19-03-2024 |
| The system generated too many and too many false alarms. These alarms were experienced as disruptive and caused feelings of uncertainty and lead to irritation. (Leenen 2022(1)) | I found the number of alarms that you got on your telephone the most inconvenient for me. There were really too any. This was often already with a deviation or technical problem for a short time. For instance, when you support in mobilization, you don’t have time to check the notification on your phone every time. You can’t leave the patient at all at that moment so an alarm does not add up to better care. Sometimes I was happy when the alarms didn’t ring for a while. (6) | X |  |  | Berte van Zeist-de Jonge  19-03-2024 |
| Feeling of agitation about the alarms, potentially related to the extra workload caused by the need to respond to the alarms. (Leenen 2022(1)) | Often as a nurse you could not do anything with the alarm because the heart rate had already dropped again of the connection had already been restored. (7) | X |  |  | Berte van Zeist-de Jonge  19-03-2024 |
| Feelings of uncertainty raised by alarms because of having doubts about their own clinical experience by receiving multiple and frequent alarms. (Leenen 2022(1)) | Then you start doubting whether you are doing your work right or not missing any abnormalities in the patient condition (7) | X |  |  | Berte van Zeist-de Jonge  19-03-2024 |
| Nurses suggest user-adjustable alarm settings to decrease false alarm rate and prevent alarm fatigue. (Leenen 2022(1)) | Also, adjusting values to the specific patient could be helpful in reducing alarms. (7) | X |  |  | Berte van Zeist-de Jonge  19-03-2024 |
| Nurses preferred a CM system technically integrated into their existing mobile devices without restrictions in the range of the wireless connection. (Leenen 2022(1)) | Also receiving alarms on the calling system instead od using a separate phone. This makes everyday use much easier. (7) | X |  |  | Berte van Zeist-de Jonge  19-03-2024 |
| Nurses favour integration of vital sign trends into the EMR allowing more effective documentation, evaluation and productivity. (Leenen 2022(1)) | It does work better for me if we can assess the trends in the current used systems such as the EMR. (7) | X |  |  | Berte van Zeist-de Jonge  19-03-2024 |
| Cm should not lead to earlier discharge of patients from the ICU. Reason 1: Nurses fear that this might result in a higher workload and unsafe nursing care. Reason is the inability to respond to alarms immediately. (Leenen 2022(1)) | If an alarm rings from one patient and at the moment you are bathing a patient and you also have to care for four other patients, then responding to the alarm can be challenging. I think that’s different on an ICU. (7) | X |  |  | Berte van Zeist-de Jonge  19-03-2024 |
| Reason 2: high workload because of lower nurse-patient ratio. Also they believe not to have the technical nursing skills an knowledge of CM that ICU patients would need. (Leenen 2022(1)) | CM should not be a reason for patients to be discharged from the ICU to our ward earlier. We care for many more patients per nurse and in case of acute deterioration we do not have the same resources. (7) | X |  |  | Berte van Zeist-de Jonge  19-03-2024 |
| Nurses hope CM can reduce workload of current routine manual measuring and registering vital signs, allowing them to be more productive and have more dedicated time for patient care. (Leenen 2022(1)) | I hope in the future wearable sensor will measure the full spectrum of vital signs so I don’t have to collect them manually several times a day. This will save time which I can still devote to many other tasks during a busy shift. (7) | X |  |  | Berte van Zeist-de Jonge  19-03-2024 |
| Nurses are able to detect deviations of vital signs earlier using regular trend analysis and recognized the importance of vital sign trends over the intermittent vital sign manual measurements, because of the insight in the periods between intermittent measurements, especially during the night. (Leenen 2022(2)) |  |  | X |  | Berte van Zeist-de Jonge  19-03-2024 |
| CM enables them to act earlier on deviating vital signs than when using intermittent monitoring alone. (Leenen 2022(2)) |  |  | X |  | Berte van Zeist-de Jonge  19-03-2024 |
| CM enabled them to better monitor the effect of interventions in vital signs. (Leenen 2022(2)) | After each administration of metoclopramide, we observed an abnormality in the heart rate trend, which ultimately led the doctors to stop de administration of this drug. (6) | X |  |  | Berte van Zeist-de Jonge  19-03-2024 |
| It is necessary to take clinical status and context factors into account when assessing the vital sign trend, rather then just acting solely on the trend data. (Leenen 2022(2)) | For example, when the patient is washing and dressing in the morning, you expect a higher breathing and heart rate. In that case this is not clinically relevant and you should not take any action. (6) | X |  |  | Berte van Zeist-de Jonge  19-03-2024 |
| Nurses preferred more guidance, when there were problems with the technology. (Leenen 2022(2)) |  |  | X |  | Berte van Zeist-de Jonge  19-03-2024 |
| Teaching-on-the-job by the researcher was desirable for adoption of the technology. (Leenen 2022(2)) |  |  | X |  | Berte van Zeist-de Jonge  19-03-2024 |
| Importance of experiencing an adverse event when continuous monitoring was applied. (Leenen 2022(2)) | If you once had a patient who developed a complication and that deterioration was reflected in the vital signs trends; that experience in the trend assessment is important and you are easily convinced of the added value of CM. (7) | X |  |  | Berte van Zeist-de Jonge  19-03-2024 |
| Nurses mention the importance of automated integration of continuous vital sign data in de EMR. (Leenen 2022(2)) |  |  | X |  | Berte van Zeist-de Jonge  19-03-2024 |
| Clinical decision support is helpful for trend assessment, especially the D-EWS scores which were closely related to their conventional way of interpreting vital values with the MEWS system. (Leenen 2022(2)) | It is recognizable and corresponds to the usual working method with the EWS. This makes it easier for me to consider whether the trend actually deviates and promotes communication with the doctor when needed. (8) | X |  |  | Berte van Zeist-de Jonge  19-03-2024 |
| Multidisciplinary responsibility for monitoring vital signs important for their willingness to use the CM system. (Leenen 2022(2)) |  |  | X |  | Berte van Zeist-de Jonge  19-03-2024 |
| Communication and education about the technology and work process to all stakeholders was important. (Leenen 2022(2)) | It worked for me when I received explanation and education about the possible benefits of adding CM. (8) |  | X |  | Berte van Zeist-de Jonge  19-03-2024 |
| Vital signs values and trends must be measured reliably and the technology must not be defective. (Leenen 2022(2)) |  |  | X |  | Berte van Zeist-de Jonge  19-03-2024 |
| Practical experience was convenient for their adoption and acceptability of the intervention. (Leenen 2022(2 |  |  | X |  | Berte van Zeist-de Jonge  19-03-2024 |
| Analysis of trends required experience because they were only used to interpret absolute values of the intermitting measurements of vital signs. (Leenen 2022(2)) |  |  | X |  | Berte van Zeist-de Jonge  19-03-2024 |
| There is no added value of alarms if trend analysis was carried out according to the protocol used in this study. (Leenen 2022(2)) | If every nurse is assessing the trend and reporting it adequately in their shift, then I think receiving an alarm when the trend are deviating is unnecessary. | X |  |  | Berte van Zeist-de Jonge  19-03-2024 |
| Nurses do not want alarms (Leenen 2022(2 | Especially because we already have al lot of distractions and interruptions when caring for patients, like calls by patients or other healthcare professionals. | X |  |  | Berte van Zeist-de Jonge  19-03-2024 |
| Alarms are only desirable when they are fully reliable and not generating frequent false alarms and an alarm should require immediate follow-up by the nurse, such as taking extra vital sign measurements or notifying a doctor. (Leenen 2022(2)) | I wonder if this would work in practice. The clinical judgement of us nurses is also important in this regard. In addition, we also have to care for many more patients then our colleagues in the ICU, which means that following up an alarm is different then in a high care department. | X |  |  | Berte van Zeist-de Jonge  19-03-2024 |
| Prioritization of CM depends on the caseload during the shift. (Leenen 2023) | Yes, I think it is when the workload is high, and then it easily forgotten because of it is not your priority to check and report the trend. If it’s just a quiet shift, then it’s easier to perform. (13) | X |  |  | Berte van Zeist-de Jonge  19-03-2024 |
| Workload varied by type of shift, day shifts higher then night shifts. Actual intervention fidelity was not better during evenings or night. (Leenen 2023) | During night shifts, I do not assess the vital signs trends because patients are supposed to be asleep end the standard manual measurement rounds are enough to assess their condition properly (13) | X |  |  | Berte van Zeist-de Jonge  19-03-2024 |
| Nurses experience CM as a relatively unnecessary addition to their manual measurements, especially during morning rounds, when the priority for additional trend assessment was lower. (Leenen 2023) | Because in the morning you still measure your vital signs with the spot-check monitoring and then CM is on top of that. I am able to perform without those trends. (13) | X |  |  | Berte van Zeist-de Jonge  19-03-2024 |
| Less need for assessing the vital sing trends in patient with an uncomplicated course. (Leenen 2023) | If I only just once had a case where you can actually see deviating trends, then you’ll probably use CM better. My experience is (mainly) with stable patients who have CM that shows the same trends over three consecutive shifts; I think in that case actual use and usefulness fades a bit. (13) | X |  |  | Berte van Zeist-de Jonge  19-03-2024 |
| When clinically relevant, correct assessment of trends was performed better. (Leenen 2023) | If I only just once had a case where you can actually see deviating trends, then you’ll probably use CM better. My experience is (mainly) with stable patients who have CM that shows the same trends over three consecutive shifts; I think in that case actual use and usefulness fades a bit. (13) |  | X |  | Berte van Zeist-de Jonge  19-03-2024 |
| Importance of their clinical bedside assessment. (this is more then only vital sign measurement) (Leenen 2023) | During rounds we assess more than just measuring the values of the vital signs. For instance, in patients with oxygen supplementation, you really want to know what that the breathing looks like.. Besides, by talking to the patient you can also obtain a more comprehensive impression of the patient who is lying in bed. (13) | X |  |  | Berte van Zeist-de Jonge  19-03-2024 |
| Trends were often a confirmation of their clinical perspective of the patient rather than it prompting them to reconsider their assessment. (Leenen 2023) | I do find that when a patient is more ill, you assess the CM more often.. But I do not often experience that it really detects something I did not know yet. However, I think it’s a very nice addition to our work and may possibly stimulate clinical reasoning; especially for young nurses. (13) | X |  |  | Berte van Zeist-de Jonge  19-03-2024 |
| Nurses do not fully trust the accuracy of the technology without physically assessing the patient. (discrepancies between what they observed and what the trend indicated) (Leenen 2023) | And you have to compare trends to the patient context. For instance, with the respiratory rote. You have to verify if the patient is mobilized and assess if the trend deviation is clinically relevant. (13) | X |  |  | Berte van Zeist-de Jonge  19-03-2024 |
| Cm provides more insights into the patient’s clinical status, especially during night shifts and patients who are critically ill. (Leenen 2023) |  |  |  | X | Berte van Zeist-de Jonge  19-03-2024 |
| Nurses have limited, or no experience with deviating vital sign trends and taking action on them. Therefore, nurses questioned whether proactive trend assessment was feasible as standard care as, in many cases, it did not alter their nursing care at the time. (Leenen 2023) | You have to assess regularly with most of the time not performing any actions based up on the trends. In my opinion, this does not bring any benefit to the patient, nor to us as professionals. (13) |  | X |  | Berte van Zeist-de Jonge  19-03-2024 |
| When witnessing deviating trends and taking action as a result, the added value of CM had become clearer afterward. (Leenen 2023) | I had a patient during my night shift with deviating trends, so I did an extra check and administered additional pain medication. (14) | X |  |  | Berte van Zeist-de Jonge  19-03-2024 |
| Pairing of the sensor with the software platform is a barriers for regular daily use (it uses a separate web-based application rather than via the regularly used phone). (Leenen 2023) | Sometimes the separate mobile phone with the specific codes malfunctions and it simple takes too much time, which eventually results in that you leave it at that. (14) | X |  |  | Berte van Zeist-de Jonge  19-03-2024 |
| Visibility of the trends was not convenient because software for assessing trends not integrated in patient file. (Leenen 2023) |  |  |  | X | Berte van Zeist-de Jonge  19-03-2024 |
| Removing the sensor when performing diagnostics for the prevention of interference was considered a barrier. (ill patients) (Leenen 2023) | It is annoying when a sick patient has to go for a scan and then just at that important moment, the sensor must be removed. (14) | X |  |  | Berte van Zeist-de Jonge  19-03-2024 |
| Sensor needs to be able to measure more vital signs than only HR and ReR to result in time-saving benefits, although it would not eliminate the need and value of bedside nursing assessments during rounds. (Leenen 2023) | It would help enormously (all data and trends visible in the HER), but even if everything is measured automatically, you still have to go and assess the patient yourself. (14) |  | X |  | Berte van Zeist-de Jonge  19-03-2024 |
| Specific alarm strategies for deviating trends could be an alternative to timely detect deterioration. (Leenen 2023) | Yes I also hear my colleugues about it: when scoring a (MEWS of) 3 or higher, they do not perform repeat measurements because the respiratory rate is normal for this patient. I do think it’s sometimes way too sensitive for a lot of patients. |  | X |  | Berte van Zeist-de Jonge  19-03-2024 |
| The use of assistive technology is desirable for the future of nursing care, considering the enrichment of nursing care and in view of future challenges in terms of capacity shortages. (Leenen 2023) | I do support the inclusion of technology and innovation in nursing care. I think we still integrate technology too little and therefore we are less familiar with it in nursing care. Support by technology can bring so much, and I think my colleagues sometimes forget that. | X |  |  | Berte van Zeist-de Jonge  19-03-2024 |
| Poor Wi-fi connection. (Van Noort, 2024) |  |  |  | x | Berte van Zeist-de Jonge  17-03-2024 |
| Battery too big, hampered the patient in daily activities. (Van Noort, 2024) | The device of the Visi Mobile is unfriendly for patients because the battery is too rude, and heavy. It never fits well, turns around my wrist and slides back and forth. It is not comfortable. (5) | X |  |  | Berte van Zeist-de Jonge  17-03-2024 |
| Incorrect cables led to measurement errors and false alarms. (Van Noort, 2024) |  |  | X |  | Berte van Zeist-de Jonge  17-03-2024 |
| Blood pressure not always a reliable estimation of the parameter. (Van Noort, 2024) |  |  | X |  | Berte van Zeist-de Jonge  17-03-2024 |
| Overload of data due to CM.  It enables nurses to make trend analysis, but this was still far from optimal. (Van Noort, 2024) | In the future, there may be a possibility to apply AI to handle and cluster the data overload. (5) | X |  |  | Berte van Zeist-de Jonge  17-03-2024 |
| Nurses perceived CM as an extra set of eyes, enabling them to provide better care. (Van Noort, 2024) | From a distance, you can estimate how your patient is doing, to some extent. Also, if your patient does not feel well, and you really want to be in the room all the time, which is not possible in a nursing ward, you feel you can better monitor your patient. So yes, it provides me a save feeling. | X |  |  | Berte van Zeist-de Jonge  17-03-2024 |
| CM provides a feeling of trust. (Especially during the night). (Van Noort, 2024) |  |  | X |  | Berte van Zeist-de Jonge  17-03-2024 |
| Nurses do not get a warning in case of abnormal vital signs of other patients, this gives a feeling of uncertainty. (Van Noort, 2024) | Recently, we had transferred a patient from the ICU to our unit, having a NEWS 6 and respiratory unstable. In that case, it would be great if there is someone who keep and extra eye on that patient, because I cannot constantly look to the display, and do not get an alarm on my pager. | X |  |  | Berte van Zeist-de Jonge  17-03-2024 |
| Deterioration is notices earlier with CM. (Van Noort, 2024) |  |  | X |  | Berte van Zeist-de Jonge  17-03-2024 |
| Nurses gained knowledge about working with continuous monitoring. (Van Noort, 2024) |  |  | X |  | Berte van Zeist-de Jonge  17-03-2024 |
| They now understand all types of and reasons for alarms and that they handled the continuous data availability of vital signs efficiently. (Van Noort, 2024) | Nin out of ten times you do not have to respond to a false alarm, but you just wait a few seconds before breathing frequency or saturation will improve. I have the idea we are on the right track in recognizing false alarms. | X |  |  | Berte van Zeist-de Jonge  17-03-2024 |
| Knowledge regarding alarms must be integrated into training for nurses. (Van Noort, 2024) |  |  | X |  | Berte van Zeist-de Jonge  17-03-2024 |
| Attitudes changed over time from sceptical to enthusiastic. CM became and essential part of care which facilitated them in anticipating patients’ clinical deterioration. (Van Noort, 2024) | How sceptical we were about CM. And now, three years later, we cannot work without it. (5) | X |  |  | Berte van Zeist-de Jonge  17-03-2024 |
| Over-monitoring, measuring vital signs when the clinical added value was not clear, did provoke a tired feeling towards CM. (Van Noort, 2024) | Some patients will be discharged soon, for example today or tomorrow. Why should you still monitor all vital signs and check trends? (5) | X |  |  | Berte van Zeist-de Jonge  17-03-2024 |
| Longer use of CM affects their alertness to changes. Critical note towards the possibility of only analysing trends, because small changes may be overlooked. (Van Noort, 2024) | Sometimes, a saturation drops during the night as it also does at home. We check on the patient because of this saturation drop and then the patient seems to be okay. (5) | X |  |  | Berte van Zeist-de Jonge  17-03-2024 |
| Nurses express the need to investigate how patients use the CM system, to be able to supervise them better. (Van Noort, 2024) |  |  | X |  | Berte van Zeist-de Jonge  17-03-2024 |
| Nurse-assistants could make an assisting contribution to the measurements of vital signs monitoring. (Van Noort, 2024) | We can say that connecting the device can be a task for nurse assistants. It will give a nice touch to their job profile. It seemed that some nurse assistants do really like that, and they see it as a challenge. (5) | X |  |  | Berte van Zeist-de Jonge  17-03-2024 |
| Nurses are not able to continuously watch the vital signs, as they were not all the time in one of the rooms where the dashboards were available. (Van Noort, 2024) | I would like to maintain CM, but I would also like to retain total care for my patients, without shifting tasks. That is very important to me. (5) | X |  |  | Berte van Zeist-de Jonge  17-03-2024 |
| Dedicated nurse could be a supportive role for ward nurses by making them aware of abnormalities. Can be helpful for unexperienced nurses, but other want to have the final responsibility for themselves. (Van Noort, 2024) | I think, for example, during evening and nightshifts we are much of our time present in the nursing office. During that shift, we do not need a dedicated nurse. During day shifts, when everyone is at the patient’ rooms, I think a dedicated nurse is necessary. The question rises if you can deploy the dedicated nurse in patient care, and that he also receives all alarms, so that he can respond to the alarms. (5) | X |  |  | Berte van Zeist-de Jonge  17-03-2024 |
| General ward is organized different then an ICU, therefore CM is different. A clear definition of what CM of vital signs a general wards is, and what kind of boundaries are determined, would enhance clarity as to the expectations towards nurses. (Van Noort, 2024) | The difference between a high care ward and our general ward is getting smaller using this system. Subsequently, it is difficult to set boundaries, and to frame, between what you should do and not do. (5) | X |  |  | Berte van Zeist-de Jonge  17-03-2024 |
| Alarms are a positive effect of CM using wearable devices. (Weenk 2020) | We should all receive a mini-Ipad. It can show us patients’ vital signs during our shift and will send us an alert in case the vital signs drop outside the normal ranges. (5) | X |  |  | Berte van Zeist-de Jonge  19-03-2024 |
| Clinical deterioration can be detected in an earlier phase using CM. (Weenk 2020) |  |  | X |  | Berte van Zeist-de Jonge  19-03-2024 |
| Earlier detection can result in earlier interventions. (Weenk 2020) |  |  | X |  | Berte van Zeist-de Jonge  19-03-2024 |
| CM can save time. (Weenk 2020) | Just talking to the patient. To have more time for the story of the patient. (5) | X |  |  | Berte van Zeist-de Jonge  19-03-2024 |
| CM increases the feelings of safety. (Weenk 2020) | Postoperative patients have been monitored continuously at the ICU. Some do feel unsafe after return at the general ward because of a lower number of vital sign measurements. (5) | X |  |  | Berte van Zeist-de Jonge  19-03-2024 |
| Nurses encourage the implementation of wearable devices for CM of patients. (Weenk 2020) | This is the future. We have to deal with it and the sooner we start working with those wearable devices, the more profit we will have. (5)  The future.. I think only 30% of the patients will be hospitalized by then. Patients will be monitored from home with this kind of smart devices. | X |  |  | Berte van Zeist-de Jonge  19-03-2024 |
| CM can generate an overload of information. (Weenk 2020) | Sometimes you just do not want to know, making yourself crazy with too much data. Particularly when data does not influence your decision in patient’s treatment. (5) | X |  |  | Berte van Zeist-de Jonge  19-03-2024 |
| Nurses are afraid the ward would become like an ICU.(CM can lead to reluctance in transfer to the ICU) (Weenk 2020) |  |  | X |  | Berte van Zeist-de Jonge  19-03-2024 |
| Nurses are afraid that interaction between patient and HCP would be reduced. (Weenk 2020) | You need the confidence from the nurses, I would miss that. However, quantity time might become quality time. (5) | X |  |  | Berte van Zeist-de Jonge  19-03-2024 |
| CM would cost more time and increase workload. (Weenk 2020) | Maybe it will increase work load. What if you receive an alarm every time a patient falls asleep and the oxygen saturation decreases a little bit (6) | X |  |  | Berte van Zeist-de Jonge  19-03-2024 |
| Patients can become worried by being able to see their own vital signs. (Weenk 2020) | Some people are very anxious. Like my wife.. like she already said: She would overreact. I would like to know my vital signs, but she would panic. (6) | X |  |  | Berte van Zeist-de Jonge  19-03-2024 |
| Because of CM HCP are able to see trends in vital signs. (Weenk 2020) | Last night we saw a patient with an EWS of 3 and in the morning it suddenly was 13. Using CM, we would have been able to see the EWS slowly increasing during the night (6). | X |  |  | Berte van Zeist-de Jonge  19-03-2024 |
| Opposite opinions about practicality of the device (small, but wrist device to big and heavy). (Invisible under clothes, cable and electrodes patches) (Weenk 2020) | Yesterday I felt very ill. I noticed that when you do not feel very well, every line, every device is just too much. (6) | X |  |  | Berte van Zeist-de Jonge  19-03-2024 |
| Device is not able to measure all the vital signs. (like core temperature) (Weenk 2020) |  |  | X |  | Berte van Zeist-de Jonge  19-03-2024 |
| Nurses thought that there would not be enough personnel to monitor all data. (Weenk 2020) | At this moment it is not feasible to monitor all patients 24 hours a day and to anticipate adequately to clinical deterioration with the amount of nursing staff we have. (6) | X |  |  | Berte van Zeist-de Jonge  19-03-2024 |
